# Balance and proprioception impairment, assessment tools, and rehabilitation training in patients with total hip arthroplasty: a systematic review

**DOI:** 10.1186/s12891-021-04919-w

**Published:** 2021-12-20

**Authors:** Luciana Labanca, Francesca Ciardulli, Fabio Bonsanto, Nadia Sommella, Alberto Di Martino, Maria Grazia Benedetti

**Affiliations:** 1 Physical Medicine and Rehabilitation Unit, IRCCS – Istituto Ortopedico Rizzoli, Via Giulio Cesare Pupilli 1, 40136 Bologna, Italy; 2 Department of Biomedical and Neuromotor Sciences, University of Bologna, Bologna, Italy; 3 I Orthopaedic Clinic, IRCSS- Istituto Ortopedico Rizzoli, Bologna, Italy

**Keywords:** Total hip replacement, Hip surgery, Postural stability, Functional assessment, Risk of falls

## Abstract

**Background:**

Osteoarthritis and subsequent total hip arthroplasty (THA) lead to damages to hip joint mechanoceptors, which in turns lead to impairments in proprioception. One of the abilities mainly affected by an altered joint proprioception is balance. The aim of this work was to investigate the balance and proprioception impairments, current assessment tools, and rehabilitation training after THA.

**Methods:**

A systematic literature revision was conducted on PubMed, Web of Science and Cochrane databases. Articles reporting balance and proprioception impairments, current assessment tools, or rehabilitation interventions were included. Methodological quality was assessed using the Downs and Black checklist. A total of 41 articles were included, 33 discussing balance and proprioception assessment, and 8 dealing with training. Data related to type of surgical approach, type and timing of assessment protocols, assessment instrumentation, and type, volume and duration of the rehabilitation training were extracted from each study.

**Results:**

Thirty-one studies were of high quality, 2 of moderate quality and 8 of low-quality. Literature review showed an improvement in balance following THA in comparison with the pre-operative performance, although balance abnormalities persist up to 5 years after surgery, with THA patients showing an increased risk for falls. Balance training is effective in all the rehabilitation phases if specifically structured for balance enhancement and consistent in training volume. It remains unclear which assessments are more appropriate for the different rehabilitation phases, and if differences exist between the different surgical procedures used for THA. Only two studies assessed proprioception.

**Conclusion:**

Balance and proprioception show impairments up to 5 years after THA, increasing the risk of falls. However, patients with THA may benefit of an adequate balance training. Further research is needed to investigate the gaps in balance and proprioception assessment and training following THA surgery.

**Supplementary Information:**

The online version contains supplementary material available at 10.1186/s12891-021-04919-w.

## Background

Total hip arthroplasty (THA) is the most used treatment for severe hip osteoarthritis, leading patients to an immediate decrease of pain and to consistent improvements in hip joint function and quality of life [1–3]. However, osteoarthritis and major surgical interventions for joints replacement may compromise part of the joint structures and surrounding components, with joint mechanoceptors being the most affected structure by THA surgery. The damages to these mechanoceptors lead to impairments in proprioception, i.e., to a lack or to abnormal afferent signals informing the brain of joints’ position and movement. Abnormal proprioceptive signals do not only affect sensory function, but also motor control, since sensory information is essential for movement programming [4]. For these reasons, patients undergoing joint replacement surgery show both sensitive and motor abnormalities [2, 5, 6]. One of the “abilities” mainly affected by abnormal proprioception is balance, and its impairments may compromise the quality of life [7–9], and the risk of falls [10]. In THA patients, balance deficits may be persistent after surgery [11, 12], leading to an increase in the risk of falls [13], especially in the first year after surgery [14].

The abnormalities in proprioception may also affect the biomechanics of functional movements. In fact, if afferent information is lacking, movements cannot be controlled across the whole range of motion of the joint. It is not surprising that following THA, patients show gait abnormalities up to 1 year after surgery [2]. In particular, a reduction of gait velocity and stride length have been reported, accompanied by a reduction of the time of single limb support, and a reduced range of motion in the sagittal plane [2].

Different surgical approaches for THA implant may affect hip proprioception in different ways. In fact, while the direct anterior approach (DAA) affects only hip joint capsule [15], other procedures, such as the lateral and posterior techniques, may also affect muscles and tendons [15] causing higher damages to proprioceptors. However, while the effect of different surgical approaches on hip biomechanics and clinical outcomes has been widely studied [16–19], the entity of proprioception compromise in this setting has not been extensively explored so far.

Moreover, few studies have been reported by previous systematic reviews on the benefits of balance training following THA [20],and it is not clear which kind of exercises should be adopted and how these should be differentiated in the different rehabilitation phases after THA surgery.

Having clear information on the magnitude of balance and proprioception impairments following THA would be of high clinical relevance, to ascertain which kind of balance and proprioception training interventions should be adopted, and how deficits and improvements throughout rehabilitation should be assessed.

Therefore, the current study has three main objectives. The first is to provide an updated systematic review on balance and proprioception impairments following THA surgery. The second is to investigate how balance and proprioception deficits are measured. The third objective is to investigate how balance and proprioception are trained during the rehabilitation following THA. These three points will be investigated by differentiating results according to the surgical approach used for THA implant.

## Methods

### Population and diagnosis of interest

This review includes all studies on balance and proprioception impairments, assessment or training after THA for degenerative arthritis.

### Search strategy and inclusion criteria

A systematic review of PubMed, Web of Science, and Cochrane database was performed. The inclusion criteria were: (1) articles published between June 1, 2000, and August 31, 2021; (2) patients with THA for degenerative arthritis were recruited, (3) assessment of balance and/or proprioception, and (4) training of balance and/or proprioception. No limitations were placed over the type of surgical procedure used for THA. Non-English language publications, review articles, conference proceedings, editorials, case-studies, letters, methodological studies, animal studies, and cadaveric studies were excluded. A time frame of 20 years was chosen for two main reasons. The first, is related to the fact that methodological literature suggest to write reviews based on nearly the last 5–10 years to be considered up-to-date [21, 22]. Then the suggested timing was extended to 20 years to include a broader number of evidences. The second reason is related to the to the fact that studies older than 20 years may have been based on surgical procedures, as for example high invasive approaches requiring long healing time, which are no longer adopted in clinical practice to date [23]. As a consequence, the post-surgical assessment, in particular in the early post-surgery was mainly based on surgery-related consequences (e.g, pain, bleeding, …) rather than on the assessment of functional abilities having an impact on the quality of life [23].

The terms and key words used for the research strategy were: (total hip arthroplasty OR THA OR hip replacement OR hip prosthesis) AND (balance OR propriocept* OR postural control) located within the title and/or abstract and/or keywords. The character * was used to include in the research both the terms proprioceptive, proprioception, and proprioceptors. Reference lists and citations of the included articles were manually screened to identify additional studies of interest. The Preferred Reporting Items for Systematic Reviews and Meta-Analyses (PRISMA) checklist was employed to guarantee review methodological quality (Additional file 1: Appendix 1). The study was registered in PROSPERO (CRD42020213412).

### Selection process

**Fig. 1:**
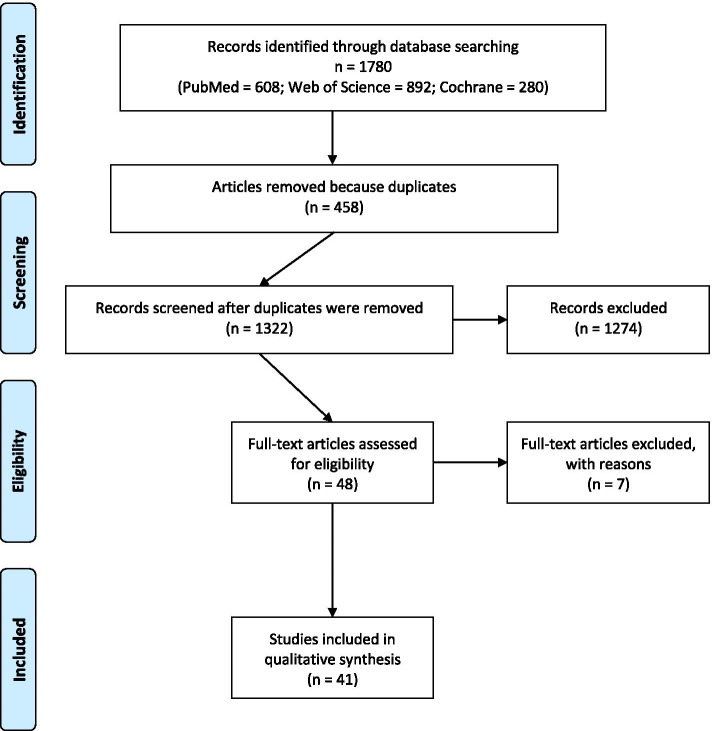
PRISMA 2009 Flow-chart of articles selection

Three independent reviewers (FC, FB, NS) performed the research and selection of the papers. Duplicates were removed and then the titles and abstracts of all the studies were reviewed to determine their eligibility. In case of disagreement in the appropriateness of the paper, a fourth author (LL) was consulted to determine abstracts inclusion; in that case, the full-text version of the paper was retrieved and screened to determine the eligibility of the paper. Then, the full-text of all the eligible articles was retrieved and assessed to further verify inclusion and exclusion criteria meeting. Selection process of the papers’ selection is represented in Fig. 1.

### Risk of bias assessment

The Quality Index Checklist by Downs and Black [24] was used to assess the risk of bias of the studies. The full version of the Downs and Black Checklist [24] was used to assess randomized and non-randomized clinical trials, while the modified version was used to assess all the other studies. The number of items of the modified version is reduced from 27 to 14 (1, 2, 3, 5, 6, 7, 10, 11, 12, 16, 18, 20, 21, 22) to eliminate questions that are applicable only for intervention studies. Each study was divided by quality according to the percentage of met items: low quality (< 60%), moderate quality (61–74%), or high quality (≥75%) [25]. Detailed information on the risk of bias assessment is reported in Additional file 2: Appendix 2. The reviewers assigned the judgment and the Fleiss’ Kappa measure was used for assessing the reliability of agreement between the two reviewers by the use of an independent observer. This measure calculates the degree of agreement in classification compared to what would be expected by chance, and it is scored between 0 and 1. Reliability from Kappa values was interpreted as *<* 0 = Poor agreement; 0.0–0.20 = Slight agreement; 0.21–0.40 = Fair agreement; 0.41–0.60 = Moderate agreement; 0.61–0.80 = Substantial agreement and 0.81–1.00 = Almost perfect agreement, according to Landis and Koch [26].

### Outcomes of interest and data synthesis

Data were extracted by three independent reviewers (FC, FB, NS), and in case of disagreement a fourth reviewer (LL) was consulted. Data extracted from studies focused on balance and proprioception assessment were: (1) authors of the study; (2) characteristics of the patients involved in the study; (3) surgical approach for THA implant; (4) the training intervention performed; (5) the protocol of assessment; (6) timepoints at which the assessments were performed; and (7) clinical results. Data extracted from the studies focused on the training of balance and proprioception were: (1) authors of the study; (2) characteristics of the patients involved in the study; (3) surgical approach for THA; (4) volume, duration and time of the training; (5) the assessments performed and timepoints in which these were performed; (6) the protocol of assessment; and (7) clinical results. If relevant data were not reported in any of the studies, the corresponding author was contacted. The data extracted from the studies and included in the qualitative synthesis are reported in Table 1 and Table 2.

**Table 1:** Balance and proprioception assessment

*Authors*	*Participants*	*Type of surgery*	*Assessments*	*Protocol*	*Time*	*Results*
*Brauner* et al.	THA GROUP: 61 patients (F 27, M 34; mean age: 62 y) HEALTHY CONTROL 38 healthy participants (16 F, 22 M; mean age: 47 y)	?	**Dynamic stability** was assessed using 2 pedal posture- pressure mats with a sensing surface (44X20cm) incorporating 220 capacitance-based, individually calibrated pressure sensors.	Participants were asked to stand with each foot on 1 of 2 pressure mats, a therapeutic chair was placed behind them, with sitting surface 30° tilted and height individually fixed so that to ensure that participants approached but did not exceed 90° hip flexion. They were instructed to keep their arms outstretched and positioned horizontally in front of them with two fingers of each hand placed on a linear bearing. From the upright position subjects performed 2 sets of 5 squats, lowest position was reached when they perceived the chair. Participants performed exercises at their preferred movement velocity with 1 min rest between each exercise set.	13.2 days postoperatively (T1), then after 2 weeks rehabilitation period, 26.6 days postoperatively (T2).	At T1, unloading of the operated limb was 15.8% greater (*P* < .001) and anteroposterior and mediolateral centre of pressure root mean square values were 30 to 34% higher in patients who had undergone THA than in the healthy reference group (*P* < .05). Unloading was reduced by 12.8% toward a more equal distribution from T1 to T2 (P < .001). Although mediolateral stability improved between T1 and T2 (operated limb: 14.8%; P 0.024 non operated limb: 13.1%; P 0.015), anteroposterior stability was not significantly different.
*Butler* et al.	THA GROUP: 75 patients (F?, M?, mean age: 66.1 y) TKA GROUP: 65 patients (F?, M?, mean age: 61.8 y) TAA GROUP: 94 patients (F?, M?, mean age: 64.8 y)	?	**Single leg balance** was assessed using single-leg stance (SLS) time.	Participants were asked to maintain unilateral balance without assistance for 10 s on their surgical and non-surgical limbs while barefoot and with eyes open on a firm surface. Subjects allowed to move their upper extremities, but not to use they non-supportive limb for balance.	Twelve months after surgery.	THA group (63%) and TKA group (69%) had similar pass rates compared to TAA group (9%). For the THA patients the SLS time on their surgical limb was 3.3 s while it was 5.3 s on their non-surgical limb. For the TKA patients the SLS time on their surgical limb was 4.4 s, while it was 5.7 s on their non-surgical limb. For TAA patients SLS time on their surgical limb was 3.2 s, while it was 6.3 s on their non-surgical limb.
*Calò* et al.	THA GROUP: 23 patients (F 13, M 10; mean age: 64.2 y) HEALTHY CONTROL 20 healthy participants (10 F, 10 M; age matched with THR group)	THA with lateral approach	**Postural control** was assessed by means of dynamic posturography (Equi-Test Dynamic Posturography System by NeuroCom. Int. Inc., Clackamas, OR, USA)	Participants were asked to stand on a dual force-plate enclosed by a visual surround while performing a number of standing tasks based on quiet standing with eyes open and closed, with fix or moving back ground, responses to sudden translations or inclinations of the force platforms.	4 months after THR	No statistically significant differences were observed between patients and controls.
*Chang* et al.	23 patients (F 13, M 10; mean age 60 y)	THA with anterior, anterolateral or posterior approach.	**Postural stability** was assessed using a pressure measurement mat (RSscan International Co.,Belgium). **Balance** was assessed using the Berg Balance Scale (BBS) **Balance confidence** was assessed with the Activities-specific Balance Confidence Scale (ABC) Functional reach test.	Participants were asked to stand barefoot with 4 ft placements: shoulder width stance (SWS), feet side-by-side stance (SSS), tandem stance with affected limb in the front (AFS), and tandem stance with non-affected limb in the front (SFS). They were instructed to stand as still as possible with both arms at their sides and eyes staring at a target 5 m away in front of them. In each foot placement condition, three trials lasting 30 s were recorded with 15 s resting interval between trials. The BBS is comprised of 14 tasks and is scored on a 5-point scale (0–4). The ABC is a 16-item self-report measure of a person’s confidence in performing various activities of daily living without falling or experiencing a sense of unsteadiness.	One day before surgery, at 2 weeks, 6 weeks, 3 months, 6 months, and one year after surgery.	When the foot placement changes from shoulder width stance (SWS), side-by-side stance (SSS), and non-affected limb in the front stance (SFS) to affected limb in the front stance (AFS), the subjects tended to increase their postural sway after surgery and the progressive increment of CoP sway path length (CoPR). The CoPR had the greatest postural sway at the time of 2 weeks after surgery, but the CoP sway in ML direction (CoPML) at the time of 6 months after surgery. The descriptive data showed that the CoPR tended to decrease progressively after surgery. CoP sway in AP direction (CoPAP) peaked between6 months and 1 year after surgery, especially at the AFS and SFS position. Berg balance test decreased significantly (*p* < 0.05) at 2 weeks and improved gradually, reaching the highest score of53.2 ± 1.9 at 6 months. The difference in functional reach test in all three directions was approaching significant level (*p* = 0.07–0.08). The descriptive data showed that functional reach distance tended to improve gradually after surgery, reached the maximum distance, and decreased at 1 year postoperatively.
*Esbjornsson* et al.	HIP OSTEOARTHRITIS GROUP: 28 patients (F 18, M 10; mean age 66.0 y) HEALTHY CONTROL 21 healthy participants (F 13, M 8; mean age 66.2 y)	THA with an anterolateral approach.	**The centre of mass trajectories** was assessed using a 8 camera system (Vicon Motion Systems Ltd., Oxford, UK) and a conventional biomechanical model, the Plug-In-Gait full-body.	Participants were asked to rise from a seated position to a standing position as fast as possible five times consecutively, with the arms placed across their chest. The test was performed twice.	One month prior to THA and 1 year postoperatively.	Preoperatively patients in the hip osteoarthritis group displayed a larger contralateral shift (*p* < 0.001) and forward displacement of the centre of mass (*p* = 0.022) compared to control group. After surgery, deviations in both dimensions were reduced (medial-lateral *p* = 0.013; anterior-posterior *p* = 0.009), but the contralateral shift of the centre of mass remained larger (*p* = 0.010), indicative of persistent asymmetric limb loading.
*Esposito* et al.	THA ES group: 15 patients (mean age 68.7 y) THA EF group: 15 patients (mean age 69.6 y)	THA procedure through a tissue sparing technique using a hybrid implant (uncemented cup and cemented stem	Two force platforms and an optoelectronic motion tracking system (SMART DX-700, BTS Bioengineering, Milan, Italy), were used. CoP total path and maximal excursion were evaluated in both medio-lateral (ML) and anterior – posterior (AP) planes. Shoulder and lower limbs loading were assessed.	Participants were asked to stand on a force platform in a comfortable position with the crutch positioned on the unaffected side, facing forward for 10 s, with elbow straight or elbow flexed.	At a mean of 4 ± 1 days after surgery	No significant differences between the two groups were found for postural stability parameters. Shoulder load increased significantly in EF group compared to ES group. Leg loading symmetry was significantly reduced in the EF group.
*Eyvazov* et al.	28 patients with hip-spine syndrome (F 17, M 11; mean age 61.7 y)	THA with an anterolateral approach (cementless prosthesis)	**Spinal sagittal alignment** was assessed using anteroposterior and lateral standing X-rays. **Static balance** was assessed using a 950–460 Bio Sway system (BIODEX Medical Systems, Inc. Shirley, NY, USA).	Patients were asked to stand in a comfortable position, with knee fully extended and arms slightly forward flexed while gazing forward. Patients were asked to stand in a comfortable position with knee fully extended and arms forward flexed and the following parameters were measured: PST – postural stability test (patient’s ability to maintain centre of balance), LOS – limits of stability test (the maximum angle patient can achieve from vertical without losing balance), CTSIB – clinical test of sensory integration of balance (objective measure for patient postural control on a static surface).	One month before surgery and 6 months after surgery.	No significant changes in sagittal alignment were observed postoperatively. Median CTSIB improved from 1.22 (1.07, 1.45) to 1.01 (0.80, 1.19) and LOS from46.0 (42.0, 58.0) to 37.0 (32.0, 39.0) postoperatively.
*Holnapy* et al.	DIRECT-LATERAL (DL) EXPOSURE GROUP: (F 13, M 12; mean age 59.9 y 60.1 y respectively) ANTERO-LATERAL (AL) EXPOSURE GROUP: (F 11, M 11; mean age 62.1 y 61.3 y respectively) POSTERIOR (P) EXPOSURE GROUP: (F 12, M 13; mean age 60.8 y 61.2 y respectively) CONTROL GROUP: (F 22, M 23; mean age 60.4 y 60.9 y respectively)	THA with traditional direct-lateral (DL) exposure with the joint capsule removed THA with antero-lateral (AL) exposure with the joint capsule removed THA with posterior (P) exposure with the joint capsule preserved	**Dynamic balance** was assessed using Posturo Med (Haider-Bioswing GmbH, Weiden, Germany)	Subjects were asked to stand on the platform, a sudden unidirectional perturbation (medial and lateral direction in the horizontal plane) was given and the subjects had to balance on the moving platform to regain their equilibrium in the given position. In this case, the rigid plate performed damped free oscillation; the damping corresponded to the subject’s balancing ability. The testing was performed in the sequence of standing on both limbs, standing on the non-affected limb (dominant limb for controls) and standing on the affected limb (non-dominant limb for controls). Each subject underwent 9 tests. Lehr’s damping ratio was calculated as the balancing capacity in response to a sudden unidirectional perturbation.	Prior to and at 6 weeks, 12 weeks and 6 months after THA.	In the case of direct-lateral and antero-lateral exposure, Lehr’s damping ratio significantly decreased compared to the preoperative values at 6 weeks postoperatively, but it increased steadily after-wards. Lehr’s damping ratio while standing on the affected limb was significantly lower – even at 6 months postoperatively – than that of the control group. In the case of posterior exposure (with the joint capsule preserved), Lehr’s damping ratio continuously increased in the postoperative period and corresponded to that of the control group at 6 months after total hip arthroplasty.
*Hunter* et al. *2020a*	DIRECT ANTERIOR (DA) SURGICAL APPROACH GROUP: 61 patients (F?, M?; mean age 68.4 y) DIRECT LATERAL (DL) SURGICAL APPROACH GROUP: 74 patients (F?, M?; mean age 69.4 y)	THA with direct anterior surgical approach THA with direct lateral surgical approach	Activities-specific Balance Confidence Scale (ABC) **Future falls risk** was assessed with the Falls Risk for Older People in a Community Setting (FROP-Com) **Dynamic balance** was assessed with the Step Test.	The ABC is a 16-item self-report measure of a person’s confidence in performing various activities of daily living without falling or experiencing a sense of unsteadiness. The FROP-Com is a multifactorial falls risk assessment consisting of 28 questions assessing 13 known falls risk factors. Step Test: participants were instructed to stand with feet parallel, approximately 10 cm apart, with a step measuring 15 cm in height placed 5 cm in front of them. Participants placed their entire foot on the step and then back to the floor as rapidly as possible over 15 s. Each leg was tested separately and the total number of times the foot was placed on the step for each leg was recorded.	One year after THA	No statistically significant (*p* = .052) or clinically important difference for the ABC scale scores, though there was a small effect size of 0.34, 95% CI (−0.00, 0.68) in favour of DA approach. No statistically significant (*p* = .055) or clinically important differences for the FROP-com while again there was a small effect size of 0.32, 95% CI (− 0.66, 0.02) in favour of the DA approach. No statistically significant or clinically important difference for Step Test for least number of steps (*p* = .09), Step Test for non-surgical side (*p* = .15), Step Test for the THA side (*p* = .11). There were small effect sizes for these non-significant outcome measures in favour of the DA approach.
*Hunter* et al. *2020b*	PATIENTS SUSTAINING at least 1 FALL AFTER THA (F 19, M 6; mean age: 71.6 y) PATIENTS SUSTAINING NO FALL EPISODES (F 46, M 37; mean age: 72.7 y)	Direct anterior (47 patients); direct lateral (61 patients)	Activities-specific Balance Confidence Scale.	Each item of the Activities-specific Balance Confidence Scale is rated on a scale of 0 to 100%, with a score of 0 representing no confidence, whereas a score of 100 represents complete confidence. A summary score is calculated by adding responses on each item and dividing by the total number of items.	Twelve months after THR	ABC scale result was not a predictor of falls in the first year after surgery.
*Jo* et al.	FRACTURE GROUP: 16 patients (F 10, M 6; mean age: 66.6 y) NON-FRACTURE GROUP: 15 patients (F 10, M 5; mean age: 64.6 y)	THR with posterior approach	**Dyanamic balance** was assessed using the Berg Balance Scale (BBS). **Proprioception** was assessed using the joint position sense test (JPS).	The BBS is comprised of 14 tasks and is scored on a 5-point scale (0–4). The test takes about 20 min. The JPS test measured the hip flexion accuracy of position replication in a supine position.	Three months after the surgery.	BBS scores of the non-fracture group were 44.06 ± 11.94 which was statistically higher than fracture group scores of 29.00 ± 17.73. However, there was no statistical significance in the JPS between the two groups. In the correlation between balance and other variables 3 months after the surgery, the BBS score and JPS were correlated.
*Larkin* et al.	TOTAL HIP RESURFACING: 16 patients (F 10, M 6; mean age: 66.6 y) THA FEMORAL HEAD > 32 MM: 25 patients (F 12, M 13; mean age: 53.3 y) THA FEMORAL HEAD ≤32 MM: 25 patients (F 13, M 12; mean age: 53.6 y) CONTROL GROUP: 25 patients (F 3, M 22; mean age: 52.9 y)	?	**Dynamic postural stability** was assessed using a stabilometric force platform (PROPRIO 5000 machine, Perry Dynamics, Decatur, IL, USA)	Patients were placed on the platform with their feet shoulder width apart, knees slightly flexed, and center of mass centered over the center of the board by a single examiner. A 6-in. piece of rope was placed in the patients’ hands and they were instructed to hold this in front of them to remove the effect of the upper extremities on balance. An ultrasonic sensor was placed at the L5-S1 junction and this transmitted the patients’ position in space. After the double-limb testing was complete, three 1-min tests in each single-limb stance were performed with the side being tested first being randomly assigned. Each trial finished when one of the following criteria was met: 1 min had elapsed, the patient exceeded 3 in. of movement in 0.25 s, the patient moved greater than 5 in. from the starting point, the patient let go of the rope, the patient moved their feet, or the patient asked to stop.	Between 1 and 5 years after THA	Double-limb scores were not different among the four groups compared. With single-limb testing of the operative limb only considered, the total hip resurfacing arthroplasty group performed better than patients undergoing standard THA, and there was a trend for total hip resurfacing arthroplasty to perform better than the entire THA population but not better than large-head THA. However, when the operative limb was normalized against the nonoperative limb, the differences among groups were no longer significant.
*Lavigne* et al.	HIP RESURFACING GROUP: 24 patients (F 10, M 14; mean age: 49.6 y) LARGE-DIAMETER HEAD THA GROUP: 24 patients (F 9, M 15; mean age: 49.8 y) CONTROL GROUP: 14 patients (F 6, M 8; mean age: 44.4 y)	THA and HR with a posterior surgical approach and cementation of the femoral component.	**Postural control** was assessed using two embedded AMTI force platforms (Advanced Mechanical Technology Inc., Watertown, MA) TUG test Functional reach test. Step test.	Subjects were asked to stand as still as possible with feet at shoulder width for 120 s on the force platforms recorded at 120 Hz the ground reaction forces and moments. The patients were asked to fix a target located 3 m in front of them at eye level. For the timed up and go test, the patient had to rise from a standard arm chair, walk as fast as possible until he reached a stool placed 10 ft ahead of him, contour it, and come back to the chair and sit back. During the functional reach test, the patient was standing still, shoulder flexed at 90°and the other arm on the side. We asked the patient to reach as far as possible ahead of him with his index finger without lifting the heels from the ground and the maximum distance reached (in centimetres) was measured with a ruler. The step test consisted of five consecutive rises and descents from an 18-in. step (using the operated leg to climb up and keeping the same leg on the step when going down) that were performed as fast as possible. We recorded the time to accomplish this task.	Before surgery, 3, 6, and minimum 12 months	No difference was found preoperatively and at all follow-ups among HR, large-diameter head THA, and control subjects during the quiet standing test as shown by similar total path length of the COP in all groups. There were no differences between the prosthesis groups for the timed up and go test. We found differences between the two prostheses groups for only two tests: the functional reach and the step test at the advantage of patients undergoing HR and those undergoing large-diameter head THA, respectively. The timed up and go test is the only functional test in which the control group surpassed the prostheses groups with a faster time at all evaluations compared with both study groups.
*Lugade* et al.	THA GROUP: 20 patients (F 6, M 14; mean age: 57 y) HEALTHY CONTROL 10 healthy participants (F 5, M 5; mean age: 59.9 y)	All patients underwent primary THA using the anterior (12 patients) or lateral (eight patients) surgical approach on the affected limb and received uncemented (17 patients) or cemented (three patients) Zimmer hip implants	**Dynamic balance** control during gait was assessed using 3D Gait Analysis (Motion Analysis Corp, Santa Rosa, CA) and two force plates (Advanced Mechanical Technologies Inc., Watertown, MA).	Dynamic balance was assessed during gait using the CoM (centre of mass)-CoP (centre of pressure) inclination angles in the frontal and sagittal planes. Control subjects were tested for identical variables during two visits. During each visit, were asked to subjects to walk at a self-selected pace along a 10-m walkway. Were collected four level walking trials during each visit as subjects ambulated along the walkway. Control subjects were tested during two visits 1 month apart to ensure test repeatability.	Pre-surgery, 6 weeks, and 16 weeks post-surgery	Patients having THA had improved gait and balance control by 16 weeks post-surgery. Patients undergoing THA had a smaller medial inclination angle with a greater posterior inclination angle 16 weeks post-surgery when compared with before surgery. The CoM velocity also increased for patients undergoing THA at maximum anterior and posterior CoM-CoP separation by 16 weeks post-surgery. However, at 6 weeks post-surgery, only the medial inclination angle improved from before surgery. When compared with the control subjects at 16 weeks post-surgery, patients undergoing THA performed similarly in gait but their CoM-CoP inclination angles in anterior and medial directions failed to reach control levels.
*Majewski* et al.	THA GROUP: 25 patients (F 11, M 14; mean age: 67 y) HEALTHY CONTROL 50 healthy participants (age and gender matched)	THA with trans-gluteal approach	**Balance** in the form of trunk pitch (forwards-backwards) and roll (side-to-side) movements was assessed with the SwayStar balance system (Balance International Innovations GmbH)	Participants were asked to perform quiet stance, gait, gait over barriers, stairs and sit-to-stand tasks.	Before surgery and at 4 and 12 months after surgery	There was a progressive improvement at 4 and 12 months for gait and sit-to-stand tasks parameters. By 12 months, the values approached those of the control group. Trunk pitch (forwards-backwards) and roll (side-to-side) velocities were less stable when walking over barriers as was roll for the sit-to-stand task, indicative of a residual deficit of balance in THA patients.
*Merle* et al.	14 patients (F 6, M 8; mean age between 57 and 85 y)	THA with postero-exterior (11) and anterior (3) approach	**Quiet standing** over a dual force platform (PF02 Equi+, Aix-les-Bains, France). The center of pressure displacement was recorded separately under each limb.	Participants were instructed to remain as stable as possible in a standardized upright position in two conditions: spontaneous, i.e. no instructions as to how to distribute the body weight, and imposed, i.e. instructions to load the body weight on the operated limb.	12 ± 3 days after surgery	In the SPO condition, the operated limb was less loaded than the healthy limb. In the IMP condition load distribution was close to symmetry. Greater displacements along the ML axis was found for the trajectories measured under the healthy limb than under the operated limb.
*Nantel* et al.	SURFACE REPLACEMENT ARTHROPLASTY (SRA) GROUP: 10 patients (F 4, M 6; mean age: 43.1 y) TOTAL HIP ARTHROPLASTY (THA) GROUP: 10 patients (F 5, M 5; mean age: 51.1 y) CONTROL GROUP: 10 patients (F 4, M 6; mean age: 45.1 y)	THA and SRA with a posterior surgical approach and uncemented prosthesis.	**Postural balance** was assessed using a force platform AMTI force plate (Advance Mechanical Technology Inc., MA, USA).	All participants were asked to perform two postural tasks. For the first task, patients were requested to maintain a quiet standing posture on the force platform, with eyes open and feet at shoulder width for 120 s. For the second task, patients had to maintain a one leg stance position for 10 s. The operated leg was tested twice with an inter-trial resting period of 30 s.	Six months after surgery	During static dual stance, the statistical analyses revealed significantly larger Root-Mean-Square and RMS_COM_ - Root-mean-square (RMS) of the center of pressure (COP)- amplitude in the medial**–**lateral direction for THA subjects compared to SRA and control subjects. Statistical analysis showed significant dependence between groups and one leg stance completion (*P* = 0.01). Five of the ten patients in the THA group did not complete the task compared to one for the SRA subject. In the control group all subjects completed the task
*Nantel* et al.	SURFACE REPLACEMENT ARTHROPLASTY (SRA) GROUP: 14 patients (F 5, M 9; mean age: 45.0 y) LARGE-DIAMETER HEAD TOTAL HIP ARTHROPLASTY (LDH THA) GROUP: 14 patients (F 3, M 11; mean age 50.8 y) CONTROL GROUP: 14 patients (F 6, M 8; mean age 44.5 y)	LDH-THA and SRA with a posterior surgical approach and uncemented prosthesis.	**Postural balance** was assessed using a force platform AMTI force plate (Advance Mechanical Technology Inc., MA, USA)	Each participant had to achieve 2 postural tasks. The first task consisted of quiet standing for 120 s with eyes open. In the second task, patients had to hold a one-leg stance position on the operated limb for 10 s. The operated leg was tested twice with a sufficient intertrial resting period. This task was considered successful when the patient was able to stay still on 1 leg for 10 s and was considered unsuccessful if the patient had to touch the ground with the contralateral foot.	Between 5 and 7 months	The statistical analyses for the dual stance task revealed significantly lower larger Root-Mean-Square_COP_ amplitudes in the medial-lateral direction for large diameter head THA and SRA subjects compared to control subjects. No significant differences between groups in the anterior-posterior direction for both RMS_COP_ R Root-mean-square (RMS) of the center of pressure (COP)- RMS_COM_ -Root-mean-square (RMS) of the center of mass (COM)- and RMS _COM_ amplitudes. There was no significant difference in the ability to complete the one-leg stance task between the 3 groups.
*Ninomiya* et al.	THA GROUP: 58 patients (F 48, M 10; mean age: 68) CONTROL GROUP: 46 patients (F 38, M 10; mean age: 69.9)	Antero-lateral operative procedure	**Single-limb stance time**. Fall Rate.	Single-limb stance time was measured with subjects in a standing posture with both hands on the hips, starting at the time when one foot was lifted from the floor from the standing position. The evaluation was stopped when 1) the support leg shifted from the floor, 2) the lifted leg touched the floor, 3) the lifted leg came in contact with the support leg, or 4) 60 s was reached. The fall rate was surveyed by asking participants if they had fallen in the past year. Falls were defined as “a person falling onto the same level or a lower level on their own, with no external force from another person, loss of consciousness, paralysis from a sudden attack such as stroke, or an epileptic seizure.	Ten years after THA	THA patients had 42.1% shorter single-leg stance time on the operated side and 2.8 times higher fall rate.
*Pop* et al.	THA GROUP: 55 patients (F 31, M 24; mean age: 56 y) HEALTHY CONTROL 48 healthy participants (F 31, M 17; mean age: 58 y)	THA with a lateral approach and uncemented prosthesis.	**Static balance** was assessed using a stabilometric force platform (Alfa, AC International East, Poland).	Subjects were asked to stand barefoot on the force platform in a comfortable, self-chosen, **double-leg stance position**, with their arms alongside the body. They were instructed to stand as still as possible during the tests and to breathe normally. Data were collected in both visual conditions (eyes opened and eyes closed) in random order. During the test with opened eyes, subjects were asked to look straight ahead at a visual reference point. Each test lasted 30 s, followed by 30 s of rest interval.	Between 24 and 36 months after THR	In the eyes-open condition, COP mean velocity in ML direction was significantly higher in THR compared to controls. In the eyes-closed condition, COP mean velocity in AP and ML direction and COP path length were significantly higher in THR compared to controls. In both groups COP mean velocity in AP and ML direction, and COP path length and area were higher in the closed-eyes compared to the open-eyes condition. THR males had higher COP mean velocity in AP and ML direction, and COP path length than male controls, while no between-groups differences were found for females.
*Quagliarella* et al.	EXPERIMENTAL GROUP: 181 patients divided into THA and TKA group (F 118, M 63). THA 81 patients pre-operatively (mean age: 64.1 y), THA 20 patients at 6 months (mean age: 62.9 y), THA 14 patients at 12 months (mean age: 66.6 y) CONTROL GROUP: 59 patients (F 24, M 35; mean age: 67.4 y)	?	**Static balance** was assessed using orthostatic posturography (Kistler Instrumente AG Winterthur, Switzerland).	Participants were asked to stand barefoot on the force platform facing in the anterior-posterior (AP) direction, in a comfortable self-chosen stance, with their arms hanging down beside the body. Each subject carried out one test with eyes open and one with eyes closed, to evaluate the Romberg ratio. During the eyes open trial, subjects were asked to look straight ahead at a visual reference point (a red dot 3 cm in diameter, located 2 m away on the wall, at eyes height). Between trials, subjects could rest in a chair for approximately 2 min.	Prior to surgery, after 6 months and after 12 months.	No statistically significant differences (SSD) due to age were found in the Posturographic Parameters (PPs) after subdividing the CG and the EGs (for each trial session) into two subgroups (under/over 60 years. The assessment with closed eyes evidenced more SSD among the two EGs and the CG than the test with open eyes. Preoperatively SA (sway area), MV (mean velocity of the CoP), RMSD (as resultant and in the CoP AP and ML directions) in particular in the ML direction, and PF95_AP_ (that estimates the frequency extent of the CoP time series) were higher in THA than in the CG. During the follow-up only in the THA group was there a steady reduction toward normal values in RMSD_RD_ (the vector of distances from the mean CoP to each of its points), RMSD_AP_ and SA, whereas the variations between sessions of the other PPs did not present a clear trend.
*Queen* et al.	THA female group (n: 57; mean age 57.7 y) THA male group (n: 70; mean age 52.7 y)	?	**Static balance** was assessed with a 10 s single-limb stance task **Dynamic balance** was assessed with the Lower Quarter Y-Balance Test	Patients were asked to stand on a single limb for 10 s. Values were treated as binary with success being greater than 10 s and failure being less than 10 s. For the YBT-LQ, the participant was instructed to remain in unilateral stance on the stance platform while pushing the reach indicator in three independent directions (Anterior, Postero-medial, and Postero-lateral)	At least 1 year following surgery	Women failed single-leg stance at a higher rate than men. Reach distance was different between limbs for all reach directions with greater reach distance on the nonoperative limb for all patients. Men had a greater reach distance in the ANT and PM directions.
*Rasch* et al.	THA group: 22 patients (F 18, M 4; mean age 67 y)	THA with 2 types of hip prostheses: a cementless porous-coated femur stem (*n* = 8) and a cemented polished and tapered femur stem (*n* = 12).	Assessment of **postural stability** with a force plate (MuscleLab; Ergotest, Langesund, Norway).	The patients were told to stand still on the force plate with a gap of 20 cm between the feet. 6 measurements of bilateral standing with alternating open or closed eyes were first conducted, followed by 6 measurements standing on 1 ft—alternating OA and healthy limb—with eyes open.	The day before surgery, and 6 months and 2 years after THA.	Sway measurement of unilateral standing before and after operation showed no statistically significant differences between OA limbs and healthy limbs except for the 6-month follow-up of sagittal sway, where it was greater in the OA limb (*p* = 0.02). Measurement of bilateral standing showed a reduced lateral and sagittal sway postoperatively compared to preoperatively, although this was only statistically significant with eyes closed (*p* = 0.006). The sagittal sway was more pronounced than the lateral sway in both OA and healthy limbs, both preoperatively and postoperatively.
*Rougier* et al.	THA GROUP: 14 patients (F 6, M 8; mean age between 57 and 85 y) CONTROL GROUP: 13 patients (F 10, M 3; mean age between 69 and 95 y)	THA with posterolateral (10) and anterior (4) approach	**Standing balance** on a double force platform (PF02, Equi+, Aix les Bains, France).	The subjects stood barefoot on a double force platform with eyes closed. Control group participants were required to adopt an asymmetrical body weight distribution close to that observed on average for the patients. The center-of-pressure (CP) and the center-of-gravity (CG) movements were analysed for each limb.	12 days after surgery	Patient with THA showed greater movements for both plantar and resultant CP displacements, principally along the antero-posterior (AP) axis, a decreased contribution of the hip mechanisms in the production of CP displacements along the medio-lateral (ML) axis, greater resultant CP and CG movements along the AP axis and increased differences between CP and CG along both ML and AP axes.
*Sliwinski* et al.	THR GROUP: 16 patients (M 7, F 9; mean age 70.9 y) HEALTY CONTROL: 16 healthy participants (M 5, F 11; mean age 74 y)	THA (16 participants had cemented procedures and 2 had non-cemented)	**Dynamic stability** (the vertical projection of the centre of mass -COM- to the base of support -BOS) n the medial–lateral direction during walking was evaluated with a VICON 370 system and workstation software.	27 spherical retro reflective markers (2.5 cm in diameter) were affixed to the participant’s skin using adhesive tape to the following land marks (1), forehead (2), chin (3), right and left acromion processes (4), right and left lateral epicondyles of the humerus (5), right and left wrist midpoints (6), right and left third metacarpal phalangeal joints (7), sacrum (8), right and left ASISs (9), right and left greater trochanters (10), right and left femoral wand markers (11), right and left lateral knee joints (12), right and left calf wand markers (13), right and left lateral malleoli (14), right and left calcanei (15), and right and left second metatarsal phalangeal joints. Heel switches were affixed to the bottom of the heels of each shoe to assist in data processing of heel strike and toe off identification for temporal–spatial analysis. Participants were instructed to wear their customary walking shoes and to walk as they would normally at their typical walking speed	All measurements were obtained in a single session. The patients were a minimum of 2 months post-surgery (Ten of the THA participants were 2–3 months post-surgery, and one each 5, 6 and 7 months, and one 2 years post-surgery).	No significant difference was identified during single-limb-support phase for within and between group comparisons. During each leading limb condition, for the healthy older adults, the vertical projection of the COM during double-limb-support was held on average laterally (2% lean to the right, and 4% lean to the left) towards the leading limb side. The absolute difference score for DLS dynamic stability was larger for the healthy older adults (5.7 ± 2.5%) in comparison to the individuals with THA (1.7 ± 0.74%). The individuals with THA held their COM on average toward their operated limb (operated 54.2 ± 4.7%, non-operated 52.3 ± 5.0%) during both limb lead conditions as the COM location was greater than 50%. Walking velocity was slower with individuals with THA (1.09 ± 0.19 m/s) compared to healthy older adults (1.25 ± 0.15 m/s).
*Szymansky* et al.	THR GROUP: 20 patients (F 10, M 10; mean age 61,2 y) RESURFACING GROUP: 20 patients (F 8 M 12; mean age 54,1 y) CONTROL GROUP: 20 subjects (F 10, M 10; mean age 31,2 y)	Hip resurfacing implantation with posterolateral approach and total hip replacement cementless stem	**Balance** was evaluated with a force platform (QFP Système, Medicapteurs, Nice, France)	During measurement, the subject was standing at ease, arm along the body and feet in 30° external rotation in line with AFP standard 85. The subject was to focus on a point 3.5 m in front of him or her, and remain standing for 25.6 s, followed by 25.6 s focusing on the point in monopedal weight-bearing on the operated side and then on the contralateral (healthy) side for 25.6 s; the contralateral healthy side served as reference for all measurements. In the healthy control group, the same three tests were performed, with bipedal and left and right monopedal weight-bearing. An observer stood at either side of the subject to guard against any fall.	The two cohorts which underwent surgery were evaluated in one occasion post op at 15.5 ± 2.3 months -range, 12–20 months- the THR group had a mean follow-up of 15.3 ± 2.6 months).	Balance analysis on both legs found comparable results in the control and resurfacing groups. The weight-bearing both leg balance area was greater in the hip replacement than in either of the other two groups (*p* < 0.05), and five times greater than in the resurfacing group (p < 0.05). The single leg weight-bearing balance results were significantly better in the resurfacing group, with a balance area half that of the hip replacement group, whether on the operated or the non-operated side (*p* < 0.001). In all groups, the difference between left and right monopedal stance was non-significant.
*Talis* et al.	THE GROUP: 27 patients (F 20 M 7; mean age 56 y) HEALTHY CONTROL: 27 healthy participants (F 18, M 9; mean age 55 y)	?	**Balance** was assessed with the use of two force platforms measured vertical forces under each foot during quiet standing and sit-to-stand manoeuvre.	During quiet standing (barefoot) the feet of the subject were placed on separate stabiloplatforms, with the heels spaced 10 cm apart. Thus, the bilateral loadings were measured directly and independent of the varus/valgus moments. Subjects were instructed to stand naturally for 40 s with their arms at their sides: 20 s with eyes open (EO) and 20 s with eyes closed (EC). Subjects were seated on an armless chair (without a back support) whose height was adjusted to keep the thigh horizontal. The feet were placed on separate force plates, with the heels spaced 10 cm apart being the same as in the quiet standing task. Subjects were instructed to rise from a chair as they would usually do except without using the arms. An acoustic tone served as a trigger (‘go’) signal to stand up. Subjects (barefoot) executed this movement at natural speed with eyes open (EO), as fast as possible (fast) and at natural speed with eyes closed (EC) (three trials in each condition).	Mean time after surgery 19 months (between 2 months and 2.8 years)	In all tasks patients tended to preferentially load the non-operated limb, though the amount of asymmetry depended on the task being most prominent during standing up (inter-limb weight bearing difference exceeded 20%, independent of speed or visual conditions). In contrast, when performing quiet standing, the inter-limb difference was typically less than 10%. Visual information seems to play only a minor role in the control of the weight-bearing ability.
*Temporiti* et al.	GROUP BILATERAL THA: 20 patients (F 2, M18; mean age 51.6 y) GROUP UNILATERAL THA: 20 patient (F 6, M 14; mean age 53.1 y)	One stage bilateral or unilateral THA.	Postural tasks were recorded with an eight-camera optoelectronic system (SMART DX, BTS, Italy) synchronized with two force platforms (P-6000, BTS, Italy). Timed Up and Go (TUG), Body Weight Distribution Symmetry Index (BWDSI) during stand-to-sit (STS).	TUG: Patients were asked to rise from an armchair, walk at a comfortable pace for three meters, turn and walk back to the chair and sit down again. Postural tasks: Three retro-reflective markers were placed on the two acromial angles and on the seventh cervical vertebra to detect their motion with the optoelectronic system. The following tasks were recorded: 1) Standing - patients were asked to stand barefoot in standing position with their arms crossed over their chest (feet were positioned parallel on two adjacent force platforms with heels at 10 cm apart and equidistant from the medial edge). Study participants were asked to maintain the standing position for 60 s with eyes open (EO) while looking at a fixed point placed in front of them at two meters distance. After resting for 5 min, the same test was executed with eyes closed (EC). 2) Stand-to-Sit - patients were seated on an adjustable-height chair with back support, with knees flexed at 100° (feet placed parallel on two adjacent force platforms with heels at 20 cm and equidistant from the medial edges). Patients were asked to stand up and, after 10 s, they were asked to sit down with their arm across their chest returning to the same initial position. Only the stand-to-sit component of the task (STS) was processed due to the inability of several participants to rise from the chair without using their arms.	Before surgery, at three and seven days after.	No between-group differences were found for TUG. BWDSI during STS and standing revealed differences over time in favour of patients with bilateral THA, who showed better symmetry in weight distribution. Shorter CoP path length was observed during standing in patients with unilateral THA, who mainly used their non-affected limb to maintain balance.
*Ulivi* et al.	DIRECT SUPERIOR APPROACH: 22 patients (F 15, M 7; mean age 74 y) POSTEROLATERAL APPROACH: 23 patients (F 13, M 10; mean age 72 y)		The **risk of falls** was assessed by means of the Brief-BESTest Timed Up and Go (TUG)	Brief-BESTest: 36 items grouped into 6 specific postural control systems: biomechanical constraints, stability limits and verticality, anticipatory postural adjustments, postural responses to external perturbations, sensory orientation during stance, and stability in gait. TUG: Patients were asked to rise from an armchair, walk at a comfortable pace for three meters, turn and walk back to the chair and sit down again.	Before (PRE), 1 month (T1) and 3 months after (T3) surgery	Patients with DSA had a lower risk of falls at T3 compared with T1 and higher TUG scores at T3 compared with T1 and PRE. Furthermore, PL showed a lower risk of falls at T3 compared with T1 and PRE while TUG did not show any statistically significant difference
*Van Driessche* et al.	THA GROUP: 44 patients divided into: - posterior approach (F 7, M 7; mean age: 70.9 y) - anterior approach (F 8, M 7; mean age: 69.3 y) - Rottinger approach (F 7, M 8; mean age: 69.3 y) CONTROL GROUP: 26 asymptomatic subjects (F 20, M 6; mean age: 66.6 y)		**Static balance** was assessed on a stabilometric platform AMTI AccuGait TM 50 Hz (AMTI, Watertown, MA, USA)	Participants performed two single-leg stance tests (left followed by right leg stance). Center of pressure displacement was analysed.	Within 2 months after surgery	No significant differences between approaches were found for fulfilment of the postural task. Subjects operated on with the anterior or Röttinger approach showed higher average CP displacement speed and path length than asymptomatic subjects. Subjects operated on through the posterior approach showed no significant differences from asymptomatic subjects.
*Wang* et al.	THR GROUP: 20 patients (F 7, M 13; mean age 48 y) HRA GROUP: 20 patients (F 11, M 9; mean age 46 y)	Each surgery was performed through a posterolateral surgical approach by the same surgical team.	**Hip joint position sense** (JPS) was tested using Active-active method in supine position.	Patients were placed in a supine position and blindfolded to eliminate visual cues. The test started at neutral position (0°) and patient actively flexed the hip toward the flexion target position of 45°, which was indicated by a mechanical obstruction. After holding that position for 5 s, patient focused and remembered the angle, and brought the joint actively back to the neutral starting position. Then, patient was asked to actively reproduce the target position without the mechanical obstruction and hold at where he/she felt it was the just position. Five repetitions were performed for each leg and “absolute angular error” values were obtained from the start and stop angles.	Preoperatively, postoperative 6, 12, 24 and 36 months.	Both groups of patients had similar absolute angle error preoperatively and decreased absolute angle error. The THA group had higher mean absolute angle error than that of HRA group at postoperative 6 month and 12 months, thereafter, both groups had similar absolute angle error. During the follow up, a tendency towards decreased absolute angle error from 6 month to 36 months was witnessed in both groups. Especially between 6 month and 12 month and between 12 month and 24 months, there were significant differences in absolute angle error. After one year, both group of patients had stable absolute angle error.
*Warenckack* et al. *2020*	THR GROUP: 30 patients. (F 25, M 5; mean age 69.4 y) CONTROL GROUP: 30 participants (F 25, M 5; mean age 68.8 y)	THR with anterolateral approach	**Dynamic balance** and functional mobility were assessed with Metitur’s Good Balance force platform. Timed Up and Go test, 3 m walk test, Functional Reach Test, 30s Chair Stand Test, Step Test and Berg Balance Scale.	Dynamic test on force platform. The dynamic test on the force platform was based on the principle of biofeedback. It was performed on 1 board (including “path”) and with different sensitivity of platform (B100 and B60). The board showed “paths” for displacement of centre of feet pressure. Patients could observe certain position of the centre of feet pressure (COP) on the screen. It was visualized as a cursor and the tasks were to achieve the targets successively displayed on the screen during displacement of the body. The subject’s position during dynamic tests was upright with feet placed parallel and 20 cm apart. TUG: During the test, patients were to rise from a chair, walk a distance of 3 m, make a turn of 180 ° having crossed a designated line and return to the chair. Recording the time of performing the task was initiated by the “start” command and stopped the moment a patient returned to the sitting position with the back resting against the chair. Patients were instructed to do the task as quickly as possible, but at the maximum speed at which the patient could walk safely without running Each participant completed three trials. 3 m walk test: Patients were instructed to stand with their toes touching the start line and walk fast beyond the taped finish line. The time from the moment their foot crossed the start line to the moment their both feet crossed the stop line was measured. Each participant completed three trials. Functional reach test: During test, the patient was standing by the wall with their feet shoulder-width apart,one shoulder flexed at 90° and the other arm on the side. A ruler was attached to the wall. Patients were instructed to reach their maximal distance (in centimetres) without moving their feet or losing balance and come back to the standing position. If subjects raised a heel or took a step during testing; the trial was repeated. Each participant completed three trials. 30s Chair Stand Test: The test consists of standing up and sitting down from a chair as many times as possible within 30 s. Initially, subjects were seated on the chair with their arms folded across the chest and with a back in an upright position. They performed only one trial and started it after a command. A standard chair with armrests was used. The step test: When tested, subjects were instructed to place one foot onto a 7.5 cm high step and then take it back down to the floor repeatedly as fast as possible. The score is the number of steps completed in the 15-s period for each lower extremity. Both sides were tested two times, with the THR group completing the test first with the operated leg (ST O) and then the non operated leg (ST N). BBS: Subjects did 14 different tasks including static tests with different feet positions and functional balance control tasks including transfer, getting up and sitting on a chair, reach, turning and stepping.	5 years after surgery	Time and distance of COP displacement were found to be higher in the study group. From the research group performed the task slower and demonstrated greater COP displacement. Also distance in the frontal plane on B100 board was significantly worse in the THR group. There was significant difference in TUG, 3 m, FRT and CST between groups. Both tests assessing gait in patients after total hip replacement showed that they completed the task slower than members of the control group. Completing the TUG test by an individual from the study group took on average 1.64 s longer than in the control group, whereas the walking time over the distance of 3 m was 0.55 s longer. In the FRT trial the reach distance of THR patients were on average 3.9 cm shorter than of the control group. Furthermore, considerable differences were observed in the CST, where members of the control group did more repetitions than subjects in the study groups. It was found that there are not statistically significant differences in the results of the BBS test between the study and the control group. Step Test results of individuals with prosthetic implants differed significantly from results in the control group. The differences were present when comparing to the control group (average 16.3) both the operated limb (mean 13.2; *p* < 0.001) and the limb without a prosthetic implant (mean 13.5; *p* < 0.002) in both cases people with total hip replacement conducted fewer repetitions.
*Warenczack* et al. *2019*	THR GROUP: 30 patients (F 25, M 5; mean age 69.4 y) CONTROL GROUP: 30 participants (F 25, M 5; mean age 68.8 y)	THR with anterolateral approach	A balance platform and a one-leg standing test (OLS) were used to assess static balance. The postural balance tests were performed on the Metitur Good Balance platform.	Static balance tests on the balance platform were conducted in several positions with different foot placement: normal standing, eyes open (NS EO) and eyes closed. The subjects were asked to maintain a motionless upright position with both arms along their sides and eyes looking at a target in front of them (barefoot). In the normal standing (NS) position the feet were placed precisely 20 cm apart. The duration of the tests with EO and EC was 30 s each. During the TP test (asymmetric position of feet) one foot was placed directly ahead of the other. We performed TP tests with the left foot in front (TLF) and with the right foot in front (TRF) separately. In the THR group, the placement of the operated limb in the front stance was called the TOF test, while the placement of the other limb in the front stance was called the TNF test. Time of test: 10 s. In 2TP the left or right foot was alternately placed in front of the other, but the feet were placed on both sides of a line that divided the platform into two parts (the line was tangent to the medial edge of the feet). Similarly, to the tandem test, the patients were examined with the operated (2TOF) and non-operated limb (2TNF) in the front. The length of the exercise was 20 s. When testing the leg standing position on the balance platform, the patients were asked raise one foot to the mid-calf level of the supporting leg but not touch the loaded limb. The test was stopped when the subject had to use the arms (touched the handrail) or used the raised foot (touched the floor). The procedure was usually stopped after two failed attempts or fear of falling and results were not recorded. Patients were examined when standing on the operated (1O) or non-operated limb (1 N) and the control group on the left (1 L) and right (1R) limb. Time of test: 5 s. During the one-leg standing test (OLS) the time of maintaining this position by the patients was measured. The placement of the limbs was the same as in the balance platform test. The subjects performed three attempts on each lower limb. The test ended when the subject used the raised foot (touched the floor) or moved the supporting leg on the ground or a significant loss of balance was observed or a maximum of 60 s elapsed. The patients performed the tests both on the operated (OLS-O) and non-operated (OLS-N) limb and the control group on the left (OLS-L) and right (OLS-R) limb.	At the beginning of the rehabilitation process.	Significant imbalance in the sagittal plane during normal standing EO and EC positions were found in the THR group. No significant differences in the measured parameters were found during tests in tandem, the second form of tandem and one-leg standing positions in the groups. The mean time of standing on the operated limb in the THR group during the OLS test was significantly shorter than that in the control group.

**Table 2:** Balance and proprioception training

*Authors*	*Participants*	*Type of surgery*	*Training*	*Volume, duration and Time of the training*	*Assessments* *(and time of assessment)*	*Protocol*	*Results*
*Aprile* et al.	THR EXPERIMENTAL: 36 patients (26 F, 10 M; mean age: 68.4 y) THR CONTROL: 28 patients (18 F, 10 M; mean age: 63.9 y)	Not reported.	THR EXPERIMENTAL. Group treatment sessions (3 or 4 patients) on the stabilometric platform. The technological experimental protocol consisted of a series of rehabilitative paths proposed automatically by the software and designed to improve the perceptive conditions of each movement. The difficulty of the exercise was gradually increased when the patient’s condition allowed it. THR CONTROL: group treatment sessions (3 or 4 patients). The treatment included: techniques to improve joint range of motion, muscle force, ability to adopt different postures and proprioceptive exercises.	45 min sessions, 5 times/week, for 4 weeks, in the post-surgical rehabilitation	Instrumental assessment of **postural stability** was performed using bipodalic platform (Prokin, Technobody, Italy). The system provides, at 40 Hz, the coordinates of the subject’s Centre of Pressure (CoP) and a biaxial accelerometer measures trunk tilts in the antero-posterior and medio-lateral directions. All outcome measures were administered before (T0) and after treatment (T1).	For the stabilometric assessment, with the platform in a blocked position, the following parameters were considered: area and perimeter of the CoP, anteroposterior and mediolateral velocity of the CoP with the eyes open and closed. The anteroposterior, mediolateral and total root mean square (RMS) of the trunk movements (with eyes open and closed) were also calculated to measure the stability of the trunk. The dynamic assessment consisted of an evaluation of global proprioceptive control and postural instability with the platform in an unblocked position, the level of the damper was set according to each subject’s physical characteristics. The following parameters were considered: total, anteroposterior and mediolateral dynamic stability indexes and their relative RMS.	Greater improvement in the experimental group than in the control group in the following stabilometric variables: total RMS trunk with eyes opened (*p* = 2460.024), mediolateral RMS trunk with eyes opened (*p* = 0.030) and Romberg Area opened eyes/closed eyes (*p* = 0.029). Greater improvement in the experimental group than in the control group in the following dynamic variables: Total Dynamic Stability Index (*p* = 0.003), Anteroposterior Dynamic Stability Index (*p* = 0.048) and Mediolateral Dynamic Stability Index (*p* = 0.001).
*Bitterli* et al.	THR EXPERIMETAL: 41 patients (19 F, 22 M; mean age: 65.3 y) THR CONTROL: 39 patients (12 F, 27 M; mean age: 68.4 y)	THR with lateral trans-gluteal approach	THR EXPERIMENTAL: The training programme was a so-called minimal intervention strategy, demanding minimal training effort exercises. Six exercises were performed. In supine position: 1 = Tense muscles of legs and buttocks; 2 = Move affected leg out to side and back on supporting surface; 3 = Raise knees, move foot backwards and forwards on supporting surface; 4 = Make a “bridge” (raise buttocks from supporting surface). Performed while standing: 5 = Stand upright with legs slightly apart, bend hips and knees and then straighten up again; 6 = Stand on unaffected leg and move other leg out to the side and back. THR CONTROL: no exercises.	The training was performed before surgery.10 repetition of each exercise performed twice each day, for a duration from 2 to 6 weeks.	**Balance** was assessed with the Biodex Balance System (BBS; produced by Biodex Medical Systems, New York). Assessment was performed before surgery, and 4 and 12 months after surgery.	In the static mode the BBS measures the angular displacement of the centre of gravity. From the degrees of tilt about the anterior-posterior and medial-lateral axes, the anterior-posterior stability index (APSI), the medial-lateral stability index (MLSI) and the overall stability index (OSI) is calculated. The participants received support from visual feedback displayed on a screen. Each test lasted 20 s. The participants completed 3 trial repetitions prior to the actual test, to rule out short-term learning effects. The test was performed standing barefoot in the most comfortable position.	TR showed better mean balance ability than CO before the THEP, regarding both the overall stability index (M = 2.34, SD = 0.55) compared to CO (M = 2.62, SD = 0.81) and the medial-lateral stability index (M = 1.58, SD = 0.48 and M = 1.90, SD = 0.72 respectively). No significant differences between the two groups were found at the 4-month and the one-year follow-up point.
*Nelson* et al.	THR EXPERIMENTAL:35 patients (23 F, 12 M; mean age: 62 y) THR EXPERIMENTAL:35 patients (21 F, 14 M; mean age: 67 y)	93% of patients received a posterior approach.	THR EXPERIMENTAL: received a standard Home Exercise Program delivered through a telerehabilitation system for the first six weeks after discharge. THR CONTROL received a standard Home Exercise Program for the first six weeks after discharge. The standard protocol consisted of strengthening exercises for quadriceps, hip abductors, extensors, and flexors. At At two, four, and six weeks post-operatively all participants attended a one-to-one physiotherapy session focussing on gait and reviewing and progressing their HEP. The experimental group attended it via telemedicine while control group attended it in outpatient setting. After six weeks all participants were provided with a paper-based HEP to continue independently. All sessions beyond the six-week intervention period were in-person appointments, regardless of allocation to the control or intervention group.	Standardised HEP three times daily for six weeks. **Six post-operatively weeks**	**Dynamic balance** was assessed via the step test and Timed-up-and-go (TUG) tests. Outcomes were collected at baseline (pre-operatively), discharge from inpatient physiotherapy, six weeks and six months post-operatively.	The step test was performed standing on the study leg the entire time, while the other leg was moved back and forth from the step to the floor (eg, the stepping foot was placed flat up onto the step, then back down flat onto the ground) as many times as possible in 15 s without overbalancing (moving the stance leg from the start position). During the TUG the patients were required to rise from a chair of standard height, walk 3 m, turn 180°, return to the chair, and sit down.	No between group difference were found. TUG and step test showed significant improvement over time in both groups.
*Pethe-Kania* et al.	THR EXPERIMENTAL: 30 patients (19 F, 11 M; mean age: 61.4 y) THR CONTROL: 30 patients (18 F, 12 M; mean age: 65.1 y)	?	THR EXPERIMENTAL: Standardized rehabilitation + follow-up posturography with an adaptively modified biofeedback. The training was based on performance of the visually stimulated exercises on a double-plate posturographic platform. While standing on the plat-form patients were supposed to sway their body in such a way that the scaled position of the trained per-son’s Center of Pressure (COP) visualized on the computer screen coincided as closely as possible witht he moving point representing the visual stimulus. The COP constitutes a good approximation of the patient’s center of gravity projected onto the supporting plane (the platform). During the training the position of the visualized COP marker is being scaled according to the value of the biofeedback coefficient evaluated in the static posturography examination just before the training session is started. If in such an examination a given limb is diagnosed to be underloaded, a correspondingly greater loading is imposed on it during the symmetry training session. THR CONTROL: Standardized rehabilitation	Between 3 and 6 months after the operation. 6 and 5 times a week, for a total of 21 days	Assessment of the lower limb loading symmetry was performed using a double-plate posturographic platform. The limb loading symmetry evaluation was carried out before and after 3-week hospital rehabilitation.	During the examinations patients were supposed to stand still on the platform for a pe-riod of 30 s (having left foot positioned on the left plate of the platform and the right foot on the right plate). The examinations were conducted in both open and closed eyes scenarios. The essence of the performed limb loading symmetry evaluation boils down to a precise measurement of the average weight exerted on each plate of the posturographic platform. Analysis of the COP trajectories registered individually for the left and right leg were also performed.	The eyes-open static posturography examinations indicated significantly improvement in the lower limb loading symmetry in 29 (97%) patients from the experimental group (*p* = 0.000003). In the control group, such an improvement was observed in 20 (67%) patients (*p* = 0.034796). In the eyes closed examinations correction in the limb loading symmetry was evident in 23 (77%) patients from the experimental group (*p* = 0.000247) and 18 (60%) patients from the control group (*p* = 0.043327).
*Shabana* et al.	THR EXPERIMENTAL: 10 patients (3 F, 7 M; mean age: 61.9 y) THR CONTROL: 10 patients (4 F, 6 M; mean age: 58.4 y)	?	THR EXPERIMENTAL: received dynamic balance training program in addition to traditional rehabilitation programme. The balance training consisted in standing over an unstable board. THR CONTROL: received traditional rehabilitation program only in form of therapeutic exercise, transfer training and gait training.	From immediate post-surgery for 12 weeks, three times per week	Balance was assessed at the beginning and at the 6th & 12th week post operatively.	The assessment of balance was performed by means of a pre-determined protocol of the Biodex Stability System (BSS). Patients were instructed to step onto the platform of the BSS with the knee of the supported leg flexed about 10 degrees. In addition, the subject was instructed to keep his hands at his sides throughout the test. A single limb test was conducted. The test consisted of 30 s test using all eight levels of instability provided by the system.	The experimental group showed a statistical improvement in the mean Biodex overall stability index at 6 weeks and 12 weeks interval compared to the initial. The control group did not show any improvement across time.
*Trudelle-Jackson* et al.	THR EXPERIMENTAL: 14 patients (6 F, 8 M; mean age: 59.4 y) THR CONTROL: 14 patients (9 F, 5 M; mean age: 59.6 y)	THR with antero-lateral approach	THR EXPERIMENTAL: a set of 7 weight-bearing exercises: sit to stand, unilateral heel raises, partial knee bends, 1-legged standing balance, knee raises with alternating arm raises (marching), side and back leg raises in standing, and unilateral pelvic raising and lowering in standing. No resistance was added to any of the exercises, and abdominal contraction was emphasized during all weight-bearing exercises to promote trunk stability. THR CONTROL: The exercise protocol for the control group consisted of 7 basic isometric and active ROM exercises: gluteal muscle sets, quadriceps sets, hamstring sets, ankle pumps, heel slides, hip abduction in supine, and internal and external rotation in supine.	15 to 20 repetitions of each exercise, 3 to 4 times a week for 8 weeks of training, 4 to 12 months after THA.	**Postural stability** was assessed using the BEP-IV force platform (Human Performance Measurement [HPM]a). Assessments were performed pre-and post- the exercise intervention.	Stability was measured as subjects attempted to stand steadily on the involved lower extremity while holding the opposite leg in full hip extension and 90°of knee flexion with eyes open. Subjects did not wear shoes during the testing. The recording lasted 10 s. The BEP-IV postural stability measurement system uses a lightweight, portable force platform to measure ML stability, AP stability, and total stability by tracking changes in the centre of pressure (COP). The ratio of average movement of the COP to the size and placement of the stance foot was calculated. The resulting normalized score represents a “percentage instability” score.	Postural stability improved 36.8% in the THR experimental group (from 66.1 to 90.4) and 0.9% in the THR control group (from 76.3 to 77.0).
*Winther* et al.	THR EXPERIMENTAL: 14 patients (6 F, 8 M; mean age: 59.4 y) THR CONTROL: 14 patients (9 F, 5 M; mean age: 59.6 y)	THR with posterior approach	THR EXPERIMENTAL: The training consisted of leg press and abduction performed by the operated leg. THR CONTROL: The patients in the received conventional physiotherapy, consisting of different types of strength exercises with low or no external load (10–20 repetitions). Warm-up exercises were mainly cycling, step, and treadmill walking. Other workouts used were aquatic exercises, balance training, range-of motion exercises, massage, and sling exercises.	THR: EXPERIMENTAL: 3 days a week, 4–5 repetitions × 4 series with a load equal to 85–95% of one-repetition maximum. THR CONTROL: 3 days a week, 10–20 repetitions. Intervention lasted 3 months.	At 3, 6, and 12 mos postoperatively, **postural sway** was evaluated in two gait tests; ie, one test before and one test after conducting a battery of physical performance tests.	Postural sway in the test before (TB) and test after (TA) conducting a battery of individually validated physical performance tests that resemble daily living activities. First, an initial walking test (the TB) was conducted as each patient walked back and forth along a 5-m OptoGait walkway, a floor-based photocell system with a validated electronic walkway system for movement analysis (Microgate Bolzano, Italy), Body sway was assessed using a validated body-worn inertial measurement tool (Gyko Interial System; Microgate, Bolzano, Italy) placed in a belt at the lower back, as described by the manufacturer.	At 3 mos postoperatively, postural sway in the test after was significantly higher for the conventional rehabilitation group than the maximal strength training group; however, there was no between-group difference at the test before. Postural sway was also significantly higher in the test after compared with the test before in the conventional rehabilitation group. No difference was found between the test before and test after in the maximal strength training group. At 6 and 12 mos postoperatively, there were no statistically significant within- or between-group differences in postural sway.
*Zeng* et al.	TRAINING GROUP: 32 patients (F 15, M.1; mean age 65,19 y) CONTROL GROUP: 27 patients (F 13, M: 14; mean age 64,81 y)	THR with anterolateral approach	TG: Tai Chi training, hip muscle strengthens training and ROM training. 10 simplified forms of TC exercise procedures included: Opening Form, Parting Wild Horse’s Mane, Apparent Close Up, wave hand like clouds, Step back and whirl arms on both sides, Grasp the Sparrow’s Tail, Brush Knee and Push, Golden Rooster Stands on One Leg, Heel Kick, and Cross Hands (with Closing form). CG and TG: Both received a standardized postoperative exercise program.	45–60 min Tai Chi training, 20–30 min hip muscle strengthens training and ROM training. 5 times per week for 12 weeks before scheduled THA.	Unipedal stance test (UPST); Timed-up-and-go (TUG) tests. Tests were performed within three days after they were allocated into the study, within three days preoperatively and Week 13 and Week 26 postoperatively.	TUG: During the TUG the patients were required to rise from a chair of standard height, walk 3 m, turn 180°, return to the chair, and sit down. UPST: Patients stood on the preferred leg with the shoes off, placed their arms across chest with hands touching their shoulders and did not let legs touch each other. Look straight ahead with eyes open and focus on an object about 1 m in front of body. A stopwatch was used to record in seconds the duration of standing.	Significant improvement in both TUG and UPST were found. In TG after exercise program the improvement maintained to Week 26 post-operation.

### Deviations from the study protocol registered in PROSPERO database

Two adjustments with respect to the original study protocol registered on PROSPERO database should be mentioned. First, the Downs and Black checklist was adopted instead of previously planned tool as it can be adapted to both intervention and non-intervention studies, and thus allowed to use a single tool for all the studies included in the present review. This choice was not made on the basis of the results of methodological assessment, rather on the willingness to provide better clarity in the reporting and the discussion of the risk of bias assessment in the text of the manuscript. The assessment with the Downs & Black checklist and the assessment with previously planned tools was performed and the results showed comparable outcomes between the tools. Detailed information on the assessment and the comparison between the tools is reported in Additional file 3: Appendix 3. In addition, in this review there was also an attempt to analyse differences between patients undergoing different surgical approaches for THA, and this was not planned in the original study protocol. However, it was deemed appropriate to extract also these data since this information is highly useful for clinical practice and for the setting-up of the rehabilitation programs.

## Results

### Search results

According to the PRISMA flow-chart (Fig. 1), a total of 1780 articles were retrieved from the initial literature search. After removal of duplicates and of studies not meeting inclusion and exclusion criteria, 48 articles were considered eligible for a full-text review. Five articles [27, 28, 29–32] were excluded because the results from patients with THA were mixed to results of patients operated on for other orthopaedic conditions. One study was excluded because it was a reliability study [30], and another one was excluded because it did not report post-surgical data on balance assessment [33]. At the end of the selection protocol, 41 articles were included and evaluated for systematic review; of these, 33 were assessment studies [11, 13, 34–64], while the remaining 8 were rehabilitation training studies [65–72]. A summary of the data extracted from each article is reported in Table 1 and Table 2.

### Studies quality and risk of bias

**Fig. 2:**
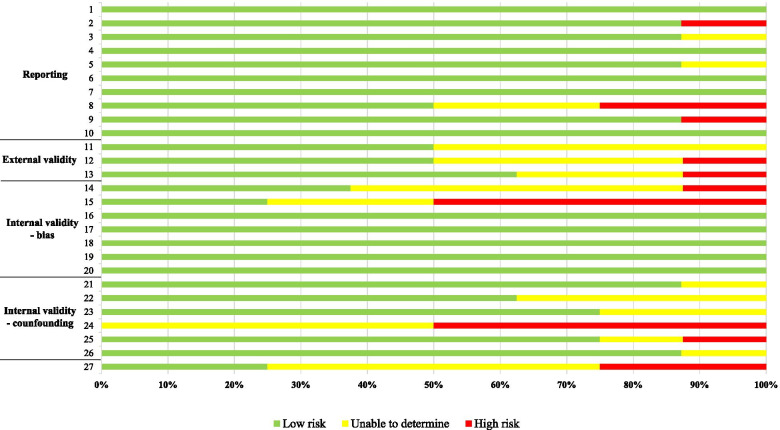
Risk of bias across studies on balance and proprioception impairments and assessment, express as a percentage. Data are provided for each item of each domain of the modified Downs and Black checklist

**Fig. 3:**
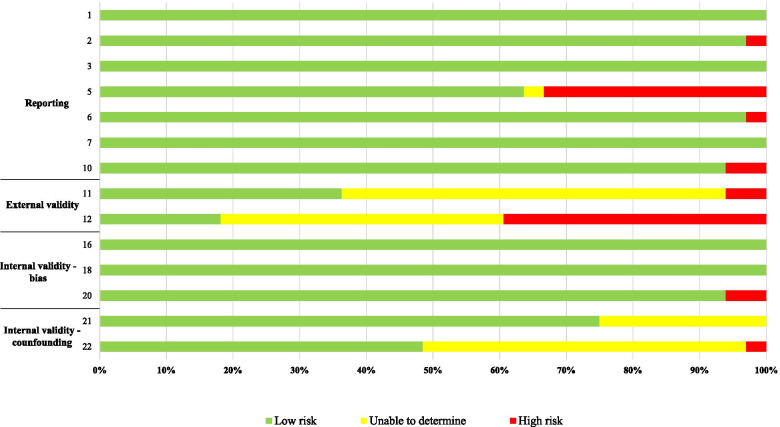
Risk of bias across studies on rehabilitation training interventions for balance and proprioception, express as a percentage. Data are provided for each item of each domain of the Downs and Black checklist

The scores of the modified Quality Index for the included studies ranged between 64.2 and 100%. A total of 2 studies were of moderate quality (score 60–74%), and 31 were of high quality (score ≥ 75%), while the remaining 8 articles were of low quality (score < 60%). The risk of bias score for each individual study is reported in Additional file 2: Appendix 2. A graphical representation of the risk of bias across all the studies reporting balance and proprioception impairments and assessments is provided in Fig. 2, while risk of bias across studies investigating rehabilitation training for balance is represented in Fig. 3. The majority of the papers included in this review (31 out of 41) were of high quality, thus showing a low risk of bias. However, regarding articles on balance and proprioception assessment, sources of high risk of bias were observed regarding the items 5, 12 and 21 of the checklists, which referred to the reporting of confounders among the patients recruited (reporting bias), the representativity of the participants recruited with respect to the whole population (external validity) and the common source population between cases and controls (selection bias), respectively. In addition, since a high percentage of study did not clearly report the required information, some concerns arise regarding items 11 and 22, i.e., the representativity of the participants which were asked to participate in the study with respect to the whole population (external validity) and the recruitment of cases and controls in the same period of time (selection bias).

Regarding studies on balance and proprioception training, sources of high risk of bias were observed regarding the items 2, 8, 9, 15 and 24, i.e., the clear description of the outcome measures (reporting bias), at least an attempt to measure adverse events (reporting bias), the description of patients lost in follow-ups (reporting bias), blinding of the assessors (internal validity bias) and the concealment of the randomized assignment to study groups of both patients and health-care staff (selection bias), respectively. No information or no information mixed with sources of high risk of bias were observed regarding items 11, 12 and 13, which referred all to as external validity of the study (i.e., the representativity of the participants which were asked to participate in the study with respect to the whole population, the representativity of the participants recruited with respect to the whole population, and the representativity of the staff, places and facilities used for the study with regards to those usually used for patients), and the items 14, 22 and 27, i.e., the blinding of patients with respect to their own group assignment (internal validity bias), the recruitment of cases and controls in the same period of time (selection bias), and the statistical power of the study, respectively.

### Balance and proprioception impairment and assessment tools

Static balance/postural stability was assessed in 20 studies [11, 35, 37–53, 63]; dynamic balance was assessed in 10 studies [34, 36, 41, 52, 54–59]; proprioception in terms of sense of joint position was assessed in 2 studies [60, 61]; clinical scales and other test batteries for balance assessment were used in 10 studies [11, 13, 40, 41, 52, 59, 60, 62–64].

#### Static balance / postural stability

Static balance, also referred to as postural stability, was assessed by double- or single-limb stance tasks on force platforms in 11 studies, which analysed centre of pressure (COP) velocity and displacement [11, 38–47]. Four of these [41–43, 47] were performed in three groups: patients with THA, patients with Hip Resurfacing (HR), and healthy age-matched controls. Two studies were performed on patients undergoing a regular size THA, and these reported a higher COP displacement in patients with THA compared to HR and healthy age-matched controls during the double-limb standing task [42, 47]. The other two studies which involved patients with large-diameter THA reported no differences in COP path length and displacement between controls, HR and THA patients [41], or a lower medial-lateral displacement in HR and THA compared to controls [43]. A better balance performance in HR and controls compared to THA was found also during the single-limb stance task [42, 47], and no differences between the three groups were found in the study involving patients with large-diameter THA [43]. Timing of assessment was heterogenous between studies, and ranged between 5- and 15-months following surgery. One study reported an increased COP displacement in THA patients 12 days after surgery in comparison to healthy matched controls [38]. The last study [39] compared 3 groups of patients within 2 months after surgery, who received 3 different surgical approaches (posterior, anterior and Röttinger approches) and a group of healthy matched controls. No significant differences between approaches were found for fulfilment of the single-limb balance tasks. However, subjects operated on with the anterior or Röttinger approach showed higher average COP displacement speed and path length than controls. Subjects operated on through the posterior approach showed no significant differences from controls.

Two studies compared COP displacement during double-limb stance, in open- and closed-eyes condition [ 44, 45]. Both studies reported in the closed-eyes condition a higher displacement of COP in THR compared to healthy age-matched controls, at 6- and 12-months post-surgery, and in a single assessment between 24- and 36-months post-surgery [44]. A significant improvement was reported between 6- and 12-months following surgery [45].

A lower displacement of the COP was reported in patients undergoing unilateral THA in comparison with patients undergoing bilateral THA [11]. Two studies having no control group reported a reduction of postural sway in the post-operative compared to pre-operative period during double-limb stance, and a higher sagittal sway compared to medial-lateral sway during single-limb or tandem stances, independently from the examined limb [ 40, 46].

One study assessed stability by means of pre-determined protocols of an instrumented platform (Biodex Balance System), and it reported an improvement in the parameters related to postural stability at 6 months after surgery compared to pre-surgery data [48]. Another study, using another instrumented device (PROPRIO 5000 machine) found that with single-limb testing of the operated limb, the HR group performed better than patients undergoing standard THA, and there was a trend for HRs to perform better than the overall THA population, but not better than large-headed THAs [49]. In the same study [49], no differences were found for postural stability in double-limb stance between patients with HR, patients with THA femoral head > 32 mm, patients with THA femoral head ≤32 mm, and the control group.

Four studies assessed the time that patients were able to spend in a single-limb stance position [ 50–52]. Two of them found a significantly shorter time in THA group compared to healthy controls for the operated side [ 51, 52], while another study without a control group of healthy participants, reported a shorter time in the operated compared to the non-operated side [ 50]. The fourth study reported a higher failure rate during the stance position in female patients compared to male patients, more than 1 year after surgery [63].

Two studies assessed the between-limbs loading difference while standing [11, 53]. A higher loading of the non-operated limb was reported in patients with unilateral THA [11, 53], while loading was more symmetrical in patients with bilateral THA [11].

In the study by Merle et al. [37], THA patients were asked to stand on two force plates in two conditions: a comfortable position, or with the requirement to load the operated limb. In the first condition, the operated limb was less loaded than the healthy limb, while in the second condition load distribution was close to symmetry, but a higher displacement along the medial-lateral axis was found for the trajectories measured under the healthy limb than under the operated limb.

Finally, balance in terms of COP displacement seems to be not affected by the position of the elbow (straight or flexed) for holding a crutch at a mean of 4 days after surgery, even if the elbow flexed increases shoulder loading [ 35].

#### Dynamic balance

Two studies investigated dynamic balance during the performance of a 5-times-sit-to-stand [54] and a 5-times-squat [55] on force plates. Within 1 month after surgery, THA patients showed a higher asymmetrical loading and higher anterior-posterior (AP) and medial-lateral (ML) COP displacement during squatting movements when compared with healthy age-matched controls [55]. Asymmetry and COP displacement were found to be significantly different from healthy controls 1 year after surgery during sit-to-stands movements [54].

Two studies investigated dynamic balance during walking by means of force platform and 3D motion analysis [56, 57]. One study reported that, compared with healthy age-matched control subjects, THA patients showed greater frontal plane (FP) center of mass-center of pressure (COM-COP) inclination angles and smaller sagittal plane angles which improved postoperatively, but remained significantly different from healthy controls [57]. The other study [56] found no differences during single-limb-support phase for within and between group comparisons for the COM projection over the base of support. During the double-limb support phase for the healthy age-matched older adults serving as a control group, the vertical projection of the COM during double-limb-support was held on average laterally towards the leading limb side, while the individuals with THA held their COM on average toward their operated limb both limb lead conditions.

One study investigated dynamic balance in response to a sudden medial-lateral perturbation in three groups of THA patients undergoing the three different surgical approaches [58]. It was found that, in the case of direct-lateral and antero-lateral exposure, Lehr’s damping ratio significantly decreased compared to the preoperative values at 6 weeks postoperatively, but it increased steadily afterwards. Lehr’s damping ratio while standing on the affected limb was significantly lower – even at 6 months postoperatively – than that of the healthy age-matched control group. In the case of posterior surgical approach, Lehr’s damping ratio continuously increased in the postoperative period and corresponded to that of the control group at 6 months after THA.

Balance in response to sudden platform inclinations and translations was investigate also in another study [34], which found no differences between THA patients 4 months after surgery and healthy age-matched controls.

Three studies investigated dynamic balance during a step test. One year after THA no differences for least number of steps, Step Test for non-surgical side and Step Test for the THA side, between patients operated with lateral approach and patients operated by the anterior approach were found [59]. The other study [41] reported that patient operated with the large-diameter head THA were able to complete the task approximately 3 s faster than the HR group (*p* = 0.001). A few numbers of steps were reported in THA patients 5 years after surgery [52].

One study investigated dynamic balance in the immediate post-surgical period during a biofeedback test based on COP displacement, and found that in THA patients Time and distance of COP displacement were higher, the performance of the task was slower and the displacement of the COP was higher compared to controls [52].

The last study [36], investigated postural sway by means of a portable system embedded with gyroscopes, applied over the low back, during the performance of gait and gait over barriers, stairs climbing and sist-to-stand tasks. It was found a progressive improvement of balance between 4 and 12 months after surgery for gait and sit-to-stand tasks parameters. At 12 months post-surgery, THA patients results approached those of healthy control group participants, however, trunk pitch (forwards-backwards) and roll (side-to-side) velocities were less stable when walking over barriers as was roll for the sit-to-stand task, indicative of a residual deficit of balance in THA patients.

#### Hip joint position sense

The study by Jo et al. [60] investigating proprioception by means of hip joint position sense reported no differences between patients undergoing THA for osteoarthritis or after hip fracture 3 months after surgery, while Wang et al. [61] reported that THA patients had higher mean absolute angle error than HR patients at 6 and 12 months from surgery; after 1 year and up to 36 months, both groups had similar absolute angle error. In either study, results were not compared with a control group of healthy age-matched participants.

#### Clinical scales and other tasks

Berg Balance Scale was used in three studies. Warenczackc et al. [52] reported no differences between THA patients and healthy age-matched controls at 5 years after surgery. Chang et al. [40] found that Berg balance test decreased significantly after 2 weeks from THA surgery and improved gradually thereafter, reaching the highest score at 6 months. Jo et al. [60] reported that 3 months after surgery BBS scores of patients undergoing THA for degenerative arthritis were significantly higher than patients undergoing THA because of fractures scores.

Activities-specific Balance Confidence Scale (ABC) was used in two studies [13, 59]. No differences were found at 1 year from surgery when patients operated on by a direct anterior and direct lateral approaches were compared [59]. Moreover, ABC scale was found not to be a predictor of falls in THA patients [13].

Timed-up-and-go was used in four studies. No differences have been reported between patients with unilateral and bilateral THA [11], patients undergoing either a direct superior approach or a posterolateral approach for surgery [64], as well as in patients undergoing large-diameter THA or HR. [41] However, THA patients were significantly slower than healthy age-matched controls [ 41, 62].

Functional reach test was used in three studies [ 40, 41, 62]. Patients with THA reached shorter distance than healthy age-matched controls when studied between 3 and 5 years after surgery [ 41, 62]. Accordingly, another study reported no significant improvements between 2 weeks and 1 year after surgery [40]. Patients undergoing HR had better performance from 3 to 12 months after surgery when compared to large-diameter heads THA patients [41].

One study reported a higher reaching distance during the Low Quarter Y-balance test in male compared to female patients, more than 1 year after surgery [63].

Risk of falls was assessed in two studies. The first study the assessment was performed by means of the Falls Risk for Older People in a Community Setting (FROP-Com) tool; the authors found no differences between patients undergoing THA with the direct anterior approach and patients undergoing THA with the direct lateral approach [59]. The second study, performed the assessment by means of the Brief BEST-test in two groups of THA patients, undergoing either a direct superior approach or a posterolateral approach for surgery [64]. A reduction in the risk of falls was observed in both groups between 1- and 3-months following surgery.

Fall rate was assessed in one study, which reported a 2.8 times higher fall rate in THA patients compared to healthy age-matched controls in the ten years after surgery [51].

The 3-m walk and 30-s chair standing tests were used by Wareczack et al. [62] 5 years after THA surgery. Patients with THA performed shorter distance and a lower number of repetitions, respectively, when compared to healthy age-matched controls.

### Balance rehabilitation training

Three studies investigated the effects of a training intervention in the immediate post-surgical rehabilitation [65, 66, 72], one study in the period between 4 and 12 months after surgery [67], and one study in the period between 3 and 6 months after surgery [70]. Four studies reported a higher increase in balance in patients undergoing balance training [65, 66, 70, 72], while the study by Nelson et al. [66] reported a similar between-groups increase in balance following a non-specific balance training.

Two studies investigated the effects of a pre-operative balance training program on the post-operative outcomes. One study found positive effects of the preoperative balance training, based on Tai-Chi practice performed up to week 26 post-surgery [68], while another study found no differences between the groups in the post-surgery [69].

Better balance abilities while walking, in terms of reduced postural sway, were found in a group of THA patients undergoing a strength training protocol in comparison with THA patients undergoing a traditional rehabilitation [71]. This result was observed after a fatiguing test battery 3 months after surgery, while no between-group differences were observed at 6 and 12 months after surgery.

All the studies referred to balance or postural stability. The term proprioception was not used.

## Discussion

### Balance and proprioception impairment and assessment tools

The first aim of this review was to investigate if patients following THA show impairments in balance and proprioception. The high heterogeneity of studies methodologies and the lack of controls groups of healthy participants, or comparisons with normative data, makes difficult drawing conclusions. However, almost all the studies comparing results of THA patients with healthy controls reports significant differences between-groups, with THA patients have worse balance performance than healthy controls. Impairments have been reported during static [38, 39, 41, 44, 47, 51, 63] and dynamic balance assessments [36, 54, 58], as well as with the assessment by means of clinical scales or tests batteries [41, 51, 62–64].Only 4 studies reported no differences between THA patients and controls in balance during the single-limb phase of walking [56], the Berg-balance scale results 5 year after surgery [62], and in response to balance perturbations [34, 58]. Further studies with control groups of healthy participants are needed to better clarify these results.

Regarding proprioception impairments, it should be mentioned that in the only two studies retrieved for this review [60, 61], the results of patients with THA were not compared to results of a control group. Further studies are strongly recommended given the essential role of proprioception for both static and dynamic balance performance. It is not possible to draw conclusion on the timing of rehabilitation in which impairments are higher. It seems that a general improvement is reported in the post-operative compared to the pre-operative period [40, 46, 48]. Some studies with more than one assessment in the post-surgical follow-up reported a balance improvement across time [45, 58, 64], while other did not [40, 46, 49]. However, there are many studies reporting balance impairments also years after surgery [40, 41, 45, 51, 54, 62, 63], and persistent differences with healthy controls despite the improvements [57], thus it is likely to think that balance impairments are never completely addressed following THA. These observations are confirmed by the results of the study by Ninomiya et al. [51], which observed a 2.8 times higher fall rate in THA patients compared to healthy controls in the 10 years after surgery. The second aim of this review was to investigate how balance and proprioception are commonly assessed in THA patients. It seems that the most used tasks for balance assessment are those investigating COP displacement or other similar parameters during double- and single-limb stance performed on force platforms or other instrumented devices (22 out of the 32 studies in this review). In general, the use of force platforms for the assessment of static and dynamic balance is well accepted in literature given the high reliability of the instrumentation [73]. In the specific case of patients undergoing THA, the use of force platforms together with other devices, such as camera for motion capture or inertial sensors, may provide also additional information on the kinematic of the operated hip joint and the whole lower limb, which may show peculiar abnormalities or compensations negatively affecting for example the non-operated limb or the whole-body posture. Further research is needed to deeply investigate these aspects and how balance changes in the long-term following THA. Regarding proprioception, it was assessed in only two studies [60, 61] by means of hip joint repositioning tasks. These tasks are mainly aimed at investigating the joint position sense. It will be useful adding other tasks to investigate proprioception during dynamic tasks. In addition, further research is needed to understand the extent to which abnormal hip proprioception affects whole-body balance above and beyond the other body functions and structures involved in balance ability. It is not possible to draw conclusion on which tests are more indicated in the different times of rehabilitation because of the heterogeneity of the studies. However, some considerations have to be mentioned regarding some of the other tests used for balance assessment. The assessment of dynamic balance by means of asymmetrical loading during squatting and sit-to-standing, as well as loading during walking, needs to consider that balance is not the only variable affecting the results of the measure. Other factors such as muscle strength, post-operative training of loading symmetry, and fear of loading the operated limb might play a role in the performance of those tasks. The same is for tasks such as the functional reach test, used as a measure of balance. Undoubtfully balance is required for the functional reach test, but also muscle strength of hip, back and in general upper body muscles play an important role for a good performance of the test. At the same time, it should be mentioned that muscle strength might contributes per se to balance abilities. In support of this observation, one of the studies included in the present review reported better balance abilities while walking in the early post-surgery in THA patients undergoing a strength training intervention in comparison with patients receiving a usual rehabilitation [71]. In addition, in another study [63] male patients showed better static and dynamic balance abilities than the female counterpart. The author concluded that this difference might be related to between-groups differences in muscle strength.

Regarding surgical approaches used for THA, it is not possible to draw conclusions regarding the best surgical approach for the performance of double- and single-limb stance, because of the contrasting results when THA, HR or large-diameter-THAs patients are compared [ 41–43, 47, 49]. Similarly, it is not possible to draw conclusions for the other functional tasks, such as the timed-up-and-go, the functional reach test or the stepping tasks, because of the paucity of studies [ 41, 62, 64]. Similar findings are observed about hip joint proprioception [61], for the differences between patients with monolateral and bilateral THAs [11, 53], and for the differences between patients undergoing THA in the traumatic and elective setting [60]. No differences have been found among patients undergoing anterior or lateral surgical approaches in terms of step test performance [59], of confidence during balance task [59], and of the risk of post-surgical falls [59]. Moreover, no differences were found for balance recovery following sudden perturbations in patients operated on using direct-lateral and antero-lateral surgical approaches, in which the dynamic balancing ability continuously improved in the first 6 months postoperatively [58]. Accordingly, no significant differences between approaches were found for the risk of falls in the first 3 months after surgery [64], as well as for fulfilment of the single-limb balance tasks 2 months following surgery [39]. However, while subjects operated on through the posterior approach showed no significant differences from controls subjects, patients operated on with the anterior or Röttinger approach showed worse balance than controls [39]. In the case of joint capsule preserving posterior approach, the dynamic balancing ability showed a more rapidly improvement across timelines compared to the other two exposures, with no differences in the long-term [59]. However, further evidence is required to confirm these observations.

### Balance rehabilitation training

Although the limited number of studies and the different methodological approach do not allow a univocal conclusion, it could be stated that balance training seems to be effective in all the phases of THA, pre-operative [68], immediate post-surgery [65, 70, 72] and medium-log-term [68, 70], if specifically structured for balance enhancement and consistent in training volume. In fact, the two studies reporting no higher benefits in the intervention group, assessed balance following a non-balance specific training [66], or after a minimal intervention strategy, demanding minimal training effort exercises [69]. Therefore, balance training interventions for patients with THA should be well structured in terms of type and volume. Just one study suggest that an early strength training intervention leads to better balance while walking in the early post-surgery [71], thus it seems beneficial for the prevention of early re-injuries. No conclusion can be drawn regarding the best training options for the different post-surgical phases.

### Limitations

The main limitation of this literature revision was that it was difficult to sum up a conclusion for a number of the investigated points since a limited number of studies were available and most of them were excluded during the selection process mainly because of low quality or because data of THA patients were mixed to data of patients with other orthopaedic impairments. Further, in a number of studies there was not a control group of healthy matched participants to make comparisons with normative data. It is paramount for future studies to eliminate sources of bias and improve studies quality. In addition, since it has been reported that balance abnormalities lead to an increase in the risk of falls [13], another point which should be investigated in future studies, is the relationship between the introduction of specific balance training interventions in the rehabilitation following THA, and the long-term effects on the risk of falls. The studies included in the present review did not report this information, thus results should be considered in light of this limitation. Finally, for some of the points discussed in this review few studies exist. Thus, caution is needed before the modification of clinical practice, and it seems thus essential to conduct further high-quality research to increase the amount of evidences.

## Conclusion

Even if a firm conclusion cannot be drawn because of the heterogeneity of the studies and the reduced number of evidences, an improvement in balance abilities is observed following THA surgery in comparison with the pre-operative performance, and balance abnormalities persists for year after surgery, with THA patients showing an increased risk for falls. Since it seems that balance training is effective in all the rehabilitation phases if specifically structured for balance enhancement and consistent in training volume, it is not clear if long-term balance impairments are related to the lack of appropriate training interventions, or to the sensory-motor impairments related to THA surgery. It remains unclear which assessments are more appropriate for the different rehabilitation phases, and if differences exist between the different surgical procedure used for THA. Moreover, only two studies exist assessing hip joint proprioception. Further research is needed to better investigate these gaps in balance and proprioception assessment and training in THA patients.

## Supplementary Information

**Additional file 1: Appendix 1.**

**Additional file 2: Appendix 2.**

**Additional file 3: Appendix 3.**

## Data Availability

All the materials related the current study are available from the corresponding author upon reasonable request.

## Abbreviations

THA: Total Hip Arthroplasty
DAA: Direct Anterior Approach
PRISMA: Preferred Reporting Items for Systematic Reviews and Meta-Analyses
COP: Center Of Pressure
HR: Hip Resurfacing
ML: Medial-Lateral
AP: Anterior-Posterior
COM: Center Of Mass
FP: Frontal Plane
ABC: Activities-specific Balance Confidence Scale
FROP-Com: Falls Risk for Older People in a Community setting
