# Supplementary material for: Balance and proprioception impairment, assessment tools, and rehabilitation training in patients with total hip arthroplasty: a systematic review
Source: BMC Musculoskelet Disord. 2021 Dec 20;22:1055. doi: 10.1186/s12891-021-04919-w (PMC8690357; doi:10.1186/s12891-021-04919-w)
Supplement: Supplementary file 2 — Additional file 2: Appendix 2. [file 12891_2021_4919_MOESM2_ESM.docx]

**Appendix 2.** Assessment of methodological quality of the included studies.

|  |  | **Reporting** | | | | | | | | | | **External Validity** | | | **Internal Validity - bias** | | | | | | | **Internal Validity - confounding** | | | | | | **Power** | **Score (%)** |
| --- | --- | --- | --- | --- | --- | --- | --- | --- | --- | --- | --- | --- | --- | --- | --- | --- | --- | --- | --- | --- | --- | --- | --- | --- | --- | --- | --- | --- | --- |
|  | | **1** | **2** | **3** | **4** | **5** | **6** | **7** | **8** | **9** | **10** | **11** | **12** | **13** | **14** | **15** | **16** | **17** | **18** | **19** | **20** | **21** | **22** | **23** | **24** | **25** | **26** | **27** |  |
| 1 | *Aprile et al. 2019* |  |  |  |  |  |  |  |  |  |  |  | ***?*** |  | ***?*** |  |  |  |  |  |  |  | **?** | ***?*** | ***?*** |  | ***?*** | ***?*** | **62.9** |
| 2 | *Batting et al. 2019** |  |  |  |  |  |  |  |  |  |  |  |  |  |  |  |  |  |  |  |  |  | ***?*** |  |  |  |  |  | **78.5** |
| 3 | *Bitterli et al. 2011* |  |  |  |  |  |  |  |  |  |  |  |  |  | ***?*** | ***?*** |  |  |  |  |  |  |  |  | ***?*** |  |  |  | **88.8** |
| 4 | *Boeer et. al. 2010** |  |  |  |  |  |  |  |  |  |  | ***?*** |  |  |  |  |  |  |  |  |  |  |  |  |  |  |  |  | **62.9** |
| 5 | *Brauner et al. 2014* |  |  |  |  |  |  |  |  |  |  |  |  |  |  |  |  |  |  |  |  |  |  |  |  |  |  |  | **92.8** |
| 6 | *Butler et al. 2015* |  |  |  |  |  |  |  |  |  |  |  | ***?*** |  |  |  |  |  |  |  |  |  | ***?*** |  |  |  |  |  | **85.7** |
| 7 | *Calò et al. 2009* |  |  |  |  |  |  |  |  |  |  | ***?*** | ***?*** |  |  |  |  |  |  |  |  | ***?*** | ***?*** |  |  |  |  |  | **42.8** |
| 8 | *Chang et al. 2015* |  |  |  |  | ***?*** |  |  |  |  |  | **?** |  |  |  |  |  |  |  |  |  |  | ***?*** |  |  |  |  |  | **71.4** |
| 9 | *Esbjörnsson et al. 2020* |  |  |  |  |  |  |  |  |  |  | **?** |  |  |  |  |  |  |  |  |  |  |  |  |  |  |  |  | **85.7** |
| 10 | *Esposito et al. 2017* |  |  |  |  |  |  |  |  |  |  | ***?*** | ***?*** | ***?*** | ***?*** | ***?*** |  |  |  | ***?*** |  | ***?*** | ***?*** |  | ***?*** |  | ***?*** | ***?*** | **48.1** |
| 11 | *Eyvazov et al. 2016* |  |  |  |  |  |  |  |  |  |  |  |  |  |  |  |  |  |  |  |  |  |  |  |  |  |  |  | **78.6** |
| 12 | *Holnapy et al. 2013* |  |  |  |  |  |  |  |  |  |  | **?** |  |  |  |  |  |  |  |  |  |  | **?** |  |  |  |  |  | **78.6** |
| 13 | *Hunter et al. 2020 a* |  |  |  |  |  |  |  |  |  |  |  | ***?*** |  |  |  |  |  |  |  |  |  | ***?*** |  |  |  |  |  | **85.7** |
| 14 | *Hunter et al. 2020 b* |  |  |  |  |  |  |  |  |  |  | ***?*** | ***?*** |  |  |  |  |  |  |  |  | **?** |  |  |  |  |  |  | **78.5** |
| 15 | *Jo et al. 2016* |  |  |  |  |  |  |  |  |  |  |  |  |  |  |  |  |  |  |  |  |  |  |  |  |  |  |  | **100** |
| 16 | *Jogi et al. 2011** |  |  |  |  |  |  |  |  |  |  | ***?*** | ***?*** |  |  |  |  |  |  |  |  | ***?*** | ***?*** |  |  |  |  |  | **71.4** |
| 17 | *Jogi et al. 2015** |  |  |  |  |  |  |  |  |  |  |  |  |  |  |  |  |  |  |  |  |  |  |  |  |  |  |  | **85.1** |
| 18 | *Jogi et al. 2016** |  |  |  |  |  |  |  |  |  |  | ***?*** | ***?*** |  |  |  |  |  |  |  |  |  |  |  |  |  |  |  | **77.8** |
| 19 | *Larkin et al. 2013* |  |  |  |  |  |  |  |  |  |  | **?** |  |  |  |  |  |  |  |  |  |  |  |  |  |  |  |  | **78.6** |
| 20 | *Lavigne et al. 2010* |  |  |  |  |  |  |  | ***?*** |  |  |  |  |  |  |  |  |  |  |  |  |  |  |  |  |  |  |  | **96.2** |
| 21 | *Lugade et al. 2008* |  |  |  |  |  |  |  |  |  |  |  | ***?*** |  |  |  |  |  |  |  |  |  | ***?*** |  |  |  |  |  | **85.7** |
| 22 | *Majewski et al. 2005* |  |  |  |  |  |  |  |  |  |  | **?** |  |  |  |  |  |  |  |  |  | **?** | **?** |  |  |  |  |  | **50.0** |
| 23 | *Merle et al. 2009* |  |  |  |  |  |  |  |  |  |  |  |  |  |  |  | ***?*** |  | ***?*** |  |  |  |  |  |  |  |  |  | **42.8** |
| 24 | *Nantel et al. 2008* |  |  |  |  |  |  |  |  |  |  | ***?*** | ***?*** |  |  |  |  |  |  |  |  |  | ***?*** |  |  |  |  |  | **71.4** |
| 25 | *Nantel et al. 2009* |  |  |  |  |  |  |  |  |  |  | ***?*** | ***?*** |  |  |  |  |  |  |  |  |  | ***?*** |  |  |  |  |  | **64.2** |
| 26 | *Nelson et al. 2020* |  |  |  |  |  |  |  |  |  |  |  |  |  |  |  |  |  |  |  |  |  |  |  |  |  |  |  | **88.9** |
| 27 | *Ninomiya et al. 2018* |  |  |  |  |  |  |  |  |  |  |  | ***?*** |  |  |  |  |  |  |  |  |  |  |  |  |  |  |  | **92.8** |
| 28 | *Pethe-Kania et al. 2017* |  |  |  |  |  |  |  |  |  |  | **?** |  | **?** | **?** |  |  |  |  |  |  | **?** | **?** |  |  |  |  |  | **55.6** |
| 29 | *Pohl et al. 2015** |  |  |  |  |  |  |  |  |  |  | ***?*** | ***?*** |  |  |  |  |  |  |  |  |  |  |  |  |  |  |  | **77.8** |
| 30 | *Pop et al. 2018* |  |  |  |  |  |  |  |  |  |  | ***?*** |  |  |  |  |  |  |  |  |  |  | ***?*** |  |  |  |  |  | **85.7** |
| 31 | *Quagliarella et al. 2011* |  |  |  |  |  |  |  |  |  |  | **?** |  |  |  |  |  |  |  |  |  |  | ***?*** |  |  |  |  |  | **71.4** |
| 32 | *Queen et al. 2021* |  |  |  |  |  |  |  |  |  |  | **?** | **?** |  |  |  |  |  |  |  |  |  |  |  |  |  |  |  | **85.7** |
| 33 | *Rasch et al. 2010* |  |  |  |  |  |  |  |  |  |  | ***?*** | ***?*** |  |  |  |  |  |  |  |  | ***?*** | ***?*** |  |  |  |  |  | **64.2** |
| 34 | *Rougier et al. 2008* |  |  |  |  |  |  |  |  |  |  | ***?*** | ***?*** |  |  |  |  |  |  |  |  | ***?*** | ***?*** |  |  |  |  |  | **57.1** |
| 35 | *Shabana et al. 2021* |  |  | ***?*** |  | ***?*** |  |  | ***?*** |  |  | ***?*** | ***?*** | ***?*** |  | ***?*** |  |  |  |  |  |  |  | ***?*** | ***?*** | ***?*** |  | ***?*** | **59.2** |
| 36 | *Slaven et al. 2012** |  |  |  |  |  |  |  |  |  |  |  |  |  |  |  |  |  |  |  |  |  |  |  |  |  |  |  | **85.7** |
| 37 | *Sliwinski et al. 2004* |  |  |  |  |  |  |  |  |  |  | ***?*** | ***?*** |  |  |  |  |  |  |  |  |  | ***?*** |  |  |  |  |  | **71.4** |
| 38 | *Szymanski et al. 2012* |  |  |  |  |  |  |  |  |  |  | **?** |  |  |  |  |  |  |  |  |  | **?** |  |  |  |  |  |  | **78.6** |
| 39 | *Talis et al. 2007* |  |  |  |  |  |  |  |  |  |  |  |  |  |  |  |  |  |  |  |  |  | ***?*** |  |  |  |  |  | **92.8** |
| 40 | *Temporiti et al. 2019* |  |  |  |  |  |  |  |  |  |  | **?** |  |  |  |  |  |  |  |  |  |  |  |  |  |  |  |  | **85.7** |
| 41 | *Trudelle-Jackson et al. 2004* |  |  |  |  |  |  |  |  |  |  |  |  |  |  |  |  |  |  |  |  |  | ***?*** |  |  |  |  |  | **85.1** |
| 42 | *Ulivi et al. 2021* |  |  |  |  |  |  |  |  |  |  |  |  |  |  |  |  |  |  |  |  |  |  |  |  |  |  |  | **100** |
| 43 | *Van Driessche et al. 2016* |  |  |  |  |  |  |  | ***?*** |  |  | ***?*** | ***?*** |  | ***?*** | ***?*** |  |  |  |  |  |  |  |  | ***?*** |  | ***?*** | ***?*** | **55.5** |
| 44 | *Wang et al. 2016* |  |  |  |  |  |  |  |  |  |  |  |  |  |  |  |  |  |  |  |  |  |  |  |  |  |  |  | **100** |
| 45 | *Wareńczak et al. 2019* |  |  |  |  |  |  |  |  |  |  |  |  |  |  |  |  |  |  |  |  |  |  |  |  |  |  |  | **100** |
| 46 | *Warenczak et al. 2020* |  |  |  |  |  |  |  |  |  |  |  |  |  |  |  |  |  |  |  |  |  |  |  |  |  |  |  | **100** |
| 47 | *Winther et al. 2021* |  |  |  |  |  |  |  | ***?*** |  |  | **?** | **?** |  | ***?*** |  |  |  |  |  |  |  |  |  | ***?*** |  |  | **?** | **74.0** |
| 48 | *Zeng et al. 2014* |  |  |  |  |  |  |  |  |  |  | **?** |  |  |  |  |  |  |  |  |  |  |  |  |  |  |  | **?** | **85.1** |

*Note:*

 *Yes;*

 *No;*

***?*** *Unable to determine.*

** Studies excluded from the qualitative synthesis.*

*Judgment and a support for the judgment for each entry is provided in the following pages.*

| **Aprile et al. 2019** | | | | |
| --- | --- | --- | --- | --- |
| **Reporting** | **YES** | **NO** | **UTD** | **Notes/Justification** |
| *1. Is the hypothesis/aim/objective of the study clearly described?* | X |  |  | The aim is clearly described. |
| *2. Are the main outcomes to be measured clearly described in the Introduction or Methods section?*  If the main outcomes are first mentioned in the Results section, the question should be answered no. | X |  |  | Clearly described in the Methods section. |
| *3. Are the characteristics of the patients included in the study clearly described?*  In cohort studies and trials, inclusion and/or exclusion criteria should be given. In case‐control studies, a case‐definition and the source for controls should be given. | X |  |  | Clearly described in the Methods section. |
| *4. Are the interventions of interest clearly described?*  Treatments and placebo (where relevant) that are to be compared should be clearly described. | X |  |  | The interventions are clearly described. |
| *5. Are the distributions of principal confounders in each group of subjects to be compared clearly described?*  A list of principal confounders is provided. | X |  |  | Confounders have been listed. |
| *6. Are the main findings of the study clearly described?*  Simple outcome data (including denominators and numerators) should be reported for all major findings so that the reader can check the major analyses and conclusions. (This question does not cover statistical tests  which are considered below). | X |  |  | The main findings are clearly described. |
| *7. Does the study provide estimates of the random variability in the data for the main outcomes?*  In non-normally distributed data the inter‐quartile range of results should be reported. In normally distributed data the standard error, standard deviation or confidence intervals should be reported. If the distribution of the data is not described, it must be assumed that the estimates used were appropriate and the question should be answered yes. | X |  |  | Variability has been reported. |
| *8. Have all important adverse events that may be a consequence of the intervention been reported?*  This should be answered yes if the study demonstrates that there was a comprehensive attempt to measure adverse events. (A list of possible adverse events is provided). | X |  |  | There was an attempt to measure adverse events by asking participants to note problems encountered during training. |
| *9. Have the characteristics of patients lost to follow‐up been described?*  This should be answered yes where there were no losses to follow‐up or where losses to follow‐up were so small that findings would be unaffected by their inclusion. This should be answered NO where a study does not report the number of patients lost to follow‐up. |  | X |  | Losses to follow-up have not been reported. |
| *10. Have actual probability values been reported (e.g. 0.035 rather than < 0.05) for the main outcomes except where the probability value is less than 0.001?* | X |  |  | Probability values have been reported. |
| **External Validity** | **YES** | **NO** | **UTD** | **Notes/Justification** |
| *11. Were the subjects asked to participate in the study representative of the entire population from which they were recruited?*  The study must identify the source population for patients and describe how the patients were selected. Patients would be representative if they comprised the entire source population, an unselected sample of consecutive patients, or a random sample. Random sampling is only feasible where a list of all members of the relevant population exists. Where a study does not report the proportion of the source population from which the patients are derived, the question should be answered as unable to determine. | X |  |  | Patients were recruited from the same hospital. |
| *12. Were those subjects who were prepared to participate representative of the entire population from which they were recruited?*  The proportion of those asked who agreed should be stated. Validation that the sample was representative would include demonstrating that the distribution of the main confounding factors was the same in the study sample and the source population. |  |  | X |  |
| *13. Were the staff, places, and facilities where the patients were treated, representative of the treatment the majority of patients receive?*  For the question to be answered yes the study should demonstrate that the intervention was representative of that in use in the source population. The question should be answered no if, for example, the intervention was  undertaken in a specialist centre unrepresentative of the hospitals most of the source population would attend. | X |  |  | Staff, places, and facilities were representative. |
| **Internal validity – bias** | **YES** | **NO** | **UTD** | **Notes/Justification** |
| *14. Was an attempt made to blind study subjects to the intervention they have received?*  For studies where the patients would have no way of knowing which intervention they received, this should be answered yes. |  |  | X | No information on blinding has been reported. |
| *15. Was an attempt made to blind those measuring the main outcomes of the intervention?* |  | X |  | Physical therapists were not blinded. |
| *16. If any of the results of the study were based on “data dredging”, was this made clear?*  Any analyses that had not been planned at the outset of the study should be clearly indicated. If no retrospective unplanned subgroup analyses were reported, then answer yes. | X |  |  | No retrospective unplanned subgroup analyses were reported. |
| *17. In trials and cohort studies, do the analyses adjust for different lengths of follow‐up of patients, or in case-control studies, is the time period between the intervention and outcome the same for cases and controls?*  Where follow‐up was the same for all study patients the answer should yes.  If different lengths of follow‐up were adjusted for by, for example, survival analysis the answer should be yes. Studies where differences in follow‐up are ignored should be answered no. | X |  |  | All patients were tested at the same time points. |
| *18. Were the statistical tests used to assess the main outcomes appropriate?*  The statistical techniques used must be appropriate to the data. For example, Non-parametric methods should be used for small sample sizes. Where little statistical analysis has been undertaken but where there is no evidence of bias, the question should be answered yes. If the distribution of the data (normal or not) is not described it must be assumed that the estimates used were appropriate and the question should be answered yes. | X |  |  | Statistics were appropriate and correctly reported. |
| *19. Was compliance with the intervention/s reliable?*  Where there was non compliance with the allocated treatment or where there was contamination of one group, the question should be answered no.  For studies where the effect of any misclassification was likely to bias any association to the null, the question should be answered yes. | X |  |  | Compliance with the interventions was reliable. |
| *20. Were the main outcome measures used accurate (valid and reliable)?*  For studies where the outcome measures are clearly described, the question should be answered yes. For studies which refer to other work or that demonstrates the outcome measures are accurate, the question should be  answered as yes. | X |  |  | Outcome measures were accurate. |
| **Internal validity ‐ confounding (selection bias)** | **YES** | **NO** | **UTD** | **Notes/Justification** |
| *21. Were the patients in different intervention groups (trials and cohort studies) or were the cases and controls (case‐control studies) recruited from the same population?*  For example, patients for all comparison groups should be selected from the same hospital. The question should be answered unable to determine for cohort and case-control studies where there is no information concerning the source of patients included in the study. | X |  |  | Yes, patients were recruited at the same hospital. |
| *22. Were study subjects in different intervention groups (trials and cohort- studies) or were the cases and controls (case‐control studies) recruited over the same period of time?*  For a study which does not specify the time period over which patients were recruited, the question should be answered as unable to determine. |  |  | X |  |
| *23. Were study subjects randomised to intervention groups?*  Studies which state that subjects were randomized should be answered yes except where method of randomisation would not ensure random allocation.  For example, alternate allocation would score no because it is predictable. |  | X |  | No information on randomization has been reported. |
| *24. Was the randomised intervention assignment concealed from both patients and health care staff until recruitment was complete and irrevocable?*  All non‐randomised studies should be answered no. If assignment was concealed from patients but not from staff, it should be answered no. |  |  | X | There is no information in the text. |
| *25. Was there adequate adjustment for confounding in the analyses from which the main findings were drawn?*  This question should be answered no for trials if: the main conclusions of the study were based on analyses of treatment rather than intention to treat; the distribution of known confounders in the different treatment groups was  not described; or the distribution of known confounders differed between the treatment groups but was not taken into account in the analyses. In non-randomized studies if the effect of the main confounders was not investigated or confounding was demonstrated but no adjustment was made in the final analyses the question should be answered as no. |  | X |  | No data adjustment has been reported. |
| *26. Were losses of patients to follow‐up taken into account?*  If the numbers of patients lost to follow‐up are not reported, the question should be answered as unable to determine. If the proportion lost to follow-up was too small to affect the main findings, the question should be answered yes. |  |  | X | No information has been reported. |
| **Power** | **YES** | **NO** | **UTD** | **Notes/Justification** |
| *27. Did the study have sufficient power to detect a clinically important effect where the probability value for a difference being due to chance is less than 5%?*  Sample sizes have been calculated to detect a difference of x% and y%. |  |  | X | No information on statistical power calculation. |
| **Total Score 17/27 = 62.9 %** | | | | |

| **Batting et al. 2019** | | | | |
| --- | --- | --- | --- | --- |
| **Reporting** | **YES** | **NO** | **UTD** | **Notes/Justification** |
| *1. Is the hypothesis/aim/objective of the study clearly described?* | X |  |  | The aim is clearly described in the abstract. |
| *2. Are the main outcomes to be measured clearly described in the Introduction or Methods section?*  If the main outcomes are first mentioned in the Results section, the question should be answered no. | X |  |  | The main outcomes are described both in the introduction and the methods section. |
| *3. Are the characteristics of the patients included in the study clearly described?*  In cohort studies and trials, inclusion and/or exclusion criteria should be given. In case‐control studies, a case‐definition and the source for controls should be given. | X |  |  |  |
| *4. Are the interventions of interest clearly described?*  Treatments and placebo (where relevant) that are to be compared should be clearly described. |  |  |  |  |
| *5. Are the distributions of principal confounders in each group of subjects to be compared clearly described?*  A list of principal confounders is provided. |  | X |  | No reference to confounders has been made. |
| *6. Are the main findings of the study clearly described?*  Simple outcome data (including denominators and numerators) should be reported for all major findings so that the reader can check the major analyses and conclusions. (This question does not cover statistical tests  which are considered below). | X |  |  | The main findings are described in results section with table 2 and figures1 and 2. |
| *7. Does the study provide estimates of the random variability in the data for the main outcomes?*  In non-normally distributed data the inter‐quartile range of results should be reported. In normally distributed data the standard error, standard deviation or confidence intervals should be reported. If the distribution of the data is not described, it must be assumed that the estimates used were appropriate and the question should be answered yes. | X |  |  | SD is reported. |
| *8. Have all important adverse events that may be a consequence of the intervention been reported?*  This should be answered yes if the study demonstrates that there was a comprehensive attempt to measure adverse events. (A list of possible adverse events is provided). |  |  |  |  |
| *9. Have the characteristics of patients lost to follow‐up been described?*  This should be answered yes where there were no losses to follow‐up or where losses to follow‐up were so small that findings would be unaffected by their inclusion. This should be answered NO where a study does not report the number of patients lost to follow‐up. |  |  |  |  |
| *10. Have actual probability values been reported (e.g. 0.035 rather than < 0.05) for the main outcomes except where the probability value is less than 0.001?* |  | X |  | Probability values have been reported only as <0.05 o <0.01. |
| **External Validity** | **YES** | **NO** | **UTD** | **Notes/Justification** |
| *11. Were the subjects asked to participate in the study representative of the entire population from which they were recruited?*  The study must identify the source population for patients and describe how the patients were selected. Patients would be representative if they comprised the entire source population, an unselected sample of consecutive patients, or a random sample. Random sampling is only feasible where a list of all members of the relevant population exists. Where a study does not report the proportion of the source population from which the patients are derived, the question should be answered as unable to determine. | X |  |  | The proportion of the source population is described in fig.1. |
| *12. Were those subjects who were prepared to participate representative of the entire population from which they were recruited?*  The proportion of those asked who agreed should be stated. Validation that the sample was representative would include demonstrating that the distribution of the main confounding factors was the same in the study sample and the source population. | X |  |  | The proportion of those who agreed to participate is described in Fig1. |
| *13. Were the staff, places, and facilities where the patients were treated, representative of the treatment the majority of patients receive?*  For the question to be answered yes the study should demonstrate that the intervention was representative of that in use in the source population. The question should be answered no if, for example, the intervention was  undertaken in a specialist centre unrepresentative of the hospitals most of the source population would attend. |  |  |  |  |
| **Internal validity – bias** | **YES** | **NO** | **UTD** | **Notes/Justification** |
| *14. Was an attempt made to blind study subjects to the intervention they have received?*  For studies where the patients would have no way of knowing which intervention they received, this should be answered yes. |  |  |  |  |
| *15. Was an attempt made to blind those measuring the main outcomes of the intervention?* |  |  |  |  |
| *16. If any of the results of the study were based on “data dredging”, was this made clear?*  Any analyses that had not been planned at the outset of the study should be clearly indicated. If no retrospective unplanned subgroup analyses were reported, then answer yes. | X |  |  | No retrospective unplanned analyses were described. |
| *17. In trials and cohort studies, do the analyses adjust for different lengths of follow‐up of patients, or in case-control studies, is the time period between the intervention and outcome the same for cases and controls?*  Where follow‐up was the same for all study patients the answer should yes.  If different lengths of follow‐up were adjusted for by, for example, survival analysis the answer should be yes. Studies where differences in follow‐up are ignored should be answered no. |  |  |  |  |
| *18. Were the statistical tests used to assess the main outcomes appropriate?*  The statistical techniques used must be appropriate to the data. For example, Non-parametric methods should be used for small sample sizes. Where little statistical analysis has been undertaken but where there is no evidence of bias, the question should be answered yes. If the distribution of the data (normal or not) is not described it must be assumed that the estimates used were appropriate and the question should be answered yes. | X |  |  | Statistics were appropriate and correctly reported. |
| *19. Was compliance with the intervention/s reliable?*  Where there was no compliance with the allocated treatment or where there was contamination of one group, the question should be answered no.  For studies where the effect of any misclassification was likely to bias any association to the null, the question should be answered yes. |  |  |  |  |
| *20. Were the main outcome measures used accurate (valid and reliable)?*  For studies where the outcome measures are clearly described, the question should be answered yes. For studies which refer to other work or that demonstrates the outcome measures are accurate, the question should be  answered as yes. | X |  |  | Outcome measures were accurate. |
| **Internal validity ‐ confounding (selection bias)** | **YES** | **NO** | **UTD** | **Notes/Justification** |
| *21. Were the patients in different intervention groups (trials and cohort studies) or were the cases and controls (case‐control studies) recruited from the same population?*  For example, patients for all comparison groups should be selected from the same hospital. The question should be answered unable to determine for cohort and case-control studies where there is no information concerning the source of patients included in the study. | X |  |  | All patients were from the same population. |
| *22. Were study subjects in different intervention groups (trials and cohort- studies) or were the cases and controls (case‐control studies) recruited over the same period of time?*  For a study which does not specify the time period over which patients were recruited, the question should be answered as unable to determine. |  |  | X | The period of time of the recruitment has not been reported. |
| *23. Were study subjects randomised to intervention groups?*  Studies which state that subjects were randomized should be answered yes except where method of randomisation would not ensure random allocation.  For example, alternate allocation would score no because it is predictable. |  |  |  |  |
| *24. Was the randomised intervention assignment concealed from both patients and health care staff until recruitment was complete and irrevocable?*  All non‐randomised studies should be answered no. If assignment was concealed from patients but not from staff, it should be answered no. |  |  |  |  |
| *25. Was there adequate adjustment for confounding in the analyses from which the main findings were drawn?*  This question should be answered no for trials if: the main conclusions of the study were based on analyses of treatment rather than intention to treat; the distribution of known confounders in the different treatment groups was  not described; or the distribution of known confounders differed between the treatment groups but was not taken into account in the analyses. In non-randomized studies if the effect of the main confounders was not investigated or confounding was demonstrated but no adjustment was made in the final analyses the question should be answered as no. |  |  |  |  |
| *26. Were losses of patients to follow‐up taken into account?*  If the numbers of patients lost to follow‐up are not reported, the question should be answered as unable to determine. If the proportion lost to follow-up was too small to affect the main findings, the question should be answered yes. |  |  |  |  |
| **Power** | **YES** | **NO** | **UTD** | **Notes/Justification** |
| *27. Did the study have sufficient power to detect a clinically important effect where the probability value for a difference being due to chance is less than 5%?*  Sample sizes have been calculated to detect a difference of x% and y%. |  |  |  |  |
| **Total Score 11/14 = 78.5** | | | | |

| **Bitterli et al. 2011** | | | | |
| --- | --- | --- | --- | --- |
| **Reporting** | **YES** | **NO** | **UTD** | **Notes/Justification** |
| *1. Is the hypothesis/aim/objective of the study clearly described?* | X |  |  | The aim is clearly described. |
| *2. Are the main outcomes to be measured clearly described in the Introduction or Methods section?*  If the main outcomes are first mentioned in the Results section, the question should be answered no. | X |  |  | Clearly described in the Methods section. |
| *3. Are the characteristics of the patients included in the study clearly described?*  In cohort studies and trials, inclusion and/or exclusion criteria should be given. In case‐control studies, a case‐definition and the source for controls should be given. | X |  |  | Clearly described in the Methods section. |
| *4. Are the interventions of interest clearly described?*  Treatments and placebo (where relevant) that are to be compared should be clearly described. | X |  |  | The interventions are clearly described. |
| *5. Are the distributions of principal confounders in each group of subjects to be compared clearly described?*  A list of principal confounders is provided. | X |  |  | Confounders have been listed. |
| *6. Are the main findings of the study clearly described?*  Simple outcome data (including denominators and numerators) should be reported for all major findings so that the reader can check the major analyses and conclusions. (This question does not cover statistical tests  which are considered below). | X |  |  | The main findings are clearly described. |
| *7. Does the study provide estimates of the random variability in the data for the main outcomes?*  In non-normally distributed data the inter‐quartile range of results should be reported. In normally distributed data the standard error, standard deviation or confidence intervals should be reported. If the distribution of the data is not described, it must be assumed that the estimates used were appropriate and the question should be answered yes. | X |  |  | Variability has been reported. |
| *8. Have all important adverse events that may be a consequence of the intervention been reported?*  This should be answered yes if the study demonstrates that there was a comprehensive attempt to measure adverse events. (A list of possible adverse events is provided). | X |  |  | There was an attempt to measure adverse events by asking participants to note problems encountered during training. |
| *9. Have the characteristics of patients lost to follow‐up been described?*  This should be answered yes where there were no losses to follow‐up or where losses to follow‐up were so small that findings would be unaffected by their inclusion. This should be answered NO where a study does not report the number of patients lost to follow‐up. | X |  |  | Losses to follow-up were small. |
| *10. Have actual probability values been reported (e.g. 0.035 rather than < 0.05) for the main outcomes except where the probability value is less than 0.001?* | X |  |  | Probability values have been reported. |
| **External Validity** | **YES** | **NO** | **UTD** | **Notes/Justification** |
| *11. Were the subjects asked to participate in the study representative of the entire population from which they were recruited?*  The study must identify the source population for patients and describe how the patients were selected. Patients would be representative if they comprised the entire source population, an unselected sample of consecutive patients, or a random sample. Random sampling is only feasible where a list of all members of the relevant population exists. Where a study does not report the proportion of the source population from which the patients are derived, the question should be answered as unable to determine. | X |  |  | Patients were recruited from the same hospital. |
| *12. Were those subjects who were prepared to participate representative of the entire population from which they were recruited?*  The proportion of those asked who agreed should be stated. Validation that the sample was representative would include demonstrating that the distribution of the main confounding factors was the same in the study sample and the source population. | X |  |  | A large proportion of patients participate in the study; thus the sample can be considered representative. |
| *13. Were the staff, places, and facilities where the patients were treated, representative of the treatment the majority of patients receive?*  For the question to be answered yes the study should demonstrate that the intervention was representative of that in use in the source population. The question should be answered no if, for example, the intervention was  undertaken in a specialist centre unrepresentative of the hospitals most of the source population would attend. | X |  |  | Staff, places, and facilities were representative. |
| **Internal validity – bias** | **YES** | **NO** | **UTD** | **Notes/Justification** |
| *14. Was an attempt made to blind study subjects to the intervention they have received?*  For studies where the patients would have no way of knowing which intervention they received, this should be answered yes. |  |  | X | No information on blinding has been reported. |
| *15. Was an attempt made to blind those measuring the main outcomes of the intervention?* |  |  | X | No information on blinding has been reported. |
| *16. If any of the results of the study were based on “data dredging”, was this made clear?*  Any analyses that had not been planned at the outset of the study should be clearly indicated. If no retrospective unplanned subgroup analyses were reported, then answer yes. | X |  |  | No retrospective unplanned subgroup analyses were reported. |
| *17. In trials and cohort studies, do the analyses adjust for different lengths of follow‐up of patients, or in case-control studies, is the time period between the intervention and outcome the same for cases and controls?*  Where follow‐up was the same for all study patients the answer should yes.  If different lengths of follow‐up were adjusted for by, for example, survival analysis the answer should be yes. Studies where differences in follow‐up are ignored should be answered no. | X |  |  | All patients were tested at the same time points. |
| *18. Were the statistical tests used to assess the main outcomes appropriate?*  The statistical techniques used must be appropriate to the data. For example, Non-parametric methods should be used for small sample sizes. Where little statistical analysis has been undertaken but where there is no evidence of bias, the question should be answered yes. If the distribution of the data (normal or not) is not described it must be assumed that the estimates used were appropriate and the question should be answered yes. | X |  |  | Statistics were appropriate and correctly reported. |
| *19. Was compliance with the intervention/s reliable?*  Where there was non compliance with the allocated treatment or where there was contamination of one group, the question should be answered no.  For studies where the effect of any misclassification was likely to bias any association to the null, the question should be answered yes. | X |  |  | Compliance with the interventions was reliable. |
| *20. Were the main outcome measures used accurate (valid and reliable)?*  For studies where the outcome measures are clearly described, the question should be answered yes. For studies which refer to other work or that demonstrates the outcome measures are accurate, the question should be  answered as yes. | X |  |  | Outcome measures were accurate. |
| **Internal validity ‐ confounding (selection bias)** | **YES** | **NO** | **UTD** | **Notes/Justification** |
| *21. Were the patients in different intervention groups (trials and cohort studies) or were the cases and controls (case‐control studies) recruited from the same population?*  For example, patients for all comparison groups should be selected from the same hospital. The question should be answered unable to determine for cohort and case-control studies where there is no information concerning the source of patients included in the study. | X |  |  | Yes, patients were recruited at the same hospital. |
| *22. Were study subjects in different intervention groups (trials and cohort- studies) or were the cases and controls (case‐control studies) recruited over the same period of time?*  For a study which does not specify the time period over which patients were recruited, the question should be answered as unable to determine. | X |  |  | Patients were recruited over the same time period. |
| *23. Were study subjects randomised to intervention groups?*  Studies which state that subjects were randomized should be answered yes except where method of randomisation would not ensure random allocation.  For example, alternate allocation would score no because it is predictable. | X |  |  | Participants were randomly allocated to one of the groups. |
| *24. Was the randomised intervention assignment concealed from both patients and health care staff until recruitment was complete and irrevocable?*  All non‐randomised studies should be answered no. If assignment was concealed from patients but not from staff, it should be answered no. |  |  | X | There is no information in the text. |
| *25. Was there adequate adjustment for confounding in the analyses from which the main findings were drawn?*  This question should be answered no for trials if: the main conclusions of the study were based on analyses of treatment rather than intention to treat; the distribution of known confounders in the different treatment groups was  not described; or the distribution of known confounders differed between the treatment groups but was not taken into account in the analyses. In non-randomized studies if the effect of the main confounders was not investigated or confounding was demonstrated but no adjustment was made in the final analyses the question should be answered as no. | X |  |  | Distribution of potentially confounders was the same between the groups. |
| *26. Were losses of patients to follow‐up taken into account?*  If the numbers of patients lost to follow‐up are not reported, the question should be answered as unable to determine. If the proportion lost to follow-up was too small to affect the main findings, the question should be answered yes. | X |  |  | Lost in follow-up was small. |
| **Power** | **YES** | **NO** | **UTD** | **Notes/Justification** |
| *27. Did the study have sufficient power to detect a clinically important effect where the probability value for a difference being due to chance is less than 5%?*  Sample sizes have been calculated to detect a difference of x% and y%. | X |  |  | The study has sufficient power. |
| **Total Score 24/27 = 88.8 %** | | | | |

| **Boeer et. al. 2010** | | | | |
| --- | --- | --- | --- | --- |
| **Reporting** | **YES** | **NO** | **UTD** | **Notes/Justification** |
| *1. Is the hypothesis/aim/objective of the study clearly described?* | X |  |  | The aim is clearly described. |
| *2. Are the main outcomes to be measured clearly described in the Introduction or Methods section?*  If the main outcomes are first mentioned in the Results section, the question should be answered no. | X |  |  | Clearly described in the Measuring Device section. |
| *3. Are the characteristics of the patients included in the study clearly described?*  In cohort studies and trials, inclusion and/or exclusion criteria should be given. In case‐control studies, a case‐definition and the source for controls should be given. | X |  |  | Clearly described in the Partecipants and Methods section |
| *4. Are the interventions of interest clearly described?*  Treatments and placebo (where relevant) that are to be compared should be clearly described. | X |  |  | The interventions are clearly described. |
| *5. Are the distributions of principal confounders in each group of subjects to be compared clearly described?*  A list of principal confounders is provided. |  | X |  | Confounders haven’t been listed. |
| *6. Are the main findings of the study clearly described?*  Simple outcome data (including denominators and numerators) should be reported for all major findings so that the reader can check the major analyses and conclusions. (This question does not cover statistical tests  which are considered below). |  | X |  | Results have been pooled between THA and hip arthritis patients. it is not possible to check data related only to THA patients (which is the aim of this review). |
| *7. Does the study provide estimates of the random variability in the data for the main outcomes?*  In non-normally distributed data the inter‐quartile range of results should be reported. In normally distributed data the standard error, standard deviation or confidence intervals should be reported. If the distribution of the data is not described, it must be assumed that the estimates used were appropriate and the question should be answered yes. | X |  |  | The distribution of the data is not described, it must be assumed that the estimates used were appropriate |
| *8. Have all important adverse events that may be a consequence of the intervention been reported?*  This should be answered yes if the study demonstrates that there was a comprehensive attempt to measure adverse events. (A list of possible adverse events is provided). | X |  |  | Some measures to counteract adverse events of the training have been reported. (“The TG did Hip School activities once per week, supervised by a physiotherapist. In addition to the training within the group, participants performed balance exercises, using balance pads on their own at home several times a week”) |
| *9. Have the characteristics of patients lost to follow‐up been described?*  This should be answered yes where there were no losses to follow‐up or where losses to follow‐up were so small that findings would be unaffected by their inclusion. This should be answered NO where a study does not report the number of patients lost to follow‐up. | X |  |  | there were no losses to follow‐up |
| *10. Have actual probability values been reported (e.g. 0.035 rather than < 0.05) for the main outcomes except where the probability value is less than 0.001?* | X |  |  | Probability values have been reported. |
| **External Validity** | **YES** | **NO** | **UTD** | **Notes/Justification** |
| *11. Were the subjects asked to participate in the study representative of the entire population from which they were recruited?*  The study must identify the source population for patients and describe how the patients were selected. Patients would be representative if they comprised the entire source population, an unselected sample of consecutive patients, or a random sample. Random sampling is only feasible where a list of all members of the relevant population exists. Where a study does not report the proportion of the source population from which the patients are derived, the question should be answered as unable to determine. |  |  | X | Patients were recruited by means of a large physical therapy provider.  “We used a quasi-randomization method, with the criterion being the month they applied for the Hip School Program” |
| *12. Were those subjects who were prepared to participate representative of the entire population from which they were recruited?*  The proportion of those asked who agreed should be stated. Validation that the sample was representative would include demonstrating that the distribution of the main confounding factors was the same in the study sample and the source population. |  | X |  | Confounders haven’t been listed and thus there is no validation that the sample was representative, in particular there is no demonstration that the distribution of the main confounding factors was the same in the study sample and the source population. |
| *13. Were the staff, places, and facilities where the patients were treated, representative of the treatment the majority of patients receive?*  For the question to be answered yes the study should demonstrate that the intervention was representative of that in use in the source population. The question should be answered no if, for example, the intervention was  undertaken in a specialist centre unrepresentative of the hospitals most of the source population would attend. | X |  |  | Patients have been trained in group and at home, and this is representative of the treatment that the majority of patients receives. |
| **Internal validity – bias** | **YES** | **NO** | **UTD** | **Notes/Justification** |
| *14. Was an attempt made to blind study subjects to the intervention they have received?*  For studies where the patients would have no way of knowing which intervention they received, this should be answered yes. | X |  |  | The CG was tested 12 weeks prior to the start of the scheduled Hip School Program and remained without any specific training during 12w observation period |
| *15. Was an attempt made to blind those measuring the main outcomes of the intervention?* |  | X |  | There is no information about it. |
| *16. If any of the results of the study were based on “data dredging”, was this made clear?*  Any analyses that had not been planned at the outset of the study should be clearly indicated. If no retrospective unplanned subgroup analyses were reported, then answer yes. | X |  |  | No retrospective unplanned subgroup analyses were reported. |
| *17. In trials and cohort studies, do the analyses adjust for different lengths of follow‐up of patients, or in case-control studies, is the time period between the intervention and outcome the same for cases and controls?*  Where follow‐up was the same for all study patients the answer should yes.  If different lengths of follow‐up were adjusted for by, for example, survival analysis the answer should be yes. Studies where differences in follow‐up are ignored should be answered no. | X |  |  | the time period between the intervention and outcome is the same for cases and controls |
| *18. Were the statistical tests used to assess the main outcomes appropriate?*  The statistical techniques used must be appropriate to the data. For example, Non-parametric methods should be used for small sample sizes. Where little statistical analysis has been undertaken but where there is no evidence of bias, the question should be answered yes. If the distribution of the data (normal or not) is not described it must be assumed that the estimates used were appropriate and the question should be answered yes. | X |  |  | Statistics were appropriate and correctly reported. |
| *19. Was compliance with the intervention/s reliable?*  Where there was non compliance with the allocated treatment or where there was contamination of one group, the question should be answered no.  For studies where the effect of any misclassification was likely to bias any association to the null, the question should be answered yes. | X |  |  | Compliance with the interventions was reliable. |
| *20. Were the main outcome measures used accurate (valid and reliable)?*  For studies where the outcome measures are clearly described, the question should be answered yes. For studies which refer to other work or that demonstrates the outcome measures are accurate, the question should be  answered as yes. | X |  |  | Outcome measures were accurate. |
| **Internal validity ‐ confounding (selection bias)** | **YES** | **NO** | **UTD** | **Notes/Justification** |
| *21. Were the patients in different intervention groups (trials and cohort studies) or were the cases and controls (case‐control studies) recruited from the same population?*  For example, patients for all comparison groups should be selected from the same hospital. The question should be answered unable to determine for cohort and case-control studies where there is no information concerning the source of patients included in the study. | X |  |  | Yes, patients were recruited by the same physical therapy provider. |
| *22. Were study subjects in different intervention groups (trials and cohort- studies) or were the cases and controls (case‐control studies) recruited over the same period of time?*  For a study which does not specify the time period over which patients were recruited, the question should be answered as unable to determine. |  | X |  | Patients were not recruited over the same time period. |
| *23. Were study subjects randomised to intervention groups?*  Studies which state that subjects were randomized should be answered yes except where method of randomisation would not ensure random allocation.  For example, alternate allocation would score no because it is predictable. |  | X |  | They used a quasi-randomization method, with the criterion being the month  they applied for the Hip School program |
| *24. Was the randomised intervention assignment concealed from both patients and health care staff until recruitment was complete and irrevocable?*  All non‐randomised studies should be answered no. If assignment was concealed from patients but not from staff, it should be answered no. |  | X |  | They used a quasi-randomization method, with the criterion being the month  they applied for the Hip School program |
| *25. Was there adequate adjustment for confounding in the analyses from which the main findings were drawn?*  This question should be answered no for trials if: the main conclusions of the study were based on analyses of treatment rather than intention to treat; the distribution of known confounders in the different treatment groups was  not described; or the distribution of known confounders differed between the treatment groups but was not taken into account in the analyses. In non-randomized studies if the effect of the main confounders was not investigated or confounding was demonstrated but no adjustment was made in the final analyses the question should be answered as no. |  | X |  | potential confounders has not been investigated |
| *26. Were losses of patients to follow‐up taken into account?*  If the numbers of patients lost to follow‐up are not reported, the question should be answered as unable to determine. If the proportion lost to follow-up was too small to affect the main findings, the question should be answered yes. | X |  |  | There was no follow-up. No patient dropped out. |
| **Power** | **YES** | **NO** | **UTD** | **Notes/Justification** |
| *27. Did the study have sufficient power to detect a clinically important effect where the probability value for a difference being due to chance is less than 5%?*  Sample sizes have been calculated to detect a difference of x% and y%. |  | X |  | The sample size calculation and the probability for a difference haven’t been reported. However, patients with TKA and THA have been pooled together to reach an adequate sample size for the study.  The aim of this systematic review is to investigate the effects of training only in THA patients, and thus the study has not an adequate number of participants to our purpose. |
| **Total Score 17/27 = 62.9 %** | | | | |

| **Brauner et al. 2014** | | | | |
| --- | --- | --- | --- | --- |
| **Reporting** | **YES** | **NO** | **UTD** | **Notes/Justification** |
| *1. Is the hypothesis/aim/objective of the study clearly described?* | X |  |  | The aim, hypothesis and objective are clearly described in the Introduction section. |
| *2. Are the main outcomes to be measured clearly described in the Introduction or Methods section?*  If the main outcomes are first mentioned in the Results section, the question should be answered no. | X |  |  | Clearly described in the Introduction section. |
| *3. Are the characteristics of the patients included*  *in the study clearly described?*  In cohort studies and trials, inclusion and/or exclusion criteria should be given. In case‐control studies, a case‐definition and the source for controls should be given. | X |  |  | Clearly described in the Methods section. |
| *4. Are the interventions of interest clearly described?*  Treatments and placebo (where relevant) that are to be compared should be clearly described. |  |  |  |  |
| *5. Are the distributions of principal confounders in each group of subjects to be compared clearly described?*  A list of principal confounders is provided. | X |  |  | Reported in the Introduction section. |
| *6. Are the main findings of the study clearly described?*  Simple outcome data (including denominators and numerators) should be reported for all major findings so that the reader can check the major analyses and conclusions. (This question does not cover statistical tests  which are considered below). | X |  |  | Reported in the Results section. |
| *7. Does the study provide estimates of the random variability in the data for the main outcomes?*  In non-normally distributed data the inter‐quartile range of results should be reported. In normally distributed data the standard error, standard deviation or confidence intervals should be reported. If the distribution of the data is not described, it must be assumed that the estimates used were appropriate and the question should be answered yes. | X |  |  | Standard deviation has been reported. |
| *8. Have all important adverse events that may be a consequence of the intervention been reported?*  This should be answered yes if the study demonstrates that there was a comprehensive attempt to measure adverse events. (A list of possible adverse events is provided). |  |  |  |  |
| *9. Have the characteristics of patients lost to follow‐up been described?*  This should be answered yes where there were no losses to follow‐up or where losses to follow‐up were so small that findings would be unaffected by their inclusion. This should be answered NO where a study does not report the number of patients lost to follow‐up. |  |  |  |  |
| *10. Have actual probability values been reported ( e.g. 0.035 rather than < 0.05) for the main outcomes except where the probability value is less than 0.001?* | X |  |  | Probability values have been reported. |
| **External Validity** | **YES** | **NO** | **UTD** | **Notes/Justification** |
| *11. Were the subjects asked to participate in the study representative of the entire population from which they were recruited?*  The study must identify the source population for patients and describe how the patients were selected. Patients would be representative if they comprised the entire source population, an unselected sample of consecutive patients, or a random sample. Random sampling is only feasible where a list of all members of the relevant population exists. Where a study does not report the proportion of the source population from which the patients are derived, the question should be answered as unable to determine. | X |  |  | Participants were representative. |
| *12. Were those subjects who were prepared to participate representative of the entire population from which they were recruited?*  The proportion of those asked who agreed should be stated. Validation that the sample was representative would include demonstrating that the distribution of the main confounding factors was the same in the study sample and the source population. | X |  |  | The population is representative. |
| *13. Were the staff, places, and facilities where the patients were treated, representative of the treatment the majority of patients receive?*  For the question to be answered yes the study should demonstrate that the intervention was representative of that in use in the source population. The question should be answered no if, for example, the intervention was  undertaken in a specialist centre unrepresentative of the hospitals most of the source population would attend. |  |  |  |  |
| **Internal validity – bias** | **YES** | **NO** | **UTD** | **Notes/Justification** |
| *14. Was an attempt made to blind study subjects to the intervention they have received?*  For studies where the patients would have no way of knowing which intervention they received, this should be answered yes. |  |  |  |  |
| *15. Was an attempt made to blind those measuring the main outcomes of the intervention?* |  |  |  |  |
| *16. If any of the results of the study were based on “data dredging”, was this made clear?*  Any analyses that had not been planned at the outset of the study should be clearly indicated. If no retrospective unplanned subgroup analyses were reported, then answer yes. | X |  |  | No retrospective unplanned subgroup analyses were reported. |
| *17. In trials and cohort studies, do the analyses adjust for different lengths of follow‐up of patients, or in case-control studies, is the time period between the intervention and outcome the same for cases and controls?*  Where follow‐up was the same for all study patients the answer should yes.  If different lengths of follow‐up were adjusted for by, for example, survival analysis the answer should be yes. Studies where differences in follow‐up are ignored should be answered no. |  |  |  |  |
| *18. Were the statistical tests used to assess the main outcomes appropriate?*  The statistical techniques used must be appropriate to the data. For example, Non-parametric methods should be used for small sample sizes. Where little statistical analysis has been undertaken but where there is no evidence of bias, the question should be answered yes. If the distribution of the data (normal or not) is not described it must be assumed that the estimates used were appropriate and the question should be answered yes. | X |  |  | Statistics were appropriate and correctly reported. |
| *19. Was compliance with the intervention/s reliable?*  Where there was non compliance with the allocated treatment or where there was contamination of one group, the question should be answered no.  For studies where the effect of any misclassification was likely to bias any association to the null, the question should be answered yes. |  |  |  |  |
| *20. Were the main outcome measures used accurate (valid and reliable)?*  For studies where the outcome measures are clearly described, the question should be answered yes. For studies which refer to other work or that demonstrates the outcome measures are accurate, the question should be  answered as yes. | X |  |  | Outcome measures clearly described. |
| **Internal validity ‐ confounding (selection bias)** | **YES** | **NO** | **UTD** | **Notes/Justification** |
| *21. Were the patients in different intervention groups (trials and cohort studies) or were the cases and controls (case‐control studies) recruited from the same population?*  For example, patients for all comparison groups should be selected from the same hospital. The question should be answered unable to determine for cohort and case-control studies where there is no information concerning the source of patients included in the study. |  | X |  | The control group was composed by other patients and members of the staff. The authors underline they did not individually match the reference and patient groups on age. |
| *22. Were study subjects in different intervention groups (trials and cohort- studies) or were the cases and controls (case‐control studies) recruited over the same period of time?*  For a study which does not specify the time period over which patients were recruited, the question should be answered as unable to determine. | X |  |  | Patients were recruited over the same time period. |
| *23. Were study subjects randomised to intervention groups?*  Studies which state that subjects were randomized should be answered yes except where method of randomisation would not ensure random allocation.  For example, alternate allocation would score no because it is predictable. |  |  |  |  |
| *24. Was the randomised intervention assignment concealed from both patients and health care staff until recruitment was complete and irrevocable?*  All non‐randomised studies should be answered no. If assignment was concealed from patients but not from staff, it should be answered no. |  |  |  |  |
| *25. Was there adequate adjustment for confounding in the analyses from which the main findings were drawn?*  This question should be answered no for trials if: the main conclusions of the study were based on analyses of treatment rather than intention to treat; the distribution of known confounders in the different treatment groups was  not described; or the distribution of known confounders differed between the treatment groups but was not taken into account in the analyses. In non-randomized studies if the effect of the main confounders was not investigated or confounding was demonstrated but no adjustment was made in the final analyses the question should be answered as no. |  |  |  |  |
| *26. Were losses of patients to follow‐up taken into account?*  If the numbers of patients lost to follow‐up are not reported, the question should be answered as unable to determine. If the proportion lost to follow-up was too small to affect the main findings, the question should be  answered yes. |  |  |  |  |
| **Power** |  |  |  |  |
| *27. Did the study have sufficient power to detect a clinically important effect where the probability value for a difference being due to chance is less than 5%?*  Sample sizes have been calculated to detect a difference of x% and y%. |  |  |  |  |
| **Total Score 13/14 = 92.8 %** | | | | |

| **Butler et al. 2015** | | | | |
| --- | --- | --- | --- | --- |
| **Reporting** | **YES** | **NO** | **UTD** | **Notes/Justification** |
| *1. Is the hypothesis/aim/objective of the study clearly described?* | X |  |  | The aim is clearly described. |
| *2. Are the main outcomes to be measured clearly described in the Introduction or Methods section?*  If the main outcomes are first mentioned in the Results section, the question should be answered no. | X |  |  | Clearly described in the Methods section. |
| *3. Are the characteristics of the patients included*  *in the study clearly described?*  In cohort studies and trials, inclusion and/or exclusion criteria should be given. In case‐control studies, a case‐definition and the source for controls should be given. | X |  |  | Clearly described in the Methods section and Table 1. |
| *4. Are the interventions of interest clearly described?*  Treatments and placebo (where relevant) that are to be compared should be clearly described. |  |  |  |  |
| *5. Are the distributions of principal confounders in each group of subjects to be compared clearly described?*  A list of principal confounders is provided. | X |  |  | Reported in the Methods section. |
| *6. Are the main findings of the study clearly described?*  Simple outcome data (including denominators and numerators) should be reported for all major findings so that the reader can check the major analyses and conclusions. (This question does not cover statistical tests  which are considered below). | X |  |  | Reported in the Results section and Table 2. |
| *7. Does the study provide estimates of the random variability in the data for the main outcomes?*  In non-normally distributed data the inter‐quartile range of results should be reported. In normally distributed data the standard error, standard deviation or confidence intervals should be reported. If the distribution of the data is not described, it must be assumed that the estimates used were appropriate and the question should be answered yes. | X |  |  | Standard deviation has been reported. |
| *8. Have all important adverse events that may be a consequence of the intervention been reported?*  This should be answered yes if the study demonstrates that there was a comprehensive attempt to measure adverse events. (A list of possible adverse events is provided). |  |  |  |  |
| *9. Have the characteristics of patients lost to follow‐up been described?*  This should be answered yes where there were no losses to follow‐up or where losses to follow‐up were so small that findings would be unaffected by their inclusion. This should be answered NO where a study does not report the number of patients lost to follow‐up. |  |  |  |  |
| *10. Have actual probability values been reported ( e.g. 0.035 rather than < 0.05) for the main outcomes except where the probability value is less than 0.001?* | X |  |  | Probability values have been reported. |
| **External Validity** | **YES** | **NO** | **UTD** | **Notes/Justification** |
| *11. Were the subjects asked to participate in the study representative of the entire population from which they were recruited?*  The study must identify the source population for patients and describe how the patients were selected. Patients would be representative if they comprised the entire source population, an unselected sample of consecutive patients, or a random sample. Random sampling is only feasible where a list of all members of the relevant population exists. Where a study does not report the proportion of the source population from which the patients are derived, the question should be answered as unable to determine. | X |  |  | Participants were representative. |
| *12. Were those subjects who were prepared to participate representative of the entire population from which they were recruited?*  The proportion of those asked who agreed should be stated. Validation that the sample was representative would include demonstrating that the distribution of the main confounding factors was the same in the study sample and the source population. |  |  | X | It is not known if the distribution of the main confounding factors was the same in the study sample and the source population. |
| *13. Were the staff, places, and facilities where the patients were treated, representative of the treatment the majority of patients receive?*  For the question to be answered yes the study should demonstrate that the intervention was representative of that in use in the source population. The question should be answered no if, for example, the intervention was  undertaken in a specialist centre unrepresentative of the hospitals most of the source population would attend. |  |  |  |  |
| **Internal validity – bias** | **YES** | **NO** | **UTD** | **Notes/Justification** |
| *14. Was an attempt made to blind study subjects to the intervention they have received?*  For studies where the patients would have no way of knowing which intervention they received, this should be answered yes. |  |  |  |  |
| *15. Was an attempt made to blind those measuring the main outcomes of the intervention?* |  |  |  |  |
| *16. If any of the results of the study were based on “data dredging”, was this made clear?*  Any analyses that had not been planned at the outset of the study should be clearly indicated. If no retrospective unplanned subgroup analyses were reported, then answer yes. | X |  |  | No retrospective unplanned subgroup analyses were reported. |
| *17. In trials and cohort studies, do the analyses adjust for different lengths of follow‐up of patients, or in case-control studies, is the time period between the intervention and outcome the same for cases and controls?*  Where follow‐up was the same for all study patients the answer should yes.  If different lengths of follow‐up were adjusted for by, for example, survival analysis the answer should be yes. Studies where differences in follow‐up are ignored should be answered no. |  |  |  |  |
| *18. Were the statistical tests used to assess the main outcomes appropriate?*  The statistical techniques used must be appropriate to the data. For example, Non-parametric methods should be used for small sample sizes. Where little statistical analysis has been undertaken but where there is no evidence of bias, the question should be answered yes. If the distribution of the data (normal or not) is not described it must be assumed that the estimates used were appropriate and the question should be answered yes. | X |  |  | Statistics were appropriate and correctly reported. |
| *19. Was compliance with the intervention/s reliable?*  Where there was non compliance with the allocated treatment or where there was contamination of one group, the question should be answered no.  For studies where the effect of any misclassification was likely to bias any association to the null, the question should be answered yes. |  |  |  |  |
| *20. Were the main outcome measures used accurate (valid and reliable)?*  For studies where the outcome measures are clearly described, the question should be answered yes. For studies which refer to other work or that demonstrates the outcome measures are accurate, the question should be  answered as yes. | X |  |  | Outcome measures clearly described. |
| **Internal validity ‐ confounding (selection bias)** | **YES** | **NO** | **UTD** | **Notes/Justification** |
| *21. Were the patients in different intervention groups (trials and cohort studies) or were the cases and controls (case‐control studies) recruited from the same population?*  For example, patients for all comparison groups should be selected from the same hospital. The question should be answered unable to determine for cohort and case-control studies where there is no information concerning the source of patients included in the study. | X |  |  | Patients were recruited from the same hospital. |
| *22. Were study subjects in different intervention groups (trials and cohort- studies) or were the cases and controls (case‐control studies) recruited over the same period of time?*  For a study which does not specify the time period over which patients were recruited, the question should be answered as unable to determine. |  |  | X | The same time period over which patients were recruited is not known. |
| *23. Were study subjects randomised to intervention groups?*  Studies which state that subjects were randomized should be answered yes except where method of randomisation would not ensure random allocation.  For example, alternate allocation would score no because it is predictable. |  |  |  |  |
| *24. Was the randomised intervention assignment concealed from both patients and health care staff until recruitment was complete and irrevocable?*  All non‐randomised studies should be answered no. If assignment was concealed from patients but not from staff, it should be answered no. |  |  |  |  |
| *25. Was there adequate adjustment for confounding in the analyses from which the main findings were drawn?*  This question should be answered no for trials if: the main conclusions of the study were based on analyses of treatment rather than intention to treat; the distribution of known confounders in the different treatment groups was  not described; or the distribution of known confounders differed between the treatment groups but was not taken into account in the analyses. In non-randomized studies if the effect of the main confounders was not investigated or confounding was demonstrated but no adjustment was made in the final analyses the question should be answered as no. |  |  |  |  |
| *26. Were losses of patients to follow‐up taken into account?*  If the numbers of patients lost to follow‐up are not reported, the question should be answered as unable to determine. If the proportion lost to follow-up was too small to affect the main findings, the question should be  answered yes. |  |  |  |  |
| **Power** |  |  |  |  |
| *27. Did the study have sufficient power to detect a clinically important effect where the probability value for a difference being due to chance is less than 5%?*  Sample sizes have been calculated to detect a difference of x% and y%. |  |  |  |  |
| **Total Score 12/14 = 85,7 %** | | | | |

| **Calò et al. 2009** | | | | |
| --- | --- | --- | --- | --- |
| **Reporting** | **YES** | **NO** | **UTD** | **Notes/Justification** |
| *1. Is the hypothesis/aim/objective of the study clearly described?* | X |  |  | The aim is clearly described. |
| *2. Are the main outcomes to be measured clearly described in the Introduction or Methods section?*  If the main outcomes are first mentioned in the Results section, the question should be answered no. | X |  |  | Outcomes are clearly described in the Methods section. |
| *3. Are the characteristics of the patients included in the study clearly described?*  In cohort studies and trials, inclusion and/or exclusion criteria should be given. In case‐control studies, a case‐definition and the source for controls should be given. | X |  |  | Clearly described in the Methods section. |
| *4. Are the interventions of interest clearly described?*  Treatments and placebo (where relevant) that are to be compared should be clearly described. |  |  |  |  |
| *5. Are the distributions of principal confounders in each group of subjects to be compared clearly described?*  A list of principal confounders is provided. |  | X |  | Confounders have not been clearly described. |
| *6. Are the main findings of the study clearly described?*  Simple outcome data (including denominators and numerators) should be reported for all major findings so that the reader can check the major analyses and conclusions. (This question does not cover statistical tests  which are considered below). |  | X |  | The main findings are not clearly described. There is confusion in the description of tests, data analysis and results. Some of the results are reported in the methods section. |
| *7. Does the study provide estimates of the random variability in the data for the main outcomes?*  In non-normally distributed data the inter‐quartile range of results should be reported. In normally distributed data the standard error, standard deviation or confidence intervals should be reported. If the distribution of the data is not described, it must be assumed that the estimates used were appropriate and the question should be answered yes. | X |  |  | Variability has been reported. |
| *8. Have all important adverse events that may be a consequence of the intervention been reported?*  This should be answered yes if the study demonstrates that there was a comprehensive attempt to measure adverse events. (A list of possible adverse events is provided). |  |  |  |  |
| *9. Have the characteristics of patients lost to follow‐up been described?*  This should be answered yes where there were no losses to follow‐up or where losses to follow‐up were so small that findings would be unaffected by their inclusion. This should be answered NO where a study does not report the number of patients lost to follow‐up. |  |  |  |  |
| *10. Have actual probability values been reported (e.g. 0.035 rather than < 0.05) for the main outcomes except where the probability value is less than 0.001?* |  | X |  | Probability values have not been reported. |
| **External Validity** | **YES** | **NO** | **UTD** | **Notes/Justification** |
| *11. Were the subjects asked to participate in the study representative of the entire population from which they were recruited?*  The study must identify the source population for patients and describe how the patients were selected. Patients would be representative if they comprised the entire source population, an unselected sample of consecutive patients, or a random sample. Random sampling is only feasible where a list of all members of the relevant population exists. Where a study does not report the proportion of the source population from which the patients are derived, the question should be answered as unable to determine. |  |  | X | The source population has not been mentioned. |
| *12. Were those subjects who were prepared to participate representative of the entire population from which they were recruited?*  The proportion of those asked who agreed should be stated. Validation that the sample was representative would include demonstrating that the distribution of the main confounding factors was the same in the study sample and the source population. |  |  | X | The source population has not been mentioned. |
| *13. Were the staff, places, and facilities where the patients were treated, representative of the treatment the majority of patients receive?*  For the question to be answered yes the study should demonstrate that the intervention was representative of that in use in the source population. The question should be answered no if, for example, the intervention was  undertaken in a specialist centre unrepresentative of the hospitals most of the source population would attend. |  |  |  |  |
| **Internal validity – bias** | **YES** | **NO** | **UTD** | **Notes/Justification** |
| *14. Was an attempt made to blind study subjects to the intervention they have received?*  For studies where the patients would have no way of knowing which intervention they received, this should be answered yes. |  |  |  |  |
| *15. Was an attempt made to blind those measuring the main outcomes of the intervention?* |  |  |  |  |
| *16. If any of the results of the study were based on “data dredging”, was this made clear?*  Any analyses that had not been planned at the outset of the study should be clearly indicated. If no retrospective unplanned subgroup analyses were reported, then answer yes. | X |  |  | No retrospective analysis have been reported. |
| *17. In trials and cohort studies, do the analyses adjust for different lengths of follow‐up of patients, or in case-control studies, is the time period between the intervention and outcome the same for cases and controls?*  Where follow‐up was the same for all study patients the answer should yes.  If different lengths of follow‐up were adjusted for by, for example, survival analysis the answer should be yes. Studies where differences in follow‐up are ignored should be answered no. |  |  |  |  |
| *18. Were the statistical tests used to assess the main outcomes appropriate?*  The statistical techniques used must be appropriate to the data. For example, Non-parametric methods should be used for small sample sizes. Where little statistical analysis has been undertaken but where there is no evidence of bias, the question should be answered yes. If the distribution of the data (normal or not) is not described it must be assumed that the estimates used were appropriate and the question should be answered yes. | X |  |  | Statistics are appropriate. |
| *19. Was compliance with the intervention/s reliable?*  Where there was non compliance with the allocated treatment or where there was contamination of one group, the question should be answered no.  For studies where the effect of any misclassification was likely to bias any association to the null, the question should be answered yes. |  |  |  |  |
| *20. Were the main outcome measures used accurate (valid and reliable)?*  For studies where the outcome measures are clearly described, the question should be answered yes. For studies which refer to other work or that demonstrates the outcome measures are accurate, the question should be  answered as yes. |  | X |  | Outcome measures are not clearly described |
| **Internal validity ‐ confounding (selection bias)** | **YES** | **NO** | **UTD** | **Notes/Justification** |
| *21. Were the patients in different intervention groups (trials and cohort studies) or were the cases and controls (case‐control studies) recruited from the same population?*  For example, patients for all comparison groups should be selected from the same hospital. The question should be answered unable to determine for cohort and case-control studies where there is no information concerning the source of patients included in the study. |  |  | X | The source population has not been mentioned. |
| *22. Were study subjects in different intervention groups (trials and cohort- studies) or were the cases and controls (case‐control studies) recruited over the same period of time?*  For a study which does not specify the time period over which patients were recruited, the question should be answered as unable to determine. |  |  | X | Time period in which patients were recruited has not been reported.. |
| *23. Were study subjects randomised to intervention groups?*  Studies which state that subjects were randomized should be answered yes except where method of randomisation would not ensure random allocation.  For example, alternate allocation would score no because it is predictable. |  |  |  |  |
| *24. Was the randomised intervention assignment concealed from both patients and health care staff until recruitment was complete and irrevocable?*  All non‐randomised studies should be answered no. If assignment was concealed from patients but not from staff, it should be answered no. |  |  |  |  |
| *25. Was there adequate adjustment for confounding in the analyses from which the main findings were drawn?*  This question should be answered no for trials if: the main conclusions of the study were based on analyses of treatment rather than intention to treat; the distribution of known confounders in the different treatment groups was  not described; or the distribution of known confounders differed between the treatment groups but was not taken into account in the analyses. In non-randomized studies if the effect of the main confounders was not investigated or confounding was demonstrated but no adjustment was made in the final analyses the question should be answered as no. |  |  |  |  |
| *26. Were losses of patients to follow‐up taken into account?*  If the numbers of patients lost to follow‐up are not reported, the question should be answered as unable to determine. If the proportion lost to follow-up was too small to affect the main findings, the question should be answered yes. |  |  |  |  |
| **Power** | **YES** | **NO** | **UTD** | **Notes/Justification** |
| *27. Did the study have sufficient power to detect a clinically important effect where the probability value for a difference being due to chance is less than 5%?*  Sample sizes have been calculated to detect a difference of x% and y%. |  |  |  |  |
| **Total Score 6/14 = 42.8 %** | | | | |

| **Chang et al. 2015** | | | | |
| --- | --- | --- | --- | --- |
| **Reporting** | **YES** | **NO** | **UTD** | **Notes/Justification** |
| *1. Is the hypothesis/aim/objective of the study clearly described?* | X |  |  | The aim is clearly described. |
| *2. Are the main outcomes to be measured clearly described in the Introduction or Methods section?*  If the main outcomes are first mentioned in the Results section, the question should be answered no. | X |  |  | Clearly described in subjects and methods section (2.3). |
| *3. Are the characteristics of the patients included in the study clearly described?*  In cohort studies and trials, inclusion and/or exclusion criteria should be given. In case‐control studies, a case‐definition and the source for controls should be given. | X |  |  | Clearly described in subjects and method section. |
| *4. Are the interventions of interest clearly described?*  Treatments and placebo (where relevant) that are to be compared should be clearly described. |  |  |  |  |
| *5. Are the distributions of principal confounders in each group of subjects to be compared clearly described?*  A list of principal confounders is provided. |  |  | X |  |
| *6. Are the main findings of the study clearly described?*  Simple outcome data (including denominators and numerators) should be reported for all major findings so that the reader can check the major analyses and conclusions. (This question does not cover statistical tests  which are considered below). | X |  |  | All the findings are reported in table 1 and 2. |
| *7. Does the study provide estimates of the random variability in the data for the main outcomes?*  In non-normally distributed data the inter‐quartile range of results should be reported. In normally distributed data the standard error, standard deviation or confidence intervals should be reported. If the distribution of the data is not described, it must be assumed that the estimates used were appropriate and the question should be answered yes. | X |  |  | Variability has been reported. |
| *8. Have all important adverse events that may be a consequence of the intervention been reported?*  This should be answered yes if the study demonstrates that there was a comprehensive attempt to measure adverse events. (A list of possible adverse events is provided). |  |  |  |  |
| *9. Have the characteristics of patients lost to follow‐up been described?*  This should be answered yes where there were no losses to follow‐up or where losses to follow‐up were so small that findings would be unaffected by their inclusion. This should be answered NO where a study does not report the number of patients lost to follow‐up. |  |  |  |  |
| *10. Have actual probability values been reported (e.g. 0.035 rather than < 0.05) for the main outcomes except where the probability value is less than 0.001?* | X |  |  | Probability values have been reported. |
| **External Validity** | **YES** | **NO** | **UTD** | **Notes/Justification** |
| *11. Were the subjects asked to participate in the study representative of the entire population from which they were recruited?*  The study must identify the source population for patients and describe how the patients were selected. Patients would be representative if they comprised the entire source population, an unselected sample of consecutive patients, or a random sample. Random sampling is only feasible where a list of all members of the relevant population exists. Where a study does not report the proportion of the source population from which the patients are derived, the question should be answered as unable to determine. |  |  | X | The study does not report the proportion of the source population from which the patients are derived. |
| *12. Were those subjects who were prepared to participate representative of the entire population from which they were recruited?*  The proportion of those asked who agreed should be stated. Validation that the sample was representative would include demonstrating that the distribution of the main confounding factors was the same in the study sample and the source population. |  | X |  | No mention to the proportion of patient who agreed to participate to the study is present. |
| *13. Were the staff, places, and facilities where the patients were treated, representative of the treatment the majority of patients receive?*  For the question to be answered yes the study should demonstrate that the intervention was representative of that in use in the source population. The question should be answered no if, for example, the intervention was  undertaken in a specialist centre unrepresentative of the hospitals most of the source population would attend. |  |  |  |  |
| **Internal validity – bias** | **YES** | **NO** | **UTD** | **Notes/Justification** |
| *14. Was an attempt made to blind study subjects to the intervention they have received?*  For studies where the patients would have no way of knowing which intervention they received, this should be answered yes. |  |  |  |  |
| *15. Was an attempt made to blind those measuring the main outcomes of the intervention?* |  |  |  |  |
| *16. If any of the results of the study were based on “data dredging”, was this made clear?*  Any analyses that had not been planned at the outset of the study should be clearly indicated. If no retrospective unplanned subgroup analyses were reported, then answer yes. | X |  |  | No retrospective unplanned analyses were described. |
| *17. In trials and cohort studies, do the analyses adjust for different lengths of follow‐up of patients, or in case-control studies, is the time period between the intervention and outcome the same for cases and controls?*  Where follow‐up was the same for all study patients the answer should yes.  If different lengths of follow‐up were adjusted for by, for example, survival analysis the answer should be yes. Studies where differences in follow‐up are ignored should be answered no. |  |  |  |  |
| *18. Were the statistical tests used to assess the main outcomes appropriate?*  The statistical techniques used must be appropriate to the data. For example, Non-parametric methods should be used for small sample sizes. Where little statistical analysis has been undertaken but where there is no evidence of bias, the question should be answered yes. If the distribution of the data (normal or not) is not described it must be assumed that the estimates used were appropriate and the question should be answered yes. | X |  |  | Statistics were appropriate and correctly reported. |
| *19. Was compliance with the intervention/s reliable?*  Where there was no compliance with the allocated treatment or where there was contamination of one group, the question should be answered no.  For studies where the effect of any misclassification was likely to bias any association to the null, the question should be answered yes. |  |  |  |  |
| *20. Were the main outcome measures used accurate (valid and reliable)?*  For studies where the outcome measures are clearly described, the question should be answered yes. For studies which refer to other work or that demonstrates the outcome measures are accurate, the question should be  answered as yes. | X |  |  | Outcome measures were accurate. |
| **Internal validity ‐ confounding (selection bias)** | **YES** | **NO** | **UTD** | **Notes/Justification** |
| *21. Were the patients in different intervention groups (trials and cohort studies) or were the cases and controls (case‐control studies) recruited from the same population?*  For example, patients for all comparison groups should be selected from the same hospital. The question should be answered unable to determine for cohort and case-control studies where there is no information concerning the source of patients included in the study. | X |  |  | The same 23 patients were tested before and after surgery (at 1 day before surgery and 2 weeks, 6 weeks, 3 months, 6 months, and 1 year after surgery). |
| *22. Were study subjects in different intervention groups (trials and cohort- studies) or were the cases and controls (case‐control studies) recruited over the same period of time?*  For a study which does not specify the time period over which patients were recruited, the question should be answered as unable to determine. |  |  | X | The period of time has not been reported. |
| *23. Were study subjects randomised to intervention groups?*  Studies which state that subjects were randomized should be answered yes except where method of randomisation would not ensure random allocation.  For example, alternate allocation would score no because it is predictable. |  |  |  |  |
| *24. Was the randomised intervention assignment concealed from both patients and health care staff until recruitment was complete and irrevocable?*  All non‐randomised studies should be answered no. If assignment was concealed from patients but not from staff, it should be answered no. |  |  |  |  |
| *25. Was there adequate adjustment for confounding in the analyses from which the main findings were drawn?*  This question should be answered no for trials if: the main conclusions of the study were based on analyses of treatment rather than intention to treat; the distribution of known confounders in the different treatment groups was  not described; or the distribution of known confounders differed between the treatment groups but was not taken into account in the analyses. In non-randomized studies if the effect of the main confounders was not investigated or confounding was demonstrated but no adjustment was made in the final analyses the question should be answered as no. |  |  |  |  |
| *26. Were losses of patients to follow‐up taken into account?*  If the numbers of patients lost to follow‐up are not reported, the question should be answered as unable to determine. If the proportion lost to follow-up was too small to affect the main findings, the question should be answered yes. |  |  |  |  |
| **Power** | **YES** | **NO** | **UTD** | **Notes/Justification** |
| *27. Did the study have sufficient power to detect a clinically important effect where the probability value for a difference being due to chance is less than 5%?*  Sample sizes have been calculated to detect a difference of x% and y%. |  |  |  |  |
| **Total Score 10/14 = 71.4** | | | | |

| **Esbjörnsson et al. 2020** | | | | |
| --- | --- | --- | --- | --- |
| **Reporting** | **YES** | **NO** | **UTD** | **Notes/Justification** |
| *1. Is the hypothesis/aim/objective of the study clearly described?* | X |  |  | The aim is clearly described. |
| *2. Are the main outcomes to be measured clearly described in the Introduction or Methods section?*  If the main outcomes are first mentioned in the Results section, the question should be answered no. | X |  |  | Clearly described in the Methods section. |
| *3. Are the characteristics of the patients included in the study clearly described?*  In cohort studies and trials, inclusion and/or exclusion criteria should be given. In case‐control studies, a case‐definition and the source for controls should be given. | X |  |  | Inclusion and exclusion criteria were clearly described in the Methods section. |
| *4. Are the interventions of interest clearly described?*  Treatments and placebo (where relevant) that are to be compared should be clearly described. |  |  |  |  |
| *5. Are the distributions of principal confounders in each group of subjects to be compared clearly described?*  A list of principal confounders is provided. | X |  |  | A list of the principal confounders is provided |
| *6. Are the main findings of the study clearly described?*  Simple outcome data (including denominators and numerators) should be reported for all major findings so that the reader can check the major analyses and conclusions. (This question does not cover statistical tests  which are considered below). | X |  |  | The main findings of the study are clearly described |
| *7. Does the study provide estimates of the random variability in the data for the main outcomes?*  In non-normally distributed data the inter‐quartile range of results should be reported. In normally distributed data the standard error, standard deviation or confidence intervals should be reported. If the distribution of the data is not described, it must be assumed that the estimates used were appropriate and the question should be answered yes. | X |  |  | The data was normally distributed and standard deviation has been reported |
| *8. Have all important adverse events that may be a consequence of the intervention been reported?*  This should be answered yes if the study demonstrates that there was a comprehensive attempt to measure adverse events. (A list of possible adverse events is provided). |  |  |  |  |
| *9. Have the characteristics of patients lost to follow‐up been described?*  This should be answered yes where there were no losses to follow‐up or where losses to follow‐up were so small that findings would be unaffected by their inclusion. This should be answered NO where a study does not report the number of patients lost to follow‐up. |  |  |  |  |
| *10. Have actual probability values been reported (e.g. 0.035 rather than < 0.05) for the main outcomes except where the probability value is less than 0.001?* | X |  |  | Probability values have been reported. |
| **External Validity** | **YES** | **NO** | **UTD** | **Notes/Justification** |
| *11. Were the subjects asked to participate in the study representative of the entire population from which they were recruited?*  The study must identify the source population for patients and describe how the patients were selected. Patients would be representative if they comprised the entire source population, an unselected sample of consecutive patients, or a random sample. Random sampling is only feasible where a list of all members of the relevant population exists. Where a study does not report the proportion of the source population from which the patients are derived, the question should be answered as unable to determine. |  |  | X | The study does not report the proportion of the source population from which the patients are derived |
| *12. Were those subjects who were prepared to participate representative of the entire population from which they were recruited?*  The proportion of those asked who agreed should be stated. Validation that the sample was representative would include demonstrating that the distribution of the main confounding factors was the same in the study sample and the source population. |  | X |  | The study does not report the proportion of the source population from which the patients are derived. |
| *13. Were the staff, places, and facilities where the patients were treated, representative of the treatment the majority of patients receive?*  For the question to be answered yes the study should demonstrate that the intervention was representative of that in use in the source population. The question should be answered no if, for example, the intervention was  undertaken in a specialist centre unrepresentative of the hospitals most of the source population would attend. |  |  |  |  |
| **Internal validity – bias** | **YES** | **NO** | **UTD** | **Notes/Justification** |
| *14. Was an attempt made to blind study subjects to the intervention they have received?*  For studies where the patients would have no way of knowing which intervention they received, this should be answered yes. |  |  |  |  |
| *15. Was an attempt made to blind those measuring the main outcomes of the intervention?* |  |  |  |  |
| *16. If any of the results of the study were based on “data dredging”, was this made clear?*  Any analyses that had not been planned at the outset of the study should be clearly indicated. If no retrospective unplanned subgroup analyses were reported, then answer yes. | X |  |  | No retrospective unplanned subgroup analyses were reported. |
| *17. In trials and cohort studies, do the analyses adjust for different lengths of follow‐up of patients, or in case-control studies, is the time period between the intervention and outcome the same for cases and controls?*  Where follow‐up was the same for all study patients the answer should yes.  If different lengths of follow‐up were adjusted for by, for example, survival analysis the answer should be yes. Studies where differences in follow‐up are ignored should be answered no. |  |  |  |  |
| *18. Were the statistical tests used to assess the main outcomes appropriate?*  The statistical techniques used must be appropriate to the data. For example, Non-parametric methods should be used for small sample sizes. Where little statistical analysis has been undertaken but where there is no evidence of bias, the question should be answered yes. If the distribution of the data (normal or not) is not described it must be assumed that the estimates used were appropriate and the question should be answered yes. | X |  |  | Statistics were appropriate and correctly reported. |
| *19. Was compliance with the intervention/s reliable?*  Where there was non compliance with the allocated treatment or where there was contamination of one group, the question should be answered no.  For studies where the effect of any misclassification was likely to bias any association to the null, the question should be answered yes. |  |  |  |  |
| *20. Were the main outcome measures used accurate (valid and reliable)?*  For studies where the outcome measures are clearly described, the question should be answered yes. For studies which refer to other work or that demonstrates the outcome measures are accurate, the question should be  answered as yes. | X |  |  | Outcome measures were accurate. |
| **Internal validity ‐ confounding (selection bias)** | **YES** | **NO** | **UTD** | **Notes/Justification** |
| *21. Were the patients in different intervention groups (trials and cohort studies) or were the cases and controls (case‐control studies) recruited from the same population?*  For example, patients for all comparison groups should be selected from the same hospital. The question should be answered unable to determine for cohort and case-control studies where there is no information concerning the source of patients included in the study. | X |  |  | Yes, patients were recruited by the same two orthopaedic department in Stoccolma. |
| *22. Were study subjects in different intervention groups (trials and cohort- studies) or were the cases and controls (case‐control studies) recruited over the same period of time?*  For a study which does not specify the time period over which patients were recruited, the question should be answered as unable to determine. | X |  |  | The patients were recruited during a specified period of time |
| *23. Were study subjects randomised to intervention groups?*  Studies which state that subjects were randomized should be answered yes except where method of randomisation would not ensure random allocation.  For example, alternate allocation would score no because it is predictable. |  |  |  |  |
| *24. Was the randomised intervention assignment concealed from both patients and health care staff until recruitment was complete and irrevocable?*  All non‐randomised studies should be answered no. If assignment was concealed from patients but not from staff, it should be answered no. |  |  |  |  |
| *25. Was there adequate adjustment for confounding in the analyses from which the main findings were drawn?*  This question should be answered no for trials if: the main conclusions of the study were based on analyses of treatment rather than intention to treat; the distribution of known confounders in the different treatment groups was  not described; or the distribution of known confounders differed between the treatment groups but was not taken into account in the analyses. In non-randomized studies if the effect of the main confounders was not investigated or confounding was demonstrated but no adjustment was made in the final analyses the question should be answered as no. |  |  |  |  |
| *26. Were losses of patients to follow‐up taken into account?*  If the numbers of patients lost to follow‐up are not reported, the question should be answered as unable to determine. If the proportion lost to follow-up was too small to affect the main findings, the question should be answered yes. |  |  |  |  |
| **Power** | **YES** | **NO** | **UTD** | **Notes/Justification** |
| *27. Did the study have sufficient power to detect a clinically important effect where the probability value for a difference being due to chance is less than 5%?*  Sample sizes have been calculated to detect a difference of x% and y%. |  |  |  |  |
| **Total Score 12/14 = 85.7 %** | | | | |

| **Esposito et al. 2017** | | | | |
| --- | --- | --- | --- | --- |
| **Reporting** | **YES** | **NO** | **UTD** | **Notes/Justification** |
| *1. Is the hypothesis/aim/objective of the study clearly described?* | X |  |  | The aim is clearly described. |
| *2. Are the main outcomes to be measured clearly described in the Introduction or Methods section?*  If the main outcomes are first mentioned in the Results section, the question should be answered no. | X |  |  | Clearly described in the Methods section. |
| *3. Are the characteristics of the patients included in the study clearly described?*  In cohort studies and trials, inclusion and/or exclusion criteria should be given. In case‐control studies, a case‐definition and the source for controls should be given. | X |  |  | Clearly described in the Methods section and Table 1. |
| *4. Are the interventions of interest clearly described?*  Treatments and placebo (where relevant) that are to be compared should be clearly described. | X |  |  | The interventions are clearly described. |
| *5. Are the distributions of principal confounders in each group of subjects to be compared clearly described?*  A list of principal confounders is provided. |  | X |  | Confounders have not been listed. |
| *6. Are the main findings of the study clearly described?*  Simple outcome data (including denominators and numerators) should be reported for all major findings so that the reader can check the major analyses and conclusions. (This question does not cover statistical tests  which are considered below). | X |  |  | Outcome data have been reported. |
| *7. Does the study provide estimates of the random variability in the data for the main outcomes?*  In non-normally distributed data the inter‐quartile range of results should be reported. In normally distributed data the standard error, standard deviation or confidence intervals should be reported. If the distribution of the data is not described, it must be assumed that the estimates used were appropriate and the question should be answered yes. | X |  |  | Variability has been reported. |
| *8. Have all important adverse events that may be a consequence of the intervention been reported?*  This should be answered yes if the study demonstrates that there was a comprehensive attempt to measure adverse events. (A list of possible adverse events is provided). | X |  |  | Adverse events, such as shoulder overloading, have been considered and assessed. |
| *9. Have the characteristics of patients lost to follow‐up been described?*  This should be answered yes where there were no losses to follow‐up or where losses to follow‐up were so small that findings would be unaffected by their inclusion. This should be answered NO where a study does not report the number of patients lost to follow‐up. |  | X |  | Losses have not been reported. |
| *10. Have actual probability values been reported (e.g. 0.035 rather than < 0.05) for the main outcomes except where the probability value is less than 0.001?* | X |  |  | Probability values have been reported. |
| **External Validity** | **YES** | **NO** | **UTD** | **Notes/Justification** |
| *11. Were the subjects asked to participate in the study representative of the entire population from which they were recruited?*  The study must identify the source population for patients and describe how the patients were selected. Patients would be representative if they comprised the entire source population, an unselected sample of consecutive patients, or a random sample. Random sampling is only feasible where a list of all members of the relevant population exists. Where a study does not report the proportion of the source population from which the patients are derived, the question should be answered as unable to determine. |  |  | X | Source and |
| *12. Were those subjects who were prepared to participate representative of the entire population from which they were recruited?*  The proportion of those asked who agreed should be stated. Validation that the sample was representative would include demonstrating that the distribution of the main confounding factors was the same in the study sample and the source population. |  |  | X | Selection of the patients has not been described. |
| *13. Were the staff, places, and facilities where the patients were treated, representative of the treatment the majority of patients receive?*  For the question to be answered yes the study should demonstrate that the intervention was representative of that in use in the source population. The question should be answered no if, for example, the intervention was  undertaken in a specialist centre unrepresentative of the hospitals most of the source population would attend. |  |  | X | Staff, places and facilities have not been described. |
| **Internal validity – bias** | **YES** | **NO** | **UTD** | **Notes/Justification** |
| *14. Was an attempt made to blind study subjects to the intervention they have received?*  For studies where the patients would have no way of knowing which intervention they received, this should be answered yes. |  |  | X |  |
| *15. Was an attempt made to blind those measuring the main outcomes of the intervention?* |  |  | X | Blinding has not been described. |
| *16. If any of the results of the study were based on “data dredging”, was this made clear?*  Any analyses that had not been planned at the outset of the study should be clearly indicated. If no retrospective unplanned subgroup analyses were reported, then answer yes. | X |  |  | No retrospective unplanned subgroup analyses were reported. |
| *17. In trials and cohort studies, do the analyses adjust for different lengths of follow‐up of patients, or in case-control studies, is the time period between the intervention and outcome the same for cases and controls?*  Where follow‐up was the same for all study patients the answer should yes.  If different lengths of follow‐up were adjusted for by, for example, survival analysis the answer should be yes. Studies where differences in follow‐up are ignored should be answered no. | X |  |  | All patients were tested at the same time points. |
| *18. Were the statistical tests used to assess the main outcomes appropriate?*  The statistical techniques used must be appropriate to the data. For example, Non-parametric methods should be used for small sample sizes. Where little statistical analysis has been undertaken but where there is no evidence of bias, the question should be answered yes. If the distribution of the data (normal or not) is not described it must be assumed that the estimates used were appropriate and the question should be answered yes. | X |  |  | Statistics were appropriate and correctly reported. |
| *19. Was compliance with the intervention/s reliable?*  Where there was non compliance with the allocated treatment or where there was contamination of one group, the question should be answered no.  For studies where the effect of any misclassification was likely to bias any association to the null, the question should be answered yes. |  |  | X | Compliance with the interventions has not been reported. |
| *20. Were the main outcome measures used accurate (valid and reliable)?*  For studies where the outcome measures are clearly described, the question should be answered yes. For studies which refer to other work or that demonstrates the outcome measures are accurate, the question should be  answered as yes. | X |  |  | Outcome measures are accurate. |
| **Internal validity ‐ confounding (selection bias)** | **YES** | **NO** | **UTD** | **Notes/Justification** |
| *21. Were the patients in different intervention groups (trials and cohort studies) or were the cases and controls (case‐control studies) recruited from the same population?*  For example, patients for all comparison groups should be selected from the same hospital. The question should be answered unable to determine for cohort and case-control studies where there is no information concerning the source of patients included in the study. |  |  | X | This information has not been reported. |
| *22. Were study subjects in different intervention groups (trials and cohort- studies) or were the cases and controls (case‐control studies) recruited over the same period of time?*  For a study which does not specify the time period over which patients were recruited, the question should be answered as unable to determine. |  |  | X | Time period has not been specified. |
| *23. Were study subjects randomised to intervention groups?*  Studies which state that subjects were randomized should be answered yes except where method of randomisation would not ensure random allocation.  For example, alternate allocation would score no because it is predictable. | X |  |  | Participants were randomly allocated to one of the groups. |
| *24. Was the randomised intervention assignment concealed from both patients and health care staff until recruitment was complete and irrevocable?*  All non‐randomised studies should be answered no. If assignment was concealed from patients but not from staff, it should be answered no. |  |  | X | This information has not been reported. |
| *25. Was there adequate adjustment for confounding in the analyses from which the main findings were drawn?*  This question should be answered no for trials if: the main conclusions of the study were based on analyses of treatment rather than intention to treat; the distribution of known confounders in the different treatment groups was  not described; or the distribution of known confounders differed between the treatment groups but was not taken into account in the analyses. In non-randomized studies if the effect of the main confounders was not investigated or confounding was demonstrated but no adjustment was made in the final analyses the question should be answered as no. |  | X |  | Confounders have not been listed. |
| *26. Were losses of patients to follow‐up taken into account?*  If the numbers of patients lost to follow‐up are not reported, the question should be answered as unable to determine. If the proportion lost to follow-up was too small to affect the main findings, the question should be answered yes. |  |  | X | Losses in follow-up have not been reported. |
| **Power** | **YES** | **NO** | **UTD** | **Notes/Justification** |
| *27. Did the study have sufficient power to detect a clinically important effect where the probability value for a difference being due to chance is less than 5%?*  Sample sizes have been calculated to detect a difference of x% and y%. |  |  | X | Power analysis has not been reported. |
| **Total Score 13/27 = 48.1 %** | | | | |

| **Eyvazov et al. 2016** | | | | |
| --- | --- | --- | --- | --- |
| **Reporting** | **YES** | **NO** | **UTD** | **Notes/Justification** |
| *1. Is the hypothesis/aim/objective of the study clearly described?* | X |  |  | The aim is clearly described. |
| *2. Are the main outcomes to be measured clearly described in the Introduction or Methods section?*  If the main outcomes are first mentioned in the Results section, the question should be answered no. | X |  |  | Clearly described in the Methods section. |
| *3. Are the characteristics of the patients included in the study clearly described?*  In cohort studies and trials, inclusion and/or exclusion criteria should be given. In case‐control studies, a case‐definition and the source for controls should be given. | X |  |  | Inclusion and exclusion criteria were clearly described in the Methods section. |
| *4. Are the interventions of interest clearly described?*  Treatments and placebo (where relevant) that are to be compared should be clearly described. |  |  |  |  |
| *5. Are the distributions of principal confounders in each group of subjects to be compared clearly described?*  A list of principal confounders is provided. | X |  |  | A list of the principal confounders is provided |
| *6. Are the main findings of the study clearly described?*  Simple outcome data (including denominators and numerators) should be reported for all major findings so that the reader can check the major analyses and conclusions. (This question does not cover statistical tests  which are considered below). | X |  |  | The main findings of the study are clearly described |
| *7. Does the study provide estimates of the random variability in the data for the main outcomes?*  In non-normally distributed data the inter‐quartile range of results should be reported. In normally distributed data the standard error, standard deviation or confidence intervals should be reported. If the distribution of the data is not described, it must be assumed that the estimates used were appropriate and the question should be answered yes. | X |  |  | The data was normally distributed and standard deviation has been reported |
| *8. Have all important adverse events that may be a consequence of the intervention been reported?*  This should be answered yes if the study demonstrates that there was a comprehensive attempt to measure adverse events. (A list of possible adverse events is provided). |  |  |  |  |
| *9. Have the characteristics of patients lost to follow‐up been described?*  This should be answered yes where there were no losses to follow‐up or where losses to follow‐up were so small that findings would be unaffected by their inclusion. This should be answered NO where a study does not report the number of patients lost to follow‐up. |  |  |  |  |
| *10. Have actual probability values been reported (e.g. 0.035 rather than < 0.05) for the main outcomes except where the probability value is less than 0.001?* | X |  |  | Probability values have been reported. |
| **External Validity** | **YES** | **NO** | **UTD** | **Notes/Justification** |
| *11. Were the subjects asked to participate in the study representative of the entire population from which they were recruited?*  The study must identify the source population for patients and describe how the patients were selected. Patients would be representative if they comprised the entire source population, an unselected sample of consecutive patients, or a random sample. Random sampling is only feasible where a list of all members of the relevant population exists. Where a study does not report the proportion of the source population from which the patients are derived, the question should be answered as unable to determine. |  |  | X | The study does not report the proportion of the source population from which the patients are derived |
| *12. Were those subjects who were prepared to participate representative of the entire population from which they were recruited?*  The proportion of those asked who agreed should be stated. Validation that the sample was representative would include demonstrating that the distribution of the main confounding factors was the same in the study sample and the source population. |  | X |  | The study does not report the proportion of the source population from which the patients are derived. |
| *13. Were the staff, places, and facilities where the patients were treated, representative of the treatment the majority of patients receive?*  For the question to be answered yes the study should demonstrate that the intervention was representative of that in use in the source population. The question should be answered no if, for example, the intervention was  undertaken in a specialist centre unrepresentative of the hospitals most of the source population would attend. |  |  |  |  |
| **Internal validity – bias** | **YES** | **NO** | **UTD** | **Notes/Justification** |
| *14. Was an attempt made to blind study subjects to the intervention they have received?*  For studies where the patients would have no way of knowing which intervention they received, this should be answered yes. |  |  |  |  |
| *15. Was an attempt made to blind those measuring the main outcomes of the intervention?* |  |  |  |  |
| *16. If any of the results of the study were based on “data dredging”, was this made clear?*  Any analyses that had not been planned at the outset of the study should be clearly indicated. If no retrospective unplanned subgroup analyses were reported, then answer yes. | X |  |  | No retrospective unplanned subgroup analyses were reported. |
| *17. In trials and cohort studies, do the analyses adjust for different lengths of follow‐up of patients, or in case-control studies, is the time period between the intervention and outcome the same for cases and controls?*  Where follow‐up was the same for all study patients the answer should yes.  If different lengths of follow‐up were adjusted for by, for example, survival analysis the answer should be yes. Studies where differences in follow‐up are ignored should be answered no. |  |  |  |  |
| *18. Were the statistical tests used to assess the main outcomes appropriate?*  The statistical techniques used must be appropriate to the data. For example, Non-parametric methods should be used for small sample sizes. Where little statistical analysis has been undertaken but where there is no evidence of bias, the question should be answered yes. If the distribution of the data (normal or not) is not described it must be assumed that the estimates used were appropriate and the question should be answered yes. | X |  |  | Statistics were appropriate and correctly reported. |
| *19. Was compliance with the intervention/s reliable?*  Where there was non compliance with the allocated treatment or where there was contamination of one group, the question should be answered no.  For studies where the effect of any misclassification was likely to bias any association to the null, the question should be answered yes. |  |  |  |  |
| *20. Were the main outcome measures used accurate (valid and reliable)?*  For studies where the outcome measures are clearly described, the question should be answered yes. For studies which refer to other work or that demonstrates the outcome measures are accurate, the question should be  answered as yes. | X |  |  | Outcome measures were accurate. |
| **Internal validity ‐ confounding (selection bias)** | **YES** | **NO** | **UTD** | **Notes/Justification** |
| *21. Were the patients in different intervention groups (trials and cohort studies) or were the cases and controls (case‐control studies) recruited from the same population?*  For example, patients for all comparison groups should be selected from the same hospital. The question should be answered unable to determine for cohort and case-control studies where there is no information concerning the source of patients included in the study. | X |  |  | Yes, patients were recruited by the same orthopaedic surgery hospital. |
| *22. Were study subjects in different intervention groups (trials and cohort- studies) or were the cases and controls (case‐control studies) recruited over the same period of time?*  For a study which does not specify the time period over which patients were recruited, the question should be answered as unable to determine. | X |  |  | The patients were recruited during a specified period of time |
| *23. Were study subjects randomised to intervention groups?*  Studies which state that subjects were randomized should be answered yes except where method of randomisation would not ensure random allocation.  For example, alternate allocation would score no because it is predictable. |  |  |  |  |
| *24. Was the randomised intervention assignment concealed from both patients and health care staff until recruitment was complete and irrevocable?*  All non‐randomised studies should be answered no. If assignment was concealed from patients but not from staff, it should be answered no. |  |  |  |  |
| *25. Was there adequate adjustment for confounding in the analyses from which the main findings were drawn?*  This question should be answered no for trials if: the main conclusions of the study were based on analyses of treatment rather than intention to treat; the distribution of known confounders in the different treatment groups was  not described; or the distribution of known confounders differed between the treatment groups but was not taken into account in the analyses. In non-randomized studies if the effect of the main confounders was not investigated or confounding was demonstrated but no adjustment was made in the final analyses the question should be answered as no. |  |  |  |  |
| *26. Were losses of patients to follow‐up taken into account?*  If the numbers of patients lost to follow‐up are not reported, the question should be answered as unable to determine. If the proportion lost to follow-up was too small to affect the main findings, the question should be answered yes. |  |  |  |  |
| **Power** | **YES** | **NO** | **UTD** | **Notes/Justification** |
| *27. Did the study have sufficient power to detect a clinically important effect where the probability value for a difference being due to chance is less than 5%?*  Sample sizes have been calculated to detect a difference of x% and y%. |  |  |  |  |
| **Total Score 11/14 = 78.6 %** | | | | |

| **Holnapy et al. 2013** | | | | |
| --- | --- | --- | --- | --- |
| **Reporting** | **YES** | **NO** | **UTD** | **Notes/Justification** |
| *1. Is the hypothesis/aim/objective of the study clearly described?* | X |  |  | The aim is clearly described. |
| *2. Are the main outcomes to be measured clearly described in the Introduction or Methods section?*  If the main outcomes are first mentioned in the Results section, the question should be answered no. | X |  |  | Clearly described in Methods section. |
| *3. Are the characteristics of the patients included in the study clearly described?*  In cohort studies and trials, inclusion and/or exclusion criteria should be given. In case‐control studies, a case‐definition and the source for controls should be given. | X |  |  | Characteristics of the patient are clearly described in the Subjects section. |
| *4. Are the interventions of interest clearly described?*  Treatments and placebo (where relevant) that are to be compared should be clearly described. |  |  |  |  |
| *5. Are the distributions of principal confounders in each group of subjects to be compared clearly described?*  A list of principal confounders is provided. | X |  |  | A list of the principal confounders is provided |
| *6. Are the main findings of the study clearly described?*  Simple outcome data (including denominators and numerators) should be reported for all major findings so that the reader can check the major analyses and conclusions. (This question does not cover statistical tests  which are considered below). | X |  |  | The main findings of the study are clearly described |
| *7. Does the study provide estimates of the random variability in the data for the main outcomes?*  In non-normally distributed data the inter‐quartile range of results should be reported. In normally distributed data the standard error, standard deviation or confidence intervals should be reported. If the distribution of the data is not described, it must be assumed that the estimates used were appropriate and the question should be answered yes. | X |  |  | The data are normally distributed |
| *8. Have all important adverse events that may be a consequence of the intervention been reported?*  This should be answered yes if the study demonstrates that there was a comprehensive attempt to measure adverse events. (A list of possible adverse events is provided). |  |  |  |  |
| *9. Have the characteristics of patients lost to follow‐up been described?*  This should be answered yes where there were no losses to follow‐up or where losses to follow‐up were so small that findings would be unaffected by their inclusion. This should be answered NO where a study does not report the number of patients lost to follow‐up. |  |  |  |  |
| *10. Have actual probability values been reported (e.g. 0.035 rather than < 0.05) for the main outcomes except where the probability value is less than 0.001?* | X |  |  | Probability values have been reported. |
| **External Validity** | **YES** | **NO** | **UTD** | **Notes/Justification** |
| *11. Were the subjects asked to participate in the study representative of the entire population from which they were recruited?*  The study must identify the source population for patients and describe how the patients were selected. Patients would be representative if they comprised the entire source population, an unselected sample of consecutive patients, or a random sample. Random sampling is only feasible where a list of all members of the relevant population exists. Where a study does not report the proportion of the source population from which the patients are derived, the question should be answered as unable to determine. |  |  | X | The study does not report the proportion of the source population from which the patients are derived |
| *12. Were those subjects who were prepared to participate representative of the entire population from which they were recruited?*  The proportion of those asked who agreed should be stated. Validation that the sample was representative would include demonstrating that the distribution of the main confounding factors was the same in the study sample and the source population. |  | X |  | The study does not report the proportion and characteristic of the source population from which the patients are derived. |
| *13. Were the staff, places, and facilities where the patients were treated, representative of the treatment the majority of patients receive?*  For the question to be answered yes the study should demonstrate that the intervention was representative of that in use in the source population. The question should be answered no if, for example, the intervention was  undertaken in a specialist centre unrepresentative of the hospitals most of the source population would attend. |  |  |  |  |
| **Internal validity – bias** | **YES** | **NO** | **UTD** | **Notes/Justification** |
| *14. Was an attempt made to blind study subjects to the intervention they have received?*  For studies where the patients would have no way of knowing which intervention they received, this should be answered yes. |  |  |  |  |
| *15. Was an attempt made to blind those measuring the main outcomes of the intervention?* |  |  |  |  |
| *16. If any of the results of the study were based on “data dredging”, was this made clear?*  Any analyses that had not been planned at the outset of the study should be clearly indicated. If no retrospective unplanned subgroup analyses were reported, then answer yes. | X |  |  | No retrospective unplanned subgroup analyses were reported. |
| *17. In trials and cohort studies, do the analyses adjust for different lengths of follow‐up of patients, or in case-control studies, is the time period between the intervention and outcome the same for cases and controls?*  Where follow‐up was the same for all study patients the answer should yes.  If different lengths of follow‐up were adjusted for by, for example, survival analysis the answer should be yes. Studies where differences in follow‐up are ignored should be answered no. |  |  |  |  |
| *18. Were the statistical tests used to assess the main outcomes appropriate?*  The statistical techniques used must be appropriate to the data. For example, Non-parametric methods should be used for small sample sizes. Where little statistical analysis has been undertaken but where there is no evidence of bias, the question should be answered yes. If the distribution of the data (normal or not) is not described it must be assumed that the estimates used were appropriate and the question should be answered yes. | X |  |  | Statistics were appropriate and correctly reported. |
| *19. Was compliance with the intervention/s reliable?*  Where there was non compliance with the allocated treatment or where there was contamination of one group, the question should be answered no.  For studies where the effect of any misclassification was likely to bias any association to the null, the question should be answered yes. |  |  |  |  |
| *20. Were the main outcome measures used accurate (valid and reliable)?*  For studies where the outcome measures are clearly described, the question should be answered yes. For studies which refer to other work or that demonstrates the outcome measures are accurate, the question should be  answered as yes. | X |  |  | Outcome measures were accurate. |
| **Internal validity ‐ confounding (selection bias)** | **YES** | **NO** | **UTD** | **Notes/Justification** |
| *21. Were the patients in different intervention groups (trials and cohort studies) or were the cases and controls (case‐control studies) recruited from the same population?*  For example, patients for all comparison groups should be selected from the same hospital. The question should be answered unable to determine for cohort and case-control studies where there is no information concerning the source of patients included in the study. | X |  |  | The patients were recruited from the same hospital |
| *22. Were study subjects in different intervention groups (trials and cohort- studies) or were the cases and controls (case‐control studies) recruited over the same period of time?*  For a study which does specify the time period over which patients were recruited, the question should be answered as unable to determine. |  |  | ? | The cohorts included patients who have been underwent to surgery between 1 and five years prior. Thus we could assume that the referring time point is the same for all the cohorts |
| *23. Were study subjects randomised to intervention groups?*  Studies which state that subjects were randomized should be answered yes except where method of randomisation would not ensure random allocation.  For example, alternate allocation would score no because it is predictable. |  |  |  |  |
| *24. Was the randomised intervention assignment concealed from both patients and health care staff until recruitment was complete and irrevocable?*  All non‐randomised studies should be answered no. If assignment was concealed from patients but not from staff, it should be answered no. |  |  |  |  |
| *25. Was there adequate adjustment for confounding in the analyses from which the main findings were drawn?*  This question should be answered no for trials if: the main conclusions of the study were based on analyses of treatment rather than intention to treat; the distribution of known confounders in the different treatment groups was  not described; or the distribution of known confounders differed between the treatment groups but was not taken into account in the analyses. In non-randomized studies if the effect of the main confounders was not investigated or confounding was demonstrated but no adjustment was made in the final analyses the question should be answered as no. |  |  |  |  |
| *26. Were losses of patients to follow‐up taken into account?*  If the numbers of patients lost to follow‐up are not reported, the question should be answered as unable to determine. If the proportion lost to follow-up was too small to affect the main findings, the question should be answered yes. |  |  |  |  |
| **Power** | **YES** | **NO** | **UTD** | **Notes/Justification** |
| *27. Did the study have sufficient power to detect a clinically important effect where the probability value for a difference being due to chance is less than 5%?*  Sample sizes have been calculated to detect a difference of x% and y%. |  |  |  |  |
| **Total Score 11/14 = 78.6 %** | | | | |

| **Hunter et al. 2020 a** | | | | |
| --- | --- | --- | --- | --- |
| **Reporting** | **YES** | **NO** | **UTD** | **Notes/Justification** |
| *1. Is the hypothesis/aim/objective of the study clearly described?* | X |  |  | The aim, hypothesis and objective are clearly described in the Introduction section. |
| *2. Are the main outcomes to be measured clearly described in the Introduction or Methods section?*  If the main outcomes are first mentioned in the Results section, the question should be answered no. | X |  |  | Clearly described in the Methods section. |
| *3. Are the characteristics of the patients included*  *in the study clearly described?*  In cohort studies and trials, inclusion and/or exclusion criteria should be given. In case‐control studies, a case‐definition and the source for controls should be given. | X |  |  | Clearly described in the Methods section. |
| *4. Are the interventions of interest clearly described?*  Treatments and placebo (where relevant) that are to be compared should be clearly described. |  |  |  |  |
| *5. Are the distributions of principal confounders in each group of subjects to be compared clearly described?*  A list of principal confounders is provided. | X |  |  | Confounders are described in the discussion section. |
| *6. Are the main findings of the study clearly described?*  Simple outcome data (including denominators and numerators) should be reported for all major findings so that the reader can check the major analyses and conclusions. (This question does not cover statistical tests  which are considered below). | X |  |  | Reported in the Results section and Tabls 1 and 2. |
| *7. Does the study provide estimates of the random variability in the data for the main outcomes?*  In non-normally distributed data the inter‐quartile range of results should be reported. In normally distributed data the standard error, standard deviation or confidence intervals should be reported. If the distribution of the data is not described, it must be assumed that the estimates used were appropriate and the question should be answered yes. | X |  |  | Standard deviation has been reported. |
| *8. Have all important adverse events that may be a consequence of the intervention been reported?*  This should be answered yes if the study demonstrates that there was a comprehensive attempt to measure adverse events. (A list of possible adverse events is provided). |  |  |  |  |
| *9. Have the characteristics of patients lost to follow‐up been described?*  This should be answered yes where there were no losses to follow‐up or where losses to follow‐up were so small that findings would be unaffected by their inclusion. This should be answered NO where a study does not report the number of patients lost to follow‐up. |  |  |  |  |
| *10. Have actual probability values been reported ( e.g. 0.035 rather than < 0.05) for the main outcomes except where the probability value is less than 0.001?* | X |  |  | Probability values have been reported. |
| **External Validity** | **YES** | **NO** | **UTD** | **Notes/Justification** |
| *11. Were the subjects asked to participate in the study representative of the entire population from which they were recruited?*  The study must identify the source population for patients and describe how the patients were selected. Patients would be representative if they comprised the entire source population, an unselected sample of consecutive patients, or a random sample. Random sampling is only feasible where a list of all members of the relevant population exists. Where a study does not report the proportion of the source population from which the patients are derived, the question should be answered as unable to determine. | X |  |  | Participants were representative. |
| *12. Were those subjects who were prepared to participate representative of the entire population from which they were recruited?*  The proportion of those asked who agreed should be stated. Validation that the sample was representative would include demonstrating that the distribution of the main confounding factors was the same in the study sample and the source population. |  |  | X | It is not known if the distribution of the main confounding factors was the same in the study sample and the source population. |
| *13. Were the staff, places, and facilities where the patients were treated, representative of the treatment the majority of patients receive?*  For the question to be answered yes the study should demonstrate that the intervention was representative of that in use in the source population. The question should be answered no if, for example, the intervention was  undertaken in a specialist centre unrepresentative of the hospitals most of the source population would attend. |  |  |  |  |
| **Internal validity – bias** | **YES** | **NO** | **UTD** | **Notes/Justification** |
| *14. Was an attempt made to blind study subjects to the intervention they have received?*  For studies where the patients would have no way of knowing which intervention they received, this should be answered yes. |  |  |  |  |
| *15. Was an attempt made to blind those measuring the main outcomes of the intervention?* |  |  |  |  |
| *16. If any of the results of the study were based on “data dredging”, was this made clear?*  Any analyses that had not been planned at the outset of the study should be clearly indicated. If no retrospective unplanned subgroup analyses were reported, then answer yes. | X |  |  | No retrospective unplanned subgroup analyses were reported. |
| *17. In trials and cohort studies, do the analyses adjust for different lengths of follow‐up of patients, or in case-control studies, is the time period between the intervention and outcome the same for cases and controls?*  Where follow‐up was the same for all study patients the answer should yes.  If different lengths of follow‐up were adjusted for by, for example, survival analysis the answer should be yes. Studies where differences in follow‐up are ignored should be answered no. |  |  |  |  |
| *18. Were the statistical tests used to assess the main outcomes appropriate?*  The statistical techniques used must be appropriate to the data. For example, Non-parametric methods should be used for small sample sizes. Where little statistical analysis has been undertaken but where there is no evidence of bias, the question should be answered yes. If the distribution of the data (normal or not) is not described it must be assumed that the estimates used were appropriate and the question should be answered yes. | X |  |  | Statistics were appropriate and correctly reported. |
| *19. Was compliance with the intervention/s reliable?*  Where there was non compliance with the allocated treatment or where there was contamination of one group, the question should be answered no.  For studies where the effect of any misclassification was likely to bias any association to the null, the question should be answered yes. |  |  |  |  |
| *20. Were the main outcome measures used accurate (valid and reliable)?*  For studies where the outcome measures are clearly described, the question should be answered yes. For studies which refer to other work or that demonstrates the outcome measures are accurate, the question should be  answered as yes. | X |  |  | Outcome measures clearly described. |
| **Internal validity ‐ confounding (selection bias)** | **YES** | **NO** | **UTD** | **Notes/Justification** |
| *21. Were the patients in different intervention groups (trials and cohort studies) or were the cases and controls (case‐control studies) recruited from the same population?*  For example, patients for all comparison groups should be selected from the same hospital. The question should be answered unable to determine for cohort and case-control studies where there is no information concerning the source of patients included in the study. | X |  |  | Patients were recruited from the same centre. |
| *22. Were study subjects in different intervention groups (trials and cohort- studies) or were the cases and controls (case‐control studies) recruited over the same period of time?*  For a study which does not specify the time period over which patients were recruited, the question should be answered as unable to determine. |  |  | X | The time period over which patients were recruited is not specified. |
| *23. Were study subjects randomised to intervention groups?*  Studies which state that subjects were randomized should be answered yes except where method of randomisation would not ensure random allocation.  For example, alternate allocation would score no because it is predictable. |  |  |  |  |
| *24. Was the randomised intervention assignment concealed from both patients and health care staff until recruitment was complete and irrevocable?*  All non‐randomised studies should be answered no. If assignment was concealed from patients but not from staff, it should be answered no. |  |  |  |  |
| *25. Was there adequate adjustment for confounding in the analyses from which the main findings were drawn?*  This question should be answered no for trials if: the main conclusions of the study were based on analyses of treatment rather than intention to treat; the distribution of known confounders in the different treatment groups was  not described; or the distribution of known confounders differed between the treatment groups but was not taken into account in the analyses. In non-randomized studies if the effect of the main confounders was not investigated or confounding was demonstrated but no adjustment was made in the final analyses the question should be answered as no. |  |  |  |  |
| *26. Were losses of patients to follow‐up taken into account?*  If the numbers of patients lost to follow‐up are not reported, the question should be answered as unable to determine. If the proportion lost to follow-up was too small to affect the main findings, the question should be  answered yes. |  |  |  |  |
| **Power** |  |  |  |  |
| *27. Did the study have sufficient power to detect a clinically important effect where the probability value for a difference being due to chance is less than 5%?*  Sample sizes have been calculated to detect a difference of x% and y%. |  |  |  |  |
| **Total Score 12/14 = 85,7 %** | | | | |

| **Hunter et al. 2020 b** | | | | |
| --- | --- | --- | --- | --- |
| **Reporting** | **YES** | **NO** | **UTD** | **Notes/Justification** |
| *1. Is the hypothesis/aim/objective of the study clearly described?* | X |  |  | The aim is clearly described. |
| *2. Are the main outcomes to be measured clearly described in the Introduction or Methods section?*  If the main outcomes are first mentioned in the Results section, the question should be answered no. | X |  |  | Clearly described in the Methods section. |
| *3. Are the characteristics of the patients included in the study clearly described?*  In cohort studies and trials, inclusion and/or exclusion criteria should be given. In case‐control studies, a case‐definition and the source for controls should be given. | X |  |  | Clearly described in the Methods section (“participants”). |
| *4. Are the interventions of interest clearly described?*  Treatments and placebo (where relevant) that are to be compared should be clearly described. |  |  |  |  |
| *5. Are the distributions of principal confounders in each group of subjects to be compared clearly described?*  A list of principal confounders is provided. | X |  |  | Confounders have been described in the Statistical Analysis section. |
| *6. Are the main findings of the study clearly described?*  Simple outcome data (including denominators and numerators) should be reported for all major findings so that the reader can check the major analyses and conclusions. (This question does not cover statistical tests  which are considered below). | X |  |  |  |
| *7. Does the study provide estimates of the random variability in the data for the main outcomes?*  In non-normally distributed data the inter‐quartile range of results should be reported. In normally distributed data the standard error, standard deviation or confidence intervals should be reported. If the distribution of the data is not described, it must be assumed that the estimates used were appropriate and the question should be answered yes. | X |  |  | Variability has been reported. |
| *8. Have all important adverse events that may be a consequence of the intervention been reported?*  This should be answered yes if the study demonstrates that there was a comprehensive attempt to measure adverse events. (A list of possible adverse events is provided). |  |  |  |  |
| *9. Have the characteristics of patients lost to follow‐up been described?*  This should be answered yes where there were no losses to follow‐up or where losses to follow‐up were so small that findings would be unaffected by their inclusion. This should be answered NO where a study does not report the number of patients lost to follow‐up. |  |  |  |  |
| *10. Have actual probability values been reported (e.g. 0.035 rather than < 0.05) for the main outcomes except where the probability value is less than 0.001?* | X |  |  | Probability values have been reported. |
| **External Validity** | **YES** | **NO** | **UTD** | **Notes/Justification** |
| *11. Were the subjects asked to participate in the study representative of the entire population from which they were recruited?*  The study must identify the source population for patients and describe how the patients were selected. Patients would be representative if they comprised the entire source population, an unselected sample of consecutive patients, or a random sample. Random sampling is only feasible where a list of all members of the relevant population exists. Where a study does not report the proportion of the source population from which the patients are derived, the question should be answered as unable to determine. |  |  | X |  |
| *12. Were those subjects who were prepared to participate representative of the entire population from which they were recruited?*  The proportion of those asked who agreed should be stated. Validation that the sample was representative would include demonstrating that the distribution of the main confounding factors was the same in the study sample and the source population. |  |  | X |  |
| *13. Were the staff, places, and facilities where the patients were treated, representative of the treatment the majority of patients receive?*  For the question to be answered yes the study should demonstrate that the intervention was representative of that in use in the source population. The question should be answered no if, for example, the intervention was  undertaken in a specialist centre unrepresentative of the hospitals most of the source population would attend. |  |  |  |  |
| **Internal validity – bias** | **YES** | **NO** | **UTD** | **Notes/Justification** |
| *14. Was an attempt made to blind study subjects to the intervention they have received?*  For studies where the patients would have no way of knowing which intervention they received, this should be answered yes. |  |  |  |  |
| *15. Was an attempt made to blind those measuring the main outcomes of the intervention?* |  |  |  |  |
| *16. If any of the results of the study were based on “data dredging”, was this made clear?*  Any analyses that had not been planned at the outset of the study should be clearly indicated. If no retrospective unplanned subgroup analyses were reported, then answer yes. | X |  |  | No dredging data is mentioned. |
| *17. In trials and cohort studies, do the analyses adjust for different lengths of follow‐up of patients, or in case-control studies, is the time period between the intervention and outcome the same for cases and controls?*  Where follow‐up was the same for all study patients the answer should yes.  If different lengths of follow‐up were adjusted for by, for example, survival analysis the answer should be yes. Studies where differences in follow‐up are ignored should be answered no. |  |  |  |  |
| *18. Were the statistical tests used to assess the main outcomes appropriate?*  The statistical techniques used must be appropriate to the data. For example, Non-parametric methods should be used for small sample sizes. Where little statistical analysis has been undertaken but where there is no evidence of bias, the question should be answered yes. If the distribution of the data (normal or not) is not described it must be assumed that the estimates used were appropriate and the question should be answered yes. | X |  |  | Statistics were appropriate and correctly reported. |
| *19. Was compliance with the intervention/s reliable?*  Where there was no compliance with the allocated treatment or where there was contamination of one group, the question should be answered no.  For studies where the effect of any misclassification was likely to bias any association to the null, the question should be answered yes. |  |  |  |  |
| *20. Were the main outcome measures used accurate (valid and reliable)?*  For studies where the outcome measures are clearly described, the question should be answered yes. For studies which refer to other work or that demonstrates the outcome measures are accurate, the question should be  answered as yes. | X |  |  | Outcome measures were accurate. |
| **Internal validity ‐ confounding (selection bias)** | **YES** | **NO** | **UTD** | **Notes/Justification** |
| *21. Were the patients in different intervention groups (trials and cohort studies) or were the cases and controls (case‐control studies) recruited from the same population?*  For example, patients for all comparison groups should be selected from the same hospital. The question should be answered unable to determine for cohort and case-control studies where there is no information concerning the source of patients included in the study. |  |  | X |  |
| *22. Were study subjects in different intervention groups (trials and cohort- studies) or were the cases and controls (case‐control studies) recruited over the same period of time?*  For a study which does not specify the time period over which patients were recruited, the question should be answered as unable to determine. | X |  |  | Patients were recruited over the same time period. |
| *23. Were study subjects randomised to intervention groups?*  Studies which state that subjects were randomized should be answered yes except where method of randomisation would not ensure random allocation.  For example, alternate allocation would score no because it is predictable. |  |  |  |  |
| *24. Was the randomised intervention assignment concealed from both patients and health care staff until recruitment was complete and irrevocable?*  All non‐randomised studies should be answered no. If assignment was concealed from patients but not from staff, it should be answered no. |  |  |  |  |
| *25. Was there adequate adjustment for confounding in the analyses from which the main findings were drawn?*  This question should be answered no for trials if: the main conclusions of the study were based on analyses of treatment rather than intention to treat; the distribution of known confounders in the different treatment groups was  not described; or the distribution of known confounders differed between the treatment groups but was not taken into account in the analyses. In non-randomized studies if the effect of the main confounders was not investigated or confounding was demonstrated but no adjustment was made in the final analyses the question should be answered as no. |  |  |  |  |
| *26. Were losses of patients to follow‐up taken into account?*  If the numbers of patients lost to follow‐up are not reported, the question should be answered as unable to determine. If the proportion lost to follow-up was too small to affect the main findings, the question should be answered yes. |  |  |  |  |
| **Power** | **YES** | **NO** | **UTD** | **Notes/Justification** |
| *27. Did the study have sufficient power to detect a clinically important effect where the probability value for a difference being due to chance is less than 5%?*  Sample sizes have been calculated to detect a difference of x% and y%. |  |  |  |  |
| **Total Score 11/14 = 78.5** | | | | |

| **Jo et al. 2016** | | | | |
| --- | --- | --- | --- | --- |
| **Reporting** | **YES** | **NO** | **UTD** | **Notes/Justification** |
| *1. Is the hypothesis/aim/objective of the study clearly described?* | X |  |  | The aim, objective  And hypothesis are clearly described in the abstract section. |
| *2. Are the main outcomes to be measured clearly described in the Introduction or Methods section?*  If the main outcomes are first mentioned in the Results section, the question should be answered no. | X |  |  | Clearly described in the methods section. |
| *3. Are the characteristics of the patients included*  *in the study clearly described?*  In cohort studies and trials, inclusion and/or exclusion criteria should be given. In case‐control studies, a case‐definition and the source for controls should be given. | X |  |  | Clearly described in the Methods section. |
| *4. Are the interventions of interest clearly described?*  Treatments and placebo (where relevant) that are to be compared should be clearly described. |  |  |  |  |
| *5. Are the distributions of principal confounders in each group of subjects to be compared clearly described?*  A list of principal confounders is provided. | X |  |  | Confounders were reported. |
| *6. Are the main findings of the study clearly described?*  Simple outcome data (including denominators and numerators) should be reported for all major findings so that the reader can check the major analyses and conclusions. (This question does not cover statistical tests  which are considered below). | X |  |  | Clearly reported in the Results section and table 1 and 2. |
| *7. Does the study provide estimates of the random variability in the data for the main outcomes?*  In non-normally distributed data the inter‐quartile range of results should be reported. In normally distributed data the standard error, standard deviation or confidence intervals should be reported. If the distribution of the data is not described, it must be assumed that the estimates used were appropriate and the question should be answered yes. | X |  |  | Standard deviation has been reported. |
| *8. Have all important adverse events that may be a consequence of the intervention been reported?*  This should be answered yes if the study demonstrates that there was a comprehensive attempt to measure adverse events. (A list of possible adverse events is provided). |  |  |  |  |
| *9. Have the characteristics of patients lost to follow‐up been described?*  This should be answered yes where there were no losses to follow‐up or where losses to follow‐up were so small that findings would be unaffected by their inclusion. This should be answered NO where a study does not report the number of patients lost to follow‐up. |  |  |  |  |
| *10. Have actual probability values been reported ( e.g. 0.035 rather than < 0.05) for the main outcomes except where the probability value is less than 0.001?* | X |  |  | Probability values have been reported. |
| **External Validity** | **YES** | **NO** | **UTD** | **Notes/Justification** |
| *11. Were the subjects asked to participate in the study representative of the entire population from which they were recruited?*  The study must identify the source population for patients and describe how the patients were selected. Patients would be representative if they comprised the entire source population, an unselected sample of consecutive patients, or a random sample. Random sampling is only feasible where a list of all members of the relevant population exists. Where a study does not report the proportion of the source population from which the patients are derived, the question should be answered as unable to determine. | X |  |  | Participants were representative. |
| *12. Were those subjects who were prepared to participate representative of the entire population from which they were recruited?*  The proportion of those asked who agreed should be stated. Validation that the sample was representative would include demonstrating that the distribution of the main confounding factors was the same in the study sample and the source population. | X |  |  | The population is representative. |
| *13. Were the staff, places, and facilities where the patients were treated, representative of the treatment the majority of patients receive?*  For the question to be answered yes the study should demonstrate that the intervention was representative of that in use in the source population. The question should be answered no if, for example, the intervention was  undertaken in a specialist centre unrepresentative of the hospitals most of the source population would attend. |  |  |  |  |
| **Internal validity – bias** | **YES** | **NO** | **UTD** | **Notes/Justification** |
| *14. Was an attempt made to blind study subjects to the intervention they have received?*  For studies where the patients would have no way of knowing which intervention they received, this should be answered yes. |  |  |  |  |
| *15. Was an attempt made to blind those measuring the main outcomes of the intervention?* |  |  |  |  |
| *16. If any of the results of the study were based on “data dredging”, was this made clear?*  Any analyses that had not been planned at the outset of the study should be clearly indicated. If no retrospective unplanned subgroup analyses were reported, then answer yes. | X |  |  | No retrospective unplanned subgroup analyses were reported. |
| *17. In trials and cohort studies, do the analyses adjust for different lengths of follow‐up of patients, or in case-control studies, is the time period between the intervention and outcome the same for cases and controls?*  Where follow‐up was the same for all study patients the answer should yes.  If different lengths of follow‐up were adjusted for by, for example, survival analysis the answer should be yes. Studies where differences in follow‐up are ignored should be answered no. |  |  |  |  |
| *18. Were the statistical tests used to assess the main outcomes appropriate?*  The statistical techniques used must be appropriate to the data. For example, Non-parametric methods should be used for small sample sizes. Where little statistical analysis has been undertaken but where there is no evidence of bias, the question should be answered yes. If the distribution of the data (normal or not) is not described it must be assumed that the estimates used were appropriate and the question should be answered yes. | X |  |  | Statistics were appropriate and correctly reported. |
| *19. Was compliance with the intervention/s reliable?*  Where there was non compliance with the allocated treatment or where there was contamination of one group, the question should be answered no.  For studies where the effect of any misclassification was likely to bias any association to the null, the question should be answered yes. |  |  |  |  |
| *20. Were the main outcome measures used accurate (valid and reliable)?*  For studies where the outcome measures are clearly described, the question should be answered yes. For studies which refer to other work or that demonstrates the outcome measures are accurate, the question should be  answered as yes. | X |  |  | Outcome measures clearly described. |
| **Internal validity ‐ confounding (selection bias)** | **YES** | **NO** | **UTD** | **Notes/Justification** |
| *21. Were the patients in different intervention groups (trials and cohort studies) or were the cases and controls (case‐control studies) recruited from the same population?*  For example, patients for all comparison groups should be selected from the same hospital. The question should be answered unable to determine for cohort and case-control studies where there is no information concerning the source of patients included in the study. | X |  |  | The patients were recruited in the same hospital. |
| *22. Were study subjects in different intervention groups (trials and cohort- studies) or were the cases and controls (case‐control studies) recruited over the same period of time?*  For a study which does not specify the time period over which patients were recruited, the question should be answered as unable to determine. | X |  |  | Patients were recruited over the same time period. |
| *23. Were study subjects randomised to intervention groups?*  Studies which state that subjects were randomized should be answered yes except where method of randomisation would not ensure random allocation.  For example, alternate allocation would score no because it is predictable. |  |  |  |  |
| *24. Was the randomised intervention assignment concealed from both patients and health care staff until recruitment was complete and irrevocable?*  All non‐randomised studies should be answered no. If assignment was concealed from patients but not from staff, it should be answered no. |  |  |  |  |
| *25. Was there adequate adjustment for confounding in the analyses from which the main findings were drawn?*  This question should be answered no for trials if: the main conclusions of the study were based on analyses of treatment rather than intention to treat; the distribution of known confounders in the different treatment groups was  not described; or the distribution of known confounders differed between the treatment groups but was not taken into account in the analyses. In non-randomized studies if the effect of the main confounders was not investigated or confounding was demonstrated but no adjustment was made in the final analyses the question should be answered as no. |  |  |  |  |
| *26. Were losses of patients to follow‐up taken into account?*  If the numbers of patients lost to follow‐up are not reported, the question should be answered as unable to determine. If the proportion lost to follow-up was too small to affect the main findings, the question should be  answered yes. |  |  |  |  |
| **Power** |  |  |  |  |
| *27. Did the study have sufficient power to detect a clinically important effect where the probability value for a difference being due to chance is less than 5%?*  Sample sizes have been calculated to detect a difference of x% and y%. |  |  |  |  |
| **Total Score 14/14 = 100 %** | | | | |

| **Jogi et al. 2011** | | | | |
| --- | --- | --- | --- | --- |
| **Reporting** | **YES** | **NO** | **UTD** | **Notes/Justification** |
| *1. Is the hypothesis/aim/objective of the study clearly described?* | X |  |  | The objective is clearly described in the introduction section. |
| *2. Are the main outcomes to be measured clearly described in the Introduction or Methods section?*  If the main outcomes are first mentioned in the Results section, the question should be answered no. | X |  |  | Clearly described in the introduction section. |
| *3. Are the characteristics of the patients included*  *in the study clearly described?*  In cohort studies and trials, inclusion and/or exclusion criteria should be given. In case‐control studies, a case‐definition and the source for controls should be given. | X |  |  | Clearly described in the Methods section. |
| *4. Are the interventions of interest clearly described?*  Treatments and placebo (where relevant) that are to be compared should be clearly described. |  |  |  |  |
| *5. Are the distributions of principal confounders in each group of subjects to be compared clearly described?*  A list of principal confounders is provided. | X |  |  | Reported in the Methods section. |
| *6. Are the main findings of the study clearly described?*  Simple outcome data (including denominators and numerators) should be reported for all major findings so that the reader can check the major analyses and conclusions. (This question does not cover statistical tests  which are considered below). | X |  |  | Reported in the Results section and Tabls. |
| *7. Does the study provide estimates of the random variability in the data for the main outcomes?*  In non-normally distributed data the inter‐quartile range of results should be reported. In normally distributed data the standard error, standard deviation or confidence intervals should be reported. If the distribution of the data is not described, it must be assumed that the estimates used were appropriate and the question should be answered yes. | X |  |  | Standard deviation has been reported. |
| *8. Have all important adverse events that may be a consequence of the intervention been reported?*  This should be answered yes if the study demonstrates that there was a comprehensive attempt to measure adverse events. (A list of possible adverse events is provided). |  |  |  |  |
| *9. Have the characteristics of patients lost to follow‐up been described?*  This should be answered yes where there were no losses to follow‐up or where losses to follow‐up were so small that findings would be unaffected by their inclusion. This should be answered NO where a study does not report the number of patients lost to follow‐up. |  |  |  |  |
| *10. Have actual probability values been reported ( e.g. 0.035 rather than < 0.05) for the main outcomes except where the probability value is less than 0.001?* | X |  |  | Probability values have been reported. |
| **External Validity** | **YES** | **NO** | **UTD** | **Notes/Justification** |
| *11. Were the subjects asked to participate in the study representative of the entire population from which they were recruited?*  The study must identify the source population for patients and describe how the patients were selected. Patients would be representative if they comprised the entire source population, an unselected sample of consecutive patients, or a random sample. Random sampling is only feasible where a list of all members of the relevant population exists. Where a study does not report the proportion of the source population from which the patients are derived, the question should be answered as unable to determine. |  |  | X | The authors do not report clearly the proportion of the source population from which the patients wereselected. |
| *12. Were those subjects who were prepared to participate representative of the entire population from which they were recruited?*  The proportion of those asked who agreed should be stated. Validation that the sample was representative would include demonstrating that the distribution of the main confounding factors was the same in the study sample and the source population. |  |  | X | It is not known if the distribution of the main confounding factors was the same in the study sample and the source population. |
| *13. Were the staff, places, and facilities where the patients were treated, representative of the treatment the majority of patients receive?*  For the question to be answered yes the study should demonstrate that the intervention was representative of that in use in the source population. The question should be answered no if, for example, the intervention was  undertaken in a specialist centre unrepresentative of the hospitals most of the source population would attend. |  |  |  |  |
| **Internal validity – bias** | **YES** | **NO** | **UTD** | **Notes/Justification** |
| *14. Was an attempt made to blind study subjects to the intervention they have received?*  For studies where the patients would have no way of knowing which intervention they received, this should be answered yes. |  |  |  |  |
| *15. Was an attempt made to blind those measuring the main outcomes of the intervention?* |  |  |  |  |
| *16. If any of the results of the study were based on “data dredging”, was this made clear?*  Any analyses that had not been planned at the outset of the study should be clearly indicated. If no retrospective unplanned subgroup analyses were reported, then answer yes. | X |  |  | No retrospective unplanned subgroup analyses were reported. |
| *17. In trials and cohort studies, do the analyses adjust for different lengths of follow‐up of patients, or in case-control studies, is the time period between the intervention and outcome the same for cases and controls?*  Where follow‐up was the same for all study patients the answer should yes.  If different lengths of follow‐up were adjusted for by, for example, survival analysis the answer should be yes. Studies where differences in follow‐up are ignored should be answered no. |  |  |  |  |
| *18. Were the statistical tests used to assess the main outcomes appropriate?*  The statistical techniques used must be appropriate to the data. For example, Non-parametric methods should be used for small sample sizes. Where little statistical analysis has been undertaken but where there is no evidence of bias, the question should be answered yes. If the distribution of the data (normal or not) is not described it must be assumed that the estimates used were appropriate and the question should be answered yes. | X |  |  | Statistics were appropriate and correctly reported. |
| *19. Was compliance with the intervention/s reliable?*  Where there was non compliance with the allocated treatment or where there was contamination of one group, the question should be answered no.  For studies where the effect of any misclassification was likely to bias any association to the null, the question should be answered yes. |  |  |  |  |
| *20. Were the main outcome measures used accurate (valid and reliable)?*  For studies where the outcome measures are clearly described, the question should be answered yes. For studies which refer to other work or that demonstrates the outcome measures are accurate, the question should be  answered as yes. | X |  |  | Outcome measures clearly described. |
| **Internal validity ‐ confounding (selection bias)** | **YES** | **NO** | **UTD** | **Notes/Justification** |
| *21. Were the patients in different intervention groups (trials and cohort studies) or were the cases and controls (case‐control studies) recruited from the same population?*  For example, patients for all comparison groups should be selected from the same hospital. The question should be answered unable to determine for cohort and case-control studies where there is no information concerning the source of patients included in the study. |  |  | X | The authors do not provide any information concerning the source of patients included in the study. |
| *22. Were study subjects in different intervention groups (trials and cohort- studies) or were the cases and controls (case‐control studies) recruited over the same period of time?*  For a study which does not specify the time period over which patients were recruited, the question should be answered as unable to determine. |  |  | X | the time period over which patients were recruited is not specified. |
| *23. Were study subjects randomised to intervention groups?*  Studies which state that subjects were randomized should be answered yes except where method of randomisation would not ensure random allocation.  For example, alternate allocation would score no because it is predictable. |  |  |  |  |
| *24. Was the randomised intervention assignment concealed from both patients and health care staff until recruitment was complete and irrevocable?*  All non‐randomised studies should be answered no. If assignment was concealed from patients but not from staff, it should be answered no. |  |  |  |  |
| *25. Was there adequate adjustment for confounding in the analyses from which the main findings were drawn?*  This question should be answered no for trials if: the main conclusions of the study were based on analyses of treatment rather than intention to treat; the distribution of known confounders in the different treatment groups was  not described; or the distribution of known confounders differed between the treatment groups but was not taken into account in the analyses. In non-randomized studies if the effect of the main confounders was not investigated or confounding was demonstrated but no adjustment was made in the final analyses the question should be answered as no. |  |  |  |  |
| *26. Were losses of patients to follow‐up taken into account?*  If the numbers of patients lost to follow‐up are not reported, the question should be answered as unable to determine. If the proportion lost to follow-up was too small to affect the main findings, the question should be  answered yes. |  |  |  |  |
| **Power** |  |  |  |  |
| *27. Did the study have sufficient power to detect a clinically important effect where the probability value for a difference being due to chance is less than 5%?*  Sample sizes have been calculated to detect a difference of x% and y%. |  |  |  |  |
| **Total Score 10/14 = 71,4 %** | | | | |

| **Jogi et al. 2015** | | | | |
| --- | --- | --- | --- | --- |
| **Reporting** | **YES** | **NO** | **UTD** | **Notes/Justification** |
| *1. Is the hypothesis/aim/objective of the study clearly described?* | X |  |  | The aim is clearly described. |
| *2. Are the main outcomes to be measured clearly described in the Introduction or Methods section?*  If the main outcomes are first mentioned in the Results section, the question should be answered no. | X |  |  | Clearly described in the Methods section. |
| *3. Are the characteristics of the patients included in the study clearly described?*  In cohort studies and trials, inclusion and/or exclusion criteria should be given. In case‐control studies, a case‐definition and the source for controls should be given. | X |  |  | Clearly described in the Methods section and Table 1. |
| *4. Are the interventions of interest clearly described?*  Treatments and placebo (where relevant) that are to be compared should be clearly described. | X |  |  | The interventions are clearly described. |
| *5. Are the distributions of principal confounders in each group of subjects to be compared clearly described?*  A list of principal confounders is provided. | X |  |  | Confounders have been listed. |
| *6. Are the main findings of the study clearly described?*  Simple outcome data (including denominators and numerators) should be reported for all major findings so that the reader can check the major analyses and conclusions. (This question does not cover statistical tests  which are considered below). |  | X |  | Results have been pooled between THA and TKA patients. Even if F value has been reported showing no effect of the type of surgery on the outcomes, it is not possible to check data related only to THA patients (which is the aim of this review). |
| *7. Does the study provide estimates of the random variability in the data for the main outcomes?*  In non-normally distributed data the inter‐quartile range of results should be reported. In normally distributed data the standard error, standard deviation or confidence intervals should be reported. If the distribution of the data is not described, it must be assumed that the estimates used were appropriate and the question should be answered yes. | X |  |  | Variability has been reported. |
| *8. Have all important adverse events that may be a consequence of the intervention been reported?*  This should be answered yes if the study demonstrates that there was a comprehensive attempt to measure adverse events. (A list of possible adverse events is provided). | X |  |  | Some measures to counteract adverse events of the training have been reported (e.g. “All patients received routine education about safety, transfers, and limb positioning, as well as typical exercises relative to their surgery.”, “Exercise execution and repetitions were reviewed by the physical therapist at each home visit.”) |
| *9. Have the characteristics of patients lost to follow‐up been described?*  This should be answered yes where there were no losses to follow‐up or where losses to follow‐up were so small that findings would be unaffected by their inclusion. This should be answered NO where a study does not report the number of patients lost to follow‐up. | X |  |  | Losses to follow-up were small. |
| *10. Have actual probability values been reported (e.g. 0.035 rather than < 0.05) for the main outcomes except where the probability value is less than 0.001?* | X |  |  | Probability values have been reported. |
| **External Validity** | **YES** | **NO** | **UTD** | **Notes/Justification** |
| *11. Were the subjects asked to participate in the study representative of the entire population from which they were recruited?*  The study must identify the source population for patients and describe how the patients were selected. Patients would be representative if they comprised the entire source population, an unselected sample of consecutive patients, or a random sample. Random sampling is only feasible where a list of all members of the relevant population exists. Where a study does not report the proportion of the source population from which the patients are derived, the question should be answered as unable to determine. | X |  |  | Patients were recruited by means of a large physical therapy provider. |
| *12. Were those subjects who were prepared to participate representative of the entire population from which they were recruited?*  The proportion of those asked who agreed should be stated. Validation that the sample was representative would include demonstrating that the distribution of the main confounding factors was the same in the study sample and the source population. | X |  |  | A large proportion of patients participate in the study, thus the sample can be considered representative. |
| *13. Were the staff, places, and facilities where the patients were treated, representative of the treatment the majority of patients receive?*  For the question to be answered yes the study should demonstrate that the intervention was representative of that in use in the source population. The question should be answered no if, for example, the intervention was  undertaken in a specialist centre unrepresentative of the hospitals most of the source population would attend. | X |  |  | Patients trained at home, and this representative of the treatment that the majority of patients receives. |
| **Internal validity – bias** | **YES** | **NO** | **UTD** | **Notes/Justification** |
| *14. Was an attempt made to blind study subjects to the intervention they have received?*  For studies where the patients would have no way of knowing which intervention they received, this should be answered yes. | X |  |  | Patients were unaware of their allocation. |
| *15. Was an attempt made to blind those measuring the main outcomes of the intervention?* |  | X |  | Patients were tested by their physical therapists which were not blinded on patients’ allocation. |
| *16. If any of the results of the study were based on “data dredging”, was this made clear?*  Any analyses that had not been planned at the outset of the study should be clearly indicated. If no retrospective unplanned subgroup analyses were reported, then answer yes. | X |  |  | No retrospective unplanned subgroup analyses were reported. |
| *17. In trials and cohort studies, do the analyses adjust for different lengths of follow‐up of patients, or in case-control studies, is the time period between the intervention and outcome the same for cases and controls?*  Where follow‐up was the same for all study patients the answer should yes.  If different lengths of follow‐up were adjusted for by, for example, survival analysis the answer should be yes. Studies where differences in follow‐up are ignored should be answered no. | X |  |  | All patients were tested at the same time points. |
| *18. Were the statistical tests used to assess the main outcomes appropriate?*  The statistical techniques used must be appropriate to the data. For example, Non-parametric methods should be used for small sample sizes. Where little statistical analysis has been undertaken but where there is no evidence of bias, the question should be answered yes. If the distribution of the data (normal or not) is not described it must be assumed that the estimates used were appropriate and the question should be answered yes. | X |  |  | Statistics were appropriate and correctly reported. |
| *19. Was compliance with the intervention/s reliable?*  Where there was non compliance with the allocated treatment or where there was contamination of one group, the question should be answered no.  For studies where the effect of any misclassification was likely to bias any association to the null, the question should be answered yes. | X |  |  | Compliance with the interventions was reliable. |
| *20. Were the main outcome measures used accurate (valid and reliable)?*  For studies where the outcome measures are clearly described, the question should be answered yes. For studies which refer to other work or that demonstrates the outcome measures are accurate, the question should be  answered as yes. | X |  |  | Outcome measures were accurate. |
| **Internal validity ‐ confounding (selection bias)** | **YES** | **NO** | **UTD** | **Notes/Justification** |
| *21. Were the patients in different intervention groups (trials and cohort studies) or were the cases and controls (case‐control studies) recruited from the same population?*  For example, patients for all comparison groups should be selected from the same hospital. The question should be answered unable to determine for cohort and case-control studies where there is no information concerning the source of patients included in the study. | X |  |  | Yes, patients were recruited by the same physical therapy provider. |
| *22. Were study subjects in different intervention groups (trials and cohort- studies) or were the cases and controls (case‐control studies) recruited over the same period of time?*  For a study which does not specify the time period over which patients were recruited, the question should be answered as unable to determine. | X |  |  | Patients were recruited over the same time period. |
| *23. Were study subjects randomised to intervention groups?*  Studies which state that subjects were randomized should be answered yes except where method of randomisation would not ensure random allocation.  For example, alternate allocation would score no because it is predictable. | X |  |  | Participants were randomly allocated to one of the groups. |
| *24. Was the randomised intervention assignment concealed from both patients and health care staff until recruitment was complete and irrevocable?*  All non‐randomised studies should be answered no. If assignment was concealed from patients but not from staff, it should be answered no. |  | X |  | Physical therapists were aware of patients’ allocation. |
| *25. Was there adequate adjustment for confounding in the analyses from which the main findings were drawn?*  This question should be answered no for trials if: the main conclusions of the study were based on analyses of treatment rather than intention to treat; the distribution of known confounders in the different treatment groups was  not described; or the distribution of known confounders differed between the treatment groups but was not taken into account in the analyses. In non-randomized studies if the effect of the main confounders was not investigated or confounding was demonstrated but no adjustment was made in the final analyses the question should be answered as no. | X |  |  | Distribution of potentially confounders was the same between the groups. |
| *26. Were losses of patients to follow‐up taken into account?*  If the numbers of patients lost to follow‐up are not reported, the question should be answered as unable to determine. If the proportion lost to follow-up was too small to affect the main findings, the question should be answered yes. | X |  |  | Lost in follow-up was small. |
| **Power** | **YES** | **NO** | **UTD** | **Notes/Justification** |
| *27. Did the study have sufficient power to detect a clinically important effect where the probability value for a difference being due to chance is less than 5%?*  Sample sizes have been calculated to detect a difference of x% and y%. |  | X |  | The sample size calculation and the probability for a difference have been reported. However, patients with TKA and THA have been pooled together to reach an adequate sample size for the study.  The aim of this systematic review is to investigate the effects of training only in THA patients, and thus the study has not an adequate number of participants to our purpose. |
| **Total Score 23/27 = 85.1 %** | | | | |

| **Jogi et al. 2016** | | | | |
| --- | --- | --- | --- | --- |
| **Reporting** | **YES** | **NO** | **UTD** | **Notes/Justification** |
| *1. Is the hypothesis/aim/objective of the study clearly described?* | X |  |  | The aim is clearly described. |
| *2. Are the main outcomes to be measured clearly described in the Introduction or Methods section?*  If the main outcomes are first mentioned in the Results section, the question should be answered no. | X |  |  | Clearly described in Methods section. |
| *3. Are the characteristics of the patients included in the study clearly described?*  In cohort studies and trials, inclusion and/or exclusion criteria should be given. In case‐control studies, a case‐definition and the source for controls should be given. | X |  |  | Clearly described in the Partecipants paragraph into Methods section |
| *4. Are the interventions of interest clearly described?*  Treatments and placebo (where relevant) that are to be compared should be clearly described. | X |  |  | The interventions are clearly described. |
| *5. Are the distributions of principal confounders in each group of subjects to be compared clearly described?*  A list of principal confounders is provided. | X |  |  | The principal confounders were listed and compared between the two groups |
| *6. Are the main findings of the study clearly described?*  Simple outcome data (including denominators and numerators) should be reported for all major findings so that the reader can check the major analyses and conclusions. (This question does not cover statistical tests  which are considered below). |  | X |  | Despite no significant differences were observed  between the THA and the TKA groups, and all analyses were performed with the data on the THA and TKA  groups pooled together. Thus it is not possible to check data related only to THA patients (which is the aim of this review). |
| *7. Does the study provide estimates of the random variability in the data for the main outcomes?*  In non-normally distributed data the inter‐quartile range of results should be reported. In normally distributed data the standard error, standard deviation or confidence intervals should be reported. If the distribution of the data is not described, it must be assumed that the estimates used were appropriate and the question should be answered yes. | X |  |  | Outcome variables were determined to be normally distributed, and consequently means and standard deviations have been used as summary statistics |
| *8. Have all important adverse events that may be a consequence of the intervention been reported?*  This should be answered yes if the study demonstrates that there was a comprehensive attempt to measure adverse events. (A list of possible adverse events is provided). | X |  |  | Were provided safety instructions to execute the home exercise program without risks. |
| *9. Have the characteristics of patients lost to follow‐up been described?*  This should be answered yes where there were no losses to follow‐up or where losses to follow‐up were so small that findings would be unaffected by their inclusion. This should be answered NO where a study does not report the number of patients lost to follow‐up. | X |  |  | The characteristic of patients who dropped out have been described |
| *10. Have actual probability values been reported (e.g. 0.035 rather than < 0.05) for the main outcomes except where the probability value is less than 0.001?* | X |  |  | Probability values have been reported. |
| **External Validity** | **YES** | **NO** | **UTD** | **Notes/Justification** |
| *11. Were the subjects asked to participate in the study representative of the entire population from which they were recruited?*  The study must identify the source population for patients and describe how the patients were selected. Patients would be representative if they comprised the entire source population, an unselected sample of consecutive patients, or a random sample. Random sampling is only feasible where a list of all members of the relevant population exists. Where a study does not report the proportion of the source population from which the patients are derived, the question should be answered as unable to determine. |  |  | X | The study does not report the proportion of the source population from which the patients are derived |
| *12. Were those subjects who were prepared to participate representative of the entire population from which they were recruited?*  The proportion of those asked who agreed should be stated. Validation that the sample was representative would include demonstrating that the distribution of the main confounding factors was the same in the study sample and the source population. |  |  | X | The study does not report the proportion of the source population from which the patients are derived |
| *13. Were the staff, places, and facilities where the patients were treated, representative of the treatment the majority of patients receive?*  For the question to be answered yes the study should demonstrate that the intervention was representative of that in use in the source population. The question should be answered no if, for example, the intervention was  undertaken in a specialist centre unrepresentative of the hospitals most of the source population would attend. | X |  |  | The patients have been treated following the standard rehabilitation protocol used for home rehabilitation program by the outpatients provider involved in the study. |
| **Internal validity – bias** | **YES** | **NO** | **UTD** | **Notes/Justification** |
| *14. Was an attempt made to blind study subjects to the intervention they have received?*  For studies where the patients would have no way of knowing which intervention they received, this should be answered yes. | X |  |  | The patients made an Home rehabilitation Program in which the presence of additional balance training was been established by a con flip, randomly, without the patients were aware of the two different protocol. |
| *15. Was an attempt made to blind those measuring the main outcomes of the intervention?* |  | X |  | There is no information about it |
| *16. If any of the results of the study were based on “data dredging”, was this made clear?*  Any analyses that had not been planned at the outset of the study should be clearly indicated. If no retrospective unplanned subgroup analyses were reported, then answer yes. | X |  |  | No retrospective unplanned subgroup analyses were reported. |
| *17. In trials and cohort studies, do the analyses adjust for different lengths of follow‐up of patients, or in case-control studies, is the time period between the intervention and outcome the same for cases and controls?*  Where follow‐up was the same for all study patients the answer should yes.  If different lengths of follow‐up were adjusted for by, for example, survival analysis the answer should be yes. Studies where differences in follow‐up are ignored should be answered no. | X |  |  | the time period between the intervention and outcome is the same for the groups of intervention. |
| *18. Were the statistical tests used to assess the main outcomes appropriate?*  The statistical techniques used must be appropriate to the data. For example, Non-parametric methods should be used for small sample sizes. Where little statistical analysis has been undertaken but where there is no evidence of bias, the question should be answered yes. If the distribution of the data (normal or not) is not described it must be assumed that the estimates used were appropriate and the question should be answered yes. | X |  |  | Statistics were appropriate and correctly reported. |
| *19. Was compliance with the intervention/s reliable?*  Where there was non compliance with the allocated treatment or where there was contamination of one group, the question should be answered no.  For studies where the effect of any misclassification was likely to bias any association to the null, the question should be answered yes. | X |  |  | Compliance with the interventions was reliable. |
| *20. Were the main outcome measures used accurate (valid and reliable)?*  For studies where the outcome measures are clearly described, the question should be answered yes. For studies which refer to other work or that demonstrates the outcome measures are accurate, the question should be  answered as yes. | X |  |  | Outcome measures were accurate. |
| **Internal validity ‐ confounding (selection bias)** | **YES** | **NO** | **UTD** | **Notes/Justification** |
| *21. Were the patients in different intervention groups (trials and cohort studies) or were the cases and controls (case‐control studies) recruited from the same population?*  For example, patients for all comparison groups should be selected from the same hospital. The question should be answered unable to determine for cohort and case-control studies where there is no information concerning the source of patients included in the study. | X |  |  | Participants in this study were a sample of convenience of  consecutive individuals referred to a large outpatient provider of home physical therapy |
| *22. Were study subjects in different intervention groups (trials and cohort- studies) or were the cases and controls (case‐control studies) recruited over the same period of time?*  For a study which does not specify the time period over which patients were recruited, the question should be answered as unable to determine. | X |  |  | The patients has been recruited between April 2006 and April 2009. |
| *23. Were study subjects randomised to intervention groups?*  Studies which state that subjects were randomized should be answered yes except where method of randomisation would not ensure random allocation.  For example, alternate allocation would score no because it is predictable. | X |  |  | The study subjects were randomised in the intervention and control groups. |
| *24. Was the randomised intervention assignment concealed from both patients and health care staff until recruitment was complete and irrevocable?*  All non‐randomised studies should be answered no. If assignment was concealed from patients but not from staff, it should be answered no. |  | X |  | The patients made an Home rehabilitation Program in which the presence of additional balance training was been established randomly by a coin flip. The patients were aware of the two different protocol on the contrary the healthcare staff known the treatment. |
| *25. Was there adequate adjustment for confounding in the analyses from which the main findings were drawn?*  This question should be answered no for trials if: the main conclusions of the study were based on analyses of treatment rather than intention to treat; the distribution of known confounders in the different treatment groups was  not described; or the distribution of known confounders differed between the treatment groups but was not taken into account in the analyses. In non-randomized studies if the effect of the main confounders was not investigated or confounding was demonstrated but no adjustment was made in the final analyses the question should be answered as no. | X |  |  | The confounders have been taken into account in the analysis |
| *26. Were losses of patients to follow‐up taken into account?*  If the numbers of patients lost to follow‐up are not reported, the question should be answered as unable to determine. If the proportion lost to follow-up was too small to affect the main findings, the question should be answered yes. | X |  |  | The patients lost at the follow-up have been reported and haven’t affected the main findings. |
| **Power** | **YES** | **NO** | **UTD** | **Notes/Justification** |
| *27. Did the study have sufficient power to detect a clinically important effect where the probability value for a difference being due to chance is less than 5%?*  Sample sizes have been calculated to detect a difference of x% and y%. |  | X |  | The sample size calculation and the probability for a difference have been reported. However, patients with TKA and THA have been pooled together to reach an adequate sample size for the study.  The aim of this systematic review is to investigate the effects of training only in THA patients, and thus the study has not an adequate number of participants to our purpose. |
| **Total Score 21/27 = 77.8 %** | | | | |

| **Larkin et al. 2013** | | | | |
| --- | --- | --- | --- | --- |
| **Reporting** | **YES** | **NO** | **UTD** | **Notes/Justification** |
| *1. Is the hypothesis/aim/objective of the study clearly described?* | X |  |  | The aim is clearly described. |
| *2. Are the main outcomes to be measured clearly described in the Introduction or Methods section?*  If the main outcomes are first mentioned in the Results section, the question should be answered no. | X |  |  | Clearly described in the Patients and Methods section. |
| *3. Are the characteristics of the patients included in the study clearly described?*  In cohort studies and trials, inclusion and/or exclusion criteria should be given. In case‐control studies, a case‐definition and the source for controls should be given. | X |  |  | Characteristics of the patient are clearly described in the Methods section. |
| *4. Are the interventions of interest clearly described?*  Treatments and placebo (where relevant) that are to be compared should be clearly described. |  |  |  |  |
| *5. Are the distributions of principal confounders in each group of subjects to be compared clearly described?*  A list of principal confounders is provided. | X |  |  | A list of the principal confounders is provided |
| *6. Are the main findings of the study clearly described?*  Simple outcome data (including denominators and numerators) should be reported for all major findings so that the reader can check the major analyses and conclusions. (This question does not cover statistical tests  which are considered below). | X |  |  | The main findings of the study are clearly described |
| *7. Does the study provide estimates of the random variability in the data for the main outcomes?*  In non-normally distributed data the inter‐quartile range of results should be reported. In normally distributed data the standard error, standard deviation or confidence intervals should be reported. If the distribution of the data is not described, it must be assumed that the estimates used were appropriate and the question should be answered yes. | X |  |  | The distribution of the data has not been reported and we assume that the estimates used were appropriate |
| *8. Have all important adverse events that may be a consequence of the intervention been reported?*  This should be answered yes if the study demonstrates that there was a comprehensive attempt to measure adverse events. (A list of possible adverse events is provided). |  |  |  |  |
| *9. Have the characteristics of patients lost to follow‐up been described?*  This should be answered yes where there were no losses to follow‐up or where losses to follow‐up were so small that findings would be unaffected by their inclusion. This should be answered NO where a study does not report the number of patients lost to follow‐up. |  |  |  |  |
| *10. Have actual probability values been reported (e.g. 0.035 rather than < 0.05) for the main outcomes except where the probability value is less than 0.001?* | X |  |  | Probability values have been reported. |
| **External Validity** | **YES** | **NO** | **UTD** | **Notes/Justification** |
| *11. Were the subjects asked to participate in the study representative of the entire population from which they were recruited?*  The study must identify the source population for patients and describe how the patients were selected. Patients would be representative if they comprised the entire source population, an unselected sample of consecutive patients, or a random sample. Random sampling is only feasible where a list of all members of the relevant population exists. Where a study does not report the proportion of the source population from which the patients are derived, the question should be answered as unable to determine. |  |  | X | The study does not report the proportion of the source population from which the patients are derived |
| *12. Were those subjects who were prepared to participate representative of the entire population from which they were recruited?*  The proportion of those asked who agreed should be stated. Validation that the sample was representative would include demonstrating that the distribution of the main confounding factors was the same in the study sample and the source population. |  | X |  | The study does not report the proportion and characteristic of the source population from which the patients are derived. |
| *13. Were the staff, places, and facilities where the patients were treated, representative of the treatment the majority of patients receive?*  For the question to be answered yes the study should demonstrate that the intervention was representative of that in use in the source population. The question should be answered no if, for example, the intervention was  undertaken in a specialist centre unrepresentative of the hospitals most of the source population would attend. |  |  |  |  |
| **Internal validity – bias** | **YES** | **NO** | **UTD** | **Notes/Justification** |
| *14. Was an attempt made to blind study subjects to the intervention they have received?*  For studies where the patients would have no way of knowing which intervention they received, this should be answered yes. |  |  |  |  |
| *15. Was an attempt made to blind those measuring the main outcomes of the intervention?* |  |  |  |  |
| *16. If any of the results of the study were based on “data dredging”, was this made clear?*  Any analyses that had not been planned at the outset of the study should be clearly indicated. If no retrospective unplanned subgroup analyses were reported, then answer yes. | X |  |  | No retrospective unplanned subgroup analyses were reported. |
| *17. In trials and cohort studies, do the analyses adjust for different lengths of follow‐up of patients, or in case-control studies, is the time period between the intervention and outcome the same for cases and controls?*  Where follow‐up was the same for all study patients the answer should yes.  If different lengths of follow‐up were adjusted for by, for example, survival analysis the answer should be yes. Studies where differences in follow‐up are ignored should be answered no. |  |  |  |  |
| *18. Were the statistical tests used to assess the main outcomes appropriate?*  The statistical techniques used must be appropriate to the data. For example, Non-parametric methods should be used for small sample sizes. Where little statistical analysis has been undertaken but where there is no evidence of bias, the question should be answered yes. If the distribution of the data (normal or not) is not described it must be assumed that the estimates used were appropriate and the question should be answered yes. | X |  |  | Statistics were appropriate and correctly reported. |
| *19. Was compliance with the intervention/s reliable?*  Where there was non compliance with the allocated treatment or where there was contamination of one group, the question should be answered no.  For studies where the effect of any misclassification was likely to bias any association to the null, the question should be answered yes. |  |  |  |  |
| *20. Were the main outcome measures used accurate (valid and reliable)?*  For studies where the outcome measures are clearly described, the question should be answered yes. For studies which refer to other work or that demonstrates the outcome measures are accurate, the question should be  answered as yes. | X |  |  | Outcome measures were accurate. |
| **Internal validity ‐ confounding (selection bias)** | **YES** | **NO** | **UTD** | **Notes/Justification** |
| *21. Were the patients in different intervention groups (trials and cohort studies) or were the cases and controls (case‐control studies) recruited from the same population?*  For example, patients for all comparison groups should be selected from the same hospital. The question should be answered unable to determine for cohort and case-control studies where there is no information concerning the source of patients included in the study. |  | X |  | patient recruitment started both in the outpatient  clinics and through screening of an institutional total joint  registry. To be eligible for the study, patients needed to  meet the following criteria |
| *22. Were study subjects in different intervention groups (trials and cohort- studies) or were the cases and controls (case‐control studies) recruited over the same period of time?*  For a study which does specify the time period over which patients were recruited, the question should be answered as unable to determine. | X |  |  | The cohorts included patients who have been underwent to surgery between 1 and five years prior. Thus we could assume that the referring time point is the same for all the cohorts |
| *23. Were study subjects randomised to intervention groups?*  Studies which state that subjects were randomized should be answered yes except where method of randomisation would not ensure random allocation.  For example, alternate allocation would score no because it is predictable. |  |  |  |  |
| *24. Was the randomised intervention assignment concealed from both patients and health care staff until recruitment was complete and irrevocable?*  All non‐randomised studies should be answered no. If assignment was concealed from patients but not from staff, it should be answered no. |  |  |  |  |
| *25. Was there adequate adjustment for confounding in the analyses from which the main findings were drawn?*  This question should be answered no for trials if: the main conclusions of the study were based on analyses of treatment rather than intention to treat; the distribution of known confounders in the different treatment groups was  not described; or the distribution of known confounders differed between the treatment groups but was not taken into account in the analyses. In non-randomized studies if the effect of the main confounders was not investigated or confounding was demonstrated but no adjustment was made in the final analyses the question should be answered as no. |  |  |  |  |
| *26. Were losses of patients to follow‐up taken into account?*  If the numbers of patients lost to follow‐up are not reported, the question should be answered as unable to determine. If the proportion lost to follow-up was too small to affect the main findings, the question should be answered yes. |  |  |  |  |
| **Power** | **YES** | **NO** | **UTD** | **Notes/Justification** |
| *27. Did the study have sufficient power to detect a clinically important effect where the probability value for a difference being due to chance is less than 5%?*  Sample sizes have been calculated to detect a difference of x% and y%. |  |  |  |  |
| **Total Score 11/14 = 78.6 %** | | | | |

| **Lavigne et al. 2010** | | | | |
| --- | --- | --- | --- | --- |
| **Reporting** | **YES** | **NO** | **UTD** | **Notes/Justification** |
| *1. Is the hypothesis/aim/objective of the study clearly described?* | X |  |  | The aim is clearly described. |
| *2. Are the main outcomes to be measured clearly described in the Introduction or Methods section?*  If the main outcomes are first mentioned in the Results section, the question should be answered no. | X |  |  | Outcomes are clearly described in the Methods section. |
| *3. Are the characteristics of the patients included in the study clearly described?*  In cohort studies and trials, inclusion and/or exclusion criteria should be given. In case‐control studies, a case‐definition and the source for controls should be given. | X |  |  | Clearly described in the Methods section. |
| *4. Are the interventions of interest clearly described?*  Treatments and placebo (where relevant) that are to be compared should be clearly described. | X |  |  | The interventions are clearly described. |
| *5. Are the distributions of principal confounders in each group of subjects to be compared clearly described?*  A list of principal confounders is provided. | X |  |  | List of confounders and distribution in each group has been provided. |
| *6. Are the main findings of the study clearly described?*  Simple outcome data (including denominators and numerators) should be reported for all major findings so that the reader can check the major analyses and conclusions. (This question does not cover statistical tests  which are considered below). | X |  |  | Outcome data have been reported. |
| *7. Does the study provide estimates of the random variability in the data for the main outcomes?*  In non-normally distributed data the inter‐quartile range of results should be reported. In normally distributed data the standard error, standard deviation or confidence intervals should be reported. If the distribution of the data is not described, it must be assumed that the estimates used were appropriate and the question should be answered yes. | X |  |  | Variability has been reported. |
| *8. Have all important adverse events that may be a consequence of the intervention been reported?*  This should be answered yes if the study demonstrates that there was a comprehensive attempt to measure adverse events. (A list of possible adverse events is provided). |  |  | X | Adverse events have not been reported. |
| *9. Have the characteristics of patients lost to follow‐up been described?*  This should be answered yes where there were no losses to follow‐up or where losses to follow‐up were so small that findings would be unaffected by their inclusion. This should be answered NO where a study does not report the number of patients lost to follow‐up. | X |  |  | Small losses at the follow-up. |
| *10. Have actual probability values been reported (e.g. 0.035 rather than < 0.05) for the main outcomes except where the probability value is less than 0.001?* | X |  |  | Probability values have been reported. |
| **External Validity** | **YES** | **NO** | **UTD** | **Notes/Justification** |
| *11. Were the subjects asked to participate in the study representative of the entire population from which they were recruited?*  The study must identify the source population for patients and describe how the patients were selected. Patients would be representative if they comprised the entire source population, an unselected sample of consecutive patients, or a random sample. Random sampling is only feasible where a list of all members of the relevant population exists. Where a study does not report the proportion of the source population from which the patients are derived, the question should be answered as unable to determine. | X |  |  | Source population has been reported. |
| *12. Were those subjects who were prepared to participate representative of the entire population from which they were recruited?*  The proportion of those asked who agreed should be stated. Validation that the sample was representative would include demonstrating that the distribution of the main confounding factors was the same in the study sample and the source population. | X |  |  | Distribution of confounding factors has been described and is the same between the groups. |
| *13. Were the staff, places, and facilities where the patients were treated, representative of the treatment the majority of patients receive?*  For the question to be answered yes the study should demonstrate that the intervention was representative of that in use in the source population. The question should be answered no if, for example, the intervention was  undertaken in a specialist centre unrepresentative of the hospitals most of the source population would attend. | X |  |  | Staff, places and facilities are representative. |
| **Internal validity – bias** | **YES** | **NO** | **UTD** | **Notes/Justification** |
| *14. Was an attempt made to blind study subjects to the intervention they have received?*  For studies where the patients would have no way of knowing which intervention they received, this should be answered yes. | X |  |  | There was an attempt to blind participants. |
| *15. Was an attempt made to blind those measuring the main outcomes of the intervention?* | X |  |  | There was an attempt to blind the evaluators. |
| *16. If any of the results of the study were based on “data dredging”, was this made clear?*  Any analyses that had not been planned at the outset of the study should be clearly indicated. If no retrospective unplanned subgroup analyses were reported, then answer yes. | X |  |  | No retrospective unplanned subgroup analyses were reported. |
| *17. In trials and cohort studies, do the analyses adjust for different lengths of follow‐up of patients, or in case-control studies, is the time period between the intervention and outcome the same for cases and controls?*  Where follow‐up was the same for all study patients the answer should yes.  If different lengths of follow‐up were adjusted for by, for example, survival analysis the answer should be yes. Studies where differences in follow‐up are ignored should be answered no. | X |  |  | All patients were tested at the same time points. |
| *18. Were the statistical tests used to assess the main outcomes appropriate?*  The statistical techniques used must be appropriate to the data. For example, Non-parametric methods should be used for small sample sizes. Where little statistical analysis has been undertaken but where there is no evidence of bias, the question should be answered yes. If the distribution of the data (normal or not) is not described it must be assumed that the estimates used were appropriate and the question should be answered yes. | X |  |  | Statistics were appropriate and correctly reported. |
| *19. Was compliance with the intervention/s reliable?*  Where there was non compliance with the allocated treatment or where there was contamination of one group, the question should be answered no.  For studies where the effect of any misclassification was likely to bias any association to the null, the question should be answered yes. | X |  |  | Compliance with the interventions was reliable. |
| *20. Were the main outcome measures used accurate (valid and reliable)?*  For studies where the outcome measures are clearly described, the question should be answered yes. For studies which refer to other work or that demonstrates the outcome measures are accurate, the question should be  answered as yes. | X |  |  | Outcome measures are accurate. |
| **Internal validity ‐ confounding (selection bias)** | **YES** | **NO** | **UTD** | **Notes/Justification** |
| *21. Were the patients in different intervention groups (trials and cohort studies) or were the cases and controls (case‐control studies) recruited from the same population?*  For example, patients for all comparison groups should be selected from the same hospital. The question should be answered unable to determine for cohort and case-control studies where there is no information concerning the source of patients included in the study. | X |  |  | Patients have been recruited from the same population. |
| *22. Were study subjects in different intervention groups (trials and cohort- studies) or were the cases and controls (case‐control studies) recruited over the same period of time?*  For a study which does not specify the time period over which patients were recruited, the question should be answered as unable to determine. | X |  |  | Time period has been reported and is the same between-groups. |
| *23. Were study subjects randomised to intervention groups?*  Studies which state that subjects were randomized should be answered yes except where method of randomisation would not ensure random allocation.  For example, alternate allocation would score no because it is predictable. | X |  |  | Participants were randomly allocated to one of the groups. |
| *24. Was the randomised intervention assignment concealed from both patients and health care staff until recruitment was complete and irrevocable?*  All non‐randomised studies should be answered no. If assignment was concealed from patients but not from staff, it should be answered no. | X |  |  | Allocation was concealed until the intervention was performed. |
| *25. Was there adequate adjustment for confounding in the analyses from which the main findings were drawn?*  This question should be answered no for trials if: the main conclusions of the study were based on analyses of treatment rather than intention to treat; the distribution of known confounders in the different treatment groups was  not described; or the distribution of known confounders differed between the treatment groups but was not taken into account in the analyses. In non-randomized studies if the effect of the main confounders was not investigated or confounding was demonstrated but no adjustment was made in the final analyses the question should be answered as no. | X |  |  | Confounders have been considered during the analysis. |
| *26. Were losses of patients to follow‐up taken into account?*  If the numbers of patients lost to follow‐up are not reported, the question should be answered as unable to determine. If the proportion lost to follow-up was too small to affect the main findings, the question should be answered yes. | X |  |  | Losses in follow-up have been considered. |
| **Power** | **YES** | **NO** | **UTD** | **Notes/Justification** |
| *27. Did the study have sufficient power to detect a clinically important effect where the probability value for a difference being due to chance is less than 5%?*  Sample sizes have been calculated to detect a difference of x% and y%. | X |  |  | Yes, power analysis has been performed and reported. |
| **Total Score 26/27 = 96.2 %** | | | | |

| **Lugade et al. 2008** | | | | |
| --- | --- | --- | --- | --- |
| **Reporting** | **YES** | **NO** | **UTD** | **Notes/Justification** |
| *1. Is the hypothesis/aim/objective of the study clearly described?* | X |  |  | The aim is clearly described. |
| *2. Are the main outcomes to be measured clearly described in the Introduction or Methods section?*  If the main outcomes are first mentioned in the Results section, the question should be answered no. | X |  |  | Outcomes are clearly described in the Methods section. |
| *3. Are the characteristics of the patients included in the study clearly described?*  In cohort studies and trials, inclusion and/or exclusion criteria should be given. In case‐control studies, a case‐definition and the source for controls should be given. | X |  |  | Clearly described in the Methods section. |
| *4. Are the interventions of interest clearly described?*  Treatments and placebo (where relevant) that are to be compared should be clearly described. |  |  |  |  |
| *5. Are the distributions of principal confounders in each group of subjects to be compared clearly described?*  A list of principal confounders is provided. | X |  |  | Confounders have been listed. |
| *6. Are the main findings of the study clearly described?*  Simple outcome data (including denominators and numerators) should be reported for all major findings so that the reader can check the major analyses and conclusions. (This question does not cover statistical tests  which are considered below). | X |  |  | Outcome data have been reported. |
| *7. Does the study provide estimates of the random variability in the data for the main outcomes?*  In non-normally distributed data the inter‐quartile range of results should be reported. In normally distributed data the standard error, standard deviation or confidence intervals should be reported. If the distribution of the data is not described, it must be assumed that the estimates used were appropriate and the question should be answered yes. | X |  |  | Variability has been reported. |
| *8. Have all important adverse events that may be a consequence of the intervention been reported?*  This should be answered yes if the study demonstrates that there was a comprehensive attempt to measure adverse events. (A list of possible adverse events is provided). |  |  |  |  |
| *9. Have the characteristics of patients lost to follow‐up been described?*  This should be answered yes where there were no losses to follow‐up or where losses to follow‐up were so small that findings would be unaffected by their inclusion. This should be answered NO where a study does not report the number of patients lost to follow‐up. |  |  |  |  |
| *10. Have actual probability values been reported (e.g. 0.035 rather than < 0.05) for the main outcomes except where the probability value is less than 0.001?* | X |  |  | Probability values have been reported. |
| **External Validity** | **YES** | **NO** | **UTD** | **Notes/Justification** |
| *11. Were the subjects asked to participate in the study representative of the entire population from which they were recruited?*  The study must identify the source population for patients and describe how the patients were selected. Patients would be representative if they comprised the entire source population, an unselected sample of consecutive patients, or a random sample. Random sampling is only feasible where a list of all members of the relevant population exists. Where a study does not report the proportion of the source population from which the patients are derived, the question should be answered as unable to determine. | X |  |  | The source population has been reported. |
| *12. Were those subjects who were prepared to participate representative of the entire population from which they were recruited?*  The proportion of those asked who agreed should be stated. Validation that the sample was representative would include demonstrating that the distribution of the main confounding factors was the same in the study sample and the source population. |  |  | X | The proportion of patients included has not been reported. |
| *13. Were the staff, places, and facilities where the patients were treated, representative of the treatment the majority of patients receive?*  For the question to be answered yes the study should demonstrate that the intervention was representative of that in use in the source population. The question should be answered no if, for example, the intervention was  undertaken in a specialist centre unrepresentative of the hospitals most of the source population would attend. |  |  |  |  |
| **Internal validity – bias** | **YES** | **NO** | **UTD** | **Notes/Justification** |
| *14. Was an attempt made to blind study subjects to the intervention they have received?*  For studies where the patients would have no way of knowing which intervention they received, this should be answered yes. |  |  |  |  |
| *15. Was an attempt made to blind those measuring the main outcomes of the intervention?* |  |  |  |  |
| *16. If any of the results of the study were based on “data dredging”, was this made clear?*  Any analyses that had not been planned at the outset of the study should be clearly indicated. If no retrospective unplanned subgroup analyses were reported, then answer yes. | X |  |  | No retrospective analysis have been reported. |
| *17. In trials and cohort studies, do the analyses adjust for different lengths of follow‐up of patients, or in case-control studies, is the time period between the intervention and outcome the same for cases and controls?*  Where follow‐up was the same for all study patients the answer should yes.  If different lengths of follow‐up were adjusted for by, for example, survival analysis the answer should be yes. Studies where differences in follow‐up are ignored should be answered no. |  |  |  |  |
| *18. Were the statistical tests used to assess the main outcomes appropriate?*  The statistical techniques used must be appropriate to the data. For example, Non-parametric methods should be used for small sample sizes. Where little statistical analysis has been undertaken but where there is no evidence of bias, the question should be answered yes. If the distribution of the data (normal or not) is not described it must be assumed that the estimates used were appropriate and the question should be answered yes. | X |  |  | Statistics are appropiate. |
| *19. Was compliance with the intervention/s reliable?*  Where there was non compliance with the allocated treatment or where there was contamination of one group, the question should be answered no.  For studies where the effect of any misclassification was likely to bias any association to the null, the question should be answered yes. |  |  |  |  |
| *20. Were the main outcome measures used accurate (valid and reliable)?*  For studies where the outcome measures are clearly described, the question should be answered yes. For studies which refer to other work or that demonstrates the outcome measures are accurate, the question should be  answered as yes. | X |  |  | Outcome measures are accurate. |
| **Internal validity ‐ confounding (selection bias)** | **YES** | **NO** | **UTD** | **Notes/Justification** |
| *21. Were the patients in different intervention groups (trials and cohort studies) or were the cases and controls (case‐control studies) recruited from the same population?*  For example, patients for all comparison groups should be selected from the same hospital. The question should be answered unable to determine for cohort and case-control studies where there is no information concerning the source of patients included in the study. | X |  |  | Yes, the source populations have been reported. |
| *22. Were study subjects in different intervention groups (trials and cohort- studies) or were the cases and controls (case‐control studies) recruited over the same period of time?*  For a study which does not specify the time period over which patients were recruited, the question should be answered as unable to determine. |  |  | X | Time period has not been reported. |
| *23. Were study subjects randomised to intervention groups?*  Studies which state that subjects were randomized should be answered yes except where method of randomisation would not ensure random allocation.  For example, alternate allocation would score no because it is predictable. |  |  |  |  |
| *24. Was the randomised intervention assignment concealed from both patients and health care staff until recruitment was complete and irrevocable?*  All non‐randomised studies should be answered no. If assignment was concealed from patients but not from staff, it should be answered no. |  |  |  |  |
| *25. Was there adequate adjustment for confounding in the analyses from which the main findings were drawn?*  This question should be answered no for trials if: the main conclusions of the study were based on analyses of treatment rather than intention to treat; the distribution of known confounders in the different treatment groups was  not described; or the distribution of known confounders differed between the treatment groups but was not taken into account in the analyses. In non-randomized studies if the effect of the main confounders was not investigated or confounding was demonstrated but no adjustment was made in the final analyses the question should be answered as no. |  |  |  |  |
| *26. Were losses of patients to follow‐up taken into account?*  If the numbers of patients lost to follow‐up are not reported, the question should be answered as unable to determine. If the proportion lost to follow-up was too small to affect the main findings, the question should be answered yes. |  |  |  |  |
| **Power** | **YES** | **NO** | **UTD** | **Notes/Justification** |
| *27. Did the study have sufficient power to detect a clinically important effect where the probability value for a difference being due to chance is less than 5%?*  Sample sizes have been calculated to detect a difference of x% and y%. |  |  |  |  |
| **Total Score 12/14 = 85.7 %** | | | | |

| **Majewski et al. 2005** | | | | |
| --- | --- | --- | --- | --- |
| **Reporting** | **YES** | **NO** | **UTD** | **Notes/Justification** |
| *1. Is the hypothesis/aim/objective of the study clearly described?* | X |  |  | The aim is clearly described. |
| *2. Are the main outcomes to be measured clearly described in the Introduction or Methods section?*  If the main outcomes are first mentioned in the Results section, the question should be answered no. | X |  |  | Clearly described in Patients and Methods section. |
| *3. Are the characteristics of the patients included in the study clearly described?*  In cohort studies and trials, inclusion and/or exclusion criteria should be given. In case‐control studies, a case‐definition and the source for controls should be given. | X |  |  | Characteristics of the patient are clearly described in the in Patients and Methods section. |
| *4. Are the interventions of interest clearly described?*  Treatments and placebo (where relevant) that are to be compared should be clearly described. |  |  |  |  |
| *5. Are the distributions of principal confounders in each group of subjects to be compared clearly described?*  A list of principal confounders is provided. |  | X |  | The patients group and the control group were matched only for gender and age, and no measurement has been provided about this. |
| *6. Are the main findings of the study clearly described?*  Simple outcome data (including denominators and numerators) should be reported for all major findings so that the reader can check the major analyses and conclusions. (This question does not cover statistical tests  which are considered below). |  | X |  | The main findings of the study can’t be checked by the reader |
| *7. Does the study provide estimates of the random variability in the data for the main outcomes?*  In non-normally distributed data the inter‐quartile range of results should be reported. In normally distributed data the standard error, standard deviation or confidence intervals should be reported. If the distribution of the data is not described, it must be assumed that the estimates used were appropriate and the question should be answered yes. | X |  |  | The data are normally distributed |
| *8. Have all important adverse events that may be a consequence of the intervention been reported?*  This should be answered yes if the study demonstrates that there was a comprehensive attempt to measure adverse events. (A list of possible adverse events is provided). |  |  |  |  |
| *9. Have the characteristics of patients lost to follow‐up been described?*  This should be answered yes where there were no losses to follow‐up or where losses to follow‐up were so small that findings would be unaffected by their inclusion. This should be answered NO where a study does not report the number of patients lost to follow‐up. |  |  |  |  |
| *10. Have actual probability values been reported (e.g. 0.035 rather than < 0.05) for the main outcomes except where the probability value is less than 0.001?* | X |  |  | Probability values have been reported. |
| **External Validity** | **YES** | **NO** | **UTD** | **Notes/Justification** |
| *11. Were the subjects asked to participate in the study representative of the entire population from which they were recruited?*  The study must identify the source population for patients and describe how the patients were selected. Patients would be representative if they comprised the entire source population, an unselected sample of consecutive patients, or a random sample. Random sampling is only feasible where a list of all members of the relevant population exists. Where a study does not report the proportion of the source population from which the patients are derived, the question should be answered as unable to determine. |  |  | X | The study does not report the proportion of the source population from which the patients are derived. The sample is made of consecutive patients. |
| *12. Were those subjects who were prepared to participate representative of the entire population from which they were recruited?*  The proportion of those asked who agreed should be stated. Validation that the sample was representative would include demonstrating that the distribution of the main confounding factors was the same in the study sample and the source population. |  | X |  | The study does not report the proportion and characteristic of the source population from which the patients are derived. |
| *13. Were the staff, places, and facilities where the patients were treated, representative of the treatment the majority of patients receive?*  For the question to be answered yes the study should demonstrate that the intervention was representative of that in use in the source population. The question should be answered no if, for example, the intervention was  undertaken in a specialist centre unrepresentative of the hospitals most of the source population would attend. |  |  |  |  |
| **Internal validity – bias** | **YES** | **NO** | **UTD** | **Notes/Justification** |
| *14. Was an attempt made to blind study subjects to the intervention they have received?*  For studies where the patients would have no way of knowing which intervention they received, this should be answered yes. |  |  |  |  |
| *15. Was an attempt made to blind those measuring the main outcomes of the intervention?* |  |  |  |  |
| *16. If any of the results of the study were based on “data dredging”, was this made clear?*  Any analyses that had not been planned at the outset of the study should be clearly indicated. If no retrospective unplanned subgroup analyses were reported, then answer yes. | X |  |  | No retrospective unplanned subgroup analyses were reported. |
| *17. In trials and cohort studies, do the analyses adjust for different lengths of follow‐up of patients, or in case-control studies, is the time period between the intervention and outcome the same for cases and controls?*  Where follow‐up was the same for all study patients the answer should yes.  If different lengths of follow‐up were adjusted for by, for example, survival analysis the answer should be yes. Studies where differences in follow‐up are ignored should be answered no. |  |  |  |  |
| *18. Were the statistical tests used to assess the main outcomes appropriate?*  The statistical techniques used must be appropriate to the data. For example, Non-parametric methods should be used for small sample sizes. Where little statistical analysis has been undertaken but where there is no evidence of bias, the question should be answered yes. If the distribution of the data (normal or not) is not described it must be assumed that the estimates used were appropriate and the question should be answered yes. | X |  |  | Statistics were appropriate and correctly reported. |
| *19. Was compliance with the intervention/s reliable?*  Where there was non compliance with the allocated treatment or where there was contamination of one group, the question should be answered no.  For studies where the effect of any misclassification was likely to bias any association to the null, the question should be answered yes. |  |  |  |  |
| *20. Were the main outcome measures used accurate (valid and reliable)?*  For studies where the outcome measures are clearly described, the question should be answered yes. For studies which refer to other work or that demonstrates the outcome measures are accurate, the question should be  answered as yes. |  | X |  | The main findings of the study can’t be checked by the reader |
| **Internal validity ‐ confounding (selection bias)** | **YES** | **NO** | **UTD** | **Notes/Justification** |
| *21. Were the patients in different intervention groups (trials and cohort studies) or were the cases and controls (case‐control studies) recruited from the same population?*  For example, patients for all comparison groups should be selected from the same hospital. The question should be answered unable to determine for cohort and case-control studies where there is no information concerning the source of patients included in the study. |  |  | X | There is no information about the source population. |
| *22. Were study subjects in different intervention groups (trials and cohort- studies) or were the cases and controls (case‐control studies) recruited over the same period of time?*  For a study which does specify the time period over which patients were recruited, the question should be answered as unable to determine. |  |  | X | The sample is made of consecutive patients but the time period over the patients were recruited has not been specified. |
| *23. Were study subjects randomised to intervention groups?*  Studies which state that subjects were randomized should be answered yes except where method of randomisation would not ensure random allocation.  For example, alternate allocation would score no because it is predictable. |  |  |  |  |
| *24. Was the randomised intervention assignment concealed from both patients and health care staff until recruitment was complete and irrevocable?*  All non‐randomised studies should be answered no. If assignment was concealed from patients but not from staff, it should be answered no. |  |  |  |  |
| *25. Was there adequate adjustment for confounding in the analyses from which the main findings were drawn?*  This question should be answered no for trials if: the main conclusions of the study were based on analyses of treatment rather than intention to treat; the distribution of known confounders in the different treatment groups was  not described; or the distribution of known confounders differed between the treatment groups but was not taken into account in the analyses. In non-randomized studies if the effect of the main confounders was not investigated or confounding was demonstrated but no adjustment was made in the final analyses the question should be answered as no. |  |  |  |  |
| *26. Were losses of patients to follow‐up taken into account?*  If the numbers of patients lost to follow‐up are not reported, the question should be answered as unable to determine. If the proportion lost to follow-up was too small to affect the main findings, the question should be answered yes. |  |  |  |  |
| **Power** | **YES** | **NO** | **UTD** | **Notes/Justification** |
| *27. Did the study have sufficient power to detect a clinically important effect where the probability value for a difference being due to chance is less than 5%?*  Sample sizes have been calculated to detect a difference of x% and y%. |  |  |  |  |
| **Total Score 7/14 = 50.0 %** | | | | |

| **Merle et al. 2009** | | | | |
| --- | --- | --- | --- | --- |
| **Reporting** | **YES** | **NO** | **UTD** | **Notes/Justification** |
| *1. Is the hypothesis/aim/objective of the study clearly described?* | X |  |  | The aim is clearly described. |
| *2. Are the main outcomes to be measured clearly described in the Introduction or Methods section?*  If the main outcomes are first mentioned in the Results section, the question should be answered no. | X |  |  | Outcomes are clearly described in the Methods section. |
| *3. Are the characteristics of the patients included in the study clearly described?*  In cohort studies and trials, inclusion and/or exclusion criteria should be given. In case‐control studies, a case‐definition and the source for controls should be given. | X |  |  | Clearly described in the Methods section. |
| *4. Are the interventions of interest clearly described?*  Treatments and placebo (where relevant) that are to be compared should be clearly described. |  |  |  |  |
| *5. Are the distributions of principal confounders in each group of subjects to be compared clearly described?*  A list of principal confounders is provided. |  | X |  | The study has only one group of patients. |
| *6. Are the main findings of the study clearly described?*  Simple outcome data (including denominators and numerators) should be reported for all major findings so that the reader can check the major analyses and conclusions. (This question does not cover statistical tests  which are considered below). | X |  |  | Outcome data have been reported. |
| *7. Does the study provide estimates of the random variability in the data for the main outcomes?*  In non-normally distributed data the inter‐quartile range of results should be reported. In normally distributed data the standard error, standard deviation or confidence intervals should be reported. If the distribution of the data is not described, it must be assumed that the estimates used were appropriate and the question should be answered yes. | X |  |  | Variability has been reported. |
| *8. Have all important adverse events that may be a consequence of the intervention been reported?*  This should be answered yes if the study demonstrates that there was a comprehensive attempt to measure adverse events. (A list of possible adverse events is provided). |  |  |  |  |
| *9. Have the characteristics of patients lost to follow‐up been described?*  This should be answered yes where there were no losses to follow‐up or where losses to follow‐up were so small that findings would be unaffected by their inclusion. This should be answered NO where a study does not report the number of patients lost to follow‐up. |  |  |  |  |
| *10. Have actual probability values been reported (e.g. 0.035 rather than < 0.05) for the main outcomes except where the probability value is less than 0.001?* |  | X |  | Statistical analysis has not been described. |
| **External Validity** | **YES** | **NO** | **UTD** | **Notes/Justification** |
| *11. Were the subjects asked to participate in the study representative of the entire population from which they were recruited?*  The study must identify the source population for patients and describe how the patients were selected. Patients would be representative if they comprised the entire source population, an unselected sample of consecutive patients, or a random sample. Random sampling is only feasible where a list of all members of the relevant population exists. Where a study does not report the proportion of the source population from which the patients are derived, the question should be answered as unable to determine. |  | X |  | Source population has not been reported. |
| *12. Were those subjects who were prepared to participate representative of the entire population from which they were recruited?*  The proportion of those asked who agreed should be stated. Validation that the sample was representative would include demonstrating that the distribution of the main confounding factors was the same in the study sample and the source population. |  | X |  | The sample is small and the magnitude of the population where they were recruited is not known. |
| *13. Were the staff, places, and facilities where the patients were treated, representative of the treatment the majority of patients receive?*  For the question to be answered yes the study should demonstrate that the intervention was representative of that in use in the source population. The question should be answered no if, for example, the intervention was  undertaken in a specialist centre unrepresentative of the hospitals most of the source population would attend. |  |  |  |  |
| **Internal validity – bias** | **YES** | **NO** | **UTD** | **Notes/Justification** |
| *14. Was an attempt made to blind study subjects to the intervention they have received?*  For studies where the patients would have no way of knowing which intervention they received, this should be answered yes. |  |  |  |  |
| *15. Was an attempt made to blind those measuring the main outcomes of the intervention?* |  |  |  |  |
| *16. If any of the results of the study were based on “data dredging”, was this made clear?*  Any analyses that had not been planned at the outset of the study should be clearly indicated. If no retrospective unplanned subgroup analyses were reported, then answer yes. |  |  | X |  |
| *17. In trials and cohort studies, do the analyses adjust for different lengths of follow‐up of patients, or in case-control studies, is the time period between the intervention and outcome the same for cases and controls?*  Where follow‐up was the same for all study patients the answer should yes.  If different lengths of follow‐up were adjusted for by, for example, survival analysis the answer should be yes. Studies where differences in follow‐up are ignored should be answered no. |  |  |  |  |
| *18. Were the statistical tests used to assess the main outcomes appropriate?*  The statistical techniques used must be appropriate to the data. For example, Non-parametric methods should be used for small sample sizes. Where little statistical analysis has been undertaken but where there is no evidence of bias, the question should be answered yes. If the distribution of the data (normal or not) is not described it must be assumed that the estimates used were appropriate and the question should be answered yes. |  |  | X | Statistics have not been reported. |
| *19. Was compliance with the intervention/s reliable?*  Where there was non compliance with the allocated treatment or where there was contamination of one group, the question should be answered no.  For studies where the effect of any misclassification was likely to bias any association to the null, the question should be answered yes. |  |  |  |  |
| *20. Were the main outcome measures used accurate (valid and reliable)?*  For studies where the outcome measures are clearly described, the question should be answered yes. For studies which refer to other work or that demonstrates the outcome measures are accurate, the question should be  answered as yes. | X |  |  | Outcome measures are accurate. |
| **Internal validity ‐ confounding (selection bias)** | **YES** | **NO** | **UTD** | **Notes/Justification** |
| *21. Were the patients in different intervention groups (trials and cohort studies) or were the cases and controls (case‐control studies) recruited from the same population?*  For example, patients for all comparison groups should be selected from the same hospital. The question should be answered unable to determine for cohort and case-control studies where there is no information concerning the source of patients included in the study. |  | X |  | There is just one group of patients. |
| *22. Were study subjects in different intervention groups (trials and cohort- studies) or were the cases and controls (case‐control studies) recruited over the same period of time?*  For a study which does not specify the time period over which patients were recruited, the question should be answered as unable to determine. |  | X |  | The study has not a control group. |
| *23. Were study subjects randomised to intervention groups?*  Studies which state that subjects were randomized should be answered yes except where method of randomisation would not ensure random allocation.  For example, alternate allocation would score no because it is predictable. |  |  |  |  |
| *24. Was the randomised intervention assignment concealed from both patients and health care staff until recruitment was complete and irrevocable?*  All non‐randomised studies should be answered no. If assignment was concealed from patients but not from staff, it should be answered no. |  |  |  |  |
| *25. Was there adequate adjustment for confounding in the analyses from which the main findings were drawn?*  This question should be answered no for trials if: the main conclusions of the study were based on analyses of treatment rather than intention to treat; the distribution of known confounders in the different treatment groups was  not described; or the distribution of known confounders differed between the treatment groups but was not taken into account in the analyses. In non-randomized studies if the effect of the main confounders was not investigated or confounding was demonstrated but no adjustment was made in the final analyses the question should be answered as no. |  |  |  |  |
| *26. Were losses of patients to follow‐up taken into account?*  If the numbers of patients lost to follow‐up are not reported, the question should be answered as unable to determine. If the proportion lost to follow-up was too small to affect the main findings, the question should be answered yes. |  |  |  |  |
| **Power** | **YES** | **NO** | **UTD** | **Notes/Justification** |
| *27. Did the study have sufficient power to detect a clinically important effect where the probability value for a difference being due to chance is less than 5%?*  Sample sizes have been calculated to detect a difference of x% and y%. |  |  |  |  |
| **Total Score 6/14 = 42.8 %** | | | | |

| **Nantel et al. 2008** | | | | |
| --- | --- | --- | --- | --- |
| **Reporting** | **YES** | **NO** | **UTD** | **Notes/Justification** |
| *1. Is the hypothesis/aim/objective of the study clearly described?* | X |  |  | The aim is clearly described. |
| *2. Are the main outcomes to be measured clearly described in the Introduction or Methods section?*  If the main outcomes are first mentioned in the Results section, the question should be answered no. | X |  |  | Outcomes are clearly described in the Methods section. |
| *3. Are the characteristics of the patients included in the study clearly described?*  In cohort studies and trials, inclusion and/or exclusion criteria should be given. In case‐control studies, a case‐definition and the source for controls should be given. | X |  |  | Clearly described in the Methods section and Table 1. |
| *4. Are the interventions of interest clearly described?*  Treatments and placebo (where relevant) that are to be compared should be clearly described. |  |  |  |  |
| *5. Are the distributions of principal confounders in each group of subjects to be compared clearly described?*  A list of principal confounders is provided. |  | X |  | Confounders have not been clearly described. |
| *6. Are the main findings of the study clearly described?*  Simple outcome data (including denominators and numerators) should be reported for all major findings so that the reader can check the major analyses and conclusions. (This question does not cover statistical tests  which are considered below). | X |  |  | The main findings are clearly described. |
| *7. Does the study provide estimates of the random variability in the data for the main outcomes?*  In non-normally distributed data the inter‐quartile range of results should be reported. In normally distributed data the standard error, standard deviation or confidence intervals should be reported. If the distribution of the data is not described, it must be assumed that the estimates used were appropriate and the question should be answered yes. | X |  |  | Variability has been reported. |
| *8. Have all important adverse events that may be a consequence of the intervention been reported?*  This should be answered yes if the study demonstrates that there was a comprehensive attempt to measure adverse events. (A list of possible adverse events is provided). |  |  |  |  |
| *9. Have the characteristics of patients lost to follow‐up been described?*  This should be answered yes where there were no losses to follow‐up or where losses to follow‐up were so small that findings would be unaffected by their inclusion. This should be answered NO where a study does not report the number of patients lost to follow‐up. |  |  |  |  |
| *10. Have actual probability values been reported (e.g. 0.035 rather than < 0.05) for the main outcomes except where the probability value is less than 0.001?* | X |  |  | Probability values have not been reported. |
| **External Validity** | **YES** | **NO** | **UTD** | **Notes/Justification** |
| *11. Were the subjects asked to participate in the study representative of the entire population from which they were recruited?*  The study must identify the source population for patients and describe how the patients were selected. Patients would be representative if they comprised the entire source population, an unselected sample of consecutive patients, or a random sample. Random sampling is only feasible where a list of all members of the relevant population exists. Where a study does not report the proportion of the source population from which the patients are derived, the question should be answered as unable to determine. |  |  | X | The source population has not been mentioned. |
| *12. Were those subjects who were prepared to participate representative of the entire population from which they were recruited?*  The proportion of those asked who agreed should be stated. Validation that the sample was representative would include demonstrating that the distribution of the main confounding factors was the same in the study sample and the source population. |  |  | X | The source population has not been mentioned. |
| *13. Were the staff, places, and facilities where the patients were treated, representative of the treatment the majority of patients receive?*  For the question to be answered yes the study should demonstrate that the intervention was representative of that in use in the source population. The question should be answered no if, for example, the intervention was  undertaken in a specialist centre unrepresentative of the hospitals most of the source population would attend. |  |  |  |  |
| **Internal validity – bias** | **YES** | **NO** | **UTD** | **Notes/Justification** |
| *14. Was an attempt made to blind study subjects to the intervention they have received?*  For studies where the patients would have no way of knowing which intervention they received, this should be answered yes. |  |  |  |  |
| *15. Was an attempt made to blind those measuring the main outcomes of the intervention?* |  |  |  |  |
| *16. If any of the results of the study were based on “data dredging”, was this made clear?*  Any analyses that had not been planned at the outset of the study should be clearly indicated. If no retrospective unplanned subgroup analyses were reported, then answer yes. | X |  |  | No retrospective analysis has been reported. |
| *17. In trials and cohort studies, do the analyses adjust for different lengths of follow‐up of patients, or in case-control studies, is the time period between the intervention and outcome the same for cases and controls?*  Where follow‐up was the same for all study patients the answer should yes.  If different lengths of follow‐up were adjusted for by, for example, survival analysis the answer should be yes. Studies where differences in follow‐up are ignored should be answered no. |  |  |  |  |
| *18. Were the statistical tests used to assess the main outcomes appropriate?*  The statistical techniques used must be appropriate to the data. For example, Non-parametric methods should be used for small sample sizes. Where little statistical analysis has been undertaken but where there is no evidence of bias, the question should be answered yes. If the distribution of the data (normal or not) is not described it must be assumed that the estimates used were appropriate and the question should be answered yes. | X |  |  | Statistics are appropriate. |
| *19. Was compliance with the intervention/s reliable?*  Where there was non compliance with the allocated treatment or where there was contamination of one group, the question should be answered no.  For studies where the effect of any misclassification was likely to bias any association to the null, the question should be answered yes. |  |  |  |  |
| *20. Were the main outcome measures used accurate (valid and reliable)?*  For studies where the outcome measures are clearly described, the question should be answered yes. For studies which refer to other work or that demonstrates the outcome measures are accurate, the question should be  answered as yes. | X |  |  | Outcome measures are clearly described |
| **Internal validity ‐ confounding (selection bias)** | **YES** | **NO** | **UTD** | **Notes/Justification** |
| *21. Were the patients in different intervention groups (trials and cohort studies) or were the cases and controls (case‐control studies) recruited from the same population?*  For example, patients for all comparison groups should be selected from the same hospital. The question should be answered unable to determine for cohort and case-control studies where there is no information concerning the source of patients included in the study. | X |  |  | The patients were recruited by the same rehabilitation centre and hospital. |
| *22. Were study subjects in different intervention groups (trials and cohort- studies) or were the cases and controls (case‐control studies) recruited over the same period of time?*  For a study which does not specify the time period over which patients were recruited, the question should be answered as unable to determine. |  |  | X | Time period in which patients were recruited has not been reported. |
| *23. Were study subjects randomised to intervention groups?*  Studies which state that subjects were randomized should be answered yes except where method of randomisation would not ensure random allocation.  For example, alternate allocation would score no because it is predictable. |  |  |  |  |
| *24. Was the randomised intervention assignment concealed from both patients and health care staff until recruitment was complete and irrevocable?*  All non‐randomised studies should be answered no. If assignment was concealed from patients but not from staff, it should be answered no. |  |  |  |  |
| *25. Was there adequate adjustment for confounding in the analyses from which the main findings were drawn?*  This question should be answered no for trials if: the main conclusions of the study were based on analyses of treatment rather than intention to treat; the distribution of known confounders in the different treatment groups was  not described; or the distribution of known confounders differed between the treatment groups but was not taken into account in the analyses. In non-randomized studies if the effect of the main confounders was not investigated or confounding was demonstrated but no adjustment was made in the final analyses the question should be answered as no. |  |  |  |  |
| *26. Were losses of patients to follow‐up taken into account?*  If the numbers of patients lost to follow‐up are not reported, the question should be answered as unable to determine. If the proportion lost to follow-up was too small to affect the main findings, the question should be answered yes. |  |  |  |  |
| **Power** | **YES** | **NO** | **UTD** | **Notes/Justification** |
| *27. Did the study have sufficient power to detect a clinically important effect where the probability value for a difference being due to chance is less than 5%?*  Sample sizes have been calculated to detect a difference of x% and y%. |  |  |  |  |
| **Total Score 10/14 = 71.4 %** | | | | |

| **Nantel et al. 2009** | | | | |
| --- | --- | --- | --- | --- |
| **Reporting** | **YES** | **NO** | **UTD** | **Notes/Justification** |
| *1. Is the hypothesis/aim/objective of the study clearly described?* | X |  |  | The aim is clearly described. |
| *2. Are the main outcomes to be measured clearly described in the Introduction or Methods section?*  If the main outcomes are first mentioned in the Results section, the question should be answered no. | X |  |  | Outcomes are clearly described in the Methods section. |
| *3. Are the characteristics of the patients included in the study clearly described?*  In cohort studies and trials, inclusion and/or exclusion criteria should be given. In case‐control studies, a case‐definition and the source for controls should be given. | X |  |  | Clearly described in the Methods section and Table 1. |
| *4. Are the interventions of interest clearly described?*  Treatments and placebo (where relevant) that are to be compared should be clearly described. |  |  |  |  |
| *5. Are the distributions of principal confounders in each group of subjects to be compared clearly described?*  A list of principal confounders is provided. |  | X |  | Confounders have not been clearly described. |
| *6. Are the main findings of the study clearly described?*  Simple outcome data (including denominators and numerators) should be reported for all major findings so that the reader can check the major analyses and conclusions. (This question does not cover statistical tests  which are considered below). | X |  |  | The main findings are clearly described. |
| *7. Does the study provide estimates of the random variability in the data for the main outcomes?*  In non-normally distributed data the inter‐quartile range of results should be reported. In normally distributed data the standard error, standard deviation or confidence intervals should be reported. If the distribution of the data is not described, it must be assumed that the estimates used were appropriate and the question should be answered yes. | X |  |  | Variability has been reported. |
| *8. Have all important adverse events that may be a consequence of the intervention been reported?*  This should be answered yes if the study demonstrates that there was a comprehensive attempt to measure adverse events. (A list of possible adverse events is provided). |  |  |  |  |
| *9. Have the characteristics of patients lost to follow‐up been described?*  This should be answered yes where there were no losses to follow‐up or where losses to follow‐up were so small that findings would be unaffected by their inclusion. This should be answered NO where a study does not report the number of patients lost to follow‐up. |  |  |  |  |
| *10. Have actual probability values been reported (e.g. 0.035 rather than < 0.05) for the main outcomes except where the probability value is less than 0.001?* | X |  |  | Probability values have not been reported. |
| **External Validity** | **YES** | **NO** | **UTD** | **Notes/Justification** |
| *11. Were the subjects asked to participate in the study representative of the entire population from which they were recruited?*  The study must identify the source population for patients and describe how the patients were selected. Patients would be representative if they comprised the entire source population, an unselected sample of consecutive patients, or a random sample. Random sampling is only feasible where a list of all members of the relevant population exists. Where a study does not report the proportion of the source population from which the patients are derived, the question should be answered as unable to determine. |  |  | X | The source population has not been mentioned. |
| *12. Were those subjects who were prepared to participate representative of the entire population from which they were recruited?*  The proportion of those asked who agreed should be stated. Validation that the sample was representative would include demonstrating that the distribution of the main confounding factors was the same in the study sample and the source population. |  |  | X | The source population has not been mentioned. |
| *13. Were the staff, places, and facilities where the patients were treated, representative of the treatment the majority of patients receive?*  For the question to be answered yes the study should demonstrate that the intervention was representative of that in use in the source population. The question should be answered no if, for example, the intervention was  undertaken in a specialist centre unrepresentative of the hospitals most of the source population would attend. |  |  |  |  |
| **Internal validity – bias** | **YES** | **NO** | **UTD** | **Notes/Justification** |
| *14. Was an attempt made to blind study subjects to the intervention they have received?*  For studies where the patients would have no way of knowing which intervention they received, this should be answered yes. |  |  |  |  |
| *15. Was an attempt made to blind those measuring the main outcomes of the intervention?* |  |  |  |  |
| *16. If any of the results of the study were based on “data dredging”, was this made clear?*  Any analyses that had not been planned at the outset of the study should be clearly indicated. If no retrospective unplanned subgroup analyses were reported, then answer yes. | X |  |  | No retrospective analysis have been reported. |
| *17. In trials and cohort studies, do the analyses adjust for different lengths of follow‐up of patients, or in case-control studies, is the time period between the intervention and outcome the same for cases and controls?*  Where follow‐up was the same for all study patients the answer should yes.  If different lengths of follow‐up were adjusted for by, for example, survival analysis the answer should be yes. Studies where differences in follow‐up are ignored should be answered no. |  |  |  |  |
| *18. Were the statistical tests used to assess the main outcomes appropriate?*  The statistical techniques used must be appropriate to the data. For example, Non-parametric methods should be used for small sample sizes. Where little statistical analysis has been undertaken but where there is no evidence of bias, the question should be answered yes. If the distribution of the data (normal or not) is not described it must be assumed that the estimates used were appropriate and the question should be answered yes. | X |  |  | Statistics are appropriate. |
| *19. Was compliance with the intervention/s reliable?*  Where there was non compliance with the allocated treatment or where there was contamination of one group, the question should be answered no.  For studies where the effect of any misclassification was likely to bias any association to the null, the question should be answered yes. |  |  |  |  |
| *20. Were the main outcome measures used accurate (valid and reliable)?*  For studies where the outcome measures are clearly described, the question should be answered yes. For studies which refer to other work or that demonstrates the outcome measures are accurate, the question should be  answered as yes. | X |  |  | Outcome measures are clearly described |
| **Internal validity ‐ confounding (selection bias)** | **YES** | **NO** | **UTD** | **Notes/Justification** |
| *21. Were the patients in different intervention groups (trials and cohort studies) or were the cases and controls (case‐control studies) recruited from the same population?*  For example, patients for all comparison groups should be selected from the same hospital. The question should be answered unable to determine for cohort and case-control studies where there is no information concerning the source of patients included in the study. |  | X |  | Patients were recruited from different populations. |
| *22. Were study subjects in different intervention groups (trials and cohort- studies) or were the cases and controls (case‐control studies) recruited over the same period of time?*  For a study which does not specify the time period over which patients were recruited, the question should be answered as unable to determine. |  |  | X | Time period in which patients were recruited has not been reported. |
| *23. Were study subjects randomised to intervention groups?*  Studies which state that subjects were randomized should be answered yes except where method of randomisation would not ensure random allocation.  For example, alternate allocation would score no because it is predictable. |  |  |  |  |
| *24. Was the randomised intervention assignment concealed from both patients and health care staff until recruitment was complete and irrevocable?*  All non‐randomised studies should be answered no. If assignment was concealed from patients but not from staff, it should be answered no. |  |  |  |  |
| *25. Was there adequate adjustment for confounding in the analyses from which the main findings were drawn?*  This question should be answered no for trials if: the main conclusions of the study were based on analyses of treatment rather than intention to treat; the distribution of known confounders in the different treatment groups was  not described; or the distribution of known confounders differed between the treatment groups but was not taken into account in the analyses. In non-randomized studies if the effect of the main confounders was not investigated or confounding was demonstrated but no adjustment was made in the final analyses the question should be answered as no. |  |  |  |  |
| *26. Were losses of patients to follow‐up taken into account?*  If the numbers of patients lost to follow‐up are not reported, the question should be answered as unable to determine. If the proportion lost to follow-up was too small to affect the main findings, the question should be answered yes. |  |  |  |  |
| **Power** | **YES** | **NO** | **UTD** | **Notes/Justification** |
| *27. Did the study have sufficient power to detect a clinically important effect where the probability value for a difference being due to chance is less than 5%?*  Sample sizes have been calculated to detect a difference of x% and y%. |  |  |  |  |
| **Total Score 9/14 = 64.2 %** | | | | |

| **Nelson et al. 2020** | | | | |
| --- | --- | --- | --- | --- |
| **Reporting** | **YES** | **NO** | **UTD** | **Notes/Justification** |
| *1. Is the hypothesis/aim/objective of the study clearly described?* | X |  |  | The aim is clearly described in the introduction section. |
| *2. Are the main outcomes to be measured clearly described in the Introduction or Methods section?*  If the main outcomes are first mentioned in the Results section, the question should be answered no. | X |  |  | Clearly described in the Methods section. |
| *3. Are the characteristics of the patients included in the study clearly described?*  In cohort studies and trials, inclusion and/or exclusion criteria should be given. In case‐control studies, a case‐definition and the source for controls should be given. | X |  |  | Clearly described in the Methods section. |
| *4. Are the interventions of interest clearly described?*  Treatments and placebo (where relevant) that are to be compared should be clearly described. | X |  |  | The interventions are clearly described in the methods section. |
| *5. Are the distributions of principal confounders in each group of subjects to be compared clearly described?*  A list of principal confounders is provided. | X |  |  | Confounders have been considered and listed in the Discussion section. |
| *6. Are the main findings of the study clearly described?*  Simple outcome data (including denominators and numerators) should be reported for all major findings so that the reader can check the major analyses and conclusions. (This question does not cover statistical tests  which are considered below). | X |  |  | Main findings are clearly reported. |
| *7. Does the study provide estimates of the random variability in the data for the main outcomes?*  In non-normally distributed data the inter‐quartile range of results should be reported. In normally distributed data the standard error, standard deviation or confidence intervals should be reported. If the distribution of the data is not described, it must be assumed that the estimates used were appropriate and the question should be answered yes. | X |  |  | Variability has been reported. |
| *8. Have all important adverse events that may be a consequence of the intervention been reported?*  This should be answered yes if the study demonstrates that there was a comprehensive attempt to measure adverse events. (A list of possible adverse events is provided). | X |  |  | Some measures to counteract adverse events of the training have been reported (e.g. exercises reviewing to hel patients in the execution) |
| *9. Have the characteristics of patients lost to follow‐up been described?*  This should be answered yes where there were no losses to follow‐up or where losses to follow‐up were so small that findings would be unaffected by their inclusion. This should be answered NO where a study does not report the number of patients lost to follow‐up. | X |  |  | There were no losses to follow-up. |
| *10. Have actual probability values been reported (e.g. 0.035 rather than < 0.05) for the main outcomes except where the probability value is less than 0.001?* | X |  |  | Probability values have been reported. |
| **External Validity** | **YES** | **NO** | **UTD** | **Notes/Justification** |
| *11. Were the subjects asked to participate in the study representative of the entire population from which they were recruited?*  The study must identify the source population for patients and describe how the patients were selected. Patients would be representative if they comprised the entire source population, an unselected sample of consecutive patients, or a random sample. Random sampling is only feasible where a list of all members of the relevant population exists. Where a study does not report the proportion of the source population from which the patients are derived, the question should be answered as unable to determine. | X |  |  | Patients were recruited by means of a large physical therapy provider. |
| *12. Were those subjects who were prepared to participate representative of the entire population from which they were recruited?*  The proportion of those asked who agreed should be stated. Validation that the sample was representative would include demonstrating that the distribution of the main confounding factors was the same in the study sample and the source population. | X |  |  | The sample was representative. |
| *13. Were the staff, places, and facilities where the patients were treated, representative of the treatment the majority of patients receive?*  For the question to be answered yes the study should demonstrate that the intervention was representative of that in use in the source population. The question should be answered no if, for example, the intervention was  undertaken in a specialist centre unrepresentative of the hospitals most of the source population would attend. |  | X |  | Patients in the intervention group were trained by tele-rehabilitation which greatly differs from the in person usual care. |
| **Internal validity – bias** | **YES** | **NO** | **UTD** | **Notes/Justification** |
| *14. Was an attempt made to blind study subjects to the intervention they have received?*  For studies where the patients would have no way of knowing which intervention they received, this should be answered yes. |  | X |  | Participants were not blinded to allocation. The patients in the intervention group knew they were not receiving usual care. |
| *15. Was an attempt made to blind those measuring the main outcomes of the intervention?* | X |  |  | The physiotherapists assessing outcomes at each time point were blinded to group allocation. |
| *16. If any of the results of the study were based on “data dredging”, was this made clear?*  Any analyses that had not been planned at the outset of the study should be clearly indicated. If no retrospective unplanned subgroup analyses were reported, then answer yes. | X |  |  | No retrospective unplanned subgroup analyses were reported. |
| *17. In trials and cohort studies, do the analyses adjust for different lengths of follow‐up of patients, or in case-control studies, is the time period between the intervention and outcome the same for cases and controls?*  Where follow‐up was the same for all study patients the answer should yes.  If different lengths of follow‐up were adjusted for by, for example, survival analysis the answer should be yes. Studies where differences in follow‐up are ignored should be answered no. | X |  |  | All patients were tested at the same time points. |
| *18. Were the statistical tests used to assess the main outcomes appropriate?*  The statistical techniques used must be appropriate to the data. For example, Non-parametric methods should be used for small sample sizes. Where little statistical analysis has been undertaken but where there is no evidence of bias, the question should be answered yes. If the distribution of the data (normal or not) is not described it must be assumed that the estimates used were appropriate and the question should be answered yes. | X |  |  | Statistics were appropriate and correctly reported. |
| *19. Was compliance with the intervention/s reliable?*  Where there was non compliance with the allocated treatment or where there was contamination of one group, the question should be answered no.  For studies where the effect of any misclassification was likely to bias any association to the null, the question should be answered yes. | X |  |  | Compliance with the interventions was reliable. |
| *20. Were the main outcome measures used accurate (valid and reliable)?*  For studies where the outcome measures are clearly described, the question should be answered yes. For studies which refer to other work or that demonstrates the outcome measures are accurate, the question should be  answered as yes. | X |  |  | Outcome measures were accurate. |
| **Internal validity ‐ confounding (selection bias)** | **YES** | **NO** | **UTD** | **Notes/Justification** |
| *21. Were the patients in different intervention groups (trials and cohort studies) or were the cases and controls (case‐control studies) recruited from the same population?*  For example, patients for all comparison groups should be selected from the same hospital. The question should be answered unable to determine for cohort and case-control studies where there is no information concerning the source of patients included in the study. | X |  |  | Patients were recruited from the same hospital. |
| *22. Were study subjects in different intervention groups (trials and cohort- studies) or were the cases and controls (case‐control studies) recruited over the same period of time?*  For a study which does not specify the time period over which patients were recruited, the question should be answered as unable to determine. | X |  |  | Patients were recruited over the same time period. |
| *23. Were study subjects randomised to intervention groups?*  Studies which state that subjects were randomized should be answered yes except where method of randomisation would not ensure random allocation.  For example, alternate allocation would score no because it is predictable. | X |  |  | Participants were randomly allocated to one of the groups. |
| *24. Was the randomised intervention assignment concealed from both patients and health care staff until recruitment was complete and irrevocable?*  All non‐randomised studies should be answered no. If assignment was concealed from patients but not from staff, it should be answered no. |  | X |  | Physical therapists were aware of patients’ allocation. |
| *25. Was there adequate adjustment for confounding in the analyses from which the main findings were drawn?*  This question should be answered no for trials if: the main conclusions of the study were based on analyses of treatment rather than intention to treat; the distribution of known confounders in the different treatment groups was  not described; or the distribution of known confounders differed between the treatment groups but was not taken into account in the analyses. In non-randomized studies if the effect of the main confounders was not investigated or confounding was demonstrated but no adjustment was made in the final analyses the question should be answered as no. | X |  |  | The different distribution of potentially confounders was considered in the final analysis. |
| *26. Were losses of patients to follow‐up taken into account?*  If the numbers of patients lost to follow‐up are not reported, the question should be answered as unable to determine. If the proportion lost to follow-up was too small to affect the main findings, the question should be answered yes. | X |  |  | There were no losses to follow-up. |
| **Power** | **YES** | **NO** | **UTD** | **Notes/Justification** |
| *27. Did the study have sufficient power to detect a clinically important effect where the probability value for a difference being due to chance is less than 5%?*  Sample sizes have been calculated to detect a difference of x% and y%. | X |  |  | A non-inferiority power calculation was used to determine sample size. Calculations were based on 80% power and an alpha value of 0.05. |
| **Total Score 24/27 =** **88,9 %** | | | | |

| **Ninomiya et al. 2018** | | | | |
| --- | --- | --- | --- | --- |
| **Reporting** | **YES** | **NO** | **UTD** | **Notes/Justification** |
| *1. Is the hypothesis/aim/objective of the study clearly described?* | X |  |  | The aim is clearly described. |
| *2. Are the main outcomes to be measured clearly described in the Introduction or Methods section?*  If the main outcomes are first mentioned in the Results section, the question should be answered no. | X |  |  | Clearly described in the Methods section. |
| *3. Are the characteristics of the patients included*  *in the study clearly described?*  In cohort studies and trials, inclusion and/or exclusion criteria should be given. In case‐control studies, a case‐definition and the source for controls should be given. | X |  |  | Clearly described in the Methods section and Table 1. |
| *4. Are the interventions of interest clearly described?*  Treatments and placebo (where relevant) that are to be compared should be clearly described. |  |  |  |  |
| *5. Are the distributions of principal confounders in each group of subjects to be compared clearly described?*  A list of principal confounders is provided. | X |  |  | Reported in the Methods section. |
| *6. Are the main findings of the study clearly described?*  Simple outcome data (including denominators and numerators) should be reported for all major findings so that the reader can check the major analyses and conclusions. (This question does not cover statistical tests  which are considered below). | X |  |  | Reported in the Results section and Table 3. |
| *7. Does the study provide estimates of the random variability in the data for the main outcomes?*  In non-normally distributed data the inter‐quartile range of results should be reported. In normally distributed data the standard error, standard deviation or confidence intervals should be reported. If the distribution of the data is not described, it must be assumed that the estimates used were appropriate and the question should be answered yes. | X |  |  | Standard deviation has been reported. |
| *8. Have all important adverse events that may be a consequence of the intervention been reported?*  This should be answered yes if the study demonstrates that there was a comprehensive attempt to measure adverse events. (A list of possible adverse events is provided). |  |  |  |  |
| *9. Have the characteristics of patients lost to follow‐up been described?*  This should be answered yes where there were no losses to follow‐up or where losses to follow‐up were so small that findings would be unaffected by their inclusion. This should be answered NO where a study does not report the number of patients lost to follow‐up. |  |  |  |  |
| *10. Have actual probability values been reported ( e.g. 0.035 rather than < 0.05) for the main outcomes except where the probability value is less than 0.001?* | X |  |  | Probability values have been reported. |
| **External Validity** | **YES** | **NO** | **UTD** | **Notes/Justification** |
| *11. Were the subjects asked to participate in the study representative of the entire population from which they were recruited?*  The study must identify the source population for patients and describe how the patients were selected. Patients would be representative if they comprised the entire source population, an unselected sample of consecutive patients, or a random sample. Random sampling is only feasible where a list of all members of the relevant population exists. Where a study does not report the proportion of the source population from which the patients are derived, the question should be answered as unable to determine. | X |  |  | Participants were representative. |
| *12. Were those subjects who were prepared to participate representative of the entire population from which they were recruited?*  The proportion of those asked who agreed should be stated. Validation that the sample was representative would include demonstrating that the distribution of the main confounding factors was the same in the study sample and the source population. |  |  | X | It is not known if the distribution of the main confounding factors was the same in the study sample and the source population. |
| *13. Were the staff, places, and facilities where the patients were treated, representative of the treatment the majority of patients receive?*  For the question to be answered yes the study should demonstrate that the intervention was representative of that in use in the source population. The question should be answered no if, for example, the intervention was  undertaken in a specialist centre unrepresentative of the hospitals most of the source population would attend. |  |  |  |  |
| **Internal validity – bias** | **YES** | **NO** | **UTD** | **Notes/Justification** |
| *14. Was an attempt made to blind study subjects to the intervention they have received?*  For studies where the patients would have no way of knowing which intervention they received, this should be answered yes. |  |  |  |  |
| *15. Was an attempt made to blind those measuring the main outcomes of the intervention?* |  |  |  |  |
| *16. If any of the results of the study were based on “data dredging”, was this made clear?*  Any analyses that had not been planned at the outset of the study should be clearly indicated. If no retrospective unplanned subgroup analyses were reported, then answer yes. | X |  |  | No retrospective unplanned subgroup analyses were reported. |
| *17. In trials and cohort studies, do the analyses adjust for different lengths of follow‐up of patients, or in case-control studies, is the time period between the intervention and outcome the same for cases and controls?*  Where follow‐up was the same for all study patients the answer should yes.  If different lengths of follow‐up were adjusted for by, for example, survival analysis the answer should be yes. Studies where differences in follow‐up are ignored should be answered no. |  |  |  |  |
| *18. Were the statistical tests used to assess the main outcomes appropriate?*  The statistical techniques used must be appropriate to the data. For example, Non-parametric methods should be used for small sample sizes. Where little statistical analysis has been undertaken but where there is no evidence of bias, the question should be answered yes. If the distribution of the data (normal or not) is not described it must be assumed that the estimates used were appropriate and the question should be answered yes. | X |  |  | Statistics were appropriate and correctly reported. |
| *19. Was compliance with the intervention/s reliable?*  Where there was non compliance with the allocated treatment or where there was contamination of one group, the question should be answered no.  For studies where the effect of any misclassification was likely to bias any association to the null, the question should be answered yes. |  |  |  |  |
| *20. Were the main outcome measures used accurate (valid and reliable)?*  For studies where the outcome measures are clearly described, the question should be answered yes. For studies which refer to other work or that demonstrates the outcome measures are accurate, the question should be  answered as yes. | X |  |  | Outcome measures are accurate. |
| **Internal validity ‐ confounding (selection bias)** | **YES** | **NO** | **UTD** | **Notes/Justification** |
| *21. Were the patients in different intervention groups (trials and cohort studies) or were the cases and controls (case‐control studies) recruited from the same population?*  For example, patients for all comparison groups should be selected from the same hospital. The question should be answered unable to determine for cohort and case-control studies where there is no information concerning the source of patients included in the study. | X |  |  | Patients were recruited from the same hospital. |
| *22. Were study subjects in different intervention groups (trials and cohort- studies) or were the cases and controls (case‐control studies) recruited over the same period of time?*  For a study which does not specify the time period over which patients were recruited, the question should be answered as unable to determine. | X |  |  | Patients were recruited over the same time period. |
| *23. Were study subjects randomised to intervention groups?*  Studies which state that subjects were randomized should be answered yes except where method of randomisation would not ensure random allocation.  For example, alternate allocation would score no because it is predictable. |  |  |  |  |
| *24. Was the randomised intervention assignment concealed from both patients and health care staff until recruitment was complete and irrevocable?*  All non‐randomised studies should be answered no. If assignment was concealed from patients but not from staff, it should be answered no. |  |  |  |  |
| *25. Was there adequate adjustment for confounding in the analyses from which the main findings were drawn?*  This question should be answered no for trials if: the main conclusions of the study were based on analyses of treatment rather than intention to treat; the distribution of known confounders in the different treatment groups was  not described; or the distribution of known confounders differed between the treatment groups but was not taken into account in the analyses. In non-randomized studies if the effect of the main confounders was not investigated or confounding was demonstrated but no adjustment was made in the final analyses the question should be answered as no. |  |  |  |  |
| *26. Were losses of patients to follow‐up taken into account?*  If the numbers of patients lost to follow‐up are not reported, the question should be answered as unable to determine. If the proportion lost to follow-up was too small to affect the main findings, the question should be  answered yes. |  |  |  |  |
| **Power** |  |  |  |  |
| *27. Did the study have sufficient power to detect a clinically important effect where the probability value for a difference being due to chance is less than 5%?*  Sample sizes have been calculated to detect a difference of x% and y%. |  |  |  |  |
| **Total Score 13/14 = 92.8 %** | | | | |

| **Pethe-Kania et al. 2017** | | | | |
| --- | --- | --- | --- | --- |
| **Reporting** | **YES** | **NO** | **UTD** | **Notes/Justification** |
| *1. Is the hypothesis/aim/objective of the study clearly described?* | X |  |  | The aim is clearly described. |
| *2. Are the main outcomes to be measured clearly described in the Introduction or Methods section?*  If the main outcomes are first mentioned in the Results section, the question should be answered no. |  | X |  | Clearly described in the Results section. |
| *3. Are the characteristics of the patients included in the study clearly described?*  In cohort studies and trials, inclusion and/or exclusion criteria should be given. In case‐control studies, a case‐definition and the source for controls should be given. | X |  |  | Clearly described in the Materials and Method section |
| *4. Are the interventions of interest clearly described?*  Treatments and placebo (where relevant) that are to be compared should be clearly described. | X |  |  | The interventions are clearly described. |
| *5. Are the distributions of principal confounders in each group of subjects to be compared clearly described?*  A list of principal confounders is provided. | X |  |  | A list of principal confounders is provided. |
| *6. Are the main findings of the study clearly described?*  Simple outcome data (including denominators and numerators) should be reported for all major findings so that the reader can check the major analyses and conclusions. (This question does not cover statistical tests  which are considered below). | X |  |  | The main findings of the study are clearly described |
| *7. Does the study provide estimates of the random variability in the data for the main outcomes?*  In non-normally distributed data the inter‐quartile range of results should be reported. In normally distributed data the standard error, standard deviation or confidence intervals should be reported. If the distribution of the data is not described, it must be assumed that the estimates used were appropriate and the question should be answered yes. | X |  |  | For the main outcomes were provided estimates of the random variability in the data |
| *8. Have all important adverse events that may be a consequence of the intervention been reported?*  This should be answered yes if the study demonstrates that there was a comprehensive attempt to measure adverse events. (A list of possible adverse events is provided). |  | X |  | No adverse event was reported, and there was no attempt to measure adverse event. |
| *9. Have the characteristics of patients lost to follow‐up been described?*  This should be answered yes where there were no losses to follow‐up or where losses to follow‐up were so small that findings would be unaffected by their inclusion. This should be answered NO where a study does not report the number of patients lost to follow‐up. | X |  |  | there were no losses to follow‐up |
| *10. Have actual probability values been reported (e.g. 0.035 rather than < 0.05) for the main outcomes except where the probability value is less than 0.001?* | X |  |  | Probability values have been reported. |
| **External Validity** | **YES** | **NO** | **UTD** | **Notes/Justification** |
| *11. Were the subjects asked to participate in the study representative of the entire population from which they were recruited?*  The study must identify the source population for patients and describe how the patients were selected. Patients would be representative if they comprised the entire source population, an unselected sample of consecutive patients, or a random sample. Random sampling is only feasible where a list of all members of the relevant population exists. Where a study does not report the proportion of the source population from which the patients are derived, the question should be answered as unable to determine. |  |  | X | The study involved 60 randomly selected patients who underwent total hip arthroplasty, and no further informations were provided |
| *12. Were those subjects who were prepared to participate representative of the entire population from which they were recruited?*  The proportion of those asked who agreed should be stated. Validation that the sample was representative would include demonstrating that the distribution of the main confounding factors was the same in the study sample and the source population. |  | X |  | There was no demonstration that the distribution of the confounders was the same in the study sample and the population source. |
| *13. Were the staff, places, and facilities where the patients were treated, representative of the treatment the majority of patients receive?*  For the question to be answered yes the study should demonstrate that the intervention was representative of that in use in the source population. The question should be answered no if, for example, the intervention was  undertaken in a specialist centre unrepresentative of the hospitals most of the source population would attend. |  |  | X | Both groups were involved in conventional rehabilitation and the intervention group also received additional treatment which was the object of the study. No other patients or population source were mentioned |
| **Internal validity – bias** | **YES** | **NO** | **UTD** | **Notes/Justification** |
| *14. Was an attempt made to blind study subjects to the intervention they have received?*  For studies where the patients would have no way of knowing which intervention they received, this should be answered yes. |  |  | X | Both groups were involved in conventional rehabilitation and the intervention group also received additional treatment which was the objective of the study. It’s not clear if the experimental group received was aware of the differences. No attempt to blind study subjects were described. |
| *15. Was an attempt made to blind those measuring the main outcomes of the intervention?* |  | X |  | There is no information about it |
| *16. If any of the results of the study were based on “data dredging”, was this made clear?*  Any analyses that had not been planned at the outset of the study should be clearly indicated. If no retrospective unplanned subgroup analyses were reported, then answer yes. | X |  |  | No retrospective unplanned subgroup analyses were reported. |
| *17. In trials and cohort studies, do the analyses adjust for different lengths of follow‐up of patients, or in case-control studies, is the time period between the intervention and outcome the same for cases and controls?*  Where follow‐up was the same for all study patients the answer should yes.  If different lengths of follow‐up were adjusted for by, for example, survival analysis the answer should be yes. Studies where differences in follow‐up are ignored should be answered no. | X |  |  | the time period between the intervention and outcome is the same for intervention and control group |
| *18. Were the statistical tests used to assess the main outcomes appropriate?*  The statistical techniques used must be appropriate to the data. For example, Non-parametric methods should be used for small sample sizes. Where little statistical analysis has been undertaken but where there is no evidence of bias, the question should be answered yes. If the distribution of the data (normal or not) is not described it must be assumed that the estimates used were appropriate and the question should be answered yes. | X |  |  | Statistics were appropriate and correctly reported. |
| *19. Was compliance with the intervention/s reliable?*  Where there was non compliance with the allocated treatment or where there was contamination of one group, the question should be answered no.  For studies where the effect of any misclassification was likely to bias any association to the null, the question should be answered yes. | X |  |  | Compliance with the interventions was reliable. |
| *20. Were the main outcome measures used accurate (valid and reliable)?*  For studies where the outcome measures are clearly described, the question should be answered yes. For studies which refer to other work or that demonstrates the outcome measures are accurate, the question should be  answered as yes. | X |  |  | Outcome measures were accurate. |
| **Internal validity ‐ confounding (selection bias)** | **YES** | **NO** | **UTD** | **Notes/Justification** |
| *21. Were the patients in different intervention groups (trials and cohort studies) or were the cases and controls (case‐control studies) recruited from the same population?*  For example, patients for all comparison groups should be selected from the same hospital. The question should be answered unable to determine for cohort and case-control studies where there is no information concerning the source of patients included in the study. |  |  | X | The study involved 60 randomly selected patients who underwent total hip arthroplasty, and no further information were provided |
| *22. Were study subjects in different intervention groups (trials and cohort- studies) or were the cases and controls (case‐control studies) recruited over the same period of time?*  For a study which does not specify the time period over which patients were recruited, the question should be answered as unable to determine. |  |  | X | The study involved 60 randomly selected patients who underwent total hip arthroplasty, and no further information were provided |
| *23. Were study subjects randomised to intervention groups?*  Studies which state that subjects were randomized should be answered yes except where method of randomisation would not ensure random allocation.  For example, alternate allocation would score no because it is predictable. | X |  |  | The study subjects were randomised to intervention groups |
| *24. Was the randomised intervention assignment concealed from both patients and health care staff until recruitment was complete and irrevocable?*  All non‐randomised studies should be answered no. If assignment was concealed from patients but not from staff, it should be answered no. |  |  | X | There is a lack of information about recruitment and randomization methods |
| *25. Was there adequate adjustment for confounding in the analyses from which the main findings were drawn?*  This question should be answered no for trials if: the main conclusions of the study were based on analyses of treatment rather than intention to treat; the distribution of known confounders in the different treatment groups was  not described; or the distribution of known confounders differed between the treatment groups but was not taken into account in the analyses. In non-randomized studies if the effect of the main confounders was not investigated or confounding was demonstrated but no adjustment was made in the final analyses the question should be answered as no. | X |  |  | The confounders have been taken into account in the analysis |
| *26. Were losses of patients to follow‐up taken into account?*  If the numbers of patients lost to follow‐up are not reported, the question should be answered as unable to determine. If the proportion lost to follow-up was too small to affect the main findings, the question should be answered yes. | X |  |  | No patient dropped out |
| **Power** | **YES** | **NO** | **UTD** | **Notes/Justification** |
| *27. Did the study have sufficient power to detect a clinically important effect where the probability value for a difference being due to chance is less than 5%?*  Sample sizes have been calculated to detect a difference of x% and y%. |  | X |  | The sample size calculation and the probability for a difference haven’t been reported. |
| **Total Score 15/27 = 55.6 %** | | | | |

| **Pohl et al. 2015** | | | | |
| --- | --- | --- | --- | --- |
| **Reporting** | **YES** | **NO** | **UTD** | **Notes/Justification** |
| *1. Is the hypothesis/aim/objective of the study clearly described?* | X |  |  | The aim is clearly described. |
| *2. Are the main outcomes to be measured clearly described in the Introduction or Methods section?*  If the main outcomes are first mentioned in the Results section, the question should be answered no. | X |  |  | Clearly described in the Procedures paragraph into Methods section. |
| *3. Are the characteristics of the patients included in the study clearly described?*  In cohort studies and trials, inclusion and/or exclusion criteria should be given. In case‐control studies, a case‐definition and the source for controls should be given. | X |  |  | Clearly described in the Participants paragraph into Methods section |
| *4. Are the interventions of interest clearly described?*  Treatments and placebo (where relevant) that are to be compared should be clearly described. | X |  |  | The interventions are clearly described. |
| *5. Are the distributions of principal confounders in each group of subjects to be compared clearly described?*  A list of principal confounders is provided. | X |  |  | Disturbing factors (age, height, weight, days after surgery, TKA / THA, male / female) were listed and no significant differences emerged between groups using the ANOVA test. |
| *6. Are the main findings of the study clearly described?*  Simple outcome data (including denominators and numerators) should be reported for all major findings so that the reader can check the major analyses and conclusions. (This question does not cover statistical tests  which are considered below). |  | X |  | Results have been pooled between THA and TKA patients. it is not possible to check data related only to THA patients (which is the aim of this review). |
| *7. Does the study provide estimates of the random variability in the data for the main outcomes?*  In non-normally distributed data the inter‐quartile range of results should be reported. In normally distributed data the standard error, standard deviation or confidence intervals should be reported. If the distribution of the data is not described, it must be assumed that the estimates used were appropriate and the question should be answered yes. | X |  |  | Outcome variables were determined to be normally distributed, and consequently means and standard deviations have been used as summary statistics |
| *8. Have all important adverse events that may be a consequence of the intervention been reported?*  This should be answered yes if the study demonstrates that there was a comprehensive attempt to measure adverse events. (A list of possible adverse events is provided). | X |  |  | The only negative effect measured is pain, which is mainly related to surgery but could be increased by exercises. All patients were treated for pain relief and there was no difference in pain medication use between the groups. |
| *9. Have the characteristics of patients lost to follow‐up been described?*  This should be answered yes where there were no losses to follow‐up or where losses to follow‐up were so small that findings would be unaffected by their inclusion. This should be answered NO where a study does not report the number of patients lost to follow‐up. | X |  |  | there were no losses to follow‐up |
| *10. Have actual probability values been reported (e.g. 0.035 rather than < 0.05) for the main outcomes except where the probability value is less than 0.001?* | X |  |  | Probability values have been reported. |
| **External Validity** | **YES** | **NO** | **UTD** | **Notes/Justification** |
| *11. Were the subjects asked to participate in the study representative of the entire population from which they were recruited?*  The study must identify the source population for patients and describe how the patients were selected. Patients would be representative if they comprised the entire source population, an unselected sample of consecutive patients, or a random sample. Random sampling is only feasible where a list of all members of the relevant population exists. Where a study does not report the proportion of the source population from which the patients are derived, the question should be answered as unable to determine. |  |  | X | The study does not report the proportion of the source population from which the patients are derived |
| *12. Were those subjects who were prepared to participate representative of the entire population from which they were recruited?*  The proportion of those asked who agreed should be stated. Validation that the sample was representative would include demonstrating that the distribution of the main confounding factors was the same in the study sample and the source population. |  |  | X | The study does not report the proportion of the source population from which the patients are derived |
| *13. Were the staff, places, and facilities where the patients were treated, representative of the treatment the majority of patients receive?*  For the question to be answered yes, the study should demonstrate that the intervention was representative of that in use in the source population. The question should be answered no if, for example, the intervention was  undertaken in a specialist centre unrepresentative of the hospitals most of the source population would attend. | X |  |  | The patients are usually treated following the rehabilitation protocol which has been applied to the groups and that only differs for the volume. |
| **Internal validity – bias** | **YES** | **NO** | **UTD** | **Notes/Justification** |
| *14. Was an attempt made to blind study subjects to the intervention they have received?*  For studies where the patients would have no way of knowing which intervention they received, this should be answered yes. | X |  |  | The standard treatment included sensorimotor training session yet, but the three groups received three different volume of training, though there no specifications about blinding procedure, it’s reasonable to think that the patients couldn’t understand the difference of volume. |
| *15. Was an attempt made to blind those measuring the main outcomes of the intervention?* |  | X |  | There is no information about it |
| *16. If any of the results of the study were based on “data dredging”, was this made clear?*  Any analyses that had not been planned at the outset of the study should be clearly indicated. If no retrospective unplanned subgroup analyses were reported, then answer yes. | X |  |  | No retrospective unplanned subgroup analyses were reported. |
| *17. In trials and cohort studies, do the analyses adjust for different lengths of follow‐up of patients, or in case-control studies, is the time period between the intervention and outcome the same for cases and controls?*  Where follow‐up was the same for all study patients the answer should yes.  If different lengths of follow‐up were adjusted for by, for example, survival analysis the answer should be yes. Studies where differences in follow‐up are ignored should be answered no. | X |  |  | the time period between the intervention and outcome is the same for the groups of intervention. |
| *18. Were the statistical tests used to assess the main outcomes appropriate?*  The statistical techniques used must be appropriate to the data. For example, Non-parametric methods should be used for small sample sizes. Where little statistical analysis has been undertaken but where there is no evidence of bias, the question should be answered yes. If the distribution of the data (normal or not) is not described it must be assumed that the estimates used were appropriate and the question should be answered yes. | X |  |  | Statistics were appropriate and correctly reported. |
| *19. Was compliance with the intervention/s reliable?*  Where there was non compliance with the allocated treatment or where there was contamination of one group, the question should be answered no.  For studies where the effect of any misclassification was likely to bias any association to the null, the question should be answered yes. | X |  |  | Compliance with the interventions was reliable. |
| *20. Were the main outcome measures used accurate (valid and reliable)?*  For studies where the outcome measures are clearly described, the question should be answered yes. For studies which refer to other work or that demonstrates the outcome measures are accurate, the question should be  answered as yes. | X |  |  | Outcome measures were accurate. |
| **Internal validity ‐ confounding (selection bias)** | **YES** | **NO** | **UTD** | **Notes/Justification** |
| *21. Were the patients in different intervention groups (trials and cohort studies) or were the cases and controls (case‐control studies) recruited from the same population?*  For example, patients for all comparison groups should be selected from the same hospital. The question should be answered unable to determine for cohort and case-control studies where there is no information concerning the source of patients included in the study. | X |  |  | Yes, patients were recruited by the same inpatient orthopaedic rehabilitation clinic. |
| *22. Were study subjects in different intervention groups (trials and cohort- studies) or were the cases and controls (case‐control studies) recruited over the same period of time?*  For a study which does not specify the time period over which patients were recruited, the question should be answered as unable to determine. | X |  |  | The sample is made by unselected consecutive patients presenting to an inpatient orthopaedic rehabilitation clinic. |
| *23. Were study subjects randomised to intervention groups?*  Studies which state that subjects were randomized should be answered yes except where method of randomisation would not ensure random allocation.  For example, alternate allocation would score no because it is predictable. | X |  |  | The study subjects were randomised in the three-intervention group |
| *24. Was the randomised intervention assignment concealed from both patients and health care staff until recruitment was complete and irrevocable?*  All non‐randomised studies should be answered no. If assignment was concealed from patients but not from staff, it should be answered no. |  | X |  | “The recruitment of consecutive patients in need of immediate rehabilitation care necessarily highlighted to the staff the differences in treatment between the groups”. |
| *25. Was there adequate adjustment for confounding in the analyses from which the main findings were drawn?*  This question should be answered no for trials if: the main conclusions of the study were based on analyses of treatment rather than intention to treat; the distribution of known confounders in the different treatment groups was  not described; or the distribution of known confounders differed between the treatment groups but was not taken into account in the analyses. In non-randomized studies if the effect of the main confounders was not investigated or confounding was demonstrated but no adjustment was made in the final analyses the question should be answered as no. | X |  |  | The confounders have been taken into account in the analysis |
| *26. Were losses of patients to follow‐up taken into account?*  If the numbers of patients lost to follow‐up are not reported, the question should be answered as unable to determine. If the proportion lost to follow-up was too small to affect the main findings, the question should be answered yes. | X |  |  | No patient dropped out |
| **Power** | **YES** | **NO** | **UTD** | **Notes/Justification** |
| *27. Did the study have sufficient power to detect a clinically important effect where the probability value for a difference being due to chance is less than 5%?*  Sample sizes have been calculated to detect a difference of x% and y%. |  | X |  | Power analysis has not been reported. |
| **Total Score 21/27 = 77.8 %** | | | | |

| **Pop et al. 2018** | | | | |
| --- | --- | --- | --- | --- |
| **Reporting** | **YES** | **NO** | **UTD** | **Notes/Justification** |
| *1. Is the hypothesis/aim/objective of the study clearly described?* | X |  |  | The aim is clearly described. |
| *2. Are the main outcomes to be measured clearly described in the Introduction or Methods section?*  If the main outcomes are first mentioned in the Results section, the question should be answered no. | X |  |  | Clearly described in the Introduction section. |
| *3. Are the characteristics of the patients included*  *in the study clearly described?*  In cohort studies and trials, inclusion and/or exclusion criteria should be given. In case‐control studies, a case‐definition and the source for controls should be given. | X |  |  | Clearly described in the Methods section. |
| *4. Are the interventions of interest clearly described?*  Treatments and placebo (where relevant) that are to be compared should be clearly described. |  |  |  |  |
| *5. Are the distributions of principal confounders in each group of subjects to be compared clearly described?*  A list of principal confounders is provided. | X |  |  | Reported in the Method section. |
| *6. Are the main findings of the study clearly described?*  Simple outcome data (including denominators and numerators) should be reported for all major findings so that the reader can check the major analyses and conclusions. (This question does not cover statistical tests  which are considered below). | X |  |  | Clearly reported in the Results section and Table 1,2,3,4,5. |
| *7. Does the study provide estimates of the random variability in the data for the main outcomes?*  In non-normally distributed data the inter‐quartile range of results should be reported. In normally distributed data the standard error, standard deviation or confidence intervals should be reported. If the distribution of the data is not described, it must be assumed that the estimates used were appropriate and the question should be answered yes. | X |  |  | Standard deviation has been reported. |
| *8. Have all important adverse events that may be a consequence of the intervention been reported?*  This should be answered yes if the study demonstrates that there was a comprehensive attempt to measure adverse events. (A list of possible adverse events is provided). |  |  |  |  |
| *9. Have the characteristics of patients lost to follow‐up been described?*  This should be answered yes where there were no losses to follow‐up or where losses to follow‐up were so small that findings would be unaffected by their inclusion. This should be answered NO where a study does not report the number of patients lost to follow‐up. |  |  |  |  |
| *10. Have actual probability values been reported ( e.g. 0.035 rather than < 0.05) for the main outcomes except where the probability value is less than 0.001?* | X |  |  | Actual probability values have been reported. |
| **External Validity** | **YES** | **NO** | **UTD** | **Notes/Justification** |
| *11. Were the subjects asked to participate in the study representative of the entire population from which they were recruited?*  The study must identify the source population for patients and describe how the patients were selected. Patients would be representative if they comprised the entire source population, an unselected sample of consecutive patients, or a random sample. Random sampling is only feasible where a list of all members of the relevant population exists. Where a study does not report the proportion of the source population from which the patients are derived, the question should be answered as unable to determine. |  |  | X | The proportion of the source population from which the patients are derived is not reported. |
| *12. Were those subjects who were prepared to participate representative of the entire population from which they were recruited?*  The proportion of those asked who agreed should be stated. Validation that the sample was representative would include demonstrating that the distribution of the main confounding factors was the same in the study sample and the source population. | X |  |  | The proportion is representative. |
| *13. Were the staff, places, and facilities where the patients were treated, representative of the treatment the majority of patients receive?*  For the question to be answered yes the study should demonstrate that the intervention was representative of that in use in the source population. The question should be answered no if, for example, the intervention was  undertaken in a specialist centre unrepresentative of the hospitals most of the source population would attend. |  |  |  |  |
| **Internal validity – bias** | **YES** | **NO** | **UTD** | **Notes/Justification** |
| *14. Was an attempt made to blind study subjects to the intervention they have received?*  For studies where the patients would have no way of knowing which intervention they received, this should be answered yes. |  |  |  |  |
| *15. Was an attempt made to blind those measuring the main outcomes of the intervention?* |  |  |  |  |
| *16. If any of the results of the study were based on “data dredging”, was this made clear?*  Any analyses that had not been planned at the outset of the study should be clearly indicated. If no retrospective unplanned subgroup analyses were reported, then answer yes. | X |  |  | No retrospective unplanned subgroup analyses were reported. |
| *17. In trials and cohort studies, do the analyses adjust for different lengths of follow‐up of patients, or in case-control studies, is the time period between the intervention and outcome the same for cases and controls?*  Where follow‐up was the same for all study patients the answer should yes.  If different lengths of follow‐up were adjusted for by, for example, survival analysis the answer should be yes. Studies where differences in follow‐up are ignored should be answered no. |  |  |  |  |
| *18. Were the statistical tests used to assess the main outcomes appropriate?*  The statistical techniques used must be appropriate to the data. For example, Non-parametric methods should be used for small sample sizes. Where little statistical analysis has been undertaken but where there is no evidence of bias, the question should be answered yes. If the distribution of the data (normal or not) is not described it must be assumed that the estimates used were appropriate and the question should be answered yes. | X |  |  | Statistics were appropriate and correctly reported. |
| *19. Was compliance with the intervention/s reliable?*  Where there was non compliance with the allocated treatment or where there was contamination of one group, the question should be answered no.  For studies where the effect of any misclassification was likely to bias any association to the null, the question should be answered yes. |  |  |  |  |
| *20. Were the main outcome measures used accurate (valid and reliable)?*  For studies where the outcome measures are clearly described, the question should be answered yes. For studies which refer to other work or that demonstrates the outcome measures are accurate, the question should be  answered as yes. | X |  |  | Outcome measures clearly described. |
| **Internal validity ‐ confounding (selection bias)** | **YES** | **NO** | **UTD** | **Notes/Justification** |
| *21. Were the patients in different intervention groups (trials and cohort studies) or were the cases and controls (case‐control studies) recruited from the same population?*  For example, patients for all comparison groups should be selected from the same hospital. The question should be answered unable to determine for cohort and case-control studies where there is no information concerning the source of patients included in the study. | X |  |  | Patients were recruited from the same clinic. |
| *22. Were study subjects in different intervention groups (trials and cohort- studies) or were the cases and controls (case‐control studies) recruited over the same period of time?*  For a study which does not specify the time period over which patients were recruited, the question should be answered as unable to determine. |  |  | X | The time period over which patients were recruited is not specified. |
| *23. Were study subjects randomised to intervention groups?*  Studies which state that subjects were randomized should be answered yes except where method of randomisation would not ensure random allocation.  For example, alternate allocation would score no because it is predictable. |  |  |  |  |
| *24. Was the randomised intervention assignment concealed from both patients and health care staff until recruitment was complete and irrevocable?*  All non‐randomised studies should be answered no. If assignment was concealed from patients but not from staff, it should be answered no. |  |  |  |  |
| *25. Was there adequate adjustment for confounding in the analyses from which the main findings were drawn?*  This question should be answered no for trials if: the main conclusions of the study were based on analyses of treatment rather than intention to treat; the distribution of known confounders in the different treatment groups was  not described; or the distribution of known confounders differed between the treatment groups but was not taken into account in the analyses. In non-randomized studies if the effect of the main confounders was not investigated or confounding was demonstrated but no adjustment was made in the final analyses the question should be answered as no. |  |  |  |  |
| *26. Were losses of patients to follow‐up taken into account?*  If the numbers of patients lost to follow‐up are not reported, the question should be answered as unable to determine. If the proportion lost to follow-up was too small to affect the main findings, the question should be  answered yes. |  |  |  |  |
| **Power** |  |  |  |  |
| *27. Did the study have sufficient power to detect a clinically important effect where the probability value for a difference being due to chance is less than 5%?*  Sample sizes have been calculated to detect a difference of x% and y%. |  |  |  |  |
| **Total Score 12/14 = 85.7 %** | | | | |

| **Quagliarella et al. 2011** | | | | |
| --- | --- | --- | --- | --- |
| **Reporting** | **YES** | **NO** | **UTD** | **Notes/Justification** |
| *1. Is the hypothesis/aim/objective of the study clearly described?* | X |  |  | The aim is clearly described. |
| *2. Are the main outcomes to be measured clearly described in the Introduction or Methods section?*  If the main outcomes are first mentioned in the Results section, the question should be answered no. | X |  |  | Clearly described in subjects and methods section (2.1-2.5 and table 1). |
| *3. Are the characteristics of the patients included in the study clearly described?*  In cohort studies and trials, inclusion and/or exclusion criteria should be given. In case‐control studies, a case‐definition and the source for controls should be given. | X |  |  | Clearly described in section 2.1 |
| *4. Are the interventions of interest clearly described?*  Treatments and placebo (where relevant) that are to be compared should be clearly described. |  |  |  |  |
| *5. Are the distributions of principal confounders in each group of subjects to be compared clearly described?*  A list of principal confounders is provided. |  | X |  | No lists of confounders have been provided. |
| *6. Are the main findings of the study clearly described?*  Simple outcome data (including denominators and numerators) should be reported for all major findings so that the reader can check the major analyses and conclusions. (This question does not cover statistical tests  which are considered below). | X |  |  | The main findings are described in results section with table 2 and figures1 and 2. |
| *7. Does the study provide estimates of the random variability in the data for the main outcomes?*  In non-normally distributed data the inter‐quartile range of results should be reported. In normally distributed data the standard error, standard deviation or confidence intervals should be reported. If the distribution of the data is not described, it must be assumed that the estimates used were appropriate and the question should be answered yes. | X |  |  | Variability has been reported. |
| *8. Have all important adverse events that may be a consequence of the intervention been reported?*  This should be answered yes if the study demonstrates that there was a comprehensive attempt to measure adverse events. (A list of possible adverse events is provided). |  |  |  |  |
| *9. Have the characteristics of patients lost to follow‐up been described?*  This should be answered yes where there were no losses to follow‐up or where losses to follow‐up were so small that findings would be unaffected by their inclusion. This should be answered NO where a study does not report the number of patients lost to follow‐up. |  |  |  |  |
| *10. Have actual probability values been reported (e.g. 0.035 rather than < 0.05) for the main outcomes except where the probability value is less than 0.001?* | X |  |  | Probability values have been reported. |
| **External Validity** | **YES** | **NO** | **UTD** | **Notes/Justification** |
| *11. Were the subjects asked to participate in the study representative of the entire population from which they were recruited?*  The study must identify the source population for patients and describe how the patients were selected. Patients would be representative if they comprised the entire source population, an unselected sample of consecutive patients, or a random sample. Random sampling is only feasible where a list of all members of the relevant population exists. Where a study does not report the proportion of the source population from which the patients are derived, the question should be answered as unable to determine. |  |  | X | The study does not report the proportion of the source population from which the patients are derived. |
| *12. Were those subjects who were prepared to participate representative of the entire population from which they were recruited?*  The proportion of those asked who agreed should be stated. Validation that the sample was representative would include demonstrating that the distribution of the main confounding factors was the same in the study sample and the source population. |  | X |  | No mention to the proportion of patient who agreed to participate to the study is present. |
| *13. Were the staff, places, and facilities where the patients were treated, representative of the treatment the majority of patients receive?*  For the question to be answered yes the study should demonstrate that the intervention was representative of that in use in the source population. The question should be answered no if, for example, the intervention was  undertaken in a specialist centre unrepresentative of the hospitals most of the source population would attend. |  |  |  |  |
| **Internal validity – bias** | **YES** | **NO** | **UTD** | **Notes/Justification** |
| *14. Was an attempt made to blind study subjects to the intervention they have received?*  For studies where the patients would have no way of knowing which intervention they received, this should be answered yes. |  |  |  |  |
| *15. Was an attempt made to blind those measuring the main outcomes of the intervention?* |  |  |  |  |
| *16. If any of the results of the study were based on “data dredging”, was this made clear?*  Any analyses that had not been planned at the outset of the study should be clearly indicated. If no retrospective unplanned subgroup analyses were reported, then answer yes. | X |  |  | No retrospective unplanned analyses were described. |
| *17. In trials and cohort studies, do the analyses adjust for different lengths of follow‐up of patients, or in case-control studies, is the time period between the intervention and outcome the same for cases and controls?*  Where follow‐up was the same for all study patients the answer should yes.  If different lengths of follow‐up were adjusted for by, for example, survival analysis the answer should be yes. Studies where differences in follow‐up are ignored should be answered no. |  |  |  |  |
| *18. Were the statistical tests used to assess the main outcomes appropriate?*  The statistical techniques used must be appropriate to the data. For example, Non-parametric methods should be used for small sample sizes. Where little statistical analysis has been undertaken but where there is no evidence of bias, the question should be answered yes. If the distribution of the data (normal or not) is not described it must be assumed that the estimates used were appropriate and the question should be answered yes. | X |  |  | Statistics were appropriate and correctly reported. |
| *19. Was compliance with the intervention/s reliable?*  Where there was no compliance with the allocated treatment or where there was contamination of one group, the question should be answered no.  For studies where the effect of any misclassification was likely to bias any association to the null, the question should be answered yes. |  |  |  |  |
| *20. Were the main outcome measures used accurate (valid and reliable)?*  For studies where the outcome measures are clearly described, the question should be answered yes. For studies which refer to other work or that demonstrates the outcome measures are accurate, the question should be  answered as yes. | X |  |  | Outcome measures were accurate. |
| **Internal validity ‐ confounding (selection bias)** | **YES** | **NO** | **UTD** | **Notes/Justification** |
| *21. Were the patients in different intervention groups (trials and cohort studies) or were the cases and controls (case‐control studies) recruited from the same population?*  For example, patients for all comparison groups should be selected from the same hospital. The question should be answered unable to determine for cohort and case-control studies where there is no information concerning the source of patients included in the study. | X |  |  |  |
| *22. Were study subjects in different intervention groups (trials and cohort- studies) or were the cases and controls (case‐control studies) recruited over the same period of time?*  For a study which does not specify the time period over which patients were recruited, the question should be answered as unable to determine. |  |  | X |  |
| *23. Were study subjects randomised to intervention groups?*  Studies which state that subjects were randomized should be answered yes except where method of randomisation would not ensure random allocation.  For example, alternate allocation would score no because it is predictable. |  |  |  |  |
| *24. Was the randomised intervention assignment concealed from both patients and health care staff until recruitment was complete and irrevocable?*  All non‐randomised studies should be answered no. If assignment was concealed from patients but not from staff, it should be answered no. |  |  |  |  |
| *25. Was there adequate adjustment for confounding in the analyses from which the main findings were drawn?*  This question should be answered no for trials if: the main conclusions of the study were based on analyses of treatment rather than intention to treat; the distribution of known confounders in the different treatment groups was  not described; or the distribution of known confounders differed between the treatment groups but was not taken into account in the analyses. In non-randomized studies if the effect of the main confounders was not investigated or confounding was demonstrated but no adjustment was made in the final analyses the question should be answered as no. |  |  |  |  |
| *26. Were losses of patients to follow‐up taken into account?*  If the numbers of patients lost to follow‐up are not reported, the question should be answered as unable to determine. If the proportion lost to follow-up was too small to affect the main findings, the question should be answered yes. |  |  |  |  |
| **Power** | **YES** | **NO** | **UTD** | **Notes/Justification** |
| *27. Did the study have sufficient power to detect a clinically important effect where the probability value for a difference being due to chance is less than 5%?*  Sample sizes have been calculated to detect a difference of x% and y%. |  |  |  |  |
| **Total Score 10/14 = 71.4** | | | | |

| **Queen et al. 2021** | | | | |
| --- | --- | --- | --- | --- |
| **Reporting** | **YES** | **NO** | **UTD** | **Notes/Justification** |
| *1. Is the hypothesis/aim/objective of the study clearly described?* | X |  |  | The aim is clearly described. |
| *2. Are the main outcomes to be measured clearly described in the Introduction or Methods section?*  If the main outcomes are first mentioned in the Results section, the question should be answered no. | X |  |  |  |
| *3. Are the characteristics of the patients included in the study clearly described?*  In cohort studies and trials, inclusion and/or exclusion criteria should be given. In case‐control studies, a case‐definition and the source for controls should be given. | X |  |  |  |
| *4. Are the interventions of interest clearly described?*  Treatments and placebo (where relevant) that are to be compared should be clearly described. |  |  |  |  |
| *5. Are the distributions of principal confounders in each group of subjects to be compared clearly described?*  A list of principal confounders is provided. | X |  |  |  |
| *6. Are the main findings of the study clearly described?*  Simple outcome data (including denominators and numerators) should be reported for all major findings so that the reader can check the major analyses and conclusions. (This question does not cover statistical tests  which are considered below). | X |  |  |  |
| *7. Does the study provide estimates of the random variability in the data for the main outcomes?*  In non-normally distributed data the inter‐quartile range of results should be reported. In normally distributed data the standard error, standard deviation or confidence intervals should be reported. If the distribution of the data is not described, it must be assumed that the estimates used were appropriate and the question should be answered yes. | X |  |  | Variability has been reported. |
| *8. Have all important adverse events that may be a consequence of the intervention been reported?*  This should be answered yes if the study demonstrates that there was a comprehensive attempt to measure adverse events. (A list of possible adverse events is provided). |  |  |  |  |
| *9. Have the characteristics of patients lost to follow‐up been described?*  This should be answered yes where there were no losses to follow‐up or where losses to follow‐up were so small that findings would be unaffected by their inclusion. This should be answered NO where a study does not report the number of patients lost to follow‐up. |  |  |  |  |
| *10. Have actual probability values been reported (e.g. 0.035 rather than < 0.05) for the main outcomes except where the probability value is less than 0.001?* | X |  |  | Probability values have been reported. |
| **External Validity** | **YES** | **NO** | **UTD** | **Notes/Justification** |
| *11. Were the subjects asked to participate in the study representative of the entire population from which they were recruited?*  The study must identify the source population for patients and describe how the patients were selected. Patients would be representative if they comprised the entire source population, an unselected sample of consecutive patients, or a random sample. Random sampling is only feasible where a list of all members of the relevant population exists. Where a study does not report the proportion of the source population from which the patients are derived, the question should be answered as unable to determine. |  |  | X | The study does not report the proportion of the source population from which the patients are derived. |
| *12. Were those subjects who were prepared to participate representative of the entire population from which they were recruited?*  The proportion of those asked who agreed should be stated. Validation that the sample was representative would include demonstrating that the distribution of the main confounding factors was the same in the study sample and the source population. |  |  | X | No mention to the proportion of patient who agreed to participate to the study is present. |
| *13. Were the staff, places, and facilities where the patients were treated, representative of the treatment the majority of patients receive?*  For the question to be answered yes the study should demonstrate that the intervention was representative of that in use in the source population. The question should be answered no if, for example, the intervention was  undertaken in a specialist centre unrepresentative of the hospitals most of the source population would attend. |  |  |  |  |
| **Internal validity – bias** | **YES** | **NO** | **UTD** | **Notes/Justification** |
| *14. Was an attempt made to blind study subjects to the intervention they have received?*  For studies where the patients would have no way of knowing which intervention they received, this should be answered yes. |  |  |  |  |
| *15. Was an attempt made to blind those measuring the main outcomes of the intervention?* |  |  |  |  |
| *16. If any of the results of the study were based on “data dredging”, was this made clear?*  Any analyses that had not been planned at the outset of the study should be clearly indicated. If no retrospective unplanned subgroup analyses were reported, then answer yes. | X |  |  | No retrospective unplanned analyses were described. |
| *17. In trials and cohort studies, do the analyses adjust for different lengths of follow‐up of patients, or in case-control studies, is the time period between the intervention and outcome the same for cases and controls?*  Where follow‐up was the same for all study patients the answer should yes.  If different lengths of follow‐up were adjusted for by, for example, survival analysis the answer should be yes. Studies where differences in follow‐up are ignored should be answered no. |  |  |  |  |
| *18. Were the statistical tests used to assess the main outcomes appropriate?*  The statistical techniques used must be appropriate to the data. For example, Non-parametric methods should be used for small sample sizes. Where little statistical analysis has been undertaken but where there is no evidence of bias, the question should be answered yes. If the distribution of the data (normal or not) is not described it must be assumed that the estimates used were appropriate and the question should be answered yes. | X |  |  | Statistics were appropriate and correctly reported. |
| *19. Was compliance with the intervention/s reliable?*  Where there was no compliance with the allocated treatment or where there was contamination of one group, the question should be answered no.  For studies where the effect of any misclassification was likely to bias any association to the null, the question should be answered yes. |  |  |  |  |
| *20. Were the main outcome measures used accurate (valid and reliable)?*  For studies where the outcome measures are clearly described, the question should be answered yes. For studies which refer to other work or that demonstrates the outcome measures are accurate, the question should be  answered as yes. | X |  |  | Outcome measures were accurate. |
| **Internal validity ‐ confounding (selection bias)** | **YES** | **NO** | **UTD** | **Notes/Justification** |
| *21. Were the patients in different intervention groups (trials and cohort studies) or were the cases and controls (case‐control studies) recruited from the same population?*  For example, patients for all comparison groups should be selected from the same hospital. The question should be answered unable to determine for cohort and case-control studies where there is no information concerning the source of patients included in the study. | X |  |  |  |
| *22. Were study subjects in different intervention groups (trials and cohort- studies) or were the cases and controls (case‐control studies) recruited over the same period of time?*  For a study which does not specify the time period over which patients were recruited, the question should be answered as unable to determine. | X |  |  |  |
| *23. Were study subjects randomised to intervention groups?*  Studies which state that subjects were randomized should be answered yes except where method of randomisation would not ensure random allocation.  For example, alternate allocation would score no because it is predictable. |  |  |  |  |
| *24. Was the randomised intervention assignment concealed from both patients and health care staff until recruitment was complete and irrevocable?*  All non‐randomised studies should be answered no. If assignment was concealed from patients but not from staff, it should be answered no. |  |  |  |  |
| *25. Was there adequate adjustment for confounding in the analyses from which the main findings were drawn?*  This question should be answered no for trials if: the main conclusions of the study were based on analyses of treatment rather than intention to treat; the distribution of known confounders in the different treatment groups was  not described; or the distribution of known confounders differed between the treatment groups but was not taken into account in the analyses. In non-randomized studies if the effect of the main confounders was not investigated or confounding was demonstrated but no adjustment was made in the final analyses the question should be answered as no. |  |  |  |  |
| *26. Were losses of patients to follow‐up taken into account?*  If the numbers of patients lost to follow‐up are not reported, the question should be answered as unable to determine. If the proportion lost to follow-up was too small to affect the main findings, the question should be answered yes. |  |  |  |  |
| **Power** | **YES** | **NO** | **UTD** | **Notes/Justification** |
| *27. Did the study have sufficient power to detect a clinically important effect where the probability value for a difference being due to chance is less than 5%?*  Sample sizes have been calculated to detect a difference of x% and y%. |  |  |  |  |
| **Total Score 12/14 = 85.7 %** | | | | |
| **Rasch et al. 2010** | | | | |
| **Reporting** | **YES** | **NO** | **UTD** | **Notes/Justification** |
| *1. Is the hypothesis/aim/objective of the study clearly described?* | X |  |  | The aim is clearly described. |
| *2. Are the main outcomes to be measured clearly described in the Introduction or Methods section?*  If the main outcomes are first mentioned in the Results section, the question should be answered no. | X |  |  | Outcomes are clearly described in the Methods section. |
| *3. Are the characteristics of the patients included in the study clearly described?*  In cohort studies and trials, inclusion and/or exclusion criteria should be given. In case‐control studies, a case‐definition and the source for controls should be given. | X |  |  | Clearly described in the Methods section. |
| *4. Are the interventions of interest clearly described?*  Treatments and placebo (where relevant) that are to be compared should be clearly described. |  |  |  |  |
| *5. Are the distributions of principal confounders in each group of subjects to be compared clearly described?*  A list of principal confounders is provided. |  | X |  | The study has only one group of patients. |
| *6. Are the main findings of the study clearly described?*  Simple outcome data (including denominators and numerators) should be reported for all major findings so that the reader can check the major analyses and conclusions. (This question does not cover statistical tests  which are considered below). | X |  |  | Outcome data have been reported. |
| *7. Does the study provide estimates of the random variability in the data for the main outcomes?*  In non-normally distributed data the inter‐quartile range of results should be reported. In normally distributed data the standard error, standard deviation or confidence intervals should be reported. If the distribution of the data is not described, it must be assumed that the estimates used were appropriate and the question should be answered yes. | X |  |  | Variability has been reported. |
| *8. Have all important adverse events that may be a consequence of the intervention been reported?*  This should be answered yes if the study demonstrates that there was a comprehensive attempt to measure adverse events. (A list of possible adverse events is provided). |  |  |  |  |
| *9. Have the characteristics of patients lost to follow‐up been described?*  This should be answered yes where there were no losses to follow‐up or where losses to follow‐up were so small that findings would be unaffected by their inclusion. This should be answered NO where a study does not report the number of patients lost to follow‐up. |  |  |  |  |
| *10. Have actual probability values been reported (e.g. 0.035 rather than < 0.05) for the main outcomes except where the probability value is less than 0.001?* | X |  |  | Probability values have been reported. |
| **External Validity** | **YES** | **NO** | **UTD** | **Notes/Justification** |
| *11. Were the subjects asked to participate in the study representative of the entire population from which they were recruited?*  The study must identify the source population for patients and describe how the patients were selected. Patients would be representative if they comprised the entire source population, an unselected sample of consecutive patients, or a random sample. Random sampling is only feasible where a list of all members of the relevant population exists. Where a study does not report the proportion of the source population from which the patients are derived, the question should be answered as unable to determine. |  |  | X | The source population has not been mentioned. |
| *12. Were those subjects who were prepared to participate representative of the entire population from which they were recruited?*  The proportion of those asked who agreed should be stated. Validation that the sample was representative would include demonstrating that the distribution of the main confounding factors was the same in the study sample and the source population. |  |  | X | The source population has not been mentioned. |
| *13. Were the staff, places, and facilities where the patients were treated, representative of the treatment the majority of patients receive?*  For the question to be answered yes the study should demonstrate that the intervention was representative of that in use in the source population. The question should be answered no if, for example, the intervention was  undertaken in a specialist centre unrepresentative of the hospitals most of the source population would attend. |  |  |  |  |
| **Internal validity – bias** | **YES** | **NO** | **UTD** | **Notes/Justification** |
| *14. Was an attempt made to blind study subjects to the intervention they have received?*  For studies where the patients would have no way of knowing which intervention they received, this should be answered yes. |  |  |  |  |
| *15. Was an attempt made to blind those measuring the main outcomes of the intervention?* |  |  |  |  |
| *16. If any of the results of the study were based on “data dredging”, was this made clear?*  Any analyses that had not been planned at the outset of the study should be clearly indicated. If no retrospective unplanned subgroup analyses were reported, then answer yes. | X |  |  | No retrospective analysis have been reported. |
| *17. In trials and cohort studies, do the analyses adjust for different lengths of follow‐up of patients, or in case-control studies, is the time period between the intervention and outcome the same for cases and controls?*  Where follow‐up was the same for all study patients the answer should yes.  If different lengths of follow‐up were adjusted for by, for example, survival analysis the answer should be yes. Studies where differences in follow‐up are ignored should be answered no. |  |  |  |  |
| *18. Were the statistical tests used to assess the main outcomes appropriate?*  The statistical techniques used must be appropriate to the data. For example, Non-parametric methods should be used for small sample sizes. Where little statistical analysis has been undertaken but where there is no evidence of bias, the question should be answered yes. If the distribution of the data (normal or not) is not described it must be assumed that the estimates used were appropriate and the question should be answered yes. | X |  |  | Statistics are appropiate. |
| *19. Was compliance with the intervention/s reliable?*  Where there was non compliance with the allocated treatment or where there was contamination of one group, the question should be answered no.  For studies where the effect of any misclassification was likely to bias any association to the null, the question should be answered yes. |  |  |  |  |
| *20. Were the main outcome measures used accurate (valid and reliable)?*  For studies where the outcome measures are clearly described, the question should be answered yes. For studies which refer to other work or that demonstrates the outcome measures are accurate, the question should be  answered as yes. | X |  |  | Outcome measures are accurate. |
| **Internal validity ‐ confounding (selection bias)** | **YES** | **NO** | **UTD** | **Notes/Justification** |
| *21. Were the patients in different intervention groups (trials and cohort studies) or were the cases and controls (case‐control studies) recruited from the same population?*  For example, patients for all comparison groups should be selected from the same hospital. The question should be answered unable to determine for cohort and case-control studies where there is no information concerning the source of patients included in the study. |  |  | X | The source population has not been mentioned. |
| *22. Were study subjects in different intervention groups (trials and cohort- studies) or were the cases and controls (case‐control studies) recruited over the same period of time?*  For a study which does not specify the time period over which patients were recruited, the question should be answered as unable to determine. |  |  | X | The study has not a control group. |
| *23. Were study subjects randomised to intervention groups?*  Studies which state that subjects were randomized should be answered yes except where method of randomisation would not ensure random allocation.  For example, alternate allocation would score no because it is predictable. |  |  |  |  |
| *24. Was the randomised intervention assignment concealed from both patients and health care staff until recruitment was complete and irrevocable?*  All non‐randomised studies should be answered no. If assignment was concealed from patients but not from staff, it should be answered no. |  |  |  |  |
| *25. Was there adequate adjustment for confounding in the analyses from which the main findings were drawn?*  This question should be answered no for trials if: the main conclusions of the study were based on analyses of treatment rather than intention to treat; the distribution of known confounders in the different treatment groups was  not described; or the distribution of known confounders differed between the treatment groups but was not taken into account in the analyses. In non-randomized studies if the effect of the main confounders was not investigated or confounding was demonstrated but no adjustment was made in the final analyses the question should be answered as no. |  |  |  |  |
| *26. Were losses of patients to follow‐up taken into account?*  If the numbers of patients lost to follow‐up are not reported, the question should be answered as unable to determine. If the proportion lost to follow-up was too small to affect the main findings, the question should be answered yes. |  |  |  |  |
| **Power** | **YES** | **NO** | **UTD** | **Notes/Justification** |
| *27. Did the study have sufficient power to detect a clinically important effect where the probability value for a difference being due to chance is less than 5%?*  Sample sizes have been calculated to detect a difference of x% and y%. |  |  |  |  |
| **Total Score 9/14 = 64.2 %** | | | | |

| **Rougier et al. 2008** | | | | |
| --- | --- | --- | --- | --- |
| **Reporting** | **YES** | **NO** | **UTD** | **Notes/Justification** |
| *1. Is the hypothesis/aim/objective of the study clearly described?* | X |  |  | The aim is described. |
| *2. Are the main outcomes to be measured clearly described in the Introduction or Methods section?*  If the main outcomes are first mentioned in the Results section, the question should be answered no. |  | X |  | The authors mention the methodology reported in previous articles (Rougier &Caron, 2000; Genthon & Rougier, 2005). Only the main points are discussed in the method section. |
| *3. Are the characteristics of the patients included in the study clearly described?*  In cohort studies and trials, inclusion and/or exclusion criteria should be given. In case‐control studies, a case‐definition and the source for controls should be given. | X |  |  | Yes, characteristics of cases and controls are described in the section “experimental Procedure”. |
| *4. Are the interventions of interest clearly described?*  Treatments and placebo (where relevant) that are to be compared should be clearly described. |  |  |  |  |
| *5. Are the distributions of principal confounders in each group of subjects to be compared clearly described?*  A list of principal confounders is provided. |  | X |  | Fourteen patients undergoing surgery for total hip arthroplasty because osteoarthritis without controlateral impairment were included and compared with 13 healthy adults of similar age ranges and morphologic characteristics,  These reference subjects were required to adopt an asymmetrical body weight distribution close to that observed on average for the patients. To this aim, the healthy subjects performed a preliminary trial run during which oral feedback on body weight distribution on both legs was provided. So there is an attempt to minimize confounders but they aren’t clearly listed. |
| *6. Are the main findings of the study clearly described?*  Simple outcome data (including denominators and numerators) should be reported for all major findings so that the reader can check the major analyses and conclusions. (This question does not cover statistical tests  which are considered below). | X |  |  | Yes they are described in the results section. |
| *7. Does the study provide estimates of the random variability in the data for the main outcomes?*  In non-normally distributed data the inter‐quartile range of results should be reported. In normally distributed data the standard error, standard deviation or confidence intervals should be reported. If the distribution of the data is not described, it must be assumed that the estimates used were appropriate and the question should be answered yes. | X |  |  | Yes, SD is provided |
| *8. Have all important adverse events that may be a consequence of the intervention been reported?*  This should be answered yes if the study demonstrates that there was a comprehensive attempt to measure adverse events. (A list of possible adverse events is provided). |  |  |  |  |
| *9. Have the characteristics of patients lost to follow‐up been described?*  This should be answered yes where there were no losses to follow‐up or where losses to follow‐up were so small that findings would be unaffected by their inclusion. This should be answered NO where a study does not report the number of patients lost to follow‐up. |  |  |  |  |
| *10. Have actual probability values been reported (e.g. 0.035 rather than < 0.05) for the main outcomes except where the probability value is less than 0.001?* | X |  |  | Probability values have been reported. |
| **External Validity** | **YES** | **NO** | **UTD** | **Notes/Justification** |
| *11. Were the subjects asked to participate in the study representative of the entire population from which they were recruited?*  The study must identify the source population for patients and describe how the patients were selected. Patients would be representative if they comprised the entire source population, an unselected sample of consecutive patients, or a random sample. Random sampling is only feasible where a list of all members of the relevant population exists. Where a study does not report the proportion of the source population from which the patients are derived, the question should be answered as unable to determine. |  |  | X | The source population is not described, nor is described the process of selection. |
| *12. Were those subjects who were prepared to participate representative of the entire population from which they were recruited?*  The proportion of those asked who agreed should be stated. Validation that the sample was representative would include demonstrating that the distribution of the main confounding factors was the same in the study sample and the source population. |  |  | X | The source population is not described. |
| *13. Were the staff, places, and facilities where the patients were treated, representative of the treatment the majority of patients receive?*  For the question to be answered yes the study should demonstrate that the intervention was representative of that in use in the source population. The question should be answered no if, for example, the intervention was  undertaken in a specialist centre unrepresentative of the hospitals most of the source population would attend. |  |  |  |  |
| **Internal validity – bias** | **YES** | **NO** | **UTD** | **Notes/Justification** |
| *14. Was an attempt made to blind study subjects to the intervention they have received?*  For studies where the patients would have no way of knowing which intervention they received, this should be answered yes. |  |  |  |  |
| *15. Was an attempt made to blind those measuring the main outcomes of the intervention?* |  |  |  |  |
| *16. If any of the results of the study were based on “data dredging”, was this made clear?*  Any analyses that had not been planned at the outset of the study should be clearly indicated. If no retrospective unplanned subgroup analyses were reported, then answer yes. | X |  |  | No dredging data is mentioned. |
| *17. In trials and cohort studies, do the analyses adjust for different lengths of follow‐up of patients, or in case-control studies, is the time period between the intervention and outcome the same for cases and controls?*  Where follow‐up was the same for all study patients the answer should yes.  If different lengths of follow‐up were adjusted for by, for example, survival analysis the answer should be yes. Studies where differences in follow‐up are ignored should be answered no. |  |  |  |  |
| *18. Were the statistical tests used to assess the main outcomes appropriate?*  The statistical techniques used must be appropriate to the data. For example, Non-parametric methods should be used for small sample sizes. Where little statistical analysis has been undertaken but where there is no evidence of bias, the question should be answered yes. If the distribution of the data (normal or not) is not described it must be assumed that the estimates used were appropriate and the question should be answered yes. | X |  |  | Statistics were appropriate and correctly reported. |
| *19. Was compliance with the intervention/s reliable?*  Where there was no compliance with the allocated treatment or where there was contamination of one group, the question should be answered no.  For studies where the effect of any misclassification was likely to bias any association to the null, the question should be answered yes. |  |  |  |  |
| *20. Were the main outcome measures used accurate (valid and reliable)?*  For studies where the outcome measures are clearly described, the question should be answered yes. For studies which refer to other work or that demonstrates the outcome measures are accurate, the question should be  answered as yes. | X |  |  | Outcome measures were accurate. |
| **Internal validity ‐ confounding (selection bias)** | **YES** | **NO** | **UTD** | **Notes/Justification** |
| *21. Were the patients in different intervention groups (trials and cohort studies) or were the cases and controls (case‐control studies) recruited from the same population?*  For example, patients for all comparison groups should be selected from the same hospital. The question should be answered unable to determine for cohort and case-control studies where there is no information concerning the source of patients included in the study. |  |  | X | The population from which patients were recruited is not clearly described. |
| *22. Were study subjects in different intervention groups (trials and cohort- studies) or were the cases and controls (case‐control studies) recruited over the same period of time?*  For a study which does not specify the time period over which patients were recruited, the question should be answered as unable to determine. |  |  | X | It has not been described. |
| *23. Were study subjects randomised to intervention groups?*  Studies which state that subjects were randomized should be answered yes except where method of randomisation would not ensure random allocation.  For example, alternate allocation would score no because it is predictable. |  |  |  |  |
| *24. Was the randomised intervention assignment concealed from both patients and health care staff until recruitment was complete and irrevocable?*  All non‐randomised studies should be answered no. If assignment was concealed from patients but not from staff, it should be answered no. |  |  |  |  |
| *25. Was there adequate adjustment for confounding in the analyses from which the main findings were drawn?*  This question should be answered no for trials if: the main conclusions of the study were based on analyses of treatment rather than intention to treat; the distribution of known confounders in the different treatment groups was  not described; or the distribution of known confounders differed between the treatment groups but was not taken into account in the analyses. In non-randomized studies if the effect of the main confounders was not investigated or confounding was demonstrated but no adjustment was made in the final analyses the question should be answered as no. |  |  |  |  |
| *26. Were losses of patients to follow‐up taken into account?*  If the numbers of patients lost to follow‐up are not reported, the question should be answered as unable to determine. If the proportion lost to follow-up was too small to affect the main findings, the question should be answered yes. |  |  |  |  |
| **Power** | **YES** | **NO** | **UTD** | **Notes/Justification** |
| *27. Did the study have sufficient power to detect a clinically important effect where the probability value for a difference being due to chance is less than 5%?*  Sample sizes have been calculated to detect a difference of x% and y%. |  |  |  |  |
| **Total Score 8/14 = 57.1** | | | | |

| **Shabana et al. 2021** | | | | |
| --- | --- | --- | --- | --- |
| **Reporting** | **YES** | **NO** | **UTD** | **Notes/Justification** |
| *1. Is the hypothesis/aim/objective of the study clearly described?* | X |  |  | The aim is described. |
| *2. Are the main outcomes to be measured clearly described in the Introduction or Methods section?*  If the main outcomes are first mentioned in the Results section, the question should be answered no. | X |  |  |  |
| *3. Are the characteristics of the patients included in the study clearly described?*  In cohort studies and trials, inclusion and/or exclusion criteria should be given. In case‐control studies, a case‐definition and the source for controls should be given. |  |  | X | Characteristics have been partially reported. For example it is not known if they had the same type of surgery, and which were the inclusion and exclusion criteria. |
| *4. Are the interventions of interest clearly described?*  Treatments and placebo (where relevant) that are to be compared should be clearly described. | X |  |  |  |
| *5. Are the distributions of principal confounders in each group of subjects to be compared clearly described?*  A list of principal confounders is provided. |  |  | X |  |
| *6. Are the main findings of the study clearly described?*  Simple outcome data (including denominators and numerators) should be reported for all major findings so that the reader can check the major analyses and conclusions. (This question does not cover statistical tests  which are considered below). | X |  |  | Yes they are described in the results section. |
| *7. Does the study provide estimates of the random variability in the data for the main outcomes?*  In non-normally distributed data the inter‐quartile range of results should be reported. In normally distributed data the standard error, standard deviation or confidence intervals should be reported. If the distribution of the data is not described, it must be assumed that the estimates used were appropriate and the question should be answered yes. | X |  |  | Yes, SD is provided |
| *8. Have all important adverse events that may be a consequence of the intervention been reported?*  This should be answered yes if the study demonstrates that there was a comprehensive attempt to measure adverse events. (A list of possible adverse events is provided). |  |  | X |  |
| *9. Have the characteristics of patients lost to follow‐up been described?*  This should be answered yes where there were no losses to follow‐up or where losses to follow‐up were so small that findings would be unaffected by their inclusion. This should be answered NO where a study does not report the number of patients lost to follow‐up. | X |  |  |  |
| *10. Have actual probability values been reported (e.g. 0.035 rather than < 0.05) for the main outcomes except where the probability value is less than 0.001?* | X |  |  | Probability values have been reported. |
| **External Validity** | **YES** | **NO** | **UTD** | **Notes/Justification** |
| *11. Were the subjects asked to participate in the study representative of the entire population from which they were recruited?*  The study must identify the source population for patients and describe how the patients were selected. Patients would be representative if they comprised the entire source population, an unselected sample of consecutive patients, or a random sample. Random sampling is only feasible where a list of all members of the relevant population exists. Where a study does not report the proportion of the source population from which the patients are derived, the question should be answered as unable to determine. |  |  | X  X |  |
| *12. Were those subjects who were prepared to participate representative of the entire population from which they were recruited?*  The proportion of those asked who agreed should be stated. Validation that the sample was representative would include demonstrating that the distribution of the main confounding factors was the same in the study sample and the source population. |  |  | X | The source population is not described. |
| *13. Were the staff, places, and facilities where the patients were treated, representative of the treatment the majority of patients receive?*  For the question to be answered yes the study should demonstrate that the intervention was representative of that in use in the source population. The question should be answered no if, for example, the intervention was  undertaken in a specialist centre unrepresentative of the hospitals most of the source population would attend. | X |  |  |  |
| **Internal validity – bias** | **YES** | **NO** | **UTD** | **Notes/Justification** |
| *14. Was an attempt made to blind study subjects to the intervention they have received?*  For studies where the patients would have no way of knowing which intervention they received, this should be answered yes. |  |  | X |  |
| *15. Was an attempt made to blind those measuring the main outcomes of the intervention?* |  |  |  |  |
| *16. If any of the results of the study were based on “data dredging”, was this made clear?*  Any analyses that had not been planned at the outset of the study should be clearly indicated. If no retrospective unplanned subgroup analyses were reported, then answer yes. | X |  |  | No dredging data is mentioned. |
| *17. In trials and cohort studies, do the analyses adjust for different lengths of follow‐up of patients, or in case-control studies, is the time period between the intervention and outcome the same for cases and controls?*  Where follow‐up was the same for all study patients the answer should yes.  If different lengths of follow‐up were adjusted for by, for example, survival analysis the answer should be yes. Studies where differences in follow‐up are ignored should be answered no. | X |  |  |  |
| *18. Were the statistical tests used to assess the main outcomes appropriate?*  The statistical techniques used must be appropriate to the data. For example, Non-parametric methods should be used for small sample sizes. Where little statistical analysis has been undertaken but where there is no evidence of bias, the question should be answered yes. If the distribution of the data (normal or not) is not described it must be assumed that the estimates used were appropriate and the question should be answered yes. | X |  |  | Statistics were appropriate and correctly reported. |
| *19. Was compliance with the intervention/s reliable?*  Where there was no compliance with the allocated treatment or where there was contamination of one group, the question should be answered no.  For studies where the effect of any misclassification was likely to bias any association to the null, the question should be answered yes. | X |  |  |  |
| *20. Were the main outcome measures used accurate (valid and reliable)?*  For studies where the outcome measures are clearly described, the question should be answered yes. For studies which refer to other work or that demonstrates the outcome measures are accurate, the question should be  answered as yes. | X |  |  | Outcome measures were accurate. |
| **Internal validity ‐ confounding (selection bias)** | **YES** | **NO** | **UTD** | **Notes/Justification** |
| *21. Were the patients in different intervention groups (trials and cohort studies) or were the cases and controls (case‐control studies) recruited from the same population?*  For example, patients for all comparison groups should be selected from the same hospital. The question should be answered unable to determine for cohort and case-control studies where there is no information concerning the source of patients included in the study. | X |  |  |  |
| *22. Were study subjects in different intervention groups (trials and cohort- studies) or were the cases and controls (case‐control studies) recruited over the same period of time?*  For a study which does not specify the time period over which patients were recruited, the question should be answered as unable to determine. | X |  |  | It has not been described. |
| *23. Were study subjects randomised to intervention groups?*  Studies which state that subjects were randomized should be answered yes except where method of randomisation would not ensure random allocation.  For example, alternate allocation would score no because it is predictable. |  |  | X |  |
| *24. Was the randomised intervention assignment concealed from both patients and health care staff until recruitment was complete and irrevocable?*  All non‐randomised studies should be answered no. If assignment was concealed from patients but not from staff, it should be answered no. |  |  | X |  |
| *25. Was there adequate adjustment for confounding in the analyses from which the main findings were drawn?*  This question should be answered no for trials if: the main conclusions of the study were based on analyses of treatment rather than intention to treat; the distribution of known confounders in the different treatment groups was  not described; or the distribution of known confounders differed between the treatment groups but was not taken into account in the analyses. In non-randomized studies if the effect of the main confounders was not investigated or confounding was demonstrated but no adjustment was made in the final analyses the question should be answered as no. |  |  | X |  |
| *26. Were losses of patients to follow‐up taken into account?*  If the numbers of patients lost to follow‐up are not reported, the question should be answered as unable to determine. If the proportion lost to follow-up was too small to affect the main findings, the question should be answered yes. | X |  |  |  |
| **Power** | **YES** | **NO** | **UTD** | **Notes/Justification** |
| *27. Did the study have sufficient power to detect a clinically important effect where the probability value for a difference being due to chance is less than 5%?*  Sample sizes have been calculated to detect a difference of x% and y%. |  |  | X |  |
| **Total Score 16/27 = 59.2** | | | | |

| **Slaven et al. 2012** | | | | |
| --- | --- | --- | --- | --- |
| **Reporting** | **YES** | **NO** | **UTD** | **Notes/Justification** |
| *1. Is the hypothesis/aim/objective of the study clearly described?* | X |  |  | The aim is clearly described. |
| *2. Are the main outcomes to be measured clearly described in the Introduction or Methods section?*  If the main outcomes are first mentioned in the Results section, the question should be answered no. | X |  |  | Outcomes are clearly described in the Methods section. |
| *3. Are the characteristics of the patients included in the study clearly described?*  In cohort studies and trials, inclusion and/or exclusion criteria should be given. In case‐control studies, a case‐definition and the source for controls should be given. | X |  |  | Clearly described in the Methods section. |
| *4. Are the interventions of interest clearly described?*  Treatments and placebo (where relevant) that are to be compared should be clearly described. |  |  |  |  |
| *5. Are the distributions of principal confounders in each group of subjects to be compared clearly described?*  A list of principal confounders is provided. |  | X |  | The study has only one group of patients. |
| *6. Are the main findings of the study clearly described?*  Simple outcome data (including denominators and numerators) should be reported for all major findings so that the reader can check the major analyses and conclusions. (This question does not cover statistical tests  which are considered below). | X |  |  | Outcome data have been reported. |
| *7. Does the study provide estimates of the random variability in the data for the main outcomes?*  In non-normally distributed data the inter‐quartile range of results should be reported. In normally distributed data the standard error, standard deviation or confidence intervals should be reported. If the distribution of the data is not described, it must be assumed that the estimates used were appropriate and the question should be answered yes. | X |  |  | Variability has been reported. |
| *8. Have all important adverse events that may be a consequence of the intervention been reported?*  This should be answered yes if the study demonstrates that there was a comprehensive attempt to measure adverse events. (A list of possible adverse events is provided). |  |  |  |  |
| *9. Have the characteristics of patients lost to follow‐up been described?*  This should be answered yes where there were no losses to follow‐up or where losses to follow‐up were so small that findings would be unaffected by their inclusion. This should be answered NO where a study does not report the number of patients lost to follow‐up. |  |  |  |  |
| *10. Have actual probability values been reported (e.g. 0.035 rather than < 0.05) for the main outcomes except where the probability value is less than 0.001?* | X |  |  | Probability values have been reported. |
| **External Validity** | **YES** | **NO** | **UTD** | **Notes/Justification** |
| *11. Were the subjects asked to participate in the study representative of the entire population from which they were recruited?*  The study must identify the source population for patients and describe how the patients were selected. Patients would be representative if they comprised the entire source population, an unselected sample of consecutive patients, or a random sample. Random sampling is only feasible where a list of all members of the relevant population exists. Where a study does not report the proportion of the source population from which the patients are derived, the question should be answered as unable to determine. | X |  |  | Subjects were representative. |
| *12. Were those subjects who were prepared to participate representative of the entire population from which they were recruited?*  The proportion of those asked who agreed should be stated. Validation that the sample was representative would include demonstrating that the distribution of the main confounding factors was the same in the study sample and the source population. | X |  |  | Subjects were representative. |
| *13. Were the staff, places, and facilities where the patients were treated, representative of the treatment the majority of patients receive?*  For the question to be answered yes the study should demonstrate that the intervention was representative of that in use in the source population. The question should be answered no if, for example, the intervention was  undertaken in a specialist centre unrepresentative of the hospitals most of the source population would attend. |  |  |  |  |
| **Internal validity – bias** | **YES** | **NO** | **UTD** | **Notes/Justification** |
| *14. Was an attempt made to blind study subjects to the intervention they have received?*  For studies where the patients would have no way of knowing which intervention they received, this should be answered yes. |  |  |  |  |
| *15. Was an attempt made to blind those measuring the main outcomes of the intervention?* |  |  |  |  |
| *16. If any of the results of the study were based on “data dredging”, was this made clear?*  Any analyses that had not been planned at the outset of the study should be clearly indicated. If no retrospective unplanned subgroup analyses were reported, then answer yes. | X |  |  | No data dredging was performed. |
| *17. In trials and cohort studies, do the analyses adjust for different lengths of follow‐up of patients, or in case-control studies, is the time period between the intervention and outcome the same for cases and controls?*  Where follow‐up was the same for all study patients the answer should yes.  If different lengths of follow‐up were adjusted for by, for example, survival analysis the answer should be yes. Studies where differences in follow‐up are ignored should be answered no. |  |  |  |  |
| *18. Were the statistical tests used to assess the main outcomes appropriate?*  The statistical techniques used must be appropriate to the data. For example, Non-parametric methods should be used for small sample sizes. Where little statistical analysis has been undertaken but where there is no evidence of bias, the question should be answered yes. If the distribution of the data (normal or not) is not described it must be assumed that the estimates used were appropriate and the question should be answered yes. | X |  |  | Statistics are appropriate. |
| *19. Was compliance with the intervention/s reliable?*  Where there was non compliance with the allocated treatment or where there was contamination of one group, the question should be answered no.  For studies where the effect of any misclassification was likely to bias any association to the null, the question should be answered yes. |  |  |  |  |
| *20. Were the main outcome measures used accurate (valid and reliable)?*  For studies where the outcome measures are clearly described, the question should be answered yes. For studies which refer to other work or that demonstrates the outcome measures are accurate, the question should be  answered as yes. | X |  |  | Outcome measures are accurate. |
| **Internal validity ‐ confounding (selection bias)** | **YES** | **NO** | **UTD** | **Notes/Justification** |
| *21. Were the patients in different intervention groups (trials and cohort studies) or were the cases and controls (case‐control studies) recruited from the same population?*  For example, patients for all comparison groups should be selected from the same hospital. The question should be answered unable to determine for cohort and case-control studies where there is no information concerning the source of patients included in the study. | X |  |  | Patients were recruited from the same population. |
| *22. Were study subjects in different intervention groups (trials and cohort- studies) or were the cases and controls (case‐control studies) recruited over the same period of time?*  For a study which does not specify the time period over which patients were recruited, the question should be answered as unable to determine. |  | X |  | The study has not a control group. |
| *23. Were study subjects randomised to intervention groups?*  Studies which state that subjects were randomized should be answered yes except where method of randomisation would not ensure random allocation.  For example, alternate allocation would score no because it is predictable. |  |  |  |  |
| *24. Was the randomised intervention assignment concealed from both patients and health care staff until recruitment was complete and irrevocable?*  All non‐randomised studies should be answered no. If assignment was concealed from patients but not from staff, it should be answered no. |  |  |  |  |
| *25. Was there adequate adjustment for confounding in the analyses from which the main findings were drawn?*  This question should be answered no for trials if: the main conclusions of the study were based on analyses of treatment rather than intention to treat; the distribution of known confounders in the different treatment groups was  not described; or the distribution of known confounders differed between the treatment groups but was not taken into account in the analyses. In non-randomized studies if the effect of the main confounders was not investigated or confounding was demonstrated but no adjustment was made in the final analyses the question should be answered as no. |  |  |  |  |
| *26. Were losses of patients to follow‐up taken into account?*  If the numbers of patients lost to follow‐up are not reported, the question should be answered as unable to determine. If the proportion lost to follow-up was too small to affect the main findings, the question should be answered yes. |  |  |  |  |
| **Power** | **YES** | **NO** | **UTD** | **Notes/Justification** |
| *27. Did the study have sufficient power to detect a clinically important effect where the probability value for a difference being due to chance is less than 5%?*  Sample sizes have been calculated to detect a difference of x% and y%. |  |  |  |  |
| **Total Score 12/14 = 85.7 %** | | | | |

| **Sliwinski et al. 2004** | | | | |
| --- | --- | --- | --- | --- |
| **Reporting** | **YES** | **NO** | **UTD** | **Notes/Justification** |
| *1. Is the hypothesis/aim/objective of the study clearly described?* | X |  |  | The aim, hypothesis and objective are clearly described in the introduction section. |
| *2. Are the main outcomes to be measured clearly described in the Introduction or Methods section?*  If the main outcomes are first mentioned in the Results section, the question should be answered no. | X |  |  | Clearly described in the introduction and methods section. |
| *3. Are the characteristics of the patients included*  *in the study clearly described?*  In cohort studies and trials, inclusion and/or exclusion criteria should be given. In case‐control studies, a case‐definition and the source for controls should be given. | X |  |  | Clearly described in the Methods section and Table 1. |
| *4. Are the interventions of interest clearly described?*  Treatments and placebo (where relevant) that are to be compared should be clearly described. |  |  |  |  |
| *5. Are the distributions of principal confounders in each group of subjects to be compared clearly described?*  A list of principal confounders is provided. |  | X |  | Confounders are not described, and a list is not provided. |
| *6. Are the main findings of the study clearly described?*  Simple outcome data (including denominators and numerators) should be reported for all major findings so that the reader can check the major analyses and conclusions. (This question does not cover statistical tests  which are considered below). | X |  |  | Reported in the Results section and Table 3. |
| *7. Does the study provide estimates of the random variability in the data for the main outcomes?*  In non-normally distributed data the inter‐quartile range of results should be reported. In normally distributed data the standard error, standard deviation or confidence intervals should be reported. If the distribution of the data is not described, it must be assumed that the estimates used were appropriate and the question should be answered yes. | X |  |  | Standard deviation has been reported. |
| *8. Have all important adverse events that may be a consequence of the intervention been reported?*  This should be answered yes if the study demonstrates that there was a comprehensive attempt to measure adverse events. (A list of possible adverse events is provided). |  |  |  |  |
| *9. Have the characteristics of patients lost to follow‐up been described?*  This should be answered yes where there were no losses to follow‐up or where losses to follow‐up were so small that findings would be unaffected by their inclusion. This should be answered NO where a study does not report the number of patients lost to follow‐up. |  |  |  |  |
| *10. Have actual probability values been reported ( e.g. 0.035 rather than < 0.05) for the main outcomes except where the probability value is less than 0.001?* | X |  |  | Probability values have been reported. |
| **External Validity** | **YES** | **NO** | **UTD** | **Notes/Justification** |
| *11. Were the subjects asked to participate in the study representative of the entire population from which they were recruited?*  The study must identify the source population for patients and describe how the patients were selected. Patients would be representative if they comprised the entire source population, an unselected sample of consecutive patients, or a random sample. Random sampling is only feasible where a list of all members of the relevant population exists. Where a study does not report the proportion of the source population from which the patients are derived, the question should be answered as unable to determine. |  |  | X | The proportion of the source population is not reported. |
| *12. Were those subjects who were prepared to participate representative of the entire population from which they were recruited?*  The proportion of those asked who agreed should be stated. Validation that the sample was representative would include demonstrating that the distribution of the main confounding factors was the same in the study sample and the source population. |  |  | X | It is not known if the distribution of the main confounding factors was the same in the study sample and the source population. |
| *13. Were the staff, places, and facilities where the patients were treated, representative of the treatment the majority of patients receive?*  For the question to be answered yes the study should demonstrate that the intervention was representative of that in use in the source population. The question should be answered no if, for example, the intervention was  undertaken in a specialist centre unrepresentative of the hospitals most of the source population would attend. |  |  |  |  |
| **Internal validity – bias** | **YES** | **NO** | **UTD** | **Notes/Justification** |
| *14. Was an attempt made to blind study subjects to the intervention they have received?*  For studies where the patients would have no way of knowing which intervention they received, this should be answered yes. |  |  |  |  |
| *15. Was an attempt made to blind those measuring the main outcomes of the intervention?* |  |  |  |  |
| *16. If any of the results of the study were based on “data dredging”, was this made clear?*  Any analyses that had not been planned at the outset of the study should be clearly indicated. If no retrospective unplanned subgroup analyses were reported, then answer yes. | X |  |  | No retrospective unplanned subgroup analyses were reported. |
| *17. In trials and cohort studies, do the analyses adjust for different lengths of follow‐up of patients, or in case-control studies, is the time period between the intervention and outcome the same for cases and controls?*  Where follow‐up was the same for all study patients the answer should yes.  If different lengths of follow‐up were adjusted for by, for example, survival analysis the answer should be yes. Studies where differences in follow‐up are ignored should be answered no. |  |  |  |  |
| *18. Were the statistical tests used to assess the main outcomes appropriate?*  The statistical techniques used must be appropriate to the data. For example, Non-parametric methods should be used for small sample sizes. Where little statistical analysis has been undertaken but where there is no evidence of bias, the question should be answered yes. If the distribution of the data (normal or not) is not described it must be assumed that the estimates used were appropriate and the question should be answered yes. | X |  |  | Statistics were appropriate and correctly reported. |
| *19. Was compliance with the intervention/s reliable?*  Where there was non compliance with the allocated treatment or where there was contamination of one group, the question should be answered no.  For studies where the effect of any misclassification was likely to bias any association to the null, the question should be answered yes. |  |  |  |  |
| *20. Were the main outcome measures used accurate (valid and reliable)?*  For studies where the outcome measures are clearly described, the question should be answered yes. For studies which refer to other work or that demonstrates the outcome measures are accurate, the question should be  answered as yes. | X |  |  | Outcome measures clearly described. |
| **Internal validity ‐ confounding (selection bias)** | **YES** | **NO** | **UTD** | **Notes/Justification** |
| *21. Were the patients in different intervention groups (trials and cohort studies) or were the cases and controls (case‐control studies) recruited from the same population?*  For example, patients for all comparison groups should be selected from the same hospital. The question should be answered unable to determine for cohort and case-control studies where there is no information concerning the source of patients included in the study. | X |  |  | Patients were recruited from the same hospital. |
| *22. Were study subjects in different intervention groups (trials and cohort- studies) or were the cases and controls (case‐control studies) recruited over the same period of time?*  For a study which does not specify the time period over which patients were recruited, the question should be answered as unable to determine. |  |  | X | The time period of recruitment is not specified. |
| *23. Were study subjects randomised to intervention groups?*  Studies which state that subjects were randomized should be answered yes except where method of randomisation would not ensure random allocation.  For example, alternate allocation would score no because it is predictable. |  |  |  |  |
| *24. Was the randomised intervention assignment concealed from both patients and health care staff until recruitment was complete and irrevocable?*  All non‐randomised studies should be answered no. If assignment was concealed from patients but not from staff, it should be answered no. |  |  |  |  |
| *25. Was there adequate adjustment for confounding in the analyses from which the main findings were drawn?*  This question should be answered no for trials if: the main conclusions of the study were based on analyses of treatment rather than intention to treat; the distribution of known confounders in the different treatment groups was  not described; or the distribution of known confounders differed between the treatment groups but was not taken into account in the analyses. In non-randomized studies if the effect of the main confounders was not investigated or confounding was demonstrated but no adjustment was made in the final analyses the question should be answered as no. |  |  |  |  |
| *26. Were losses of patients to follow‐up taken into account?*  If the numbers of patients lost to follow‐up are not reported, the question should be answered as unable to determine. If the proportion lost to follow-up was too small to affect the main findings, the question should be  answered yes. |  |  |  |  |
| **Power** |  |  |  |  |
| *27. Did the study have sufficient power to detect a clinically important effect where the probability value for a difference being due to chance is less than 5%?*  Sample sizes have been calculated to detect a difference of x% and y%. |  |  |  |  |
| **Total Score 10/14 = 71,4 %** | | | | |

| **Szymanski et al. 2012** | | | | |
| --- | --- | --- | --- | --- |
| **Reporting** | **YES** | **NO** | **UTD** | **Notes/Justification** |
| *1. Is the hypothesis/aim/objective of the study clearly described?* | X |  |  | The aim is clearly described. |
| *2. Are the main outcomes to be measured clearly described in the Introduction or Methods section?*  If the main outcomes are first mentioned in the Results section, the question should be answered no. | X |  |  | Clearly described in the Patients and Methods section. |
| *3. Are the characteristics of the patients included in the study clearly described?*  In cohort studies and trials, inclusion and/or exclusion criteria should be given. In case‐control studies, a case‐definition and the source for controls should be given. | X |  |  | Characteristics of the patient are clearly described in the Methods section. |
| *4. Are the interventions of interest clearly described?*  Treatments and placebo (where relevant) that are to be compared should be clearly described. |  |  |  |  |
| *5. Are the distributions of principal confounders in each group of subjects to be compared clearly described?*  A list of principal confounders is provided. | X |  |  | A list of the principal confounders is provided |
| *6. Are the main findings of the study clearly described?*  Simple outcome data (including denominators and numerators) should be reported for all major findings so that the reader can check the major analyses and conclusions. (This question does not cover statistical tests  which are considered below). | X |  |  | The main findings of the study are clearly described |
| *7. Does the study provide estimates of the random variability in the data for the main outcomes?*  In non-normally distributed data the inter‐quartile range of results should be reported. In normally distributed data the standard error, standard deviation or confidence intervals should be reported. If the distribution of the data is not described, it must be assumed that the estimates used were appropriate and the question should be answered yes. | X |  |  | The distribution of the data has not been reported and we assume that the estimates used were appropriate |
| *8. Have all important adverse events that may be a consequence of the intervention been reported?*  This should be answered yes if the study demonstrates that there was a comprehensive attempt to measure adverse events. (A list of possible adverse events is provided). |  |  |  |  |
| *9. Have the characteristics of patients lost to follow‐up been described?*  This should be answered yes where there were no losses to follow‐up or where losses to follow‐up were so small that findings would be unaffected by their inclusion. This should be answered NO where a study does not report the number of patients lost to follow‐up. |  |  |  |  |
| *10. Have actual probability values been reported (e.g. 0.035 rather than < 0.05) for the main outcomes except where the probability value is less than 0.001?* | X |  |  | Probability values have been reported. |
| **External Validity** | **YES** | **NO** | **UTD** | **Notes/Justification** |
| *11. Were the subjects asked to participate in the study representative of the entire population from which they were recruited?*  The study must identify the source population for patients and describe how the patients were selected. Patients would be representative if they comprised the entire source population, an unselected sample of consecutive patients, or a random sample. Random sampling is only feasible where a list of all members of the relevant population exists. Where a study does not report the proportion of the source population from which the patients are derived, the question should be answered as unable to determine. |  |  | X | The study does not report the proportion of the source population from which the patients are derived |
| *12. Were those subjects who were prepared to participate representative of the entire population from which they were recruited?*  The proportion of those asked who agreed should be stated. Validation that the sample was representative would include demonstrating that the distribution of the main confounding factors was the same in the study sample and the source population. |  | X |  | The study does not report the proportion and characteristic of the source population from which the patients are derived. |
| *13. Were the staff, places, and facilities where the patients were treated, representative of the treatment the majority of patients receive?*  For the question to be answered yes the study should demonstrate that the intervention was representative of that in use in the source population. The question should be answered no if, for example, the intervention was  undertaken in a specialist centre unrepresentative of the hospitals most of the source population would attend. |  |  |  |  |
| **Internal validity – bias** | **YES** | **NO** | **UTD** | **Notes/Justification** |
| *14. Was an attempt made to blind study subjects to the intervention they have received?*  For studies where the patients would have no way of knowing which intervention they received, this should be answered yes. |  |  |  |  |
| *15. Was an attempt made to blind those measuring the main outcomes of the intervention?* |  |  |  |  |
| *16. If any of the results of the study were based on “data dredging”, was this made clear?*  Any analyses that had not been planned at the outset of the study should be clearly indicated. If no retrospective unplanned subgroup analyses were reported, then answer yes. | X |  |  | No retrospective unplanned subgroup analyses were reported. |
| *17. In trials and cohort studies, do the analyses adjust for different lengths of follow‐up of patients, or in case-control studies, is the time period between the intervention and outcome the same for cases and controls?*  Where follow‐up was the same for all study patients the answer should yes.  If different lengths of follow‐up were adjusted for by, for example, survival analysis the answer should be yes. Studies where differences in follow‐up are ignored should be answered no. |  |  |  |  |
| *18. Were the statistical tests used to assess the main outcomes appropriate?*  The statistical techniques used must be appropriate to the data. For example, Non-parametric methods should be used for small sample sizes. Where little statistical analysis has been undertaken but where there is no evidence of bias, the question should be answered yes. If the distribution of the data (normal or not) is not described it must be assumed that the estimates used were appropriate and the question should be answered yes. | X |  |  | Statistics were appropriate and correctly reported. |
| *19. Was compliance with the intervention/s reliable?*  Where there was non compliance with the allocated treatment or where there was contamination of one group, the question should be answered no.  For studies where the effect of any misclassification was likely to bias any association to the null, the question should be answered yes. |  |  |  |  |
| *20. Were the main outcome measures used accurate (valid and reliable)?*  For studies where the outcome measures are clearly described, the question should be answered yes. For studies which refer to other work or that demonstrates the outcome measures are accurate, the question should be  answered as yes. | X |  |  | Outcome measures were accurate. |
| **Internal validity ‐ confounding (selection bias)** | **YES** | **NO** | **UTD** | **Notes/Justification** |
| *21. Were the patients in different intervention groups (trials and cohort studies) or were the cases and controls (case‐control studies) recruited from the same population?*  For example, patients for all comparison groups should be selected from the same hospital. The question should be answered unable to determine for cohort and case-control studies where there is no information concerning the source of patients included in the study. |  |  | X | A cohort of patients was recruited from a University Hospital while for the others cohorts there is no specification about the source. |
| *22. Were study subjects in different intervention groups (trials and cohort- studies) or were the cases and controls (case‐control studies) recruited over the same period of time?*  For a study which does specify the time period over which patients were recruited, the question should be answered as unable to determine. | X |  |  | The patients were recruited during a specified period of time |
| *23. Were study subjects randomised to intervention groups?*  Studies which state that subjects were randomized should be answered yes except where method of randomisation would not ensure random allocation.  For example, alternate allocation would score no because it is predictable. |  |  |  |  |
| *24. Was the randomised intervention assignment concealed from both patients and health care staff until recruitment was complete and irrevocable?*  All non‐randomised studies should be answered no. If assignment was concealed from patients but not from staff, it should be answered no. |  |  |  |  |
| *25. Was there adequate adjustment for confounding in the analyses from which the main findings were drawn?*  This question should be answered no for trials if: the main conclusions of the study were based on analyses of treatment rather than intention to treat; the distribution of known confounders in the different treatment groups was  not described; or the distribution of known confounders differed between the treatment groups but was not taken into account in the analyses. In non-randomized studies if the effect of the main confounders was not investigated or confounding was demonstrated but no adjustment was made in the final analyses the question should be answered as no. |  |  |  |  |
| *26. Were losses of patients to follow‐up taken into account?*  If the numbers of patients lost to follow‐up are not reported, the question should be answered as unable to determine. If the proportion lost to follow-up was too small to affect the main findings, the question should be answered yes. |  |  |  |  |
| **Power** | **YES** | **NO** | **UTD** | **Notes/Justification** |
| *27. Did the study have sufficient power to detect a clinically important effect where the probability value for a difference being due to chance is less than 5%?*  Sample sizes have been calculated to detect a difference of x% and y%. |  |  |  |  |
| **Total Score 11/14 = 78.6 %** | | | | |

| **Talis et al. 2007** | | | | |
| --- | --- | --- | --- | --- |
| **Reporting** | **YES** | **NO** | **UTD** | **Notes/Justification** |
| *1. Is the hypothesis/aim/objective of the study clearly described?* | X |  |  | The aim is clearly described. |
| *2. Are the main outcomes to be measured clearly described in the Introduction or Methods section?*  If the main outcomes are first mentioned in the Results section, the question should be answered no. | X |  |  | Clearly described in the Methods section. |
| *3. Are the characteristics of the patients included*  *in the study clearly described?*  In cohort studies and trials, inclusion and/or exclusion criteria should be given. In case‐control studies, a case‐definition and the source for controls should be given. | X |  |  | Clearly described in the Methods section and Table 1. |
| *4. Are the interventions of interest clearly described?*  Treatments and placebo (where relevant) that are to be compared should be clearly described. |  |  |  |  |
| *5. Are the distributions of principal confounders in each group of subjects to be compared clearly described?*  A list of principal confounders is provided. | X |  |  | Reported in the Methods section. |
| *6. Are the main findings of the study clearly described?*  Simple outcome data (including denominators and numerators) should be reported for all major findings so that the reader can check the major analyses and conclusions. (This question does not cover statistical tests  which are considered below). | X |  |  | Reported in the Results section and Tabls. |
| *7. Does the study provide estimates of the random variability in the data for the main outcomes?*  In non-normally distributed data the inter‐quartile range of results should be reported. In normally distributed data the standard error, standard deviation or confidence intervals should be reported. If the distribution of the data is not described, it must be assumed that the estimates used were appropriate and the question should be answered yes. | X |  |  | Standard deviation has been reported. |
| *8. Have all important adverse events that may be a consequence of the intervention been reported?*  This should be answered yes if the study demonstrates that there was a comprehensive attempt to measure adverse events. (A list of possible adverse events is provided). |  |  |  |  |
| *9. Have the characteristics of patients lost to follow‐up been described?*  This should be answered yes where there were no losses to follow‐up or where losses to follow‐up were so small that findings would be unaffected by their inclusion. This should be answered NO where a study does not report the number of patients lost to follow‐up. |  |  |  |  |
| *10. Have actual probability values been reported ( e.g. 0.035 rather than < 0.05) for the main outcomes except where the probability value is less than 0.001?* | X |  |  | Probability values have been reported. |
| **External Validity** | **YES** | **NO** | **UTD** | **Notes/Justification** |
| *11. Were the subjects asked to participate in the study representative of the entire population from which they were recruited?*  The study must identify the source population for patients and describe how the patients were selected. Patients would be representative if they comprised the entire source population, an unselected sample of consecutive patients, or a random sample. Random sampling is only feasible where a list of all members of the relevant population exists. Where a study does not report the proportion of the source population from which the patients are derived, the question should be answered as unable to determine. | X |  |  | Participants were representative. |
| *12. Were those subjects who were prepared to participate representative of the entire population from which they were recruited?*  The proportion of those asked who agreed should be stated. Validation that the sample was representative would include demonstrating that the distribution of the main confounding factors was the same in the study sample and the source population. | X |  |  | Population was representative. |
| *13. Were the staff, places, and facilities where the patients were treated, representative of the treatment the majority of patients receive?*  For the question to be answered yes the study should demonstrate that the intervention was representative of that in use in the source population. The question should be answered no if, for example, the intervention was  undertaken in a specialist centre unrepresentative of the hospitals most of the source population would attend. |  |  |  |  |
| **Internal validity – bias** | **YES** | **NO** | **UTD** | **Notes/Justification** |
| *14. Was an attempt made to blind study subjects to the intervention they have received?*  For studies where the patients would have no way of knowing which intervention they received, this should be answered yes. |  |  |  |  |
| *15. Was an attempt made to blind those measuring the main outcomes of the intervention?* |  |  |  |  |
| *16. If any of the results of the study were based on “data dredging”, was this made clear?*  Any analyses that had not been planned at the outset of the study should be clearly indicated. If no retrospective unplanned subgroup analyses were reported, then answer yes. | X |  |  | No retrospective unplanned subgroup analyses were reported. |
| *17. In trials and cohort studies, do the analyses adjust for different lengths of follow‐up of patients, or in case-control studies, is the time period between the intervention and outcome the same for cases and controls?*  Where follow‐up was the same for all study patients the answer should yes.  If different lengths of follow‐up were adjusted for by, for example, survival analysis the answer should be yes. Studies where differences in follow‐up are ignored should be answered no. |  |  |  |  |
| *18. Were the statistical tests used to assess the main outcomes appropriate?*  The statistical techniques used must be appropriate to the data. For example, Non-parametric methods should be used for small sample sizes. Where little statistical analysis has been undertaken but where there is no evidence of bias, the question should be answered yes. If the distribution of the data (normal or not) is not described it must be assumed that the estimates used were appropriate and the question should be answered yes. | X |  |  | Statistics were appropriate and correctly reported. |
| *19. Was compliance with the intervention/s reliable?*  Where there was non compliance with the allocated treatment or where there was contamination of one group, the question should be answered no.  For studies where the effect of any misclassification was likely to bias any association to the null, the question should be answered yes. |  |  |  |  |
| *20. Were the main outcome measures used accurate (valid and reliable)?*  For studies where the outcome measures are clearly described, the question should be answered yes. For studies which refer to other work or that demonstrates the outcome measures are accurate, the question should be  answered as yes. | X |  |  | Outcome measures clearly described. |
| **Internal validity ‐ confounding (selection bias)** | **YES** | **NO** | **UTD** | **Notes/Justification** |
| *21. Were the patients in different intervention groups (trials and cohort studies) or were the cases and controls (case‐control studies) recruited from the same population?*  For example, patients for all comparison groups should be selected from the same hospital. The question should be answered unable to determine for cohort and case-control studies where there is no information concerning the source of patients included in the study. | X |  |  | Patients were recruited from the same hospital. |
| *22. Were study subjects in different intervention groups (trials and cohort- studies) or were the cases and controls (case‐control studies) recruited over the same period of time?*  For a study which does not specify the time period over which patients were recruited, the question should be answered as unable to determine. |  |  | X | The time period through which patients were recruited is not reported. |
| *23. Were study subjects randomised to intervention groups?*  Studies which state that subjects were randomized should be answered yes except where method of randomisation would not ensure random allocation.  For example, alternate allocation would score no because it is predictable. |  |  |  |  |
| *24. Was the randomised intervention assignment concealed from both patients and health care staff until recruitment was complete and irrevocable?*  All non‐randomised studies should be answered no. If assignment was concealed from patients but not from staff, it should be answered no. |  |  |  |  |
| *25. Was there adequate adjustment for confounding in the analyses from which the main findings were drawn?*  This question should be answered no for trials if: the main conclusions of the study were based on analyses of treatment rather than intention to treat; the distribution of known confounders in the different treatment groups was  not described; or the distribution of known confounders differed between the treatment groups but was not taken into account in the analyses. In non-randomized studies if the effect of the main confounders was not investigated or confounding was demonstrated but no adjustment was made in the final analyses the question should be answered as no. |  |  |  |  |
| *26. Were losses of patients to follow‐up taken into account?*  If the numbers of patients lost to follow‐up are not reported, the question should be answered as unable to determine. If the proportion lost to follow-up was too small to affect the main findings, the question should be  answered yes. |  |  |  |  |
| **Power** |  |  |  |  |
| *27. Did the study have sufficient power to detect a clinically important effect where the probability value for a difference being due to chance is less than 5%?*  Sample sizes have been calculated to detect a difference of x% and y%. |  |  |  |  |
| **Total Score 13/14 = 92.8 %** | | | | |

| **Temporiti et al. 2019** | | | | |
| --- | --- | --- | --- | --- |
| **Reporting** | **YES** | **NO** | **UTD** | **Notes/Justification** |
| *1. Is the hypothesis/aim/objective of the study clearly described?* | X |  |  | The aim is clearly described. |
| *2. Are the main outcomes to be measured clearly described in the Introduction or Methods section?*  If the main outcomes are first mentioned in the Results section, the question should be answered no. | X |  |  | Clearly described in the Methods section. |
| *3. Are the characteristics of the patients included in the study clearly described?*  In cohort studies and trials, inclusion and/or exclusion criteria should be given. In case‐control studies, a case‐definition and the source for controls should be given. | X |  |  | Inclusion and exclusion criteria were clearly described in the Methods section. |
| *4. Are the interventions of interest clearly described?*  Treatments and placebo (where relevant) that are to be compared should be clearly described. |  |  |  |  |
| *5. Are the distributions of principal confounders in each group of subjects to be compared clearly described?*  A list of principal confounders is provided. | X |  |  | A list of the principal confounders is provided |
| *6. Are the main findings of the study clearly described?*  Simple outcome data (including denominators and numerators) should be reported for all major findings so that the reader can check the major analyses and conclusions. (This question does not cover statistical tests  which are considered below). | X |  |  | The main findings of the study are clearly described |
| *7. Does the study provide estimates of the random variability in the data for the main outcomes?*  In non-normally distributed data the inter‐quartile range of results should be reported. In normally distributed data the standard error, standard deviation or confidence intervals should be reported. If the distribution of the data is not described, it must be assumed that the estimates used were appropriate and the question should be answered yes. | X |  |  | The data was normally distributed and standard deviation has been reported |
| *8. Have all important adverse events that may be a consequence of the intervention been reported?*  This should be answered yes if the study demonstrates that there was a comprehensive attempt to measure adverse events. (A list of possible adverse events is provided). |  |  |  |  |
| *9. Have the characteristics of patients lost to follow‐up been described?*  This should be answered yes where there were no losses to follow‐up or where losses to follow‐up were so small that findings would be unaffected by their inclusion. This should be answered NO where a study does not report the number of patients lost to follow‐up. |  |  |  |  |
| *10. Have actual probability values been reported (e.g. 0.035 rather than < 0.05) for the main outcomes except where the probability value is less than 0.001?* | X |  |  | Probability values have been reported. |
| **External Validity** | **YES** | **NO** | **UTD** | **Notes/Justification** |
| *11. Were the subjects asked to participate in the study representative of the entire population from which they were recruited?*  The study must identify the source population for patients and describe how the patients were selected. Patients would be representative if they comprised the entire source population, an unselected sample of consecutive patients, or a random sample. Random sampling is only feasible where a list of all members of the relevant population exists. Where a study does not report the proportion of the source population from which the patients are derived, the question should be answered as unable to determine. |  |  | X | The study does not report the proportion of the source population from which the patients are derived |
| *12. Were those subjects who were prepared to participate representative of the entire population from which they were recruited?*  The proportion of those asked who agreed should be stated. Validation that the sample was representative would include demonstrating that the distribution of the main confounding factors was the same in the study sample and the source population. |  | X |  | The study does not report the proportion of the source population from which the patients are derived. |
| *13. Were the staff, places, and facilities where the patients were treated, representative of the treatment the majority of patients receive?*  For the question to be answered yes the study should demonstrate that the intervention was representative of that in use in the source population. The question should be answered no if, for example, the intervention was  undertaken in a specialist centre unrepresentative of the hospitals most of the source population would attend. |  |  |  |  |
| **Internal validity – bias** | **YES** | **NO** | **UTD** | **Notes/Justification** |
| *14. Was an attempt made to blind study subjects to the intervention they have received?*  For studies where the patients would have no way of knowing which intervention they received, this should be answered yes. |  |  |  |  |
| *15. Was an attempt made to blind those measuring the main outcomes of the intervention?* |  |  |  |  |
| *16. If any of the results of the study were based on “data dredging”, was this made clear?*  Any analyses that had not been planned at the outset of the study should be clearly indicated. If no retrospective unplanned subgroup analyses were reported, then answer yes. | X |  |  | No retrospective unplanned subgroup analyses were reported. |
| *17. In trials and cohort studies, do the analyses adjust for different lengths of follow‐up of patients, or in case-control studies, is the time period between the intervention and outcome the same for cases and controls?*  Where follow‐up was the same for all study patients the answer should yes.  If different lengths of follow‐up were adjusted for by, for example, survival analysis the answer should be yes. Studies where differences in follow‐up are ignored should be answered no. |  |  |  |  |
| *18. Were the statistical tests used to assess the main outcomes appropriate?*  The statistical techniques used must be appropriate to the data. For example, Non-parametric methods should be used for small sample sizes. Where little statistical analysis has been undertaken but where there is no evidence of bias, the question should be answered yes. If the distribution of the data (normal or not) is not described it must be assumed that the estimates used were appropriate and the question should be answered yes. | X |  |  | Statistics were appropriate and correctly reported. |
| *19. Was compliance with the intervention/s reliable?*  Where there was non compliance with the allocated treatment or where there was contamination of one group, the question should be answered no.  For studies where the effect of any misclassification was likely to bias any association to the null, the question should be answered yes. |  |  |  |  |
| *20. Were the main outcome measures used accurate (valid and reliable)?*  For studies where the outcome measures are clearly described, the question should be answered yes. For studies which refer to other work or that demonstrates the outcome measures are accurate, the question should be  answered as yes. | X |  |  | Outcome measures were accurate. |
| **Internal validity ‐ confounding (selection bias)** | **YES** | **NO** | **UTD** | **Notes/Justification** |
| *21. Were the patients in different intervention groups (trials and cohort studies) or were the cases and controls (case‐control studies) recruited from the same population?*  For example, patients for all comparison groups should be selected from the same hospital. The question should be answered unable to determine for cohort and case-control studies where there is no information concerning the source of patients included in the study. | X |  |  | Yes, patients were recruited by the same orthopaedic surgery hospital. |
| *22. Were study subjects in different intervention groups (trials and cohort- studies) or were the cases and controls (case‐control studies) recruited over the same period of time?*  For a study which does not specify the time period over which patients were recruited, the question should be answered as unable to determine. | X |  |  | The patients were recruited during a specified period of time |
| *23. Were study subjects randomised to intervention groups?*  Studies which state that subjects were randomized should be answered yes except where method of randomisation would not ensure random allocation.  For example, alternate allocation would score no because it is predictable. |  |  |  |  |
| *24. Was the randomised intervention assignment concealed from both patients and health care staff until recruitment was complete and irrevocable?*  All non‐randomised studies should be answered no. If assignment was concealed from patients but not from staff, it should be answered no. |  |  |  |  |
| *25. Was there adequate adjustment for confounding in the analyses from which the main findings were drawn?*  This question should be answered no for trials if: the main conclusions of the study were based on analyses of treatment rather than intention to treat; the distribution of known confounders in the different treatment groups was  not described; or the distribution of known confounders differed between the treatment groups but was not taken into account in the analyses. In non-randomized studies if the effect of the main confounders was not investigated or confounding was demonstrated but no adjustment was made in the final analyses the question should be answered as no. |  |  |  |  |
| *26. Were losses of patients to follow‐up taken into account?*  If the numbers of patients lost to follow‐up are not reported, the question should be answered as unable to determine. If the proportion lost to follow-up was too small to affect the main findings, the question should be answered yes. |  |  |  |  |
| **Power** | **YES** | **NO** | **UTD** | **Notes/Justification** |
| *27. Did the study have sufficient power to detect a clinically important effect where the probability value for a difference being due to chance is less than 5%?*  Sample sizes have been calculated to detect a difference of x% and y%. |  |  |  |  |
| **Total Score 12/14 = 85.7 %** | | | | |

| **Trudelle-Jackson et.al 2004** | | | | |
| --- | --- | --- | --- | --- |
| **Reporting** | **YES** | **NO** | **UTD** | **Notes/Justification** |
| *1. Is the hypothesis/aim/objective of the study clearly described?* | X |  |  | The aim is clearly described. |
| *2. Are the main outcomes to be measured clearly described in the Introduction or Methods section?*  If the main outcomes are first mentioned in the Results section, the question should be answered no. | X |  |  | Clearly described in the introduction section. |
| *3. Are the characteristics of the patients included in the study clearly described?*  In cohort studies and trials, inclusion and/or exclusion criteria should be given. In case‐control studies, a case‐definition and the source for controls should be given. | X |  |  | Clearly described in the Methods section and Figure 1. |
| *4. Are the interventions of interest clearly described?*  Treatments and placebo (where relevant) that are to be compared should be clearly described. | X |  |  | The interventions are clearly described. |
| *5. Are the distributions of principal confounders in each group of subjects to be compared clearly described?*  A list of principal confounders is provided. | X |  |  | Confounders have been listed. |
| *6. Are the main findings of the study clearly described?*  Simple outcome data (including denominators and numerators) should be reported for all major findings so that the reader can check the major analyses and conclusions. (This question does not cover statistical tests  which are considered below). | X |  |  | Data are clearly reported in the results section and tables. |
| *7. Does the study provide estimates of the random variability in the data for the main outcomes?*  In non-normally distributed data the inter‐quartile range of results should be reported. In normally distributed data the standard error, standard deviation or confidence intervals should be reported. If the distribution of the data is not described, it must be assumed that the estimates used were appropriate and the question should be answered yes. | X |  |  | Variability has been reported. |
| *8. Have all important adverse events that may be a consequence of the intervention been reported?*  This should be answered yes if the study demonstrates that there was a comprehensive attempt to measure adverse events. (A list of possible adverse events is provided). | X |  |  | Some measures to counteract adverse events of the training have been reported (e.g. “The first follow-up visit was scheduled within the next 2 weeks to ensure that subjects were performing the exercises correctly and to progress the exercises as needed.”) |
| *9. Have the characteristics of patients lost to follow‐up been described?*  This should be answered yes where there were no losses to follow‐up or where losses to follow‐up were so small that findings would be unaffected by their inclusion. This should be answered NO where a study does not report the number of patients lost to follow‐up. | X |  |  | Losses to follow-up were small. |
| *10. Have actual probability values been reported (e.g. 0.035 rather than < 0.05) for the main outcomes except where the probability value is less than 0.001?* | X |  |  | Probability values have been reported. |
| **External Validity** | **YES** | **NO** | **UTD** | **Notes/Justification** |
| *11. Were the subjects asked to participate in the study representative of the entire population from which they were recruited?*  The study must identify the source population for patients and describe how the patients were selected. Patients would be representative if they comprised the entire source population, an unselected sample of consecutive patients, or a random sample. Random sampling is only feasible where a list of all members of the relevant population exists. Where a study does not report the proportion of the source population from which the patients are derived, the question should be answered as unable to determine. | X |  |  | Population was representative. |
| *12. Were those subjects who were prepared to participate representative of the entire population from which they were recruited?*  The proportion of those asked who agreed should be stated. Validation that the sample was representative would include demonstrating that the distribution of the main confounding factors was the same in the study sample and the source population. | X |  |  | The proportion of those asked who agreed is stated. |
| *13. Were the staff, places, and facilities where the patients were treated, representative of the treatment the majority of patients receive?*  For the question to be answered yes the study should demonstrate that the intervention was representative of that in use in the source population. The question should be answered no if, for example, the intervention was  undertaken in a specialist centre unrepresentative of the hospitals most of the source population would attend. | X |  |  | Patients were trained in a usual setting of rehabilitation and instructed to perform exercise at home. |
| **Internal validity – bias** | **YES** | **NO** | **UTD** | **Notes/Justification** |
| *14. Was an attempt made to blind study subjects to the intervention they have received?*  For studies where the patients would have no way of knowing which intervention they received, this should be answered yes. | X |  |  | Patients were unaware of their allocation. |
| *15. Was an attempt made to blind those measuring the main outcomes of the intervention?* |  | X |  | Patients were tested by examiners who were not blinded on patients’ allocation. |
| *16. If any of the results of the study were based on “data dredging”, was this made clear?*  Any analyses that had not been planned at the outset of the study should be clearly indicated. If no retrospective unplanned subgroup analyses were reported, then answer yes. | X |  |  | No retrospective unplanned subgroup analyses were reported. |
| *17. In trials and cohort studies, do the analyses adjust for different lengths of follow‐up of patients, or in case-control studies, is the time period between the intervention and outcome the same for cases and controls?*  Where follow‐up was the same for all study patients the answer should yes.  If different lengths of follow‐up were adjusted for by, for example, survival analysis the answer should be yes. Studies where differences in follow‐up are ignored should be answered no. | X |  |  | All patients were tested at the same time points. |
| *18. Were the statistical tests used to assess the main outcomes appropriate?*  The statistical techniques used must be appropriate to the data. For example, Non-parametric methods should be used for small sample sizes. Where little statistical analysis has been undertaken but where there is no evidence of bias, the question should be answered yes. If the distribution of the data (normal or not) is not described it must be assumed that the estimates used were appropriate and the question should be answered yes. | X |  |  | Statistics were appropriate and correctly reported. |
| *19. Was compliance with the intervention/s reliable?*  Where there was non compliance with the allocated treatment or where there was contamination of one group, the question should be answered no.  For studies where the effect of any misclassification was likely to bias any association to the null, the question should be answered yes. | X |  |  | Compliance with the interventions was reliable. |
| *20. Were the main outcome measures used accurate (valid and reliable)?*  For studies where the outcome measures are clearly described, the question should be answered yes. For studies which refer to other work or that demonstrates the outcome measures are accurate, the question should be  answered as yes. | X |  |  | Outcome measures were accurate. |
| **Internal validity ‐ confounding (selection bias)** | **YES** | **NO** | **UTD** | **Notes/Justification** |
| *21. Were the patients in different intervention groups (trials and cohort studies) or were the cases and controls (case‐control studies) recruited from the same population?*  For example, patients for all comparison groups should be selected from the same hospital. The question should be answered unable to determine for cohort and case-control studies where there is no information concerning the source of patients included in the study. | X |  |  | Patients were recruited from the same population. |
| *22. Were study subjects in different intervention groups (trials and cohort- studies) or were the cases and controls (case‐control studies) recruited over the same period of time?*  For a study which does not specify the time period over which patients were recruited, the question should be answered as unable to determine. |  |  | X | The time period over which patients were recruited is not known. |
| *23. Were study subjects randomised to intervention groups?*  Studies which state that subjects were randomized should be answered yes except where method of randomisation would not ensure random allocation.  For example, alternate allocation would score no because it is predictable. | X |  |  | Participants were randomly allocated by using a randomization table. |
| *24. Was the randomised intervention assignment concealed from both patients and health care staff until recruitment was complete and irrevocable?*  All non‐randomised studies should be answered no. If assignment was concealed from patients but not from staff, it should be answered no. |  | X |  | Physical therapists were aware of patients’ allocation. |
| *25. Was there adequate adjustment for confounding in the analyses from which the main findings were drawn?*  This question should be answered no for trials if: the main conclusions of the study were based on analyses of treatment rather than intention to treat; the distribution of known confounders in the different treatment groups was  not described; or the distribution of known confounders differed between the treatment groups but was not taken into account in the analyses. In non-randomized studies if the effect of the main confounders was not investigated or confounding was demonstrated but no adjustment was made in the final analyses the question should be answered as no. | X |  |  | Distribution of potentially confounders was the same between the groups. |
| *26. Were losses of patients to follow‐up taken into account?*  If the numbers of patients lost to follow‐up are not reported, the question should be answered as unable to determine. If the proportion lost to follow-up was too small to affect the main findings, the question should be answered yes. | X |  |  | Lost in follow-up was small. |
| **Power** | **YES** | **NO** | **UTD** | **Notes/Justification** |
| *27. Did the study have sufficient power to detect a clinically important effect where the probability value for a difference being due to chance is less than 5%?*  Sample sizes have been calculated to detect a difference of x% and y%. |  | X |  | The sample size calculation and the probability for a difference have not been reported, and the sample (14+14 subjects) is small. |
| **Total Score 23/27 = 85.1 %** | | | | |

| **Ulivi et al. 2021** | | | | |
| --- | --- | --- | --- | --- |
| **Reporting** | **YES** | **NO** | **UTD** | **Notes/Justification** |
| *1. Is the hypothesis/aim/objective of the study clearly described?* | X |  |  | The aim is clearly described. |
| *2. Are the main outcomes to be measured clearly described in the Introduction or Methods section?*  If the main outcomes are first mentioned in the Results section, the question should be answered no. | X |  |  | Clearly described in the introduction section. |
| *3. Are the characteristics of the patients included in the study clearly described?*  In cohort studies and trials, inclusion and/or exclusion criteria should be given. In case‐control studies, a case‐definition and the source for controls should be given. | X |  |  | Clearly described in the Methods section and Table 1. |
| *4. Are the interventions of interest clearly described?*  Treatments and placebo (where relevant) that are to be compared should be clearly described. | X |  |  | The interventions are clearly described. |
| *5. Are the distributions of principal confounders in each group of subjects to be compared clearly described?*  A list of principal confounders is provided. | X |  |  | Confounders have been listed. |
| *6. Are the main findings of the study clearly described?*  Simple outcome data (including denominators and numerators) should be reported for all major findings so that the reader can check the major analyses and conclusions. (This question does not cover statistical tests  which are considered below). | X |  |  | Data are clearly reported in the results section and tables. |
| *7. Does the study provide estimates of the random variability in the data for the main outcomes?*  In non-normally distributed data the inter‐quartile range of results should be reported. In normally distributed data the standard error, standard deviation or confidence intervals should be reported. If the distribution of the data is not described, it must be assumed that the estimates used were appropriate and the question should be answered yes. | X |  |  | Variability has been reported. |
| *8. Have all important adverse events that may be a consequence of the intervention been reported?*  This should be answered yes if the study demonstrates that there was a comprehensive attempt to measure adverse events. (A list of possible adverse events is provided). | X |  |  | Some measures to counteract adverse events of the training have been reported (e.g. “The first follow-up visit was scheduled within the next 2 weeks to ensure that subjects were performing the exercises correctly and to progress the exercises as needed.”) |
| *9. Have the characteristics of patients lost to follow‐up been described?*  This should be answered yes where there were no losses to follow‐up or where losses to follow‐up were so small that findings would be unaffected by their inclusion. This should be answered NO where a study does not report the number of patients lost to follow‐up. | X |  |  |  |
| *10. Have actual probability values been reported (e.g. 0.035 rather than < 0.05) for the main outcomes except where the probability value is less than 0.001?* | X |  |  |  |
| **External Validity** | **YES** | **NO** | **UTD** | **Notes/Justification** |
| *11. Were the subjects asked to participate in the study representative of the entire population from which they were recruited?*  The study must identify the source population for patients and describe how the patients were selected. Patients would be representative if they comprised the entire source population, an unselected sample of consecutive patients, or a random sample. Random sampling is only feasible where a list of all members of the relevant population exists. Where a study does not report the proportion of the source population from which the patients are derived, the question should be answered as unable to determine. | X |  |  | Population was representative. |
| *12. Were those subjects who were prepared to participate representative of the entire population from which they were recruited?*  The proportion of those asked who agreed should be stated. Validation that the sample was representative would include demonstrating that the distribution of the main confounding factors was the same in the study sample and the source population. | X |  |  | The proportion of those asked who agreed is stated. |
| *13. Were the staff, places, and facilities where the patients were treated, representative of the treatment the majority of patients receive?*  For the question to be answered yes the study should demonstrate that the intervention was representative of that in use in the source population. The question should be answered no if, for example, the intervention was  undertaken in a specialist centre unrepresentative of the hospitals most of the source population would attend. | X |  |  | Patients were trained in a usual setting of rehabilitation and instructed to perform exercise at home. |
| **Internal validity – bias** | **YES** | **NO** | **UTD** | **Notes/Justification** |
| *14. Was an attempt made to blind study subjects to the intervention they have received?*  For studies where the patients would have no way of knowing which intervention they received, this should be answered yes. |  |  | X |  |
| *15. Was an attempt made to blind those measuring the main outcomes of the intervention?* |  |  | X |  |
| *16. If any of the results of the study were based on “data dredging”, was this made clear?*  Any analyses that had not been planned at the outset of the study should be clearly indicated. If no retrospective unplanned subgroup analyses were reported, then answer yes. | X |  |  | No retrospective unplanned subgroup analyses were reported. |
| *17. In trials and cohort studies, do the analyses adjust for different lengths of follow‐up of patients, or in case-control studies, is the time period between the intervention and outcome the same for cases and controls?*  Where follow‐up was the same for all study patients the answer should yes.  If different lengths of follow‐up were adjusted for by, for example, survival analysis the answer should be yes. Studies where differences in follow‐up are ignored should be answered no. | X |  |  | All patients were tested at the same time points. |
| *18. Were the statistical tests used to assess the main outcomes appropriate?*  The statistical techniques used must be appropriate to the data. For example, Non-parametric methods should be used for small sample sizes. Where little statistical analysis has been undertaken but where there is no evidence of bias, the question should be answered yes. If the distribution of the data (normal or not) is not described it must be assumed that the estimates used were appropriate and the question should be answered yes. | X |  |  | Statistics were appropriate and correctly reported. |
| *19. Was compliance with the intervention/s reliable?*  Where there was non compliance with the allocated treatment or where there was contamination of one group, the question should be answered no.  For studies where the effect of any misclassification was likely to bias any association to the null, the question should be answered yes. | X |  |  | Compliance with the interventions was reliable. |
| *20. Were the main outcome measures used accurate (valid and reliable)?*  For studies where the outcome measures are clearly described, the question should be answered yes. For studies which refer to other work or that demonstrates the outcome measures are accurate, the question should be  answered as yes. | X |  |  | Outcome measures were accurate. |
| **Internal validity ‐ confounding (selection bias)** | **YES** | **NO** | **UTD** | **Notes/Justification** |
| *21. Were the patients in different intervention groups (trials and cohort studies) or were the cases and controls (case‐control studies) recruited from the same population?*  For example, patients for all comparison groups should be selected from the same hospital. The question should be answered unable to determine for cohort and case-control studies where there is no information concerning the source of patients included in the study. | X |  |  | Patients were recruited from the same population. |
| *22. Were study subjects in different intervention groups (trials and cohort- studies) or were the cases and controls (case‐control studies) recruited over the same period of time?*  For a study which does not specify the time period over which patients were recruited, the question should be answered as unable to determine. | X |  |  |  |
| *23. Were study subjects randomised to intervention groups?*  Studies which state that subjects were randomized should be answered yes except where method of randomisation would not ensure random allocation.  For example, alternate allocation would score no because it is predictable. | X |  |  |  |
| *24. Was the randomised intervention assignment concealed from both patients and health care staff until recruitment was complete and irrevocable?*  All non‐randomised studies should be answered no. If assignment was concealed from patients but not from staff, it should be answered no. |  |  | X |  |
| *25. Was there adequate adjustment for confounding in the analyses from which the main findings were drawn?*  This question should be answered no for trials if: the main conclusions of the study were based on analyses of treatment rather than intention to treat; the distribution of known confounders in the different treatment groups was  not described; or the distribution of known confounders differed between the treatment groups but was not taken into account in the analyses. In non-randomized studies if the effect of the main confounders was not investigated or confounding was demonstrated but no adjustment was made in the final analyses the question should be answered as no. | X |  |  |  |
| *26. Were losses of patients to follow‐up taken into account?*  If the numbers of patients lost to follow‐up are not reported, the question should be answered as unable to determine. If the proportion lost to follow-up was too small to affect the main findings, the question should be answered yes. | X |  |  |  |
| **Power** | **YES** | **NO** | **UTD** | **Notes/Justification** |
| *27. Did the study have sufficient power to detect a clinically important effect where the probability value for a difference being due to chance is less than 5%?*  Sample sizes have been calculated to detect a difference of x% and y%. | X |  |  |  |
| **Total Score 14/14 = 100 %** | | | | |

| **Van Driesschen et al. 2016** | | | | |
| --- | --- | --- | --- | --- |
| **Reporting** | **YES** | **NO** | **UTD** | **Notes/Justification** |
| *1. Is the hypothesis/aim/objective of the study clearly described?* | X |  |  | The aim is clearly described. |
| *2. Are the main outcomes to be measured clearly described in the Introduction or Methods section?*  If the main outcomes are first mentioned in the Results section, the question should be answered no. | X |  |  | Clearly described in the Methods section. |
| *3. Are the characteristics of the patients included in the study clearly described?*  In cohort studies and trials, inclusion and/or exclusion criteria should be given. In case‐control studies, a case‐definition and the source for controls should be given. | X |  |  | Clearly described in the Methods section. |
| *4. Are the interventions of interest clearly described?*  Treatments and placebo (where relevant) that are to be compared should be clearly described. | X |  |  | The interventions are clearly described. |
| *5. Are the distributions of principal confounders in each group of subjects to be compared clearly described?*  A list of principal confounders is provided. | X |  |  | Confounders have been listed. |
| *6. Are the main findings of the study clearly described?*  Simple outcome data (including denominators and numerators) should be reported for all major findings so that the reader can check the major analyses and conclusions. (This question does not cover statistical tests  which are considered below). | X |  |  | The main findings are clearly described. |
| *7. Does the study provide estimates of the random variability in the data for the main outcomes?*  In non-normally distributed data the inter‐quartile range of results should be reported. In normally distributed data the standard error, standard deviation or confidence intervals should be reported. If the distribution of the data is not described, it must be assumed that the estimates used were appropriate and the question should be answered yes. | X |  |  | Variability has been reported. |
| *8. Have all important adverse events that may be a consequence of the intervention been reported?*  This should be answered yes if the study demonstrates that there was a comprehensive attempt to measure adverse events. (A list of possible adverse events is provided). |  |  | X |  |
| *9. Have the characteristics of patients lost to follow‐up been described?*  This should be answered yes where there were no losses to follow‐up or where losses to follow‐up were so small that findings would be unaffected by their inclusion. This should be answered NO where a study does not report the number of patients lost to follow‐up. |  | X |  | Losses to follow-up have not been reported. |
| *10. Have actual probability values been reported (e.g. 0.035 rather than < 0.05) for the main outcomes except where the probability value is less than 0.001?* | X |  |  | Probability values have been reported. |
| **External Validity** | **YES** | **NO** | **UTD** | **Notes/Justification** |
| *11. Were the subjects asked to participate in the study representative of the entire population from which they were recruited?*  The study must identify the source population for patients and describe how the patients were selected. Patients would be representative if they comprised the entire source population, an unselected sample of consecutive patients, or a random sample. Random sampling is only feasible where a list of all members of the relevant population exists. Where a study does not report the proportion of the source population from which the patients are derived, the question should be answered as unable to determine. |  |  | X | The study does not report the proportion of the source population from which the patients are derived |
| *12. Were those subjects who were prepared to participate representative of the entire population from which they were recruited?*  The proportion of those asked who agreed should be stated. Validation that the sample was representative would include demonstrating that the distribution of the main confounding factors was the same in the study sample and the source population. |  |  | X |  |
| *13. Were the staff, places, and facilities where the patients were treated, representative of the treatment the majority of patients receive?*  For the question to be answered yes the study should demonstrate that the intervention was representative of that in use in the source population. The question should be answered no if, for example, the intervention was  undertaken in a specialist centre unrepresentative of the hospitals most of the source population would attend. | X |  |  | Staff, places, and facilities were representative. |
| **Internal validity – bias** | **YES** | **NO** | **UTD** | **Notes/Justification** |
| *14. Was an attempt made to blind study subjects to the intervention they have received?*  For studies where the patients would have no way of knowing which intervention they received, this should be answered yes. |  |  | X |  |
| *15. Was an attempt made to blind those measuring the main outcomes of the intervention?* |  |  | X | No information on blinding has been reported. |
| *16. If any of the results of the study were based on “data dredging”, was this made clear?*  Any analyses that had not been planned at the outset of the study should be clearly indicated. If no retrospective unplanned subgroup analyses were reported, then answer yes. | X |  |  | No retrospective unplanned subgroup analyses were reported. |
| *17. In trials and cohort studies, do the analyses adjust for different lengths of follow‐up of patients, or in case-control studies, is the time period between the intervention and outcome the same for cases and controls?*  Where follow‐up was the same for all study patients the answer should yes.  If different lengths of follow‐up were adjusted for by, for example, survival analysis the answer should be yes. Studies where differences in follow‐up are ignored should be answered no. | X |  |  |  |
| *18. Were the statistical tests used to assess the main outcomes appropriate?*  The statistical techniques used must be appropriate to the data. For example, Non-parametric methods should be used for small sample sizes. Where little statistical analysis has been undertaken but where there is no evidence of bias, the question should be answered yes. If the distribution of the data (normal or not) is not described it must be assumed that the estimates used were appropriate and the question should be answered yes. | X |  |  | Statistics were appropriate and correctly reported. |
| *19. Was compliance with the intervention/s reliable?*  Where there was non compliance with the allocated treatment or where there was contamination of one group, the question should be answered no.  For studies where the effect of any misclassification was likely to bias any association to the null, the question should be answered yes. | X |  |  | Compliance with the interventions was reliable. |
| *20. Were the main outcome measures used accurate (valid and reliable)?*  For studies where the outcome measures are clearly described, the question should be answered yes. For studies which refer to other work or that demonstrates the outcome measures are accurate, the question should be  answered as yes. | X |  |  | Outcome measures were accurate. |
| **Internal validity ‐ confounding (selection bias)** | **YES** | **NO** | **UTD** | **Notes/Justification** |
| *21. Were the patients in different intervention groups (trials and cohort studies) or were the cases and controls (case‐control studies) recruited from the same population?*  For example, patients for all comparison groups should be selected from the same hospital. The question should be answered unable to determine for cohort and case-control studies where there is no information concerning the source of patients included in the study. |  | X |  | Patients in the 3 groups have been recruited in 3 different hospitals. |
| *22. Were study subjects in different intervention groups (trials and cohort- studies) or were the cases and controls (case‐control studies) recruited over the same period of time?*  For a study which does not specify the time period over which patients were recruited, the question should be answered as unable to determine. | X |  |  | Participants are consecutive patients. |
| *23. Were study subjects randomised to intervention groups?*  Studies which state that subjects were randomized should be answered yes except where method of randomisation would not ensure random allocation.  For example, alternate allocation would score no because it is predictable. |  | X |  | No information on randomization has been reported. |
| *24. Was the randomised intervention assignment concealed from both patients and health care staff until recruitment was complete and irrevocable?*  All non‐randomised studies should be answered no. If assignment was concealed from patients but not from staff, it should be answered no. |  |  | X | There is no information in the text. |
| *25. Was there adequate adjustment for confounding in the analyses from which the main findings were drawn?*  This question should be answered no for trials if: the main conclusions of the study were based on analyses of treatment rather than intention to treat; the distribution of known confounders in the different treatment groups was  not described; or the distribution of known confounders differed between the treatment groups but was not taken into account in the analyses. In non-randomized studies if the effect of the main confounders was not investigated or confounding was demonstrated but no adjustment was made in the final analyses the question should be answered as no. |  | X |  | No data adjustment has been reported. |
| *26. Were losses of patients to follow‐up taken into account?*  If the numbers of patients lost to follow‐up are not reported, the question should be answered as unable to determine. If the proportion lost to follow-up was too small to affect the main findings, the question should be answered yes. |  |  | X | No information has been reported. |
| **Power** | **YES** | **NO** | **UTD** | **Notes/Justification** |
| *27. Did the study have sufficient power to detect a clinically important effect where the probability value for a difference being due to chance is less than 5%?*  Sample sizes have been calculated to detect a difference of x% and y%. |  |  | X | No information on statistical power calculation. |
| **Total Score 15/27 = 55.5 %** | | | | |

| **Wang et al. 2016** | | | | |
| --- | --- | --- | --- | --- |
| **Reporting** | **YES** | **NO** | **UTD** | **Notes/Justification** |
| *1. Is the hypothesis/aim/objective of the study clearly described?* | X |  |  | The aim, is clearly described in the Introduction section. |
| *2. Are the main outcomes to be measured clearly described in the Introduction or Methods section?*  If the main outcomes are first mentioned in the Results section, the question should be answered no. | X |  |  | Clearly described in the Introduction section. |
| *3. Are the characteristics of the patients included*  *in the study clearly described?*  In cohort studies and trials, inclusion and/or exclusion criteria should be given. In case‐control studies, a case‐definition and the source for controls should be given. | X |  |  | Clearly described in the Methods section. |
| *4. Are the interventions of interest clearly described?*  Treatments and placebo (where relevant) that are to be compared should be clearly described. |  |  |  |  |
| *5. Are the distributions of principal confounders in each group of subjects to be compared clearly described?*  A list of principal confounders is provided. | X |  |  | Confounders were reported. |
| *6. Are the main findings of the study clearly described?*  Simple outcome data (including denominators and numerators) should be reported for all major findings so that the reader can check the major analyses and conclusions. (This question does not cover statistical tests  which are considered below). | X |  |  | Clearly reported in the Results section and table 2. |
| *7. Does the study provide estimates of the random variability in the data for the main outcomes?*  In non-normally distributed data the inter‐quartile range of results should be reported. In normally distributed data the standard error, standard deviation or confidence intervals should be reported. If the distribution of the data is not described, it must be assumed that the estimates used were appropriate and the question should be answered yes. | X |  |  | Standard deviation has been reported. |
| *8. Have all important adverse events that may be a consequence of the intervention been reported?*  This should be answered yes if the study demonstrates that there was a comprehensive attempt to measure adverse events. (A list of possible adverse events is provided). |  |  |  |  |
| *9. Have the characteristics of patients lost to follow‐up been described?*  This should be answered yes where there were no losses to follow‐up or where losses to follow‐up were so small that findings would be unaffected by their inclusion. This should be answered NO where a study does not report the number of patients lost to follow‐up. |  |  |  |  |
| *10. Have actual probability values been reported ( e.g. 0.035 rather than < 0.05) for the main outcomes except where the probability value is less than 0.001?* | X |  |  | Probability values have been reported. |
| **External Validity** | **YES** | **NO** | **UTD** | **Notes/Justification** |
| *11. Were the subjects asked to participate in the study representative of the entire population from which they were recruited?*  The study must identify the source population for patients and describe how the patients were selected. Patients would be representative if they comprised the entire source population, an unselected sample of consecutive patients, or a random sample. Random sampling is only feasible where a list of all members of the relevant population exists. Where a study does not report the proportion of the source population from which the patients are derived, the question should be answered as unable to determine. | X |  |  | Participants were representative. |
| *12. Were those subjects who were prepared to participate representative of the entire population from which they were recruited?*  The proportion of those asked who agreed should be stated. Validation that the sample was representative would include demonstrating that the distribution of the main confounding factors was the same in the study sample and the source population. | X |  |  | The population is representative. |
| *13. Were the staff, places, and facilities where the patients were treated, representative of the treatment the majority of patients receive?*  For the question to be answered yes the study should demonstrate that the intervention was representative of that in use in the source population. The question should be answered no if, for example, the intervention was  undertaken in a specialist centre unrepresentative of the hospitals most of the source population would attend. |  |  |  |  |
| **Internal validity – bias** | **YES** | **NO** | **UTD** | **Notes/Justification** |
| *14. Was an attempt made to blind study subjects to the intervention they have received?*  For studies where the patients would have no way of knowing which intervention they received, this should be answered yes. |  |  |  |  |
| *15. Was an attempt made to blind those measuring the main outcomes of the intervention?* |  |  |  |  |
| *16. If any of the results of the study were based on “data dredging”, was this made clear?*  Any analyses that had not been planned at the outset of the study should be clearly indicated. If no retrospective unplanned subgroup analyses were reported, then answer yes. | X |  |  | No retrospective unplanned subgroup analyses were reported. |
| *17. In trials and cohort studies, do the analyses adjust for different lengths of follow‐up of patients, or in case-control studies, is the time period between the intervention and outcome the same for cases and controls?*  Where follow‐up was the same for all study patients the answer should yes.  If different lengths of follow‐up were adjusted for by, for example, survival analysis the answer should be yes. Studies where differences in follow‐up are ignored should be answered no. |  |  |  |  |
| *18. Were the statistical tests used to assess the main outcomes appropriate?*  The statistical techniques used must be appropriate to the data. For example, Non-parametric methods should be used for small sample sizes. Where little statistical analysis has been undertaken but where there is no evidence of bias, the question should be answered yes. If the distribution of the data (normal or not) is not described it must be assumed that the estimates used were appropriate and the question should be answered yes. | X |  |  | Statistics were appropriate and correctly reported. |
| *19. Was compliance with the intervention/s reliable?*  Where there was non compliance with the allocated treatment or where there was contamination of one group, the question should be answered no.  For studies where the effect of any misclassification was likely to bias any association to the null, the question should be answered yes. |  |  |  |  |
| *20. Were the main outcome measures used accurate (valid and reliable)?*  For studies where the outcome measures are clearly described, the question should be answered yes. For studies which refer to other work or that demonstrates the outcome measures are accurate, the question should be  answered as yes. | X |  |  | Outcome measures clearly described. |
| **Internal validity ‐ confounding (selection bias)** | **YES** | **NO** | **UTD** | **Notes/Justification** |
| *21. Were the patients in different intervention groups (trials and cohort studies) or were the cases and controls (case‐control studies) recruited from the same population?*  For example, patients for all comparison groups should be selected from the same hospital. The question should be answered unable to determine for cohort and case-control studies where there is no information concerning the source of patients included in the study. | X |  |  | The patients were recruited in the same hospital. |
| *22. Were study subjects in different intervention groups (trials and cohort- studies) or were the cases and controls (case‐control studies) recruited over the same period of time?*  For a study which does not specify the time period over which patients were recruited, the question should be answered as unable to determine. | X |  |  | Patients were recruited over the same time period. |
| *23. Were study subjects randomised to intervention groups?*  Studies which state that subjects were randomized should be answered yes except where method of randomisation would not ensure random allocation.  For example, alternate allocation would score no because it is predictable. |  |  |  |  |
| *24. Was the randomised intervention assignment concealed from both patients and health care staff until recruitment was complete and irrevocable?*  All non‐randomised studies should be answered no. If assignment was concealed from patients but not from staff, it should be answered no. |  |  |  |  |
| *25. Was there adequate adjustment for confounding in the analyses from which the main findings were drawn?*  This question should be answered no for trials if: the main conclusions of the study were based on analyses of treatment rather than intention to treat; the distribution of known confounders in the different treatment groups was  not described; or the distribution of known confounders differed between the treatment groups but was not taken into account in the analyses. In non-randomized studies if the effect of the main confounders was not investigated or confounding was demonstrated but no adjustment was made in the final analyses the question should be answered as no. |  |  |  |  |
| *26. Were losses of patients to follow‐up taken into account?*  If the numbers of patients lost to follow‐up are not reported, the question should be answered as unable to determine. If the proportion lost to follow-up was too small to affect the main findings, the question should be  answered yes. |  |  |  |  |
| **Power** |  |  |  |  |
| *27. Did the study have sufficient power to detect a clinically important effect where the probability value for a difference being due to chance is less than 5%?*  Sample sizes have been calculated to detect a difference of x% and y%. |  |  |  |  |
| **Total Score 14/14 = 100 %** | | | | |

| **Wareńczak et al. 2019** | | | | |
| --- | --- | --- | --- | --- |
| **Reporting** | **YES** | **NO** | **UTD** | **Notes/Justification** |
| *1. Is the hypothesis/aim/objective of the study clearly described?* | X |  |  | The aim is clearly described. |
| *2. Are the main outcomes to be measured clearly described in the Introduction or Methods section?*  If the main outcomes are first mentioned in the Results section, the question should be answered no. | X |  |  | Clearly described in the Methods section. |
| *3. Are the characteristics of the patients included*  *in the study clearly described?*  In cohort studies and trials, inclusion and/or exclusion criteria should be given. In case‐control studies, a case‐definition and the source for controls should be given. | X |  |  | Clearly described in the Methods section and table 1. |
| *4. Are the interventions of interest clearly described?*  Treatments and placebo (where relevant) that are to be compared should be clearly described. |  |  |  |  |
| *5. Are the distributions of principal confounders in each group of subjects to be compared clearly described?*  A list of principal confounders is provided. | X |  |  | Reported in the Methods section. |
| *6. Are the main findings of the study clearly described?*  Simple outcome data (including denominators and numerators) should be reported for all major findings so that the reader can check the major analyses and conclusions. (This question does not cover statistical tests  which are considered below). | X |  |  | Reported in the Results section and Table 2. |
| *7. Does the study provide estimates of the random variability in the data for the main outcomes?*  In non-normally distributed data the inter‐quartile range of results should be reported. In normally distributed data the standard error, standard deviation or confidence intervals should be reported. If the distribution of the data is not described, it must be assumed that the estimates used were appropriate and the question should be answered yes. | X |  |  | Standard deviation has been reported. |
| *8. Have all important adverse events that may be a consequence of the intervention been reported?*  This should be answered yes if the study demonstrates that there was a comprehensive attempt to measure adverse events. (A list of possible adverse events is provided). |  |  |  |  |
| *9. Have the characteristics of patients lost to follow‐up been described?*  This should be answered yes where there were no losses to follow‐up or where losses to follow‐up were so small that findings would be unaffected by their inclusion. This should be answered NO where a study does not report the number of patients lost to follow‐up. |  |  |  |  |
| *10. Have actual probability values been reported ( e.g. 0.035 rather than < 0.05) for the main outcomes except where the probability value is less than 0.001?* | X |  |  | Probability values have been reported. |
| **External Validity** | **YES** | **NO** | **UTD** | **Notes/Justification** |
| *11. Were the subjects asked to participate in the study representative of the entire population from which they were recruited?*  The study must identify the source population for patients and describe how the patients were selected. Patients would be representative if they comprised the entire source population, an unselected sample of consecutive patients, or a random sample. Random sampling is only feasible where a list of all members of the relevant population exists. Where a study does not report the proportion of the source population from which the patients are derived, the question should be answered as unable to determine. | X |  |  | Participants were representative. |
| *12. Were those subjects who were prepared to participate representative of the entire population from which they were recruited?*  The proportion of those asked who agreed should be stated. Validation that the sample was representative would include demonstrating that the distribution of the main confounding factors was the same in the study sample and the source population. | X |  |  | Sample was representative. |
| *13. Were the staff, places, and facilities where the patients were treated, representative of the treatment the majority of patients receive?*  For the question to be answered yes the study should demonstrate that the intervention was representative of that in use in the source population. The question should be answered no if, for example, the intervention was  undertaken in a specialist centre unrepresentative of the hospitals most of the source population would attend. |  |  |  |  |
| **Internal validity – bias** | **YES** | **NO** | **UTD** | **Notes/Justification** |
| *14. Was an attempt made to blind study subjects to the intervention they have received?*  For studies where the patients would have no way of knowing which intervention they received, this should be answered yes. |  |  |  |  |
| *15. Was an attempt made to blind those measuring the main outcomes of the intervention?* |  |  |  |  |
| *16. If any of the results of the study were based on “data dredging”, was this made clear?*  Any analyses that had not been planned at the outset of the study should be clearly indicated. If no retrospective unplanned subgroup analyses were reported, then answer yes. | X |  |  | No retrospective unplanned subgroup analyses were reported. |
| *17. In trials and cohort studies, do the analyses adjust for different lengths of follow‐up of patients, or in case-control studies, is the time period between the intervention and outcome the same for cases and controls?*  Where follow‐up was the same for all study patients the answer should yes.  If different lengths of follow‐up were adjusted for by, for example, survival analysis the answer should be yes. Studies where differences in follow‐up are ignored should be answered no. |  |  |  |  |
| *18. Were the statistical tests used to assess the main outcomes appropriate?*  The statistical techniques used must be appropriate to the data. For example, Non-parametric methods should be used for small sample sizes. Where little statistical analysis has been undertaken but where there is no evidence of bias, the question should be answered yes. If the distribution of the data (normal or not) is not described it must be assumed that the estimates used were appropriate and the question should be answered yes. | X |  |  | Statistics were appropriate and correctly reported. |
| *19. Was compliance with the intervention/s reliable?*  Where there was non compliance with the allocated treatment or where there was contamination of one group, the question should be answered no.  For studies where the effect of any misclassification was likely to bias any association to the null, the question should be answered yes. |  |  |  |  |
| *20. Were the main outcome measures used accurate (valid and reliable)?*  For studies where the outcome measures are clearly described, the question should be answered yes. For studies which refer to other work or that demonstrates the outcome measures are accurate, the question should be  answered as yes. | X |  |  | Outcome measures clearly described. |
| **Internal validity ‐ confounding (selection bias)** | **YES** | **NO** | **UTD** | **Notes/Justification** |
| *21. Were the patients in different intervention groups (trials and cohort studies) or were the cases and controls (case‐control studies) recruited from the same population?*  For example, patients for all comparison groups should be selected from the same hospital. The question should be answered unable to determine for cohort and case-control studies where there is no information concerning the source of patients included in the study. | X |  |  | Patients were recruited from the same hospital. |
| *22. Were study subjects in different intervention groups (trials and cohort- studies) or were the cases and controls (case‐control studies) recruited over the same period of time?*  For a study which does not specify the time period over which patients were recruited, the question should be answered as unable to determine. | X |  |  | Patients were recruited over the same time period. |
| *23. Were study subjects randomised to intervention groups?*  Studies which state that subjects were randomized should be answered yes except where method of randomisation would not ensure random allocation.  For example, alternate allocation would score no because it is predictable. |  |  |  |  |
| *24. Was the randomised intervention assignment concealed from both patients and health care staff until recruitment was complete and irrevocable?*  All non‐randomised studies should be answered no. If assignment was concealed from patients but not from staff, it should be answered no. |  |  |  |  |
| *25. Was there adequate adjustment for confounding in the analyses from which the main findings were drawn?*  This question should be answered no for trials if: the main conclusions of the study were based on analyses of treatment rather than intention to treat; the distribution of known confounders in the different treatment groups was  not described; or the distribution of known confounders differed between the treatment groups but was not taken into account in the analyses. In non-randomized studies if the effect of the main confounders was not investigated or confounding was demonstrated but no adjustment was made in the final analyses the question should be answered as no. |  |  |  |  |
| *26. Were losses of patients to follow‐up taken into account?*  If the numbers of patients lost to follow‐up are not reported, the question should be answered as unable to determine. If the proportion lost to follow-up was too small to affect the main findings, the question should be  answered yes. |  |  |  |  |
| **Power** |  |  |  |  |
| *27. Did the study have sufficient power to detect a clinically important effect where the probability value for a difference being due to chance is less than 5%?*  Sample sizes have been calculated to detect a difference of x% and y%. |  |  |  |  |
| **Total Score 14/14 = 100 %** | | | | |

| **Warenczak et al. 2020** | | | | |
| --- | --- | --- | --- | --- |
| **Reporting** | **YES** | **NO** | **UTD** | **Notes/Justification** |
| *1. Is the hypothesis/aim/objective of the study clearly described?* | X |  |  | The aim, objective and hypothesis are clearly described. |
| *2. Are the main outcomes to be measured clearly described in the Introduction or Methods section?*  If the main outcomes are first mentioned in the Results section, the question should be answered no. | X |  |  | Clearly described in the Introduction section. |
| *3. Are the characteristics of the patients included*  *in the study clearly described?*  In cohort studies and trials, inclusion and/or exclusion criteria should be given. In case‐control studies, a case‐definition and the source for controls should be given. | X |  |  | Clearly described in the Methods section. |
| *4. Are the interventions of interest clearly described?*  Treatments and placebo (where relevant) that are to be compared should be clearly described. |  |  |  |  |
| *5. Are the distributions of principal confounders in each group of subjects to be compared clearly described?*  A list of principal confounders is provided. | X |  |  | Reported in the Methods section. |
| *6. Are the main findings of the study clearly described?*  Simple outcome data (including denominators and numerators) should be reported for all major findings so that the reader can check the major analyses and conclusions. (This question does not cover statistical tests  which are considered below). | X |  |  | Reported in the Results section and Table 1,2,3,4. |
| *7. Does the study provide estimates of the random variability in the data for the main outcomes?*  In non-normally distributed data the inter‐quartile range of results should be reported. In normally distributed data the standard error, standard deviation or confidence intervals should be reported. If the distribution of the data is not described, it must be assumed that the estimates used were appropriate and the question should be answered yes. | X |  |  | Standard deviation has been reported. |
| *8. Have all important adverse events that may be a consequence of the intervention been reported?*  This should be answered yes if the study demonstrates that there was a comprehensive attempt to measure adverse events. (A list of possible adverse events is provided). |  |  |  |  |
| *9. Have the characteristics of patients lost to follow‐up been described?*  This should be answered yes where there were no losses to follow‐up or where losses to follow‐up were so small that findings would be unaffected by their inclusion. This should be answered NO where a study does not report the number of patients lost to follow‐up. |  |  |  |  |
| *10. Have actual probability values been reported ( e.g. 0.035 rather than < 0.05) for the main outcomes except where the probability value is less than 0.001?* | X |  |  | Actual probability values have been reported. |
| **External Validity** | **YES** | **NO** | **UTD** | **Notes/Justification** |
| *11. Were the subjects asked to participate in the study representative of the entire population from which they were recruited?*  The study must identify the source population for patients and describe how the patients were selected. Patients would be representative if they comprised the entire source population, an unselected sample of consecutive patients, or a random sample. Random sampling is only feasible where a list of all members of the relevant population exists. Where a study does not report the proportion of the source population from which the patients are derived, the question should be answered as unable to determine. | X |  |  | Participants were representative. |
| *12. Were those subjects who were prepared to participate representative of the entire population from which they were recruited?*  The proportion of those asked who agreed should be stated. Validation that the sample was representative would include demonstrating that the distribution of the main confounding factors was the same in the study sample and the source population. | X |  |  | The sample is representative. |
| *13. Were the staff, places, and facilities where the patients were treated, representative of the treatment the majority of patients receive?*  For the question to be answered yes the study should demonstrate that the intervention was representative of that in use in the source population. The question should be answered no if, for example, the intervention was  undertaken in a specialist centre unrepresentative of the hospitals most of the source population would attend. |  |  |  |  |
| **Internal validity – bias** | **YES** | **NO** | **UTD** | **Notes/Justification** |
| *14. Was an attempt made to blind study subjects to the intervention they have received?*  For studies where the patients would have no way of knowing which intervention they received, this should be answered yes. |  |  |  |  |
| *15. Was an attempt made to blind those measuring the main outcomes of the intervention?* |  |  |  |  |
| *16. If any of the results of the study were based on “data dredging”, was this made clear?*  Any analyses that had not been planned at the outset of the study should be clearly indicated. If no retrospective unplanned subgroup analyses were reported, then answer yes. | X |  |  | No retrospective unplanned subgroup analyses were reported. |
| *17. In trials and cohort studies, do the analyses adjust for different lengths of follow‐up of patients, or in case-control studies, is the time period between the intervention and outcome the same for cases and controls?*  Where follow‐up was the same for all study patients the answer should yes.  If different lengths of follow‐up were adjusted for by, for example, survival analysis the answer should be yes. Studies where differences in follow‐up are ignored should be answered no. |  |  |  |  |
| *18. Were the statistical tests used to assess the main outcomes appropriate?*  The statistical techniques used must be appropriate to the data. For example, Non-parametric methods should be used for small sample sizes. Where little statistical analysis has been undertaken but where there is no evidence of bias, the question should be answered yes. If the distribution of the data (normal or not) is not described it must be assumed that the estimates used were appropriate and the question should be answered yes. | X |  |  | Statistics were appropriate and correctly reported. |
| *19. Was compliance with the intervention/s reliable?*  Where there was non compliance with the allocated treatment or where there was contamination of one group, the question should be answered no.  For studies where the effect of any misclassification was likely to bias any association to the null, the question should be answered yes. |  |  |  |  |
| *20. Were the main outcome measures used accurate (valid and reliable)?*  For studies where the outcome measures are clearly described, the question should be answered yes. For studies which refer to other work or that demonstrates the outcome measures are accurate, the question should be  answered as yes. | X |  |  | Outcome measures clearly described. |
| **Internal validity ‐ confounding (selection bias)** | **YES** | **NO** | **UTD** | **Notes/Justification** |
| *21. Were the patients in different intervention groups (trials and cohort studies) or were the cases and controls (case‐control studies) recruited from the same population?*  For example, patients for all comparison groups should be selected from the same hospital. The question should be answered unable to determine for cohort and case-control studies where there is no information concerning the source of patients included in the study. | X |  |  | Patients were recruited from the same hospital. |
| *22. Were study subjects in different intervention groups (trials and cohort- studies) or were the cases and controls (case‐control studies) recruited over the same period of time?*  For a study which does not specify the time period over which patients were recruited, the question should be answered as unable to determine. | X |  |  | Patients were recruited over the same time period. |
| *23. Were study subjects randomised to intervention groups?*  Studies which state that subjects were randomized should be answered yes except where method of randomisation would not ensure random allocation.  For example, alternate allocation would score no because it is predictable. |  |  |  |  |
| *24. Was the randomised intervention assignment concealed from both patients and health care staff until recruitment was complete and irrevocable?*  All non‐randomised studies should be answered no. If assignment was concealed from patients but not from staff, it should be answered no. |  |  |  |  |
| *25. Was there adequate adjustment for confounding in the analyses from which the main findings were drawn?*  This question should be answered no for trials if: the main conclusions of the study were based on analyses of treatment rather than intention to treat; the distribution of known confounders in the different treatment groups was  not described; or the distribution of known confounders differed between the treatment groups but was not taken into account in the analyses. In non-randomized studies if the effect of the main confounders was not investigated or confounding was demonstrated but no adjustment was made in the final analyses the question should be answered as no. |  |  |  |  |
| *26. Were losses of patients to follow‐up taken into account?*  If the numbers of patients lost to follow‐up are not reported, the question should be answered as unable to determine. If the proportion lost to follow-up was too small to affect the main findings, the question should be  answered yes. |  |  |  |  |
| **Power** |  |  |  |  |
| *27. Did the study have sufficient power to detect a clinically important effect where the probability value for a difference being due to chance is less than 5%?*  Sample sizes have been calculated to detect a difference of x% and y%. |  |  |  |  |
| **Total Score 14/14 = 100 %** | | | | |
| **Winther et al. 2021** | | | | |
| **Reporting** | **YES** | **NO** | **UTD** | **Notes/Justification** |
| *1. Is the hypothesis/aim/objective of the study clearly described?* | X |  |  | The aim, objective and hypothesis are clearly described. |
| *2. Are the main outcomes to be measured clearly described in the Introduction or Methods section?*  If the main outcomes are first mentioned in the Results section, the question should be answered no. | X |  |  | Clearly described in the Introduction section. |
| *3. Are the characteristics of the patients included*  *in the study clearly described?*  In cohort studies and trials, inclusion and/or exclusion criteria should be given. In case‐control studies, a case‐definition and the source for controls should be given. | X |  |  | Clearly described in the Methods section. |
| *4. Are the interventions of interest clearly described?*  Treatments and placebo (where relevant) that are to be compared should be clearly described. | X |  |  |  |
| *5. Are the distributions of principal confounders in each group of subjects to be compared clearly described?*  A list of principal confounders is provided. | X |  |  | Reported in the Methods section. |
| *6. Are the main findings of the study clearly described?*  Simple outcome data (including denominators and numerators) should be reported for all major findings so that the reader can check the major analyses and conclusions. (This question does not cover statistical tests  which are considered below). | X |  |  |  |
| *7. Does the study provide estimates of the random variability in the data for the main outcomes?*  In non-normally distributed data the inter‐quartile range of results should be reported. In normally distributed data the standard error, standard deviation or confidence intervals should be reported. If the distribution of the data is not described, it must be assumed that the estimates used were appropriate and the question should be answered yes. | X |  |  |  |
| *8. Have all important adverse events that may be a consequence of the intervention been reported?*  This should be answered yes if the study demonstrates that there was a comprehensive attempt to measure adverse events. (A list of possible adverse events is provided). |  |  | X |  |
| *9. Have the characteristics of patients lost to follow‐up been described?*  This should be answered yes where there were no losses to follow‐up or where losses to follow‐up were so small that findings would be unaffected by their inclusion. This should be answered NO where a study does not report the number of patients lost to follow‐up. | X |  |  |  |
| *10. Have actual probability values been reported ( e.g. 0.035 rather than < 0.05) for the main outcomes except where the probability value is less than 0.001?* | X |  |  | Actual probability values have been reported. |
| **External Validity** | **YES** | **NO** | **UTD** | **Notes/Justification** |
| *11. Were the subjects asked to participate in the study representative of the entire population from which they were recruited?*  The study must identify the source population for patients and describe how the patients were selected. Patients would be representative if they comprised the entire source population, an unselected sample of consecutive patients, or a random sample. Random sampling is only feasible where a list of all members of the relevant population exists. Where a study does not report the proportion of the source population from which the patients are derived, the question should be answered as unable to determine. |  |  | X |  |
| *12. Were those subjects who were prepared to participate representative of the entire population from which they were recruited?*  The proportion of those asked who agreed should be stated. Validation that the sample was representative would include demonstrating that the distribution of the main confounding factors was the same in the study sample and the source population. |  |  | X |  |
| *13. Were the staff, places, and facilities where the patients were treated, representative of the treatment the majority of patients receive?*  For the question to be answered yes the study should demonstrate that the intervention was representative of that in use in the source population. The question should be answered no if, for example, the intervention was  undertaken in a specialist centre unrepresentative of the hospitals most of the source population would attend. | X |  |  |  |
| **Internal validity – bias** | **YES** | **NO** | **UTD** | **Notes/Justification** |
| *14. Was an attempt made to blind study subjects to the intervention they have received?*  For studies where the patients would have no way of knowing which intervention they received, this should be answered yes. |  |  | X |  |
| *15. Was an attempt made to blind those measuring the main outcomes of the intervention?* |  | X |  |  |
| *16. If any of the results of the study were based on “data dredging”, was this made clear?*  Any analyses that had not been planned at the outset of the study should be clearly indicated. If no retrospective unplanned subgroup analyses were reported, then answer yes. | X |  |  |  |
| *17. In trials and cohort studies, do the analyses adjust for different lengths of follow‐up of patients, or in case-control studies, is the time period between the intervention and outcome the same for cases and controls?*  Where follow‐up was the same for all study patients the answer should yes.  If different lengths of follow‐up were adjusted for by, for example, survival analysis the answer should be yes. Studies where differences in follow‐up are ignored should be answered no. | X |  |  |  |
| *18. Were the statistical tests used to assess the main outcomes appropriate?*  The statistical techniques used must be appropriate to the data. For example, Non-parametric methods should be used for small sample sizes. Where little statistical analysis has been undertaken but where there is no evidence of bias, the question should be answered yes. If the distribution of the data (normal or not) is not described it must be assumed that the estimates used were appropriate and the question should be answered yes. | X |  |  | Statistics were appropriate and correctly reported. |
| *19. Was compliance with the intervention/s reliable?*  Where there was non compliance with the allocated treatment or where there was contamination of one group, the question should be answered no.  For studies where the effect of any misclassification was likely to bias any association to the null, the question should be answered yes. | X |  |  |  |
| *20. Were the main outcome measures used accurate (valid and reliable)?*  For studies where the outcome measures are clearly described, the question should be answered yes. For studies which refer to other work or that demonstrates the outcome measures are accurate, the question should be  answered as yes. | X |  |  | Outcome measures clearly described. |
| **Internal validity ‐ confounding (selection bias)** | **YES** | **NO** | **UTD** | **Notes/Justification** |
| *21. Were the patients in different intervention groups (trials and cohort studies) or were the cases and controls (case‐control studies) recruited from the same population?*  For example, patients for all comparison groups should be selected from the same hospital. The question should be answered unable to determine for cohort and case-control studies where there is no information concerning the source of patients included in the study. | X |  |  |  |
| *22. Were study subjects in different intervention groups (trials and cohort- studies) or were the cases and controls (case‐control studies) recruited over the same period of time?*  For a study which does not specify the time period over which patients were recruited, the question should be answered as unable to determine. | X |  |  |  |
| *23. Were study subjects randomised to intervention groups?*  Studies which state that subjects were randomized should be answered yes except where method of randomisation would not ensure random allocation.  For example, alternate allocation would score no because it is predictable. | X |  |  |  |
| *24. Was the randomised intervention assignment concealed from both patients and health care staff until recruitment was complete and irrevocable?*  All non‐randomised studies should be answered no. If assignment was concealed from patients but not from staff, it should be answered no. |  |  | X |  |
| *25. Was there adequate adjustment for confounding in the analyses from which the main findings were drawn?*  This question should be answered no for trials if: the main conclusions of the study were based on analyses of treatment rather than intention to treat; the distribution of known confounders in the different treatment groups was  not described; or the distribution of known confounders differed between the treatment groups but was not taken into account in the analyses. In non-randomized studies if the effect of the main confounders was not investigated or confounding was demonstrated but no adjustment was made in the final analyses the question should be answered as no. | X |  |  |  |
| *26. Were losses of patients to follow‐up taken into account?*  If the numbers of patients lost to follow‐up are not reported, the question should be answered as unable to determine. If the proportion lost to follow-up was too small to affect the main findings, the question should be  answered yes. | X |  |  |  |
| **Power** |  |  |  |  |
| *27. Did the study have sufficient power to detect a clinically important effect where the probability value for a difference being due to chance is less than 5%?*  Sample sizes have been calculated to detect a difference of x% and y%. |  |  | X |  |
| **Total Score 20/27 = 74.0 %** | | | | |

| **Zeng et al. 2014** | | | | |
| --- | --- | --- | --- | --- |
|  | | | | |
| **Reporting** | **YES** | **NO** | **UTD** | **Notes/Justification** |
| *1. Is the hypothesis/aim/objective of the study clearly described?* | X |  |  | The aim is clearly described. |
| *2. Are the main outcomes to be measured clearly described in the Introduction or Methods section?*  If the main outcomes are first mentioned in the Results section, the question should be answered no. | X |  |  | Clearly described in the Methods section. |
| *3. Are the characteristics of the patients included in the study clearly described?*  In cohort studies and trials, inclusion and/or exclusion criteria should be given. In case‐control studies, a case‐definition and the source for controls should be given. | X |  |  | Clearly described in the Methods section (2.1). |
| *4. Are the interventions of interest clearly described?*  Treatments and placebo (where relevant) that are to be compared should be clearly described. | X |  |  | The interventions are clearly described in the Method section 2.3. |
| *5. Are the distributions of principal confounders in each group of subjects to be compared clearly described?*  A list of principal confounders is provided. | X |  |  | Confounders have been described. |
| *6. Are the main findings of the study clearly described?*  Simple outcome data (including denominators and numerators) should be reported for all major findings so that the reader can check the major analyses and conclusions. (This question does not cover statistical tests  which are considered below). | X |  |  | Main findings have been clearly described. |
| *7. Does the study provide estimates of the random variability in the data for the main outcomes?*  In non-normally distributed data the inter‐quartile range of results should be reported. In normally distributed data the standard error, standard deviation or confidence intervals should be reported. If the distribution of the data is not described, it must be assumed that the estimates used were appropriate and the question should be answered yes. | X |  |  | Variability has been reported. |
| *8. Have all important adverse events that may be a consequence of the intervention been reported?*  This should be answered yes if the study demonstrates that there was a comprehensive attempt to measure adverse events. (A list of possible adverse events is provided). |  | X |  |  |
| *9. Have the characteristics of patients lost to follow‐up been described?*  This should be answered yes where there were no losses to follow‐up or where losses to follow‐up were so small that findings would be unaffected by their inclusion. This should be answered NO where a study does not report the number of patients lost to follow‐up. | X |  |  | Lost to follow‐up have been described. |
| *10. Have actual probability values been reported (e.g. 0.035 rather than < 0.05) for the main outcomes except where the probability value is less than 0.001?* | X |  |  | Probability values have been reported. |
| **External Validity** | **YES** | **NO** | **UTD** | **Notes/Justification** |
| *11. Were the subjects asked to participate in the study representative of the entire population from which they were recruited?*  The study must identify the source population for patients and describe how the patients were selected. Patients would be representative if they comprised the entire source population, an unselected sample of consecutive patients, or a random sample. Random sampling is only feasible where a list of all members of the relevant population exists. Where a study does not report the proportion of the source population from which the patients are derived, the question should be answered as unable to determine. |  |  | X |  |
| *12. Were those subjects who were prepared to participate representative of the entire population from which they were recruited?*  The proportion of those asked who agreed should be stated. Validation that the sample was representative would include demonstrating that the distribution of the main confounding factors was the same in the study sample and the source population. | X |  |  | Analyses assessing the comparability of the two groups indicated that they were well matched with regard to baseline descriptors. Further analyses on the patient demographics between the two groups also indicated no significant differences (P > 0.05). |
| *13. Were the staff, places, and facilities where the patients were treated, representative of the treatment the majority of patients receive?*  For the question to be answered yes the study should demonstrate that the intervention was representative of that in use in the source population. The question should be answered no if, for example, the intervention was  undertaken in a specialist centre unrepresentative of the hospitals most of the source population would attend. | X |  |  | Patients trained at home, Exercise adherence was monitored through daily self-report  card record by patients or their training partners as a measure of  compliance. Daily self-report card was filled when the patients had  completed the exercise program and collected when they were  subsequent interviewed in our outpatient department once a  week. Patients performed their exercise training movement and  corrected their neglect postures by physiotherapists. They also  were asked individuality about their adherence to the instructed  home exercises and instructed how to avoid tumbling or other  adverse events during exercise program. Any problems were  resolved in the weekly interview time. |
| **Internal validity – bias** | **YES** | **NO** | **UTD** | **Notes/Justification** |
| *14. Was an attempt made to blind study subjects to the intervention they have received?*  For studies where the patients would have no way of knowing which intervention they received, this should be answered yes. | X |  |  | This study was a single-blind randomized controlled trial |
| *15. Was an attempt made to blind those measuring the main outcomes of the intervention?* | X |  |  | Outcome measures were evaluated in the  Rehabilitation Department of the Chaonan Minsheng Hospital by  two spine surgeons who did not participate in joint surgery. They  have previously been trained in all the outcome tools and were  blinded to the study. |
| *16. If any of the results of the study were based on “data dredging”, was this made clear?*  Any analyses that had not been planned at the outset of the study should be clearly indicated. If no retrospective unplanned subgroup analyses were reported, then answer yes. | X |  |  | No dredging data is mentioned. |
| *17. In trials and cohort studies, do the analyses adjust for different lengths of follow‐up of patients, or in case-control studies, is the time period between the intervention and outcome the same for cases and controls?*  Where follow‐up was the same for all study patients the answer should yes.  If different lengths of follow‐up were adjusted for by, for example, survival analysis the answer should be yes. Studies where differences in follow‐up are ignored should be answered no. | X |  |  | All patients were evaluated within three days after they were  allocated into the study, within three days preoperatively and  Week 13 and Week 26 postoperatively. |
| *18. Were the statistical tests used to assess the main outcomes appropriate?*  The statistical techniques used must be appropriate to the data. For example, Non-parametric methods should be used for small sample sizes. Where little statistical analysis has been undertaken but where there is no evidence of bias, the question should be answered yes. If the distribution of the data (normal or not) is not described it must be assumed that the estimates used were appropriate and the question should be answered yes. | X |  |  | Statistics were appropriate and correctly reported. |
| *19. Was compliance with the intervention/s reliable?*  Where there was no compliance with the allocated treatment or where there was contamination of one group, the question should be answered no.  For studies where the effect of any misclassification was likely to bias any association to the null, the question should be answered yes. | X |  |  | Compliance with the interventions was reliable. |
| *20. Were the main outcome measures used accurate (valid and reliable)?*  For studies where the outcome measures are clearly described, the question should be answered yes. For studies which refer to other work or that demonstrates the outcome measures are accurate, the question should be  answered as yes. | X |  |  | Outcome measures were accurate. |
| **Internal validity ‐ confounding (selection bias)** | **YES** | **NO** | **UTD** | **Notes/Justification** |
| *21. Were the patients in different intervention groups (trials and cohort studies) or were the cases and controls (case‐control studies) recruited from the same population?*  For example, patients for all comparison groups should be selected from the same hospital. The question should be answered unable to determine for cohort and case-control studies where there is no information concerning the source of patients included in the study. | X |  |  | All patients received a primary THA surgery in the Orthopaedic  Department of the Chaonan Mingsheng Hospital of Shantou  between December 2008 and December 2011. |
| *22. Were study subjects in different intervention groups (trials and cohort- studies) or were the cases and controls (case‐control studies) recruited over the same period of time?*  For a study which does not specify the time period over which patients were recruited, the question should be answered as unable to determine. | X |  |  | Patients were recruited over the same time period. |
| *23. Were study subjects randomised to intervention groups?*  Studies which state that subjects were randomized should be answered yes except where method of randomisation would not ensure random allocation.  For example, alternate allocation would score no because it is predictable. | X |  |  | Participants were randomly allocated to one of the groups. The patients were randomly divided  into two groups using a table of random numbers by an orthopedic  nurse. Even numbers were allocated to the CG and odd numbers to  the TG. |
| *24. Was the randomised intervention assignment concealed from both patients and health care staff until recruitment was complete and irrevocable?*  All non‐randomised studies should be answered no. If assignment was concealed from patients but not from staff, it should be answered no. |  | X |  | Physioterapists know the assignement as they have to educate patient to train. |
| *25. Was there adequate adjustment for confounding in the analyses from which the main findings were drawn?*  This question should be answered no for trials if: the main conclusions of the study were based on analyses of treatment rather than intention to treat; the distribution of known confounders in the different treatment groups was  not described; or the distribution of known confounders differed between the treatment groups but was not taken into account in the analyses. In non-randomized studies if the effect of the main confounders was not investigated or confounding was demonstrated but no adjustment was made in the final analyses the question should be answered as no. | X |  |  | Distribution of potentially confounders was the same between the groups. |
| *26. Were losses of patients to follow‐up taken into account?*  If the numbers of patients lost to follow‐up are not reported, the question should be answered as unable to determine. If the proportion lost to follow-up was too small to affect the main findings, the question should be answered yes. | X |  |  | In Fig.1 there is the whole flow chart |
| **Power** | **YES** | **NO** | **UTD** | **Notes/Justification** |
| *27. Did the study have sufficient power to detect a clinically important effect where the probability value for a difference being due to chance is less than 5%?*  Sample sizes have been calculated to detect a difference of x% and y%. |  |  | X | No statistical power calculations have been reported. |
| **Total Score 23/27 = 85.1** | | | | |
